# Early-Life Resource Scarcity in Mice Does Not Alter Adult Corticosterone or Preovulatory Luteinizing Hormone Surge Responses to Acute Psychosocial Stress

**DOI:** 10.1523/ENEURO.0125-24.2024

**Published:** 2024-07-26

**Authors:** Amanda G. Gibson, Suzanne M. Moenter

**Affiliations:** ^1^Neurocience Graduate Program, University of Michigan, Ann Arbor, Michigan 48109-5622; ^2^Departments of Molecular and Integrative Physiology, University of Michigan, Ann Arbor, Michigan 48109-5622; ^3^Internal Medicine, University of Michigan, Ann Arbor, Michigan 48109-5622; ^4^Obstetrics and Gynecology, University of Michigan, Ann Arbor, Michigan 48109-5622

**Keywords:** early-life stress, GABA, GnRH, LH, psychosocial stress, reproduction

## Abstract

Early-life stressors can affect reproductive development and change responses to adult stress. We tested if resource scarcity in the form of limited bedding and nesting (LBN) from postnatal days (PND) 4 to 11 delayed sexual maturation in male and female mice and/or altered the response to an acute, layered, psychosocial stress (ALPS) in adulthood. Contrary to the hypotheses, age and mass at puberty were unaffected by the present application of LBN. Under basal conditions and after ALPS, corticosterone concentrations in males, diestrous females, and proestrous females reared in standard (STD) or LBN environments were similar. ALPS disrupts the luteinizing hormone (LH) surge in most mice when applied on the morning of proestrus; this effect was not changed by resource scarcity. In this study, the paucity of effects in the offspring may relate to a milder response of CBA dams to the paradigm. While LBN dams exited the nest more often and their offspring were smaller than STD-reared offspring on PND11, dam corticosterone concentrations were similar on PND11. To test if ALPS disrupts the LH surge by blunting the increase in excitatory GABAergic input to gonadotropin-releasing hormone (GnRH) neurons on the afternoon of proestrus, we conducted whole-cell voltage-clamp recordings. The frequency of GABAergic postsynaptic currents in GnRH neurons was not altered by LBN, ALPS, or their interaction. It remains possible that ALPS acts at afferents of GnRH neurons, changes response of GnRH neurons to input, and/or alters pituitary responsiveness to GnRH and that a more pronounced resource scarcity would affect the parameters studied.

## Significance Statement

The stress and reproductive neuroendocrine systems interact, and early-life stress has reproductive consequences in humans. This study in mice rejected the hypotheses that an early-life stress, limited bedding and nesting (LBN), would delay sexual maturation and alter the response to an acute, layered, psychosocial stress (ALPS) in adulthood. ALPS disrupts the proestrous luteinizing hormone surge, which is critical for ovulation; this disruption is not altered by LBN. To assess a possible mechanism for this disruption, we conducted electrophysiological recording of gonadotropin-releasing hormone neurons to test if ALPS reduces excitatory GABAergic input to these cells. The frequency of GABAergic input was similar among groups, suggesting that LBN and ALPS act elsewhere in the broader neuroendocrine network controlling reproduction.

## Introduction

The neuroendocrine systems regulating stress and reproduction are important for organisms to respond to their environments and ensure the continuation of the species. Gonadotropin-releasing hormone (GnRH) neurons in the hypothalamus integrate many inputs and serve as the final common central output to the downstream reproductive axis. GnRH is released in a pulsatile manner and acts on the pituitary to stimulate the release of luteinizing hormone (LH) and follicle-stimulating hormone (Wildt et al., 1981), which activate gametogenesis and steroidogenesis. In males and during most of the female reproductive cycle, sex steroids exert negative feedback to reduce overall GnRH and LH concentrations. Sustained elevated estradiol concentrations in the preovulatory period (proestrus in rodents) exert positive feedback to induce prolonged surges of GnRH and LH release (Sarkar et al., 1976; Moenter et al., 1992; Pau et al., 1993; Evans et al., 1994). The LH surge triggers ovulation (Fevold et al., 1931). The organization of the neuroendocrine stress axis is similar, with corticotropin-releasing hormone release from hypothalamic neurons stimulating secretion of adrenocorticotropic hormone from the pituitary and the adrenal cortex producing glucocorticoids that provide negative feedback at the brain and pituitary (Smith and Vale, 2006).

Neuroendocrine axes interact with one another. In humans, stress has clinical effects on the reproductive system with social consequences (Chrousos et al., 1998). Perceived stress in adulthood can reduce likelihood of both natural conception and pregnancy via assisted reproductive technology (Klonoff-Cohen et al., 2001; Matthiesen et al., 2011; Akhter et al., 2016). Early-life stress can disrupt reproductive development, though whether stress delays or advances puberty appears to depend on the type and timing of stressor (Foster et al., 2008; Mendle et al., 2011, 2016; Boynton-Jarrett and Harville, 2012). In humans, it is challenging to disentangle the independent effects of early-life and adult stress on reproduction from each other and from other factors and to understand how a history of early-life stress affects the response to adult stress. These questions can be better addressed using animal models.

A common model for early-life stress in rodents is the limited bedding and nesting (LBN) paradigm in which dams and pups are moved to a low-resource environment for several days; this alters the way the dam interacts with the pup without ongoing investigator interference (Rice et al., 2008; Walker et al., 2017). Effects of LBN on reproductive outcomes have been mixed in both mice and rats. LBN delayed the age at vaginal opening, an external indicator of puberty, in some studies (Manzano Nieves et al., 2019; Knop et al., 2020), advanced it in another (Davis et al., 2020), and had no effect in others (Knop et al., 2019; Eck et al., 2020). Some studies found a delay in preputial separation in males (Knop et al., 2019, 2020; Davis et al., 2020). There are many models of adult stress including psychosocial (e.g., restraint), metabolic (e.g., hypoglycemia), and immune (e.g., endotoxin; Toufexis et al., 2014; McCosh et al., 2019; Phumsatitpong et al., 2021). In adult mice, exposure to an acute, layered, psychosocial stress (ALPS) paradigm on the morning of proestrus disrupts the preovulatory (proestrous) LH surge in most mice (Wagenmaker and Moenter, 2017). The mechanisms by which ALPS leads to this disruption are not known, but do not include disrupting the preovulatory estradiol rise. With regard to possible mechanisms, the rate of GABAergic transmission to GnRH neurons (which is excitatory in these cells; DeFazio et al., 2002) increases on the afternoon of proestrus (Adams et al., 2018) and around the onset of the estradiol-induced LH surge (Christian and Moenter, 2007). The increase in GABAergic postsynaptic currents (PSCs) could account for the increased activity in GnRH neurons during the surge. Stressors alter GABAergic signaling within other hypothalamic regions. For example, restraint stress reduced the frequency of GABA transmission in parvocellular neurons in the paraventricular nucleus (Verkuyl et al., 2005). A reduction in GABAergic input to GnRH neurons following ALPS could help explain the disruption to the LH surge by this psychosocial stressor.

Early-life stress can affect the response to adult stressors, though whether animals are more susceptible or resilient depends on the type of stressors and the outcomes measured (Peña et al., 2019). In the present study, we investigated the effect of LBN on reproductive maturation and adult response to stress in male and female mice. We also tested the hypothesis that ALPS disrupts the LH surge by reducing the excitatory input to GnRH neurons on the afternoon of proestrus and determined if LBN altered the ability of ALPS to disrupt the LH surge.

## Materials and Methods

### Animals

All animal procedures were approved by the University of Michigan Institutional Animal Care and Use Committee. Mice had *ad libitum* access to water and food; breeders were provided Teklad 2919 chow (Inotiv) through weaning. After weaning, mice were maintained on Teklad 2916 (Inotiv). The vivarium followed a 14/10 h light/dark cycle (lights on at 3 A.M. EST); at times, dim red light (<10 lux) was used overnight. A male GnRH-GFP [Tg(Gnrh1-EGFP)51Sumo MGI:6158457, C57Bl6/J background] mouse expressing GFP under the control of the GnRH promoter (Suter et al., 2000) was housed with one or two female CBA mice (strain 000656; The Jackson Laboratory) in cages containing 550–650 ml of corn cob bedding (Bed-o’Cobs ¼, The Andersons). The female body mass was measured on the day of pairing with the male. Females were examined for vaginal plugs for up to 5 d after pairing, and their body mass was measured again around hypothetical gestational days 12–14. An increased body mass (>110% of the initial mass) was used as an initial confirmation of pregnancy, at which point females were moved to individual cages with 550–650 ml bedding and a Nestlet (Ancare) and plastic igloo (Bio-Serv) for enrichment.

### Experimental design

This study assessed the independent effects of and interactions between early-life and adult stress on reproduction. Pilot studies were conducted to examine timing of LBN treatment and effects of litter size. Based on these, offspring from the first litter of a dam were studied, and the litter size was normalized to 5–8 pups per mouse as small litters had early vaginal opening, whereas large litters were delayed as has been observed (Caron et al., 2012). Dams and litters were assigned to either standard (STD) housing or LBN treatment as an early-life stress. The body mass of individual offspring was monitored after early-life treatment through adulthood. To determine if LBN affected external markers of reproductive maturation, we monitored the offspring for preputial separation (male) or vaginal opening and first appearance of estrus, as indicated by cornification of the vaginal epithelium (female; Korenbrot et al., 1977). Estrous cycles of female offspring in adulthood were monitored to test if cycles were disrupted by LBN.

To evaluate if LBN alters the response to an adult stressor, adult offspring were assigned to either control (CON) or ALPS treatment, resulting in four experimental groups (STD-CON, STD-ALPS, LBN-CON, LBN-ALPS). Block assignment within litters and minimization strategies were used to balance adult treatments. Serum corticosterone concentration was assessed in males and in diestrous and proestrous females to determine if cycle stage altered baseline corticosterone concentration or the corticosterone rise in response to stress. In proestrous females, we also assessed if a history of LBN affected the disruptions of the LH surge by ALPS. To investigate the mechanisms by which ALPS disrupts the surge, we monitored the GABAergic transmission to GnRH neurons using whole-cell voltage-clamp around the time of the typical LH surge.

### LBN paradigm

LBN was used to model early-life stress. STD cages contained 550–650 ml of bedding and one full 5 × 5 cm square of Nestlet material. LBN cages contained ∼100 ml of bedding, enough to cover the floor of the cage with a single layer, and a 2.5 × 5 cm piece of Nestlet. LBN cages were fitted with a wire mesh platform ∼1.5 cm above the cage floor (allFENZ 23 Gauge Hardware Cloth, Home Depot). Food was provided in a small container placed on the floor of the cage to prevent pups crawling into the food hopper from the raised platform and to permit unobstructed video monitoring of dam behavior. Food was replenished daily.

Females were monitored for births daily before lights off beginning 19 d after observation of vaginal plug or after pairing with a male if no plug was noted. The day of birth was designated postnatal day (PND) 0. Pups (CBB6/F1 hybrids) from litters born within 1 d of each other were cross-fostered if needed to standardize litter sizes to 5–8 pups by PND2. The STD or LBN treatment period began on PND4. From PND4 to 11, animals were undisturbed in their respective treatment cages apart from transferring for video monitoring and daily replenishment of food and water. On the morning of PND11, a tail blood sample was collected from the dam for assessment of serum corticosterone concentration, and then all dams and litters were transferred to clean cages containing 550–650 ml of bedding and a 5 × 5 cm Nestlet until weaning at PND21. All offspring were weaned with same-sex littermates into STD cages with the addition of a plastic igloo; the igloo was removed on PND28.

### Dam behavior monitoring

Continuous video monitoring occurred for either 24 h from the morning of PND5 to the morning of PND6, 48 h from the morning of PND4 to 6, or for the duration of the early-life treatment from PND4 to 11. The number of exits made by the dam from the nest was manually counted for 1 h periods beginning at zeitgeber times (ZT) 1 (4 A.M. EST), ZT15 (6 P.M. EST), and ZT19 (10 P.M. EST). The average number of exits and amount of time off the nest per hour was calculated for each dam. Recordings were conducted using FFmpeg (version 4.3.1) on the following computers: MacBook Air (Mid 2009, running OSX El Capitan, Version 10.11.6, with a 2.13 GHz Intel Core 2 Duo), MacBook Pro (Mid 2012, running macOS Catalina, Version 10.15.7, with a 2.5 GHz Dual-Core Intel Core i5), MacBook Pro (Early 2015, running macOS Catalina, Version 10.15.7, with a 2.7 GHz Dual-Core Intel Core i5), Mac mini (2018, running macOS Sonoma, Version 14.0, with a 3 GHz 6-Core Intel Core i5), and a MacBook Pro (Late 2013, running macOS Mojave, Version 10.14.6, with a 2.3 GHz Intel Core i7).

### Body mass

Dam mass was recorded on PND4 (start of paradigm), PND11 (end of paradigm), and PND21 (pup weaning). Before placing pups into the new cage at the start of the paradigm, the average mass for each litter was recorded to identify any potential outliers. At the end of the paradigm, on the morning of PND11, pups were ear-marked and identified with Sharpie markings on the tail to permit individual tracking of each pup. Pup mass was recorded daily through PND24 and then weekly through PND70 and as indicated below.

### Offspring maturation

Beginning at weaning, offspring were checked daily for preputial separation or vaginal opening (Ojeda et al., 1986; J. E. Nelson et al., 1990). Body mass was recorded on the day of preputial separation/vaginal opening. After vaginal opening, female mice underwent daily vaginal lavage to identify the day of first estrus based on cornification of vaginal epithelium; body mass was recorded on the day of first estrus. Anogenital distance (AGD) was measured with digital calipers (Marathon) for 3 consecutive days during the 10th postnatal week and averaged for each animal.

### Adult female estrous cycles

To study the effect of LBN on estrous cycles, we monitored the vaginal lavages daily from PND70 to 90 (Goldman et al., 2007; Caligioni, 2009; Byers et al., 2012). The number of cycles (defined as the number of days in proestrus preceded by diestrus or estrus per 21 d monitoring period), the mean cycle length (days between successive proestrous stages), and the percentage of days in diestrus, estrus, and proestrus are reported.

### ALPS paradigm

Animals exposed to adult stress were habituated for at least 2 weeks beforehand to tail and general handling. Female mice were studied either on the first day of diestrus or on proestrus, as determined by vaginal cytology and confirmed by uterine mass (diestrus, <100 mg; proestrus, >125 mg) measured the same day. An ALPS paradigm that disrupts the proestrous LH surge in most females was used (Wagenmaker and Moenter, 2017); both male (>PND84) and female (≥PND90) mice were studied. At 0 h (6.5 h after lights on), a tail blood sample (∼30 μl) was collected, and the serum was stored for assessment of corticosterone. In females, an additional 6 μl of whole blood was obtained for LH measurement. CON mice were only removed from their home cage for tail blood sampling at times equivalent to sampling in stressed mice and remained in the vivarium. ALPS mice were placed individually into a new cage and transferred to a new room. At 1 h, the mice were placed in a restraint tube (Braintree Scientific, flat-bottom restrainer small or Tailveiner-150 restrainer). At 3 h, restrained mice were exposed to a component of red fox (*Vulpes vulpes*) urine as a predator odor (2,3,5-trimethyl-3-thiazoline; ∼6 nmol; Contech Enterprises). Tail blood samples were collected at the end of the paradigm (5 h, 2:30 P.M. EST, 2.5 h before lights out) for corticosterone and LH in females. For females in diestrus, an additional tail blood sample for LH was collected at the time of lights out (5 P.M. EST). For those in proestrus, additional tail blood samples to monitor the effect of LBN and/or ALPS on the LH surge were taken hourly from 3 to 7 P.M. EST, unless noted. Animals were considered to exhibit an LH surge if any PM value was >3.8 ng/ml. This threshold of 3.8 ng/ml was determined from the mean + 3SD of LH concentrations measured on the morning of proestrus at 0 h.

Body mass was recorded at the start of ALPS and just before euthanasia. Males were transported to the laboratory at the end of the ALPS paradigm (2:30 P.M. EST). The mass of the adrenal glands, testes, and seminal vesicles was recorded and normalized to PM body mass. Diestrous females were transported to the laboratory after the LH sample at lights out, and proestrous females used for LH surge sampling were transported after the last sample 2 h after lights out. Adrenal and uterine masses were recorded in females.

### Corticosterone administration

To mimic serum corticosterone concentrations induced by ALPS, STD-reared males from our main colony were fed corticosterone in Nutella, a design based on ongoing studies in females in which 2 mg/kg oral corticosterone at 0, 1, and 3 h (times corresponding to the ALPS transitions) mimics the serum corticosterone pattern observed during the ALPS paradigm (Gibson, 2023). To habituate animals to this feeding paradigm, we transferred the cage mates to a holding cage, and one mouse was left in the home cage with Nutella on a Petri dish for up to 5 min. Mice were habituated daily for at least a week prior to the experiment. On the day of the experiment, corticosterone (2 mg/kg) or 36% DMSO vehicle in Nutella (60 mg Nutella mixture for a 30 g mouse; range, 56.2–75.2 mg) was administered at 0 h (9:30 A.M. EST, 6.5 h after lights on), 1 h, and 3 h. Tail blood samples were collected as described above at 0, 1, 2, 3, 4, and 5 h after initial Nutella administration. Only one mouse per cage was sampled on a given day, as preliminary experiments determined that transfer to the holding cage while a cage mate consumed Nutella increased serum corticosterone concentration. Body and tissue masses were recorded for these mice as described for the ALPS paradigm.

### Corticosterone enzyme immunoassay

Serum corticosterone concentrations were determined in duplicate samples diluted 1:100 by enzyme immunoassay (Arbor Assays, DetectX Corticosterone Kit, K014). Standard curves from 78.1 to 5,000 pg/ml or 39.0 to 10,000 pg/ml were run on each plate. Intra-assay coefficients of variation (%CVs) for standardss ranged from 3 to 6%; functional sensitivity, defined as the lowest standard with a CV <20%, was 39.0 pg/ml. The reportable range was 3.9–1,000 ng/ml.

### Ultrasensitive LH assay

At the time of tail blood collection, 6 µl of whole blood was mixed with 54 µl of assay buffer (0.2% BSA—0.05% Tween 20—PBS), pH 7.5, and immediately placed on ice for up to 3 h and then stored at −20°C. Samples were assayed by Center for Research in Reproduction at the University of Virginia with the Ultrasensitive Mouse and Rat LH ELISA method (Steyn et al., 2013). The capture monoclonal antibody (anti-bovine LH beta subunit, 518B7) was provided by Janet Roser, University of California. The detection polyclonal antibody (rabbit LH antiserum, AFP240580Rb) was provided by the National Hormone and Peptide Program (NHPP). HRP-conjugated polyclonal antibody (goat anti-rabbit) was purchased from DakoCytomation (D048701-2). Mouse LH reference prep (AFP5306A; NHPP) was used as the assay standard. The limit of quantitation (functional sensitivity) is defined as the lowest concentration that demonstrates accuracy within 20% of expected values and intra-assay %CV <20% and was determined by serial dilutions of a defined sample pool. Intra-assay %CV is 2.2%. Inter-assay %CVs were 7.3% (low QC, 0.13 ng/ml), 5.0% (medium QC, 0.8 ng/ml), and 6.5% (high QC, 2.3 ng/ml). Functional sensitivity was 0.016 ng/ml, and the reportable range is 0.016–4.0 ng/ml. Samples were diluted 1:10, making the reportable range 0.16–40 ng/ml.

### Electrophysiology

A subset of the adult proestrous females was used to characterize the effect of LBN and/or ALPS on GABAergic transmission to GnRH neurons. The mouse was transported to the laboratory between 3:00 and 3:30 P.M. EST (1.5–2 h before lights out), and the body mass was recorded. All solutions were bubbled with 95% O_2_/5% CO_2_ for at least 15 min before tissue exposure and throughout the procedures; chemicals were purchased from Sigma-Aldrich unless noted. The brain was rapidly removed and placed in ice-cold sucrose saline solution containing the following (in mM): 250 sucrose, 3.5 KCl, 26 NaHCO_3_, 10 D-glucose, 1.25 Na_2_HPO_4_, 1.2 MgSO_4_, and 3.8 MgCl_2_. Coronal brain slices (300 µM) containing the preoptic area and GnRH neurons were prepared in the sucrose saline solution with a vibratome (VT1200S, Leica Biosystems). Slices were incubated at room temperature for 30 min in a 50–50% mixture of the sucrose saline and artificial cerebrospinal fluid (ACSF) containing the following (in mM): 135 NaCl, 3.5 KCl, 26 NaHCO_3_, 10 D-glucose, 1.25 Na_2_HPO_4_, 1.2 MgSO_4_, and 2.5 CaCl_2_, pH 7.4. Slices were then incubated for 0.5–5 h in 100% ACSF before being transferred to the recording chamber mounted to an Olympus BX51WI upright fluorescent microscope.

Slices in the recording chamber were perfused (3–5 ml/min) with ACSF via a MINIPULS 3 peristaltic pump (Gilson). GABAergic PSCs were isolated by blocking ionotropic glutamate receptors with CNQX (10 µM) and D-APV (20 µM). Solution temperature was maintained between 29 and 32°C with an inline heating system (Warner Instruments). Individual GFP-positive GnRH neurons were visualized using infrared differential interference contrast and brief illumination with fluorescence microscopy. The recording pipette was filled with a high-chloride internal solution containing (in mM): 140 KCl, 10 HEPES, 5 EGTA, 0.1 CaCl_2_, 4 MgATP, and 0.4 NaGTP. A high-resistance (>1 GΩ) seal was made between the cell membrane and the pipette, and then the whole-cell configuration was achieved. The cell was held at −65 mV in voltage-clamp mode, and the recording quality was monitored by averaging the response to 16 hyperpolarizing voltage steps (5 mV, 20 ms, acquisition 100 kHz, filter 10 kHz). GABAergic PSCs were recorded during 2–3 min series (acquisition 10 kHz, filter 5 kHz).

Custom routines in Igor Pro (WaveMetrics) were used to detect PSCs, which were manually confirmed. The frequency of PSCs (number of events/recording duration) was determined for each cell. For each event, the interevent interval was calculated, defined as the backward interval from the time of that event's peak to the time of peak for the previous event. The cumulative probability distributions of interevent interval for each treatment group were also calculated. The true interevent interval for the first event in a recording is unknown and thus not included in calculations of interevent interval. For events with an interevent interval of at least 200 ms, the amplitude (the absolute value of the difference between the peak and baseline) was determined, and cumulative probabilities of amplitudes were calculated for each group; this analysis included first events preceded by >200 ms of the recording time. Isolated events (>200 ms interval in both the forward and backward direction between adjacent event peaks) were selected and averaged by cell. These averaged traces were used to estimate the decay time from 80 to 20% of the peak for each cell.

### Statistical analysis

Statistical analysis was conducted in R (R Core Team, 2023); statistical packages used included rstatix (Kassambara, 2021), lme4 (Bates et al., 2015), lmerTest (Kuznetsova et al., 2017), lspline (Bojanowski, 2017), afex (Singmann et al., 2023), emmeans (Lenth et al., 2023), and nparLD (Noguchi et al., 2012). Plots were made with the packages ggplot2 (Wickham, 2016) and cowplot (Wilke, 2023). Tables were made with the flextable package (Gohel and Skintzos, 2023).

Linear mixed models (LMMs) were used to analyze these data as these models can account for the dependencies among data attributable to experimental design and permit missing data (Singmann and Kellen, 2019). For example, litter was included as a random effect for outcomes measured in offspring, as mice within a litter are not fully independent from one another, violating assumptions of more traditional tests such as ANOVAs. The type of test for each outcome measure and associated figure are in Table 1. Type 3 tests with effects coding were used, as recommended by Singmann and Kellen (2019). The Kenward–Roger approximation was used for estimation of degrees of freedom for LMMs. Post hoc comparisons were made using pairwise tests of estimated marginal means (emmeans). For multiple pairwise comparisons, *p* values were adjusted using Holm's method; confidence intervals could only be adjusted using the more conservative Bonferroni’s method with this package in R. The pairwise comparisons for all tests are in Table 2.

**Table 1.** The type of statistical test used to analyze each outcome

Figures	Outcome	Test	Formula
1*B*	Dam mass	LMM	Mass ∼ early-life treatment × PND^a^ + (1|dam)
1*C*	Dam corticosterone	*t* test	Corticosterone ∼ early-life treatment
1*D*	Number of nest exits	Nonparametric longitudinal F1 LD F1	Number of nest exits ∼ early-life treatment × PND^a^
1*E*	Percentage of time off nest	Nonparametric longitudinal F1 LD F1	Percentage off nest ∼ early-life treatment × PND^a^
2*A*	PND4 offspring mass litter average	*t* test	Mass ∼ early-life treatment
2*B*	PND11 offspring mass individual pups	LMM	Mass ∼ early-life treatment × sex + (1|dam)
2*C*	Offspring mass males and females run separately	LMM with splines	Mass ∼ early-life treatment × PND^b^ + (1|dam) + (1|mouse)
2*D*	Age at vaginal opening	LMM	Age ∼ early-life treatment + (1|dam)
	Age at first estrus		
	Age at preputial separation		
2*E*	Mass at vaginal opening	LMM	Mass ∼ early-life treatment + (1|dam)
	Mass at first estrus		
	Mass at preputial separation		
Text	AGD	LMM	AGD ∼ early-life treatment × sex + (1|dam)
3*B*	Number of estrous cycles	LMM	Number of estrous cycles ∼ early-life treatment + (1|dam)
3*C*	Cycle length	LMM	log10(cycle length) ∼ early-life treatment + (1|dam)
3*D*	Percentage of days in stage	*χ*^2^ test	Distribution of days spent in diestrus, proestrus, and estrus by early-life treatment
4*A*	Male serum corticosterone	LMM	log10(cort) ∼ early-life treatment × adult treatment × time + (1|mouse) + (1|dam)
4*B*	Female serum corticosterone	LMM	log10(cort) ∼ cycle stage × early-life treatment × adult treatment × time + (1|mouse) + (1|dam)
4-1*A*	Body mass	LMM	Outcome ∼ early-life treatment × adult treatment + (1|dam)
4-1*B*	Percentage of change in body mass		
4-1*C*	Adrenal mass		
4-1*D*	Adrenal mass normalized to body mass		
4-1*E*	Seminal vesicle mass		
4-1*F*	Seminal vesicle mass normalized to body mass		
4-1*G*	Testicular mass		
4-1*H*	Testicular mass normalized to body mass		
4-2*A*	Body mass	LMM	Outcome ∼ early-life treatment × adult treatment × cycle stage + (1|dam)
4-2*B*	% Change body mass		
4-2*C*	Adrenal mass		
4-2*D*	Adrenal mass normalized to body mass		
4-2*E*	Uterine mass		
4-2*F*	Uterine mass normalized to body mass		
4-3*B*	Serum corticosterone	LMM	log10(cort) ∼ dosage × time + (1|mouse) + (1|dam)
4-3*A*	Body mass	LMM	Outcome ∼ dosage + (1|dam)
4-3*C*	Percentage of change in body mass		
4-3*D*	Adrenal mass		
4-3*E*	Adrenal mass normalized to body mass		
4-3*F*	Seminal vesicle mass		
4-3*G*	Seminal vesicle mass normalized to body mass		
4-3*H*	Testicular mass		
4-3*I*	Testicular mass normalized to body mass		
5*A*	Average LH, diestrus mice	LMM	Average LH ∼ early-life treatment × adult treatment + (1|dam)
5*C*	Proportion with LH surge, proestrus	GLMM—logistic regression	Surged ∼ early-life treatment + adult treatment + (1|dam)
6*A*	Capacitance	LMM	Outcome ∼ early-life treatment × adult treatment + (1|mouse) + (1|dam)
6*B*	Input resistance		
6*C*	Series resistance		
6*D*	Holding current		
7*C*	PSC frequency	GLMM—NB	Number of events in 4 min ∼ early-life treatment × adult treatment + (1|mouse) (1|dam)
7*D*	Mean interevent interval	LMM	log10(interval) ∼ early-life treatment × adult treatment + (1|mouse) + (1|dam)
7*E*	Mean relative amplitude	LMM	Outcome ∼ early-life treatment × adult treatment + (1|mouse) + (1|dam)
7*F*	Decay time		
7*G*	Distribution of interevent interval	Anderson–Darling test and bootstrapping. See Materials and Methods, Statistical analysis	
7*H*	Distribution of amplitude		

LMMs were used where appropriate for the data structure and experimental design. Generalized linear mixed models (GLMM) were fit with either logistic regression or NB families.

a Indicates that PND was treated as a factor variable. For the analysis of dam behavior with a nonparametric longitudinal test, the nparLD package in R was used, with the F1 LD F1 Model. The subject variable was each dam, early-life treatment (STD or LBN cage) was the between-subject factor (“whole-plot” factor), and PND was the within-subject factor (“sub-plot” repeated factor).

b Indicates that linear splines at PND21 and 35 for the models of offspring mass allow the model to change the slope of the line for the segments between PND11 and 21, from 21 to 35, and from 35 to 72.

**Table 2.** Statistical table for pairwise comparisons for post hoc tests

Figures	Outcome	Group level	Contrast	Estimate	95% CI	SEM	Degrees of freedom	*t*	*p*	Row
1*B*	Dam mass		STD - LBN	−1.23	[−2.36, −0.10]	0.562	47.0	−2.20	0.033	1
			PND4 - PND11	−2.78	[−3.33, −2.22]	0.227	94.0	−12.21	<0.001	2
			PND4 - PND21	−2.15	[−2.70, −1.59]	0.227	94.0	−9.45	<0.001	3
			PND11 - PND21	0.63	[0.07, 1.18]	0.227	94.0	2.76	0.007	4
2*B*	PND11 mass		STD - LBN	0.55	[0.17, 0.94]	0.192	47.0	2.87	0.006	5
2*C*	Male mass	PND 11	STD - LBN	0.67	[−0.28, 1.63]	0.355	43.2	1.90	0.129	6
		PND 21	STD - LBN	0.62	[−0.33, 1.57]	0.353	41.9	1.76	0.129	7
		PND 35	STD - LBN	0.77	[−0.21, 1.74]	0.364	47.5	2.11	0.121	8
		PND 56	STD - LBN	0.93	[−0.02, 1.88]	0.352	41.7	2.66	0.045	9
		PND 72	STD - LBN	1.06	[0.10, 2.03]	0.358	44.5	2.97	0.024	10
	AGD		Female - male	−10.35	[−10.48, −10.23]	0.065	239.8	−160.51	<0.001	11
4*A*	Male serum corticosterone	Pre	CON / ALPS	0.98	[0.71, 1.35]	0.125	121.7	−0.15	0.883	12
		Post	CON / ALPS	0.32	[0.23, 0.44]	0.041	122.2	−8.91	<0.001	13
		CON	Pre / post	0.35	[0.25, 0.47]	0.042	74.1	−8.68	<0.001	14
		ALPS	Pre / post	0.11	[0.08, 0.15]	0.014	73.2	−18.04	<0.001	15
4*B*	Female serum corticosterone	Di pre	CON / ALPS	1.21	[0.69, 2.13]	0.237	200.2	0.97	0.826	16
		Di post	CON / ALPS	0.31	[0.18, 0.55]	0.061	200.2	−5.98	<0.001	17
		Pro pre	CON / ALPS	0.79	[0.53, 1.18]	0.110	215.8	−1.67	0.387	18
		Pro post	CON / ALPS	0.40	[0.27, 0.59]	0.055	215.8	−6.70	<0.001	19
		Di CON	Pre / post	0.22	[0.13, 0.38]	0.041	109.0	−8.08	<0.001	20
		Di ALPS	Pre / post	0.06	[0.03, 0.10]	0.011	109.0	−14.47	<0.001	21
		Pro CON	Pre / post	0.26	[0.17, 0.41]	0.041	109.0	−8.62	<0.001	22
		Pro ALPS	Pre / post	0.13	[0.09, 0.18]	0.015	109.0	−18.00	<0.001	23
		Pre CON	Diestrus / proestrus	0.68	[0.41, 1.14]	0.120	213.5	−2.16	0.160	24
		Pre ALPS	Diestrus / proestrus	0.45	[0.28, 0.72]	0.074	215.5	−4.85	<0.001	25
		Post CON	Diestrus / proestrus	0.83	[0.50, 1.37]	0.145	213.5	−1.09	0.826	26
		post ALPS	Diestrus / proestrus	1.05	[0.65, 1.70]	0.174	215.5	0.31	0.826	27
4-1*A*	Body mass		STD - LBN	2.37	[0.16, 4.59]	1.067	21.9	2.22	0.037	28
			CON - ALPS	−0.97	[−1.68, −0.26]	0.353	50.7	−2.75	0.008	29
4-1*B*	Percentage of change body mass		CON - ALPS	3.66	[3.20, 4.11]	0.229	56.7	16.00	<0.001	30
4-1*D*	Adrenal mass normalized to body mass		STD - LBN	−0.01	[−0.03, 0.00]	0.007	21.2	−2.01	0.057	31
4-1*E*	Seminal vesicle mass		CON - ALPS	−12.78	[−27.44, 1.88]	7.295	49.4	−1.75	0.086	32
4-1*F*	Seminal vesicle mass normalized to body mass		STD - LBN	−0.81	[−1.39, −0.23]	0.278	20.6	−2.91	0.008	33
			CON - ALPS	−0.51	[−1.02, −0.01]	0.253	52.4	−2.03	0.048	34
4-1*G*	Testes mass		STD - LBN	15.52	[8.10, 22.94]	3.565	20.8	4.35	<0.001	35
			CON - ALPS	9.36	[3.25, 15.46]	3.046	55.0	3.07	0.003	36
	Testes mass normalized to body mass		CON - ALPS	0.27	[0.04, 0.49]	0.113	50.6	2.37	0.021	37
4-2*B*	Percentage of change body mass		Diestrus - proestrus	−0.77	[−1.49, −0.04]	0.365	103.9	−2.10	0.038	38
			CON - ALPS	2.83	[2.11, 3.55]	0.363	93.6	7.81	<0.001	39
4-2*C*	Adrenal mass		STD - LBN	−0.29	[−0.57, 0.00]	0.140	30.6	−2.05	0.049	40
4-2*D*	Adrenal mass normalized to body mass		CON - ALPS	−0.01	[−0.02, 0.00]	0.005	60.8	−1.76	0.084	41
4-2*E*	Uterine mass		Diestrus - proestrus	−71.38	[−77.31, −65.46]	2.981	85.5	−23.95	<0.001	42
4-2*F*	Uterine mass normalized to body mass		Diestrus - proestrus	−2.98	[−3.26, −2.71]	0.138	84.4	−21.62	<0.001	43
4-3*B*	Male cort admin serum corticosterone	0 h	0 / 2 mg/kg	1.12	[0.78, 1.61]	0.151	172.7	0.86	0.393	44
		1 h	0 / 2 mg/kg	0.14	[0.10, 0.20]	0.019	172.7	−14.72	<0.001	45
		2 h	0 / 2 mg/kg	0.06	[0.04, 0.09]	0.008	172.7	−20.57	<0.001	46
		3 h	0 / 2 mg/kg	0.15	[0.10, 0.21]	0.020	172.7	−14.34	<0.001	47
		4 h	0 / 2 mg/kg	0.10	[0.07, 0.14]	0.013	172.7	−17.18	<0.001	48
		5 h	0 / 2 mg/kg	0.18	[0.13, 0.26]	0.024	172.7	−12.70	<0.001	49
4-3*C*	Percentage of change body mass		0 − 2 mg/kg	0.68	[−0.02, 1.39]	0.344	27.5	1.99	0.057	50
4-3*H*	Testes mass		0 − 2 mg/kg	7.28	[1.90, 12.67]	2.613	24.5	2.79	0.010	51
4-3*I*	Testes mass normalized to body mass		0 − 2 mg/kg	0.13	[−0.02, 0.28]	0.073	24.5	1.80	0.085	52

Holm's method for *p* value adjustment was used for multiple comparisons. Confidence intervals were adjusted using the more conservative Bonferroni's method. The statistical test used for each outcome is in Table 1.

Residual and Q–Q (quantile–quantile) plots were used to check the assumptions of models; alternative models were selected when available if assumptions were not met, as reported in Table 1. Nonparametric longitudinal analysis has been used to assess mouse behavioral data across time (Noguchi et al., 2012; Pernold et al., 2019) and was used to analyze the number of exits that the dams made from the nest over time. Because the ultrasensitive LH assay can report a maximum concentration of 40 ng/ml and many of our samples in proestrous mice exceeded this concentration, data for this parameter were not normally distributed, and an accurate estimate of the effect of stress on absolute LH concentrations was precluded. We instead focused on the binary outcome of whether or not the mouse exhibited an LH surge (at least one measurement ≥3.8 ng/ml) and fit these data with a generalized linear mixed-effect model for the binomial logistic regression family. The sample sizes and the low variance in the adult CON groups, because of the high likelihood of observing a surge in those groups, precluded the model from adequately estimating the interaction term of early-life treatment (LBN) and adult treatment (ALPS). Thus, the formula was simplified to consider only the independent main effects of each stressor.

The number of PSCs was fit with a generalized linear mixed-effect model for the negative binomial (NB) family to handle count data with meaningful zeros (no observed PSCs during recording period). To assess if early-life or adult treatment affected PSC properties (interval and amplitude), we compared the distributions from all four treatment groups with the Anderson–Darling test using the kSamples package in R (Scholz and Zhu, 2023). If the test comparing all four distributions was significant, post hoc Anderson–Darling tests comparing the (1) STD-CON and STD-ALPS, (2) LBN-CON and LBN-ALPS, (3) STD-CON and LBN-CON, and (4) STD-ALPS and LBN-ALPS groups were conducted, and *p* values were adjusted for multiple comparisons. To help with interpretation of the comparison of these distributions, a bootstrapping approach was used to estimate the 95% confidence interval of the mean of each treatment group and the difference in means for the four comparisons described just above (1–4). This approach, inspired by Ho et al. (2019), was necessitated by the non-normality of the distributions and adapted using the custom code to account for the experimental design. Briefly, the dataset was resampled 5,000 times; for each resampling iteration, the mean of each treatment group was calculated, as was the difference in the means for the comparisons described above (1–4). The average of these group means and differences in means were calculated for the 5,000 iterations. The boundaries for the 95% confidence intervals, or percentile intervals, were found by ranking the group's mean estimates for all 5,000 iterations and selecting the 2.5th percentile and the 97.5th percentile as the lower and upper bounds of the interval, respectively; the same process was applied to obtain a confidence interval for the differences in means.

### Code accessibility

The PSC detection and analysis code used is freely available online at https://gitlab.com/um-mip/coding-project/. The FFmpeg and R analysis code is freely available online at https://github.com/gibson-amandag/LBN. These scripts are also provided as Extended Data 1. Analyses were conducted on a Lenovo Yoga 9, 11th Gen Intel Core i7, running Windows 11 Home.

10.1523/ENEURO.0125-24.2024.d1 Extended Data Zip file of custom code for PSC detection and analysis, ffmpeg recording of dam behavior, and R analysis. Download Extended Data, ZIP file.

## Results

### LBN dams exited the nest more frequently

**Figure 1.**
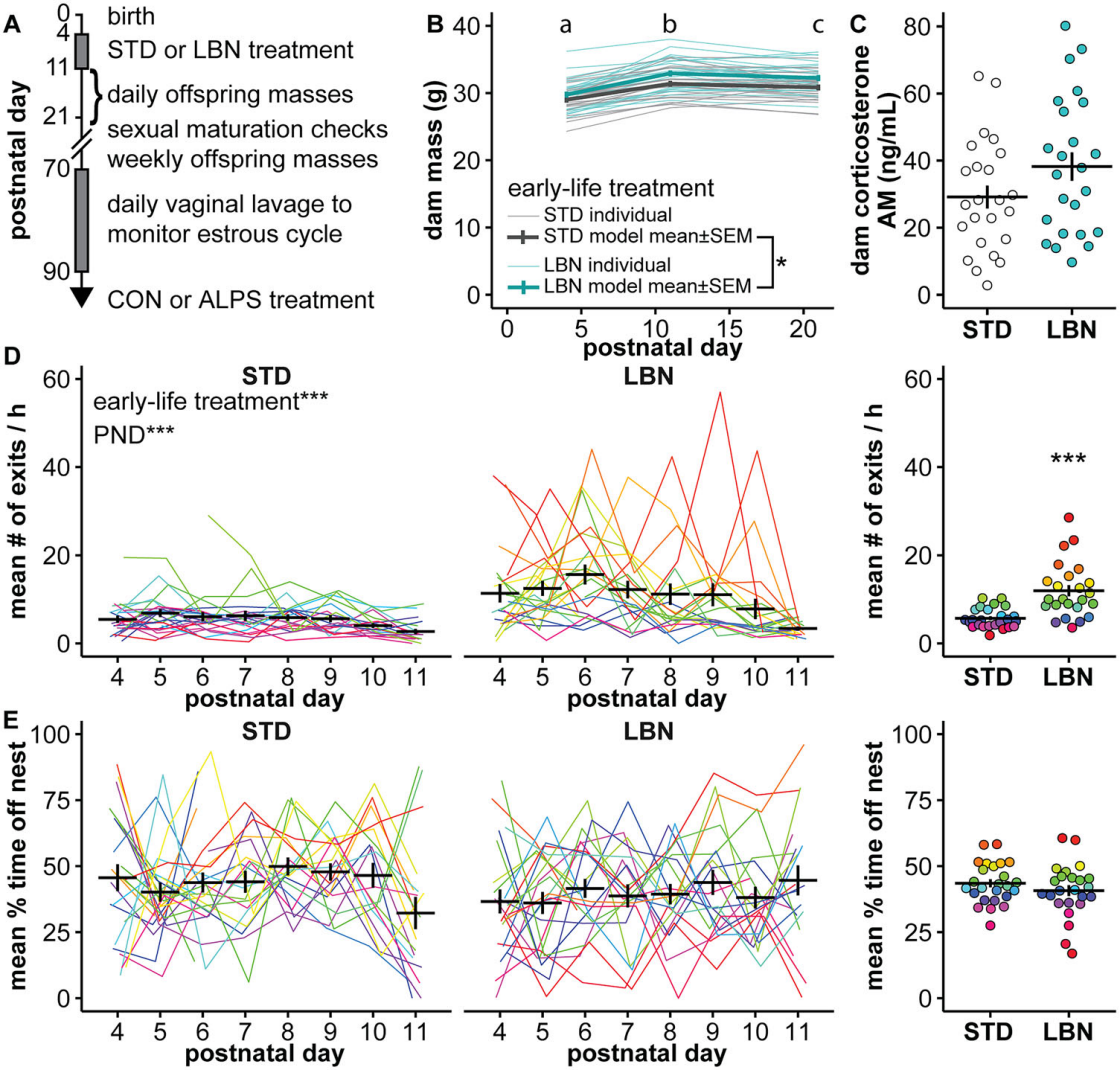
The LBN paradigm altered dam behavior. ***A***, Experimental timeline. ***B***, Dam mass before and after the paradigm and at weaning on PND21. Thin lines are individual dams, LMM model mean ± SEM shown in thick lines. Letters (a–c) indicate that dam mass, combined across treatment groups, differed on each PND (*p *< 0.01). ***C***, Individual values and mean ± SEM serum corticosterone from the dams at the end of the paradigm on the morning of PND11. ***D***, Left, The number of exits averaged by PND for individual dams are shown by colored lines; warmer colors indicate more exits; mean ± SEM number of nest exits versus PND is in black. Right, Individual dam averages and mean ± SEM number of nest exits; the color is consistent with the left graph. ***E***, Left, The percentages of the time spent off nest averaged by PND for individual dam are shown by colored lines; warmer colors indicated more time off the nest; mean ± SEM percentage of time off the nest versus PND is in black. Right, Individual dam averages and mean ± SEM percentage of time off nest; the color is consistent with the left graph. Some error bars are obscured by the mean line. *p < 0.05, ***p < 0.001. Numbers in Extended Data Table 1-1. Full statistical model results are in Extended Data Table 1-2. STD, standard-reared; LBN, limited bedding and nesting; CON, adult control treatment; ALPS, adult acute, layered, psychosocial stress; PND, postnatal day.

LBN was applied from PND4 to 11 (Fig. 1*A*; numbers in Extended Data Table 1-1). LBN dams had a higher body mass than STD dams at PND4, 11, and 21 (Fig. 1*B*; Extended Data Table 1-2; STD, *n* = 25; LBN, *n* = 24; Table 2, Row 1, *p *= 0.033). Regardless of treatment, dams gained body mass during the paradigm (Table 2, Row 2, *p *< 0.001) and decreased body mass between PND11 and 21 (Table 2, Row 4, *p *= 0.007). There were no differences in morning serum corticosterone concentration between STD and LBN dams on PND11, indicating LBN did not chronically elevate this hormone in dams [Fig. 1*C*; STD, *n* = 24; LBN, *n* = 24; *t*_46 _= −1.67; *p *= 0.102; difference (STD-LBN) = −9.08; 95% CI = [−20.04, 1.88]; Cohen's *d* = −0.48]. This may indicate the LBN phenotype is milder in CBA dams than in other strains. Dam behavior was captured on video during the paradigm. The number of exits that each dam made from the nest was scored for 1 h periods beginning at ZT1, 15, and 19 and then averaged for each PND (STD, *n* = 19–25; LBN, *n* = 19–24). LBN dams had more exits than STD dams throughout the paradigm (Fig. 1*D*; warmer colors indicate dams with more exits over time; Extended Data Table 1-2; *p *< 0.001). Interestingly, there was no difference in the percentage of time that STD and LBN dams spent on the nest (Fig. 1*E*; Extended Data Table 1-2; *p *= 0.156). Together, these observations suggest that LBN dams have more fragmented interactions with the pups.

10.1523/ENEURO.0125-24.2024.t1-1 Table 1-1 Number of dams in each group for studies in Figure 1. Dam behavior includes values for number of nest exits per hour and percentage of time spent off the nest. Download Table 1-1, DOCX file.

10.1523/ENEURO.0125-24.2024.t1-2 Table 1-2 Statistics for tests of dam mass and dam behavior over time. Linear mixed model and pairwise comparisons of the dam mass on PND4, 11, and 21 was fit with the equation mass ∼ early-life treatment * PND@ + (1 | dam). Postnatal day (PND) was treated as a factor variable. Dam behavior parameters were analyzed with a nonparametric longitudinal model using the nparLD package in R, with the F1 LD F1 Model. The subject variable was each dam, early-life treatment (STD or LBN cage) was the between-subject factor (‘whole-plot’ factor), and PND was the within-subject factor (‘sub-plot’ repeated factor). Download Table 1-2, DOCX file.

### LBN affected pup mass

**Figure 2.**
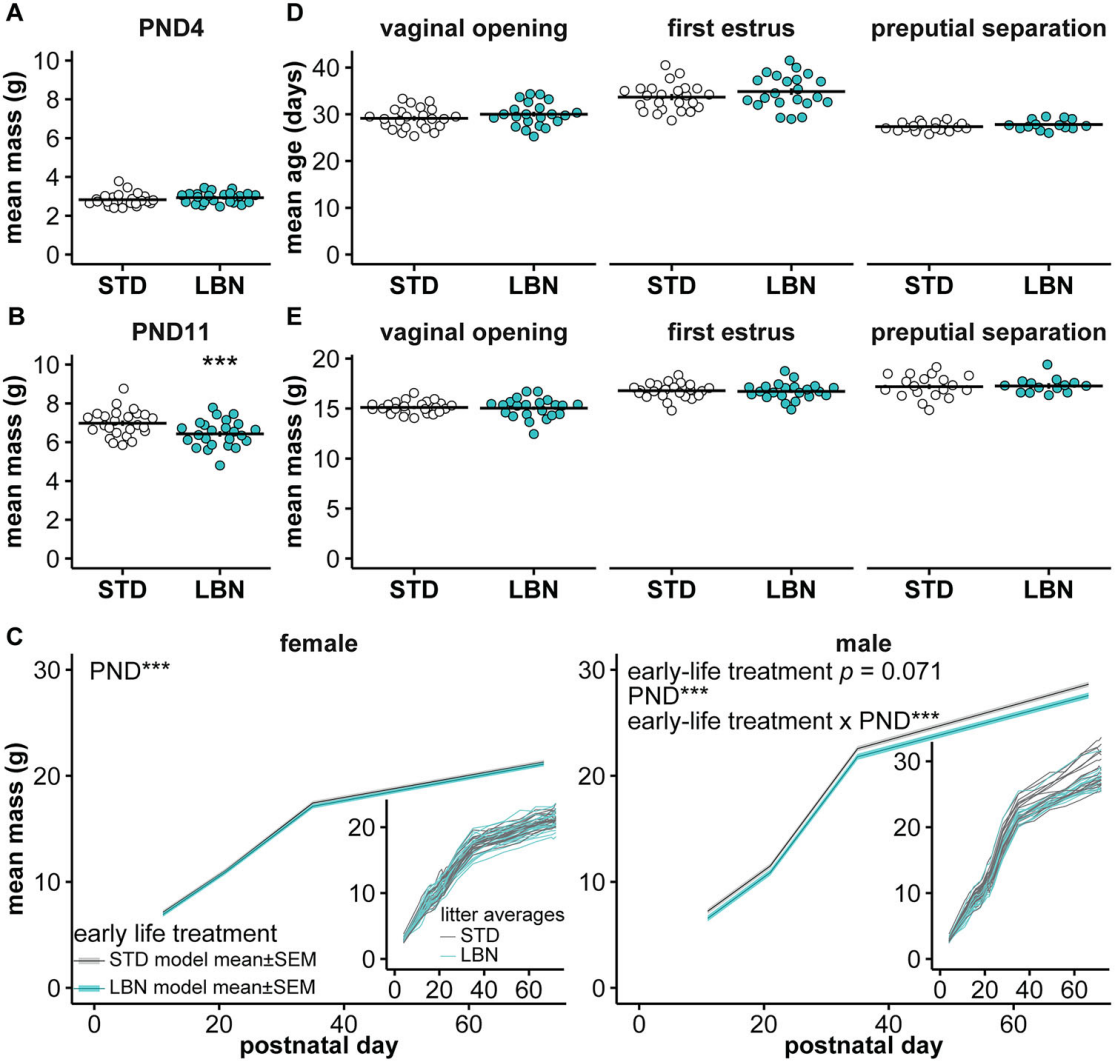
LBN decreased PND11 body mass and lowered body mass in adult males but did not affect reproductive maturation. ***A***, Mean litter values and mean ± SEM for PND4 mass. ***B***, Mean litter values (both sexes) and model mean ± SEM for PND11 mass. ***C***, Statistical model mean ± SEM for the body mass of the female (left) and male (right) offspring. The average mass of each litter is plotted in the insets. ***D***, ***E***, Mean litter values and model mean ± SEM for age (***D***) at the vaginal opening (left), first estrus (center), and preputial separation (right); for mass (***E***) at the vaginal opening (left), first estrus (center), and preputial separation (right). Some error bars are obscured by the mean line. ***p < 0.001. Full statistical model results are in Extended Data Table 2-1 (mass at PND11), Extended Data Table 2-2 (mass from PND11–72), and Extended Data Table 2-3 (maturation). STD, standard-reared; LBN, limited bedding and nesting.

Prior to treatment on PND4, there were no differences in offspring body mass between the litters that would receive STD (mean ± SEM, 2.8 g ± 0.06, 25 litters) and LBN (2.9 g ± 0.06, 24 litters) treatment [Fig. 2*A*; *t*_(47) _= −1.18; *p *= 0.245; difference (STD-LBN) = −0.10; 95% CI = [−0.27, 0.07]; Cohen's *d* = −0.34]. After treatment on PND11, LBN offspring were smaller than STD offspring (Fig. 2*B*; Extended Data Table 2-1; Table 2, Row 5, *p *= 0.006; STD, 74 females and 80 males; LBN, 74 females and 58 males). Offspring sex did not affect mass at PND11 (*p *= 0.616) or interact with treatment (*p *= 0.124); the overall litter averages are thus displayed in Figure 2*B*. This demonstrates that mass gain during the treatment window was slower in LBN offspring, consistent with prior studies of this early-life stressor.

10.1523/ENEURO.0125-24.2024.t2-1 Table 2-1 Linear mixed models of offspring mass on PND11 and anogenital distance. Equations were outcome ∼ early-life treatment * sex + (1 | dam). Early-life treatment is STD vs LBN rearing. Sex is males vs females. Download Table 2-1, DOCX file.

10.1523/ENEURO.0125-24.2024.t2-2 Table 2-2 Linear mixed model of the offspring mass from PND11-72 fit with the equation mass ∼ early-life treatment * PND@ + (1 | dam) + (1 | mouse). Early-life treatment is STD vs LBN rearing. PND@: Linear splines at PND21 and 35 allow the model to change the slope of the line for the segments between PND11-21, from 21-35, and from 35-72. Male and female offspring were fit with separate models. Download Table 2-2, DOCX file.

10.1523/ENEURO.0125-24.2024.t2-3 Table 2-3 Linear mixed models for maturation with the equation maturation feature ∼ early-life treatment + (1 | dam). Early-life treatment is STD vs LBN rearing. Models were fit for age and for mass at vaginal opening, first estrus, and preputial separation. Download Table 2-3, DOCX file.

We continued recording offspring mass into adulthood to test if there was an effect of rearing conditions on subsequent growth, creating separate linear models of growth for female and male offspring. LBN treatment did not alter body mass growth in females (Fig. 2*C*; Extended Data Table 2-2; STD, postweaning, 24 litters and 74 mice; LBN, postweaning, 22 litters and 73 mice). In contrast in males, LBN treatment altered the overall pattern of growth (Fig. 2*C*; Extended Data Table 2-2; *p *< 0.001; STD, postweaning, 19 litters and 63 mice; LBN, postweaning, 14 litters and 42 mice). To understand how LBN changed the trajectory of growth, we conducted post hoc comparisons of body mass at discrete days throughout development (PND11, 21, 35, 56, and 72). The masses were not different through PND35 (PND11, Table 2, Row 6, *p *= 0.129; PND 21, Table 2, Row 7, *p *= 0.129; PND 35, Table 2, Row 8, *p *= 0.121). As males transitioned from adolescence to adulthood, the LBN mice appeared to gain mass more slowly, and by PND56, the LBN mice were ∼1 g smaller than STD mice (Table 2, Row 9, *p *= 0.045) with the difference persisting into the end of the observation period at PND72 (Table 2, Row 10, *p *= 0.024).

### LBN did not alter reproductive maturation or estrous cycles

**Figure 3.**
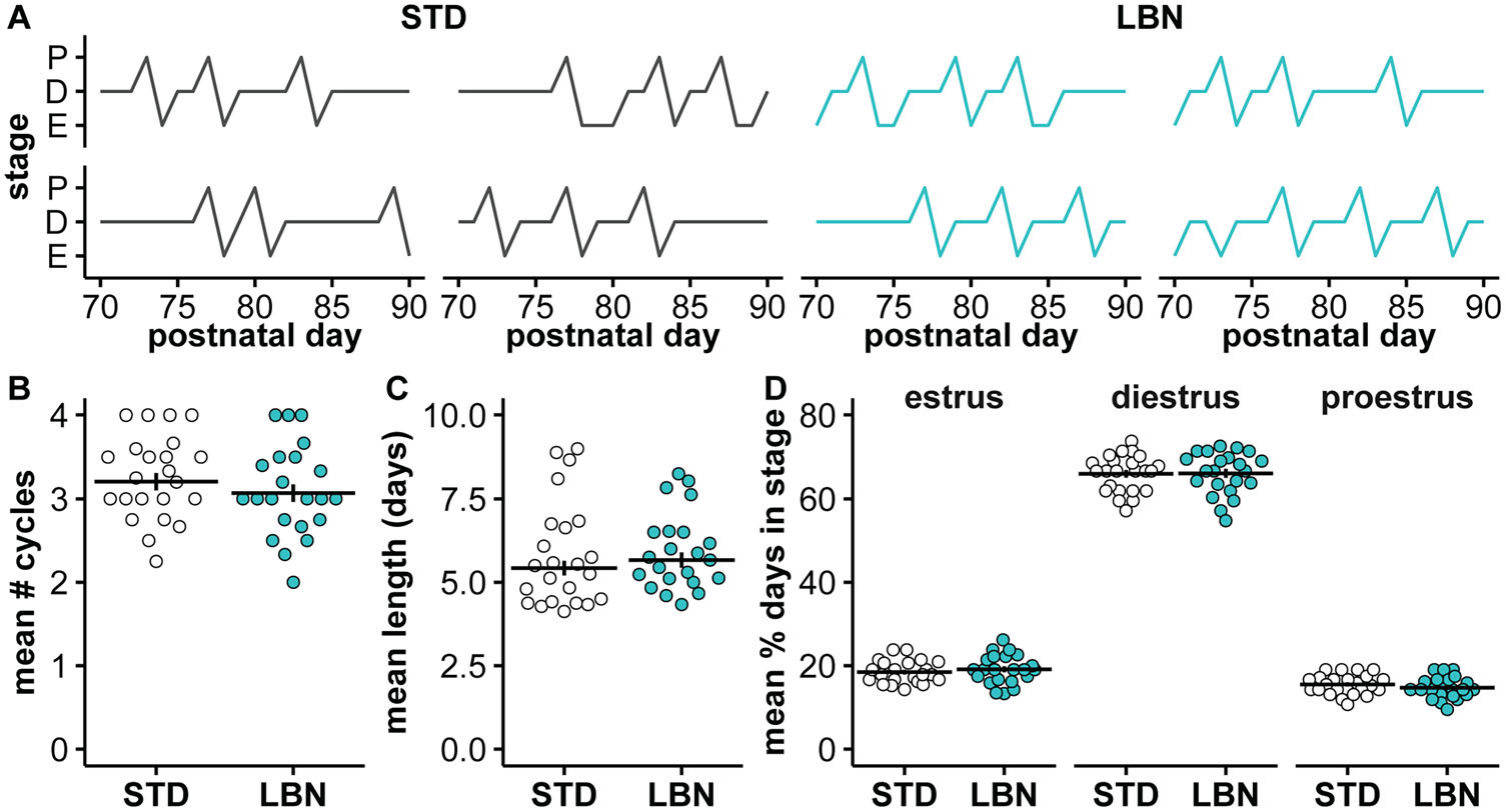
LBN did not alter estrous cyclicity from PND70 to 90. ***A***, Representative individual estrous cycle traces for STD (left) and LBN (right) offspring, proestrus (P), estrus (E), or diestrus (D). ***B***, Number of cycles averaged for female littermates; model mean ± SEM. ***C***, Mean cycle length averaged for female littermates; model mean ± SEM. ***D***, The mean percentage of days spent in each cycle stage for female littermates; data mean ± SEM; no model value is available as *χ*^2^ test was used for analysis of percentage values. Full statistical model results are in Extended Data Table 3-1. Some error bars obscured by the mean line. STD, standard-reared; LBN, limited bedding and nesting; PND, postnatal day.

To assess if LBN altered external markers of reproductive maturation, the age and mass at vaginal opening and first estrus were monitored in females (STD, 24 litters and 74 mice; LBN, 22 litters and 73 mice, unless otherwise noted below); age and mass at preputial separation were monitored in males (STD, 19 litters and 63 mice; LBN, 14 litters and 41 mice). LBN did not affect age at vaginal opening (Fig. 2*D*; Extended Data Table 2-3; *p *= 0.217) or first estrus (*p *= 0.221), and there were no differences in body mass at these milestones (Fig. 2*E*; Extended Data Table 2-3; vaginal opening, *p *= 0.754; first estrus, *p *= 0.758; STD, 23 litters and 70 mice; LBN, 22 litters and 73 mice). Similarly, the age (Fig. 2*D*; Extended Data Table 2-3; *p *= 0.177) and mass (Fig. 2*E*; Extended Data Table 2-3; *p *= 0.846) at preputial separation were not affected by LBN. Adult AGD was not affected by LBN in either sex [model mean ± SEM (mm), female STD, 6.4 ± 0.08; female LBN, 6.3 ± 0.09; male STD, 16.8 ± 0.09; male LBN, 16.6 ± 0.1; Extended Data Table 2-1; *p *= 0.257], but the typical increased AGD in males versus females was observed (Extended Data Table 2-1; Table 2, Row 11, *p *< 0.001). To test if LBN altered estrous cycles, daily vaginal lavages were obtained from PND70 to 90 (STD, 23 litters and 73 mice; LBN, 22 litters and 73 mice). Figure 3*A* shows representative estrous cycles from both groups. LBN had no effect on the number of estrous cycles (Fig. 3*B*; Extended Data Table 3-1; *p *= 0.359) or features of the cycle including the length (Fig. 3*C*; Extended Data Table 3-1; *p *= 0.457) or the percentage of days spent in each stage (Fig. 3*D*; Extended Data Table 3-1; *p *= 0.865). Together, these results indicate that LBN did not disrupt reproductive maturation or estrous cyclicity.

10.1523/ENEURO.0125-24.2024.t3-1 Table 3-1 Statistics for estrous cycles from postnatal days 70-90. The number of cycles was fit with the linear mixed model equation # of cycles ∼ early-life treatment + (1 | dam). The log of the mean cycle length in days was fit with equation log_10_(cycle length) ∼ early-life treatment + (1 | dam). Early-life treatment is STD vs LBN rearing. The number of days spent in each cycle stage was assessed with a Chi-squared test (n = 3066). Download Table 3-1, DOCX file.

### Early-life stress did not alter the corticosterone response to adult stress

**Figure 4.**
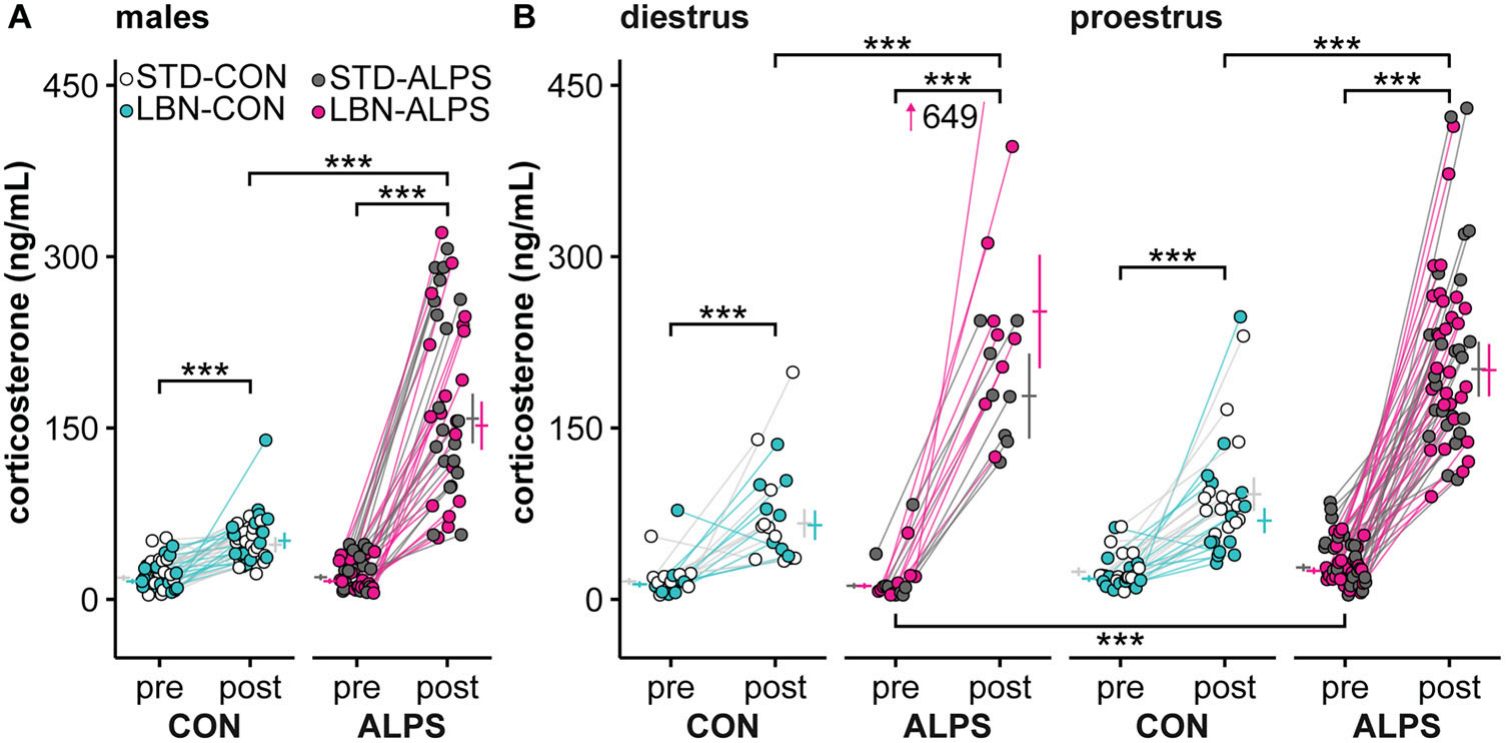
LBN does not change the corticosterone response to ALPS. Individual values and model mean ± SEM (adjacent horizontal lines and vertical error bars) for pre- and posttreatment serum corticosterone concentrations in males (***A***) and females (***B***; diestrus left, proestrus right). Early-life treatment groups are plotted together as there were no effects of LBN treatment on serum corticosterone concentrations at any point. Numbers are in Extended Data Table 4-1. Results from the full statistical models are in Extended Data Table 4-2. Additional data regarding tissue masses and the effect of corticosterone on masses are in Extended Data Figure 4-1 (male tissue masses), Extended Data Figure 4-2 (female tissue masses), Extended Data Figure 4-3 (male corticosterone administration), and Extended Data Tables 4-3–4-8. STD, standard-reared; LBN, limited bedding and nesting; CON, adult control; ALPS, acute, layered, psychosocial stress in adulthood. ***p < 0.001.

To determine if early-life stress alters the serum corticosterone response to adult stress, STD and LBN mice were exposed to an ALPS paradigm (Wagenmaker and Moenter, 2017) or remained in nonstressed, home cage CON conditions (numbers in Extended Data Table 4-1). There were no effects of early-life stress at any point in either sex or either cycle stage in females (Extended Data Table 4-2); thus, results are combined in Figure 4. Baseline corticosterone levels were the same in CON and ALPS males (Table 2, Row 12, *p *= 0.883) and diestrous (Table 2, Row 16, *p *= 0.826) and proestrous (Table 2, Row 18, *p *= 0.387) females. Baseline corticosterone levels were elevated in proestrous relative to diestrous mice that received ALPS (Table 2, Row 25, *p *< 0.001). In all three groups, CON mice exhibited the typical diurnal increase in corticosterone (male, Table 2, Row 14, *p *< 0.001; diestrus, Table 2, Row 20, *p *< 0.001; proestrus, Table 2, Row 22, *p *< 0.001). Similarly, all three groups had a similar response to ALPS treatment, postparadigm corticosterone concentrations being 2–3-fold greater in ALPS than in CON mice (male, Table 2, Row 13, *p *< 0.001; diestrus, Table 2, Row 17, *p *< 0.001; proestrus, Table 2, Row 19, *p *< 0.001). These results indicate that early-life stress in the form of LBN treatment from PND4 to 11 did not alter this neuroendocrine response to a series of psychosocial stressors in adulthood in either males or females.

10.1523/ENEURO.0125-24.2024.f4-1 Figure 4-1 The ALPS paradigm caused small changes in tissue mass in males. Individual values and model mean ± SEM for **A**. morning body mass; **B**. percent change in body mass after adult treatment; **C**. adrenal mass; **D**. normalized adrenal mass; **E**. seminal vesicle mass; **F**. normalized seminal vesicle mass; **G**. testicular mass; and **H**. normalized testicular mass. Some error bars obscured by mean lines. * *p *< 0.05, ** *p *< 0.01, *** *p *< 0.001. Numbers are in Table 4-3. Results from the full statistical models are in Table 4-4. Abbreviations: STD, standard-reared; LBN, limited bedding and nesting; CON, adult control; ALPS, acute, layered, psychosocial stress in adulthood. Download Figure 4-1, TIF file.

10.1523/ENEURO.0125-24.2024.f4-2 Figure 4-2 LBN and ALPS cause limited changes in tissue masses in females. Individual values and model mean ± SEM for **A**. morning body mass; **B**. percent change in body mass after adult treatment. **C**. adrenal mass; **D**. normalized adrenal mass; **E**. uterine mass; **F**. normalized uterine mass in diestrous (left) and proestrous (right) females. Some error bars obscured by mean lines. * *p *< 0.05, ** *p *< 0.01, *** *p *< 0.001. Numbers are in Table 4-7. Results for the full statistical models are in Table 4-8. Abbreviations: STD, standard-reared; LBN, limited bedding and nesting; CON, adult control; ALPS, acute, layered, psychosocial stress in adulthood. Download Figure 4-2, TIF file.

10.1523/ENEURO.0125-24.2024.f4-3 Figure 4-3 Acute elevation of serum corticosterone decreases testicular mass in males. Individual and model mean ± SEM for **A**. morning body mass; **B**. serum corticosterone concentrations; comparisons between 0 and 2  mg/kg treatment at each hour; **C**. percent change in body mass after adult treatment. **D**. adrenal mass; **E**. normalized adrenal mass; **F**. seminal vesicle mass; **G**. normalized seminal vesicle mass; **H**. testicular mass; and **I**. normalized testicular mass. Some error bars are obscured by mean line. Vehicle (0  mg/kg): 11 litters and 19 mice, except for adrenal mass with 18 mice; corticosterone (2  mg/kg): 11 litters and 17 mice. * *p *< 0.05, ** *p *< 0.01, *** *p *< 0.001. Results for the full statistical models are in Tables 4-5 and 4-6. Download Figure 4-3, TIF file.

10.1523/ENEURO.0125-24.2024.t4-1 Table 4-1 Number of litters and mice with serum corticosterone measurements before and after adult treatment. Download Table 4-1, DOCX file.

10.1523/ENEURO.0125-24.2024.t4-2 Table 4-2 Statistics for serum corticosterone in male and female offspring. Data from males were fit with the linear mixed model equation log_10_(cort) ∼ early-life treatment * adult treatment * time + (1 | mouse) + (1 | dam). Data from females were fit with the linear mixed model equation log_10_(cort) ∼ cycle stage * early-life treatment * adult treatment * time + (1 | mouse) + (1 | dam). Cycle stage is diestrus vs proestrus; early-life treatment is STD vs LBN rearing; adult treatment is CON vs ALPS; time is pre (0  h) vs post (5  h). Download Table 4-2, DOCX file.

10.1523/ENEURO.0125-24.2024.t4-3 Table 4-3 Number of litters and male mice with mass measurements on the day of adult treatment. Lower numbers for some tissue masses are attributable to loss of or damage to tissue at dissection. Download Table 4-3, DOCX file.

10.1523/ENEURO.0125-24.2024.t4-4 Table 4-4 Statistics from linear mixed models of male masses on day of adult treatment. Data were fit with the formula feature ∼ early-life treatment * adult treatment + (1 | dam). Early-life treatment is STD vs LBN rearing; adult treatment is CON vs ALPS. Download Table 4-4, DOCX file.

10.1523/ENEURO.0125-24.2024.t4-5 Table 4-5 Number of litters and female mice with mass measurements on the day of adult treatment. Adrenal masses were not collected from females used for electrophysiology studies (Figures 6-7). Lower numbers for some tissue masses are attributable to loss of or damage to tissue at dissection. Download Table 4-5, DOCX file.

10.1523/ENEURO.0125-24.2024.t4-6 Table 4-6 Statistics from linear mixed models of female masses on day of adult treatment. Data were fit with the formula feature ∼ early-life treatment * adult treatment * cycle stage + (1 | dam). Early-life treatment is STD vs LBN rearing; adult treatment is CON vs ALPS; cycle stage is diestrus vs proestrus. Download Table 4-6, DOCX file.

10.1523/ENEURO.0125-24.2024.t4-7 Table 4-7 Statistics from linear mixed models of male masses on day of vehicle (0  mg/kg) or corticosterone (2  mg/kg) treatment. Data were fit with the formula feature ∼ dosage + (1 | dam). Download Table 4-7, DOCX file.

10.1523/ENEURO.0125-24.2024.t4-8 Table 4-8 Statistics for serum corticosterone in males with vehicle or corticosterone administration. Data were fit with the linear mixed model equation log_10_(cort) ∼ dosage * time + (1 | mouse) + (1 | dam). Dosage is 0  mg/kg vs 2  mg/kg; time compares 0  h, 1  h, 2  h, 3  h, 4  h, and 5  h. Download Table 4-8, DOCX file.

Body mass was monitored before and after treatment (males, Extended Data Fig. 4-1; Extended Data Tables 4-3, 4-4; females, Extended Data Fig. 4-2; Extended Data Tables 4-5, 4-6). Consistent with the weekly monitoring of body mass in early adulthood, LBN males were smaller than STD males at the start of the experiment (Extended Data Fig. 4-1*A*; Extended Data Table 4-4; Table 2, Row 28, *p *= 0.037), and LBN did not affect the initial body mass in females (Extended Data Fig. 4-2*A*; Extended Data Table 4-6). ALPS animals of both sexes lost a greater percentage of body mass during treatment (males, Extended Data Fig. 4-1*B*; Extended Data Table 4-4; Table 2, Row 30, *p *< 0.001; females, Extended Data Fig. 4-2*B*; Extended Data Table 4-6; Table 2, Row 39, *p *< 0.001), likely attributable in part to no access to food or water during the last 4 h of stress treatment. By chance, morning body mass of ALPS males was greater than CON males (Extended Data Fig. 4-1*A*; Extended Data Table 4-4; Table 2, Row 29, *p *= 0.008), complicating interpretation of these observations, but males had apparent changes following ALPS in the normalized mass of seminal vesicles (Extended Data Fig. 4-1*F*; Extended Data Table 4-4; Table 2, Row 34, *p *= 0.048) and mass of the testes (absolute, Extended Data Fig. 4-1*G*; Extended Data Table 4-4; Table 2, Row 36, *p *= 0.003; normalized, Extended Data Fig. 4-1*H*; Extended Data Table 4-4; Table 2, Row 37, *p *= 0.021). Females did not exhibit changes in either uterine or adrenal mass following ALPS (Extended Data Fig. 4-2; Extended Data Table 4-6). Small changes in organ masses in males and females associated with LBN treatment are in Extended Data Tables 4-4 and 4-6 and Table 2 (Rows 31, 33, 35, and 40).

To test if corticosterone could reproduce the effects of ALPS on organ masses in males, additional mice were fed corticosterone or vehicle (Extended Data Fig. 4-3; Extended Data Tables 4-7, 4-8). Corticosterone decreased testicular mass (Extended Data Table 4-7; absolute, Extended Data Fig. 4-3*H*; Table 2, Row 51, *p *= 0.010; normalized, Extended Data Fig. 4-3*I*; Table 2, Row 52, *p *= 0.085), suggesting this parameter may be sensitive to stress.

### ALPS decreased afternoon LH in proestrous mice; LBN had no additional effect

**Figure 5.**
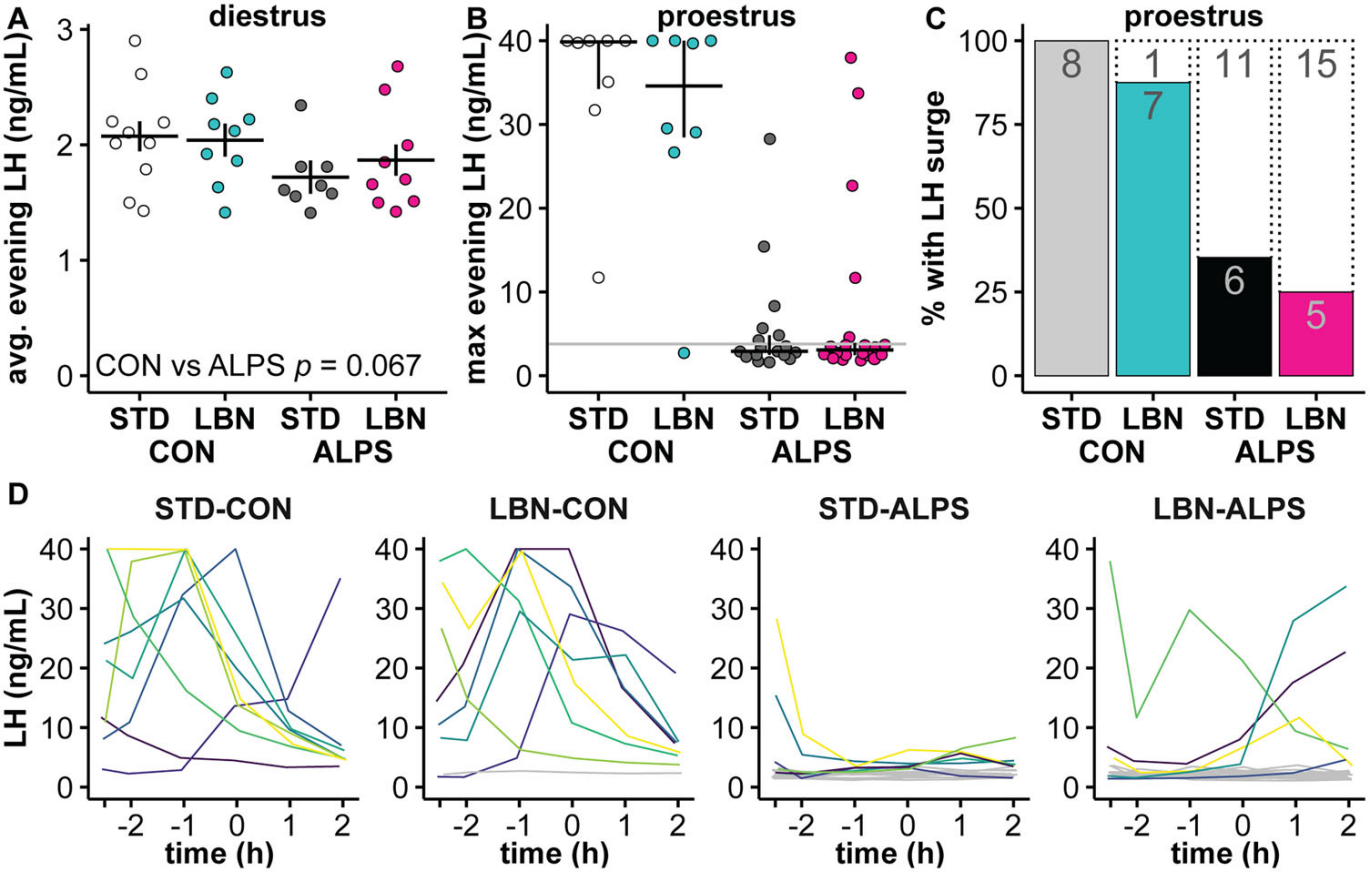
The LH surge is disrupted by adult stress. ***A***, Individual values and model mean ± SEM for the average LH concentrations on diestrus. ***B***, Individual values and median ± interquartile range of maximum evening LH for proestrous mice. The gray line at 3.8 ng/ml is the cutoff for an LH surge. ***C***, The percentage of proestrous mice with a maximum LH concentration >3.8 ng/ml (filled bars; numbers are counts per result). ***D***, Individual LH profiles for proestrous mice in each treatment group sampled hourly until 2 h after lights out; the time is relative to lights out. Gray lines show mice with no LH concentrations above 3.8 ng/ml. Results from the full statistical model of diestrous concentrations are in Extended Data Table 5-1. STD, standard-reared; LBN, limited bedding and nesting; CON, adult control; ALPS, acute, layered, psychosocial stress in adulthood.

Samples for LH collected at the end of the ALPS paradigm and at lights out from diestrous mice were averaged (STD-CON, nine litters and 10 mice; STD-ALPS, eight litters and 8 mice; LBN-CON, seven litters and 9 mice; LBN-ALPS, nine litters and 9 mice). Neither early-life stress (*p *= 0.687) nor adult stress (*p *= 0.067) had an effect on mean PM LH concentrations on diestrus (Fig. 5*A*; Extended Data Table 5-1), though the effect of adult stress approached the level set for significance. We tested if LBN alters the ability of ALPS to disrupt the proestrous LH surge (Wagenmaker and Moenter, 2017; STD-CON, 7 litters and 8 mice; STD-ALPS, 11 litters and 16 mice; LBN-CON, 8 litters and 8 mice; LBN-ALPS, 14 litters and 19 mice). The maximum observed LH, the proportion of mice with an LH surge, and individual LH concentration profiles are in Figure 5*B–D*, respectively. Adult stress decreased the proportion of proestrous mice with an LH surge (logistic regression, *χ*^2^ = 26.12; *p *< 0.001), but exposure to early-life stress did not change the likelihood of observing an LH surge (*χ*^2^ < 0.01; *p *= 0.991; Fig. 5*C*).

10.1523/ENEURO.0125-24.2024.t5-1 Table 5-1 Statistics from linear mixed models of average LH in diestrous mice on day of adult treatment. Data were fit with the formula average LH ∼ early-life treatment * adult treatment + (1 | dam). Early-life treatment is STD vs LBN treatment; adult treatment is CON vs ALPS treatment. Download Table 5-1, DOCX file.

### Neither LBN nor ALPS reduced the frequency of GABA PSCs in GnRH neurons

**Figure 6.**
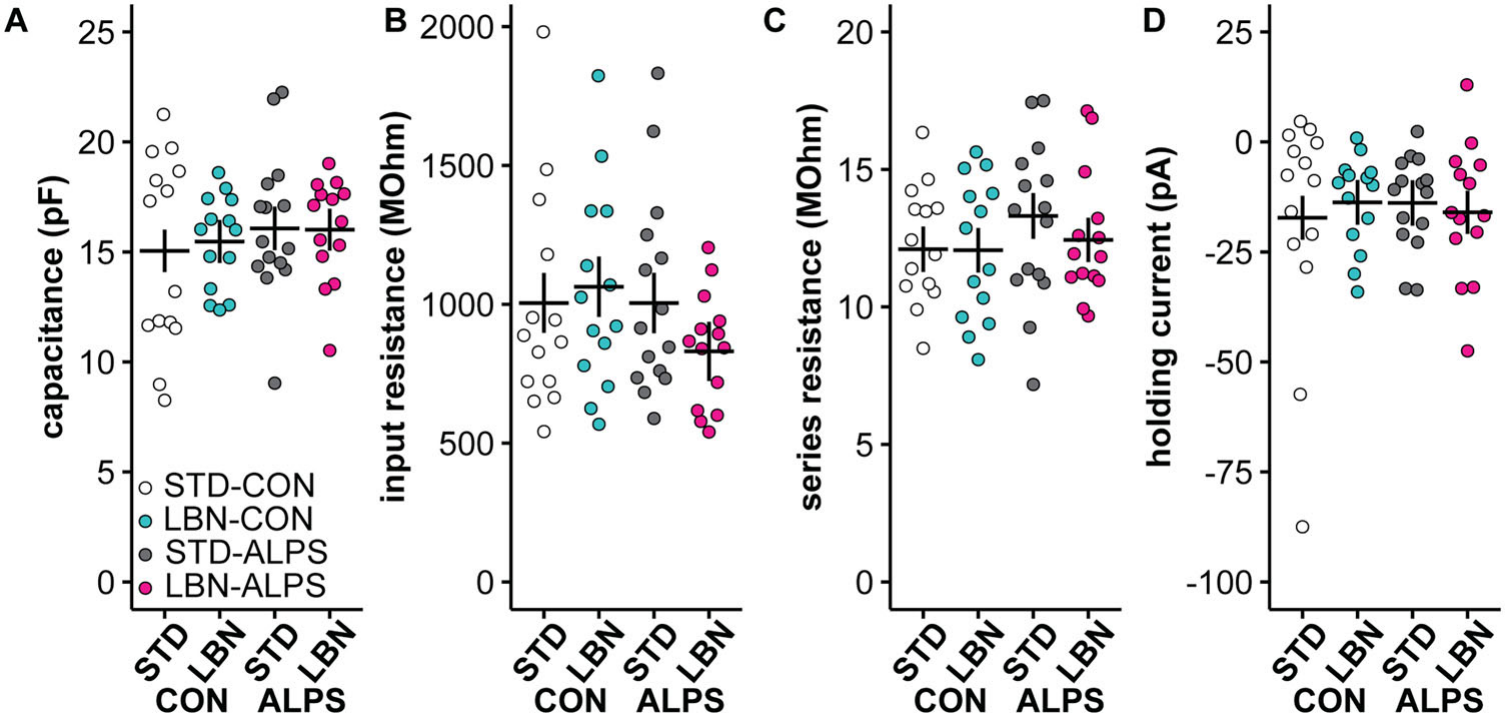
Recording quality and passive properties of GnRH neurons were similar among groups. ***A–D***, Individual cell values and model mean ± SEM for ***A***, capacitance; ***B***, input resistance; ***C***, series resistance; ***D***, holding current. Results for the full statistical model are in Extended Data Table 6-1. STD, standard-reared; LBN, limited bedding and nesting; CON, adult control; ALPS, acute, layered, psychosocial stress in adulthood.

**Figure 7.**
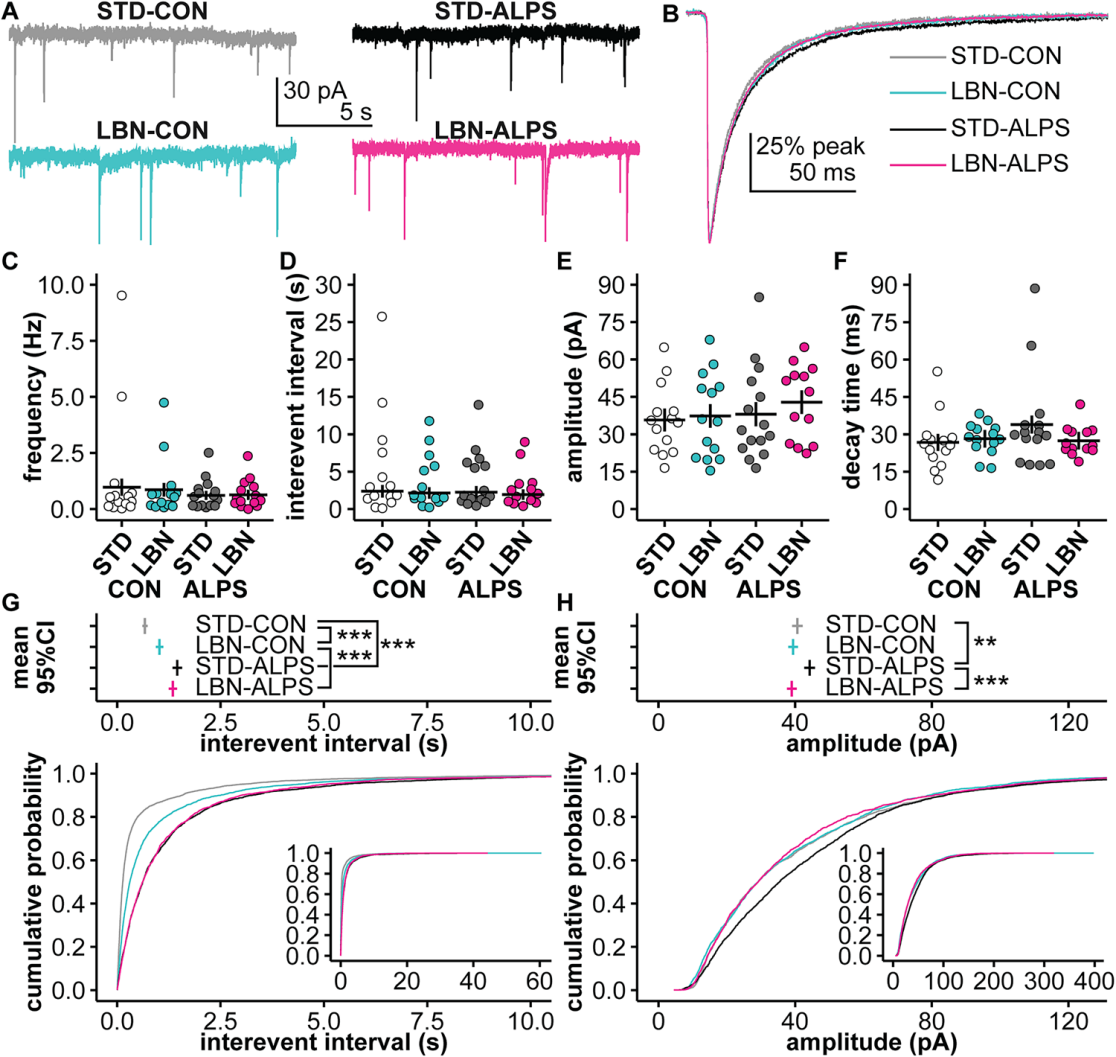
Stress treatments did not alter overall GABA PSC frequency, but ALPS may lengthen the interevent interval in GnRH neurons. ***A***, Representative 15 s traces (Box 9 smoothed) near the median frequency and amplitude from a GnRH neuron in each group. ***B***, Normalized average PSC for each treatment group. ***C–F***, Individual cell values and model mean ± SEM for ***C***, PSC frequency (number of events/duration); ***D***, mean interevent interval; ***E***, mean amplitude; and ***F***, decay time from 80 to 20% of peak calculated from the cell's normalized average trace. ***G***, ***H***, Distribution of (***G***) interevent interval and (***H***) amplitude. Top, Bootstrapped mean estimates with 95% confidence interval for each group. Bottom, Cumulative probability distribution plots for each group. Inset plots show the full range of the distribution. Results for the full statistical models are in Extended Data Tables 6-1, 7-1, and 7-2. *p < 0.05, **p < 0.01, ***p < 0.001, from bootstrapped results. STD, standard-reared; LBN, limited bedding and nesting; CON, adult control; ALPS, acute, layered, psychosocial stress in adulthood; PSC, postsynaptic current.

The frequency of GABA PSCs in GnRH neurons increases around the time of the LH surge on proestrus (Adams et al., 2018). We thus tested the hypothesis that ALPS decreases the frequency of these PSCs (STD-CON, 14 cells; STD-ALPS, 15 cells; LBN-CON, 15 cells; LBN-ALPS, 14 cells; five litters and six mice in all groups). There were no differences in the passive properties or recording quality among treatment groups (Fig. 6*A–D*; Extended Data Table 6-1). Representative PSC recordings from neurons in each group are in Figure 7*A*; the average PSC from each group is in Figure 7*B*. Neither LBN nor ALPS altered the frequency of GABA PSCs (Fig. 7*C*; Extended Data Table 7-1). Shifts in PSC patterns can occur within datasets with the same mean; however, when averaged by cell, the interevent interval of GABA PSCs was also similar among groups (Fig. 7*D*; Extended Data Table 6-1; *n* as above except LBN-ALPS *n* = 13 as one cell did not have PSCs). There were no differences in mean amplitude (Fig. 7*E*) or decay time (Fig. 7*F*; Extended Data Table 6-1). In contrast, the cumulative distribution of interevent intervals for all events is shifted toward longer intervals in the ALPS groups compared with those in the CON groups (Fig. 7*G*; Extended Data Table 7-2; pairwise AD tests; STD-CON vs STD-ALPS, *p* < 0.001; LBN-CON vs LBN-ALPS, *p *< 0.001).

10.1523/ENEURO.0125-24.2024.t6-1 Table 6-1 Statistics from linear mixed models of electrophysiology properties on day of adult treatment. The mean value for each cell was calculated, and data were fit with the formula feature ∼ early-life treatment * adult treatment + (1 | dam) + (1 | mouse). Early-life treatment is STD vs LBN rearing; adult treatment is CON vs ALPS. Download Table 6-1, DOCX file.

10.1523/ENEURO.0125-24.2024.t7-1 Table 7-1 Statistics for number of postsynaptic current (PSC) events per 240  s in GnRH neurons on the day of adult treatment. As the frequency data were skewed right and included zeros, a generalized linear mixed effects negative binomial model was used. Data were fit with the model equation # events per 240  s ∼ early-life treatment * adult treatment + (1 | mouse) + (1 | dam). The joint_tests function of the emmeans package was used to obtain these p-value estimates from the model. Early-life treatment is STD vs LBN rearing; adult treatment is CON vs ALPS. Download Table 7-1, DOCX file.

10.1523/ENEURO.0125-24.2024.t7-2 Table 7-2 Pairwise comparisons of distributions of interevent interval and amplitude for PSCs recorded in GnRH neurons. The Anderson-Darling criterion (AD), the standardized test statistic (T AD), and asymptotic p-value were calculated with the kSamples package (Scholz & Zhu, 2023). Bootstrapping was used to estimate the mean difference. The confidence interval is not adjusted for multiple comparisons. The p-values for both tests were adjusted using Holm’s method for multiple comparisons. Download Table 7-2, DOCX file.

Because of the large number of events in each group, the Anderson–Darling test could be identifying significant differences in the distribution that are not biologically relevant. To determine the effect size of these changes, we used bootstrapping to estimate 95% confidence intervals for the differences in means between groups. The mean interevent interval for events from STD-ALPS mice was estimated to be 0.78 s (95% CI, [0.65, 0.92]) longer than for events from STD-CON mice; in LBN mice, ALPS lengthened mean interevent interval by ∼0.32 s (95% CI, [0.19, 0.46]). This suggests that ALPS alters the excitatory input to GnRH neurons on the evening of proestrus. We interpret these results with caution, however, as three CON cells with high PSC frequencies contribute a disproportionate number of short interevent intervals.

The amplitude distribution for the STD-ALPS groups was shifted toward larger events (Fig. 7*H*; Extended Data Table 7-2; pairwise AD tests; STD-CON vs STD-ALPS, *p *= 0.006; STD-ALPS vs LBN-ALPS, *p *< 0.001). In LBN mice, ALPS did not shift the amplitude distribution (Extended Data Table 7-2; pairwise AD tests; LBN-CON vs LBN-ALPS, *p *= 0.711). The mean amplitude of the STD-ALPS group was ∼3.6 pA larger (95% CI, [1.36, 5.81]) than the mean of the STD-CON group and ∼5.2 pA larger (95% CI, [3.09, 7.34]) than the mean of the LBN-ALPS group, as estimated with bootstrapping.

## Discussion

The stress and reproductive neuroendocrine systems interact, and early-life stress has reproductive consequences in humans. We tested the hypotheses that early-life stress in the form of resource scarcity provided through LBN would delay sexual maturation and alter the response to subsequent stress exposure in adulthood in mice. LBN, as applied in the present study, did not delay external indicators of sexual maturation in males or females. Furthermore, the corticosterone response to adult psychosocial stress was not altered by LBN in either sex. On proestrus, adult stress disrupts the LH surge, but this is not affected by a history of LBN. In contrast to our hypothesis that ALPS disrupts the LH surge by decreasing the frequency of excitatory GABA PSCs in GnRH neurons, these currents were not appreciably altered by either adult psychosocial stress or LBN. This suggests that the disruption of the LH surge by adult stress is not attributable to changes in the GABAergic input to GnRH neurons.

These findings lead us to reject our hypotheses, but it is important to point out that this rejection could be for at least two reasons. First, the hypotheses could simply be wrong. Second, CBA dams were more resilient to effects of LBN treatment than strains previously used, as indicated by a lack of persistently elevated serum corticosterone concentrations on PND11. Such a milder maternal effect could have diminished disruptions by this early-life resource scarcity on pups’ neuroendocrine maturation.

The LBN paradigm was chosen as the model for early-life stress because animal behavior is minimally disrupted by ongoing investigator interference (Walker et al., 2017). Over its implementation in several labs, LBN effectiveness has been evaluated in three main ways: dam behavior, pup mass at the end of the paradigm, and/or dam stress parameters. In both rats (Brunson et al., 2005; Ivy et al., 2008; Molet et al., 2016) and mice (Rice et al., 2008; Gallo et al., 2019), maternal care is fragmented by LBN treatment, leading to more transitions between behavioral states and more exits from the nest. This fragmentation of maternal care was confirmed in CBA dams in the present study. On the last day of treatment, mice and rat offspring of LBN dams exhibit elevated basal corticosterone concentrations (Gilles et al., 1996; Avishai-Eliner et al., 2001; Brunson et al., 2005; Rice et al., 2008; Naninck et al., 2015) and increased adrenal masses in rat pups (Avishai-Eliner et al., 2001; Brunson et al., 2005). Because the aforementioned outcomes require terminal studies in neonates, monitoring of body mass is a common proxy measure of the impact of LBN treatment in offspring, with LBN pups being smaller than STD pups after the paradigm (Gilles et al., 1996; Avishai-Eliner et al., 2001; Brunson et al., 2005; McLaughlin et al., 2016; Moussaoui et al., 2016; Knop et al., 2019, 2020; Manzano Nieves et al., 2019; Eck et al., 2020). We similarly observed a lower pup mass at the end of the LBN paradigm in both male and female CBB6/F1 hybrid offspring, suggesting that the LBN paradigm was effectively implemented as an early-life stressor in our laboratory.

Strain can impact dam behavioral responses to LBN and the effect on offspring mass in mice (Pardo et al., 2023), and there are indications that the LBN treatment may induce a milder phenotype in CBA dams and their CBB6/F1 hybrid offspring in the present study. Morning plasma corticosterone concentrations were elevated in rat dams at the end of the paradigm on PND9 (Ivy et al., 2008); serum corticosterone was not, however, elevated on PND11 in CBA dams. Adaptation to psychosocial stressors resulting in decreased glucocorticoid output has been observed in mice (Wagenmaker and Moenter, 2017) and ewes (Wagenmaker et al., 2010). Perhaps such acclimation occurs more quickly in CBA dams than in other species or strains, which could contribute to milder outcomes in their pups. The PND11 difference in body mass resolved quickly in female offspring, and there were no further impacts of LBN treatment on body mass growth through adulthood of the females we studied. In contrast, LBN males had mildly slowed growth that was evident later in adulthood. This suggests there may also be a difference in susceptibility to LBN treatment between the female and male offspring.

The effects of LBN on reproductive maturation vary across studies (Knop et al., 2019, 2020; Manzano Nieves et al., 2019; Davis et al., 2020; Eck et al., 2020). In the present study, we did not observe differences in body mass near the time of puberty or changes in the age at reproductive maturation, in part because we normalized litter sizes to provide more consistent nutrition. Some of the variations in the literature may be related to age of LBN exposure and/or subtle protocol differences such as the type of bedding or nesting material, the wire platform material, or vivarium conditions, along with the species and strain of animals. The variability in body mass may also underlie some of the variability in vaginal opening outcomes, as a decrease in body mass is known to delay vaginal opening (R. E. Nelson and Robison, 1976; Kirkpatrick and Rutledge, 1987; Castellano et al., 2011; Caron et al., 2012; Wu et al., 2016). Together, these studies point to the importance of considering possible confounding and interacting factors when assessing the effect of early-life stress on body mass and reproductive maturation.

A lack of effect of LBN on the estrous cycle was a more consistent observation across studies in both mice (Manzano Nieves et al., 2019) and rats (Davis et al., 2020; Eck et al., 2020) and was confirmed in the present study. Estrous cycles also remain unchanged following maternal separation as an early-life stress (Rhees et al., 2001; Ferraz et al., 2023). Although the observation of typical adult estrous cyclicity does not preclude other underlying changes in reproductive physiology (Wang and Moenter, 2020), the ability of animals exposed to early-life stress to establish cyclicity is an indication that aspects of the reproductive system can recover from developmental perturbations caused by this treatment.

Our findings that LBN did not alter adult basal corticosterone concentrations in the morning or afternoon are consistent with the observations of others (Brunson et al., 2005; Knop et al., 2019, 2020; Eck et al., 2020). The original study of LBN in mice did, however, observe persistently elevated basal corticosterone concentrations in 4–7 month-old males (Rice et al., 2008). In one study, adult LBN rats responded similarly to STD-reared rats when exposed to a 1 h restraint stress (Eck et al., 2020). The latter is consistent with our finding that adult LBN mice exhibited similar corticosterone profiles to STD-reared mice in response to a 5-h, layered stress paradigm, ALPS, which included restraint. This suggests that any transient changes in the neuroendocrine stress response following perinatal LBN exposure were normalized by subsequent rearing and weaning into STD housing conditions.

The primary motivation for this work was to study the reproductive consequences of LBN, including how it affects responses to ALPS exposure. In diestrous mice, the ALPS effect to reduce mean LH concentrations approached the value accepted for significance. This may reflect stress suppression (Yang et al., 2017) of the pulsatile LH release typical of this stage (Czieselsky et al., 2016), but the infrequent sampling in the present study is not designed to assess pulse parameters. The ALPS paradigm was developed in the context of understanding the effects of acute stress exposure on the sustained preovulatory increase in LH concentration that occurs on the afternoon of proestrus. As reported (Wagenmaker and Moenter, 2017), ALPS initiated on the morning of proestrus disrupts the LH surge in most mice. We hypothesized LBN exposure would alter the effect of ALPS on the LH surge, but LBN had no additional effect, suggesting that the paradigm studied for early-life stress did not confer either resilience or susceptibility to the adult stress studied for this parameter. There are several potential explanations for this finding. First, LBN may be milder than other perinatal stressors, such as lipopolysaccharide exposure (Li et al., 2007), that have lasting effects on the reproductive consequences of adult stress. Second, the preweaning return to STD housing conditions may have also buffered the effects of early resource limitations. Third, the work by Peña et al. (2019) demonstrated the challenges of trying to predict if experiencing one stressor will lead to susceptibility or resilience to a subsequent stressor by comparing the impact of early-life stressors on the behavioral responses to 10 d of chronic social defeat. Most pertinent to the work presented here, maternal separation with reduced bedding from PND2 to 12 did not affect postdefeat behavior, whereas the same treatment from PND10 to 17 increased susceptibility (Peña et al., 2019), a difference which the authors attributed to the transition out of the stress hyporesponsive period for pups who experienced the paradigm later in development (Rincón-Cortés and Sullivan, 2014). The lack of effect of LBN from PND4 to 11 could thus be reflective of pups experiencing the paradigm during the stress hyporesponsive period.

The mechanisms by which ALPS disrupts the LH surge, both in terms of the components of the stress response and the site of action within the reproductive axis, remain unknown. GnRH neurons from proestrous mice receive a higher frequency of GABAergic input, which is excitatory in these cells (DeFazio et al., 2002), in the evening than in the morning, consistent with the switch from negative to positive feedback and the timing of the LH surge (Adams et al., 2018). We thus tested the hypothesis that GABA input to GnRH neurons is diminished by ALPS. The frequency of GABAergic PSCs in GnRH neurons was not altered by stress, nor was the mean interevent interval by cell. The distribution of all interevent intervals was shifted toward longer intervals in cells from ALPS mice, but this appears to be primarily related to three CON cells with a high frequency of PSCs. It is possible that one mechanism by which ALPS ultimately disrupts the LH surge is by reducing the incidence of GnRH neurons receiving a high frequency of GABAergic input, but the current study is not powered to assess this. The observation that the amplitude distribution of PSCs from STD-ALPS mice is shifted toward larger events runs counter to the hypothesis that this stressor reduces the efficacy of GABA input to GnRH neurons. The magnitudes of these observed changes in event distributions are small and near our limit of detection for differentiating signal and noise in these electrophysiological recordings. Amplitude did not differ when comparing the mean values from each cell; thus, the subtle shifts in the cumulative probability distributions of events may also reflect some bias toward the amplitude of PSCs from cells with more events included in the analysis.

One possible caveat to this work is that brain slice preparation could sever key neuronal networks that may be critical for the disruption of reproductive output following ALPS or that other in vivo changes attributable to early-life or adult stress do not persist in this configuration. In this regard, both acute and chronic stressors induced measurable changes in synaptic physiology of the hypothalamic paraventricular nucleus in brain slices (Levy and Tasker, 2012). The increase in GABA PSC frequency in GnRH neurons on the evening of proestrus occurs concurrently with the expected time of the LH surge, but the experimental design precludes the ability to directly correlate the properties of PSCs to the incidence of the LH surge in that animal; in this regard, uterine mass in animals used for PSC recordings were consistent with proestrus (Extended Data Fig. 4-2). The source of the increased GABAergic transmission to GnRH neurons during the LH surge is not known and has been postulated to be the suprachiasmatic nucleus (Christian and Moenter, 2007) or the anteroventral periventricular kisspeptin neurons (Wang and Moenter, 2020). Although GABAergic transmission was not altered by ALPS or LBN, it is possible that peptidergic modulators from these, or other, populations are altered in a manner that disrupts the LH surge.

While persistent effects of LBN were not observed into adulthood in this study, care must be taken not to overextrapolate these findings as demonstrating that early-life psychosocial and environmental manipulations are of no consequence. To allow rigorous control of conditions, this study considered a single type of early-life stress during 1 week of development, after which time animals were returned to STD housing conditions. Altering the timing, type, or duration of the stressor may lead to different outcomes. Indeed, a recent study found persistent reproductive effects following 3 weeks of postweaning social isolation, including on vaginal opening, estrous cycles, and activity of hypothalamic neurons (Agus et al., 2024), indicating that housing conditions during certain developmental periods can lead to changes that persist into adulthood. The observations of the present study direct future attention to the effects of both early-life and adult stress on the broader neuroendocrine network controlling reproduction, including upstream neuronal populations and pituitary gonadotropes.

## Synthesis

Reviewing Editor: Frederike Beyer, Queen Mary University of London Decisions are customarily a result of the Reviewing Editor and the peer reviewers coming together and discussing their recommendations until a consensus is reached. When revisions are invited, a fact-based synthesis statement explaining their decision and outlining what is needed to prepare a revision will be listed below. The following reviewer(s) agreed to reveal their identity: Tallie Z. Baram.

As you can see from their comments below, both reviewers highlight the interest in your work and the relevance of publishing null findings. However, they also raise important points that need to be addressed. In particular, please consider the points raised by reviewer 1 concerning the (lack of) evidence for the effectiveness of the stress induction as implemented here. In line with their comments, please clarify in your manuscript whether your data show that early life stress does not have the hypothesised impact (in which case you need to add clear evidence of your experimental manipulation inducing stress in dams and their pups), or that whether the experimental manipulation did not induce stress in either dams or pups (in which case the title and conclusions should be amended accordingly). For detailed comments, please see the reviews below.

Reviewer 1 comments:

This is a careful, interesting study aiming to determine if early-life chronic stress induced by the simulated poverty 'limited bedding and nesting' paradigm developed by Gilles et al., (1996) influences both reproductive function and adult responses to stress.

The authors found that the age and body weight of female mice at vaginal opening, first estrus, and preputial separation in males were not affected by the LBN as applied by the authors. In addition, basal

corticosterone concentrations were similar between males, diestrous females, or proestrous

females reared in standard or LBN environments. After exposure to a stress called acute layered psychosocial stress (ALPS), serum corticosterone concentrations were also similar between standard and LBN reared offspring, and the effect of this stress on the luteinizing hormone (LH) surge did not distinguish the LBN from the control groups

The authors then aim to test a mechanism for the ALPS disruption of the LH surge and refute the notion that this is a result of increased in excitatory GABAergic input to gonadotropin-releasing hormone (GnRH) neurons on the afternoon of proestrus.

The authors conclude that early-life chronic stress using the LBN model does not confer either susceptibility or resilience to stress, early puberty or the effects of adult stress on the reproductive regulation of LH in mice.

The authors are commended for summarizing their 'negative data' and by and large present their results clearly. The main, serious flaw of the paper is their inadequate application of the LBN model to the specific strain of mice they used. In essence, they were not successful in stressing the dam or the pups, which, not surprisingly, led to absence of any long-lasting effects of the planned stress.

As described in Walker et al., cited by the authors, and in prior and other protocols (e.g., Ivy et al., 2008, Molet et al., 2016) the purpose of limited bedding and nesting in the cages is to stress the dam. This influences important parameters of her behavior towards pups and triggers enduing molecular, cellular and circuit changes (e.g., Pena 2017; Short et al., 2023; Bolton et al., 2020 and many others). The amount of deprivation from nesting and bedding materials needs to be calibrated for a given mouse or rat strain to generate stress in the dam and in the pups. In figure 1 the authors clearly demonstrate that dam plasma corticosterone levels in LBN cages were not elevated compared with the controls. Hence, the dams were not stressed. (BTW, in panel F in that figure, is the Y axis title correct?).

A second hallmark of pup stress is pups/ plasma corticosterone on P11, and augmented adrenal weight, documenting the chronicity of the stress. These measures require invasive/ termina studies. However, pup stress is easily assessed through pups weight. As shown by Rice et al., and many others, mouse and rat pups gain weight more slowly during the LBN epoch, such that their weight on P9-11 is lower than that of controls. They typically catch up by weaning or shortly thereafter. Figure 2 clearly shows that pup mass/weight did not distinguish LBN from typically reared pups. Whereas the authors do not provide corticosterone levels or adrenal weight, this findings suggests that the pups were not stressed, and therefore should not be expected to differ from the controls in subsequent experiments.

Because the authors provide no evidence that the pups were effectively stressed, i.e., that they implemented the LBN model in the manner it was designed to be used, their conclusions that the LBN model is ineffective in altering neuroendocrine systems is simply not justified.

So, what to do?

This reviewer sympathizes with the significant effort exerted by the authors, and believes that the studies they present will enrich the literature if interpreted and presented in a constructive manner:

1. The authors essentially describe the resilience of the GnRH-GFP (Tg(Gnrh1-EGFP)51Sumo

88 MGI:6158457, C57Bl6/J) female mouse to environments inducing stress.

The title should focus on this finding (e.g., "GNRH female mice are resilient to stress-inducing environments, protecting their pups' sexual maturation and stress vulnerabilities) .

2. They can then show in their figures the resilience of the dams (including the disproportional weight gain that might protect the pups development)

3. Then, in a figure titled- pups of GnRH dams are not stressed- I,e, there is no acute effect highlight the protection by the dam

4. Another figure will be titled as resilience to latent effects on sexual development or adult stress.

As an aside it is interesting that there are no enlarged adrenals in adult LBN mice. chronically stressed mice in the LBN paradigm have enlarged adrenals already on P10, and this persist to adulthood (see Bolton et al., cell reports 2022).

Regarding the second part of the paper that deals with the mechanisms of the LH surge: because the authors don't really have ELA in their ::LBN" group, it is difficult to justify showing the groups separately.

One would suggest excluding that group from the results, or, alternatively, concluding that because these pups were never stressed they can be combined with the control group.

Other points:

The authors nicely point out that the effect of early-stress on the velocity of development including puberty is complex. As they point out, litter size governs the rate of growth, and lack of careful control might explain the difference between Kevin Bath's and other groups.

In the revamped discussion, it would be helpful to discuss the origin and principles of the LBN paradigm and how to ascertain its effectiveness

Reviewer 2 comments:

The authors presented a well-supported manuscript with clear and punctual hypotheses addressed with well-designed experiments, rigorous statistical analysis, and focused discussion.

Under the premise that early stress, induced by limited nest material along seven postnatal days and acute stress during adulthood, alters the development of sexual characters and periovulatory luteinizing hormone surge. Also, the authors evaluate the effect of both stressors on GABAergic neurons that modulate the GnRH release. The authors describe the no effects of both stressors on several variables that are indexes of sexual external characteristics. These results are a relevant contribution to future studies using this approximation and for constructing animal models to evaluate the effect of early stress on reproduction.

The methods are well described, and the results are well presented and described. One strength of the manuscript is that the discussion is centered on present results and offers pertinent limitations.

A few issues require attention before it is published.

1. The introduction section lacks information about how stress affects the activity of GABAergic neurons and, consequently, this impact on GnRH release.

2. In relation to the administration of corticosterone to verify the effect of this hormone on sexual characters, authors must justify the dose used.

## Author Response

We thank the reviewers for their additional helpful and constructive feedback. Our responses are below, demarcated with [Author response], detailing how we have used these comments to further strengthen the manuscript.

Synthesis Statement for Author (Required):

The reviewers and I appreciate the improvements made to the manuscript in the revisions. However, given the results suggest the effects of the stress manipulation were weaker and more short-lived than expected, some more explicit discussion of the null findings should be included. Please consider the reviewer's comments below in your revision of the manuscript:

We appreciate the opportunity to review the revised paper and the additional clarifications. However, a fundamental issue requires resolution:

The purpose of publishing 'negative results' is to allow the authors and readers to learn from the experiments and the experience. A priori, the authors set out with the belief that early-life stress will alter adult stress-induced changes in sex hormone dynamics and the general response to additional stresses later in life. Their results seem to not support the hypothesis. The main asset of the paper is to explain why. It is conceivable that the hypothesis is wrong, alternatively, as is the case here, the authors did not accomplish stress in the dam. This is the crucial element of the resource scarcity/LBN model. Stressed dams have many alterations in behaviors, and these aberrant sensory signals to the pups' brain disrupt brain circuit maturation. [Author response] We would argue that these two possibilities are not alternatives, but may actually both be possibilities. The data we obtained do not support our hypothesis. This could be because the hypothesis is incorrect, but it is also possible that the early-life stress paradigm we used did not sufficiently stress the dams to produce lasting effects in the pups. Given that there is at least a transitory change both in dam behavior and in pup mass at the end of the paradigm, we are not comfortable stating there was zero stress to the dams and pups, but we have tried to clarify and highlight the valid possibility that the stress experienced may have not been sufficient or comparable to prior studies using this approach in both the abstract and discussion.

Thus, the key remaining issues are: a. The title. A proposed one, reflecting the facts above is: "Early-life resource scarcity without maternal stress does not alter adult stress- and sex- hormone responses to acute psychological stress" [Author response] We have changed the title to "Early-life resource scarcity in mice did not alter adult corticosterone or preovulatory luteinizing hormone surge responses to acute psychosocial stress." [Author response] We maintained the reference to the species used, along with the specific hormones that we measured, while removing reference to a specific early life stress model (i.e., LBN). b. The authors should alter the abstract accordingly [Author response] We have altered the abstract to include the following sentences to provide this possible explanation for the lack of effects in LBN offspring: [Author response] "In this study, the paucity of effects in the offspring may relate to a milder response of CBA dams to the paradigm. While LBN dams exited the nest more often, and their offspring were smaller than standard-reared offspring on PND11, dam corticosterone concentrations were similar on PND11." [Author response] We also conclude the abstract with the explicit statement: "It remains possible ... that a more pronounced resource scarcity would affect the parameters studied" c. The discussion might benefit from a few sentences describing the above [Author response] In the first paragraph of the discussion, we have added the clauses "early-life stress in the form of resource scarcity provided through LBN" and "LBN, as applied in the present study," to emphasize that the conclusions are limited to the scope of treatment as applied under these conditions in this strain of mice. [Author response] We have added a second paragraph to address the rejection of our hypotheses and pose the possible two explanations suggested above: [Author response] "These findings lead us to reject our hypotheses, but it is important to point out that this rejection could be for at least two reasons. First, the hypotheses could simply be wrong. Second, CBA dams were more resilient to effects of LBN treatment, as indicated by a lack of persistently elevated serum corticosterone concentrations on PND11. Such a milder maternal effect could have diminished disruptions by this early-life resource scarcity on pups' neuroendocrine maturation." ---------- Below is the author rebuttal from the initial round of revisions, which has been included here for completeness ---------- [Author response] We thank the reviewers for their helpful and constructive comments. We've used these to inspire additional analysis and improve the presentation of the work. Responses are below preceded by "[Author response]"; sometimes multiple comments are covered within a single response section that follows those comments.

Synthesis Statement for Author (Required):

As you can see from their comments below, both reviewers highlight the interest in your work and the relevance of publishing null findings. However, they also raise important points that need to be addressed. In particular, please consider the points raised by reviewer 1 concerning the (lack of) evidence for the effectiveness of the stress induction as implemented here. In line with their comments, please clarify in your manuscript whether your data show that early life stress does not have the hypothesised impact (in which case you need to add clear evidence of your experimental manipulation inducing stress in dams and their pups), or whether the experimental manipulation did not induce stress in either dams or pups (in which case the title and conclusions should be amended accordingly). For detailed comments, please see the reviews below. [Author response] Based on the feedback offered by the reviewers, we have changed the title to "Limited bedding and nesting does not alter adult corticosterone response to acute psychosocial stress in male or female mice or the stress-induced disruption of the preovulatory luteinizing hormone surge." This change shifts the focus to the specific paradigm as applied in this study and away from the claim that the paradigm induced early-life stress, although further analysis has provided an indication that the paradigm was, at least in part, effective.

Reviewer 1 comments:

This is a careful, interesting study aiming to determine if early-life chronic stress induced by the simulated poverty 'limited bedding and nesting' paradigm developed by Gilles et al., (1996) influences both reproductive function and adult responses to stress.

The authors found that the age and body weight of female mice at vaginal opening, first estrus, and preputial separation in males were not affected by the LBN as applied by the authors. In addition, basal corticosterone concentrations were similar between males, diestrous females, or proestrous females reared in standard or LBN environments. After exposure to a stress called acute layered psychosocial stress (ALPS), serum corticosterone concentrations were also similar between standard and LBN reared offspring, and the effect of this stress on the luteinizing hormone (LH) surge did not distinguish the LBN from the control groups The authors then aim to test a mechanism for the ALPS disruption of the LH surge and refute the notion that this is a result of increased in excitatory GABAergic input to gonadotropin-releasing hormone (GnRH) neurons on the afternoon of proestrus. The authors conclude that early-life chronic stress using the LBN model does not confer either susceptibility or resilience to stress, early puberty or the effects of adult stress on the reproductive regulation of LH in mice.

The authors are commended for summarizing their 'negative data' and by and large present their results clearly. The main, serious flaw of the paper is their inadequate application of the LBN model to the specific strain of mice they used. In essence, they were not successful in stressing the dam or the pups, which, not surprisingly, led to absence of any long-lasting effects of the planned stress. As described in Walker et al., cited by the authors, and in prior and other protocols (e.g., Ivy et al., 2008, Molet et al., 2016) the purpose of limited bedding and nesting in the cages is to stress the dam. This influences important parameters of her behavior towards pups and triggers enduing molecular, cellular and circuit changes (e.g., Pena 2017; Short et al., 2023; Bolton et al., 2020 and many others). The amount of deprivation from nesting and bedding materials needs to be calibrated for a given mouse or rat strain to generate stress in the dam and in the pups. In figure 1 the authors clearly demonstrate that dam plasma corticosterone levels in LBN cages were not elevated compared with the controls. Hence, the dams were not stressed. (BTW, in panel F in that figure, is the Y axis title correct?).

A second hallmark of pup stress is pups/ plasma corticosterone on P11, and augmented adrenal weight, documenting the chronicity of the stress. These measures require invasive/ termina studies. However, pup stress is easily assessed through pups weight. As shown by Rice et al., and many others, mouse and rat pups gain weight more slowly during the LBN epoch, such that their weight on P9-11 is lower than that of controls. They typically catch up by weaning or shortly thereafter. Figure 2 clearly shows that pup mass/weight did not distinguish LBN from typically reared pups. Whereas the authors do not provide corticosterone levels or adrenal weight, this findings suggests that the pups were not stressed, and therefore should not be expected to differ from the controls in subsequent experiments. [Author response] The reviewer makes a good point about body mass of the pups. We tracked body mass when the paradigm was set up and from the end of the paradigm through PND72 at varying intervals. As we had many observations of body mass, we selected a statistical approach that could model the change in body mass over time. To understand significant main effects and interactions from the omnibus linear mixed model, we made a limited number of post hoc comparisons at discrete postnatal days, which were corrected for multiple comparisons. As the reviewer pointed out, however, many previous studies focused on differences in body mass that occurred only at the end of the paradigm on PND9 or PND11, which we had not included. To provide a more direct comparison of the data in our study to these previous studies we conducted a linear mixed model to assess these main effects and interactions on PND11, with litter as the random effects variable. There is a significant effect of early-life treatment on the body mass (F(1, 47.03) = 8.26, p = 0.006), with the LBN mice (6.4g{plus minus}0.14) having a lower body mass than STD mice (7.0g{plus minus}0.13). There was no main effect of sex (F(1, 249.68) = 0.25, p = 0.616) or interaction of early-life treatment and sex (F(1, 249.68) = 2.38, p = 0.124). We have added this analysis, which permits direct comparison, as Figure 2B, as confirmation that the LBN did indeed have an effect consistent with what has been published. We also added Figure 2A to demonstrate that there was not an initial difference in body mass of offspring assigned to the two treatments at PND4. [Author response] We also thank the reviewer for catching the incorrect Y axis title for the right graph of panel E in Figure 1. The title indeed should have been "mean % time off nest" instead of "mean % off exits." We have also updated the left graph of panel E to include "mean % time off nest." Because the authors provide no evidence that the pups were effectively stressed, i.e., that they implemented the LBN model in the manner it was designed to be used, their conclusions that the LBN model is ineffective in altering neuroendocrine systems is simply not justified.

So, what to do? This reviewer sympathizes with the significant effort exerted by the authors, and believes that the studies they present will enrich the literature if interpreted and presented in a constructive manner:

1. The authors essentially describe the resilience of the GnRH-GFP (Tg(Gnrh1-EGFP)51Sumo 88 MGI:6158457, C57Bl6/J) female mouse to environments inducing stress.

The title should focus on this finding (e.g., "GNRH female mice are resilient to stress-inducing environments, protecting their pups' sexual maturation and stress vulnerabilities) . [Author response] The GnRH-GFP transgene was introduced through the males in our breeding scheme, and we have no data to support the hypothesis that this transgene would be protective to pups. We discuss the possibility of a strain effect below.

Title was changed as described above.

2. They can then show in their figures the resilience of the dams (including the disproportional weight gain that might protect the pups development) [Author response] We agree with the reviewer that it is possible that our application of the LBN model in the strain of mice utilized may have produced a more moderate stress and/or one that was not as prolonged as has been reported. There are several points to make in considering this. [Author response] First, the behavior of the dams was altered as shown in Figure 1. Thus, the paradigm had some effect and, although much of our later data are negative, this suggests that the CBA dams were not fully resilient to the treatment. [Author response] Second, the reanalysis of pup mass at the end of the paradigm demonstrates a smaller body mass in the LBN groups, suggesting a similar effect in the present study to published work. [Author response] Third, corticosterone levels were indeed not different at the end of the paradigm in LBN vs standard-housed dams. The p value for this comparison was 0.102, which approaches the level set for significance. We scanned the data of later pup measurements to see if pups from dams that had higher corticosterone values were overrepresented towards one end of any of the other measures, but this was not observed. There are at least a couple possible explanations. One is that corticosterone was never elevated during LBN treatment in these CBA dams. We think it is more likely that this strain under these conditions adapted more quickly to the LBN paradigm so that corticosterone had returned to the typical range by the end of the week-long treatment. Of interest in this regard, the number of exits from the nest in LBN dams began higher than standard-housed dams, peaked towards the start of the paradigm and achieved values similar to standard-housed dams by the end of the week. Adaptation to stress has been observed (for example, repeated daily exposures to psychosocial stressors in ewes resulted in diminished plasma cortisol responses over time, Wagenmaker et al. 2010, and repeated daily restraint stress in mice also leads to a diminished corticosterone response, Wagenmaker and Moenter. 2017). [Author response] Fourth, we only monitored activation of the adrenal component of the hypothalamo-pituitary-adrenal axis as a marker of stress; other stress pathways could be activated in a more persistent manner. [Author response] Fifth, the reviewer's suggestion that the dams used in this study, which were wild-type CBA dams, are resilient and protect their pups is an intriguing one. A postulate that strain has an effect on the severity of the stress for the dam and thus subsequently for the pups is reasonable. A more detailed account of how the LBN paradigm affects additional outcomes in CBA dams would be necessary to support this; these studies are beyond the scope of this current project but are an interesting future direction. With regard to the statement about a disproportional increase in mass in the LBN dams, we apologize the data were not clearer and have edited this section for clarity. The LBN dams had a greater body mass than the STD dams throughout our observation period (a main effect of treatment), but there was no interaction between postanal day and treatment. An interaction would have suggested that the change in body mass during and after the treatment was disproportionate between STD and LBN dams, but the lack of interaction indicates that the two groups had similar changes in body mass. There appears to be one large LBN dam which is primarily driving the overall treatment difference; her offspring do not appear to be outliers in terms of sexual maturation.

3. Then, in a figure titled- pups of GnRH dams are not stressed- I,e, there is no acute effect highlight the protection by the dam 4. Another figure will be titled as resilience to latent effects on sexual development or adult stress. [Author response] As described above, we altered figure 2 to include the initial reduction in offspring body mass by LBN on PND11, which demonstrates an acute effect of LBN treatment on body mass that resolves later in development. With this demonstration, we have kept the sexual maturation data in the same figure as the offspring mass data.

As an aside it is interesting that there are no enlarged adrenals in adult LBN mice. chronically stressed mice in the LBN paradigm have enlarged adrenals already on P10, and this persist to adulthood (see Bolton et al., cell reports 2022). [Author response] We appreciate this discussion of adrenal mass as another potential indicator of the effects of LBN on offspring. In male offspring, the p-value approached the level set for significance (p = 0.057) for normalized adrenal mass, with the direction of difference being a larger mass for LBN offspring. This could be further indication the effect of the LBN treatment applied is mild in this strain. In female offspring, there was not a difference in adrenal mass after normalizing for body mass (p = 0.547). We have added p values that were <0.1 to the figures for the reader's convenience.

Regarding the second part of the paper that deals with the mechanisms of the LH surge: because the authors don't really have ELA in their "LBN" group, it is difficult to justify showing the groups separately.

One would suggest excluding that group from the results, or, alternatively, concluding that because these pups were never stressed they can be combined with the control group. [Author response] Because dam behavior was altered by the LBN paradigm and because the new analysis of pup mass shows a different at the end of this treatment period, it is likely the paradigm had some effect. Although much of our later data are negative, it does not seem to be rigorous to lump the LBN and standard-reared animals as if nothing had been done during the early treatment period. It is possible examination of a different set of variables would reveal early-life treatment differences.

Other points:

The authors nicely point out that the effect of early-stress on the velocity of development including puberty is complex. As they point out, litter size governs the rate of growth, and lack of careful control might explain the difference between Kevin Bath's and other groups. [Author response] We thank the reviewer for this observation. A reduced level of stress, and/or a more rapid adaptation in the LBN dams of the CBA strain may underlie the lack of persistent difference in body mass in the offspring. Because our primary interest was reproductive effects and because body mass is a documented 'stress-independent' modulator of puberty, we limited the litter sizes to a range of 5-8 pups. This normalization may contribute to a paucity of phenotypes in the offspring in the present study.

In the revamped discussion, it would be helpful to discuss the origin and principles of the LBN paradigm and how to ascertain its effectiveness [Author response] Based on the reviewer's helpful suggestions, we have modified the manuscript in 3 ways. 1) added direct comparison of pup mass on PND11 as done in prior studies to the results; 2) in the results, we have added emphasis to the lack of corticosterone difference perhaps indicating a less severe (although based on behavior arguably existent) stress. 3) providing a discussion of the caveats mentioned above in the discussion, in particular with regard to strain and ascertaining effectiveness of this paradigm.

Reviewer 2 comments:

The authors presented a well-supported manuscript with clear and punctual hypotheses addressed with well-designed experiments, rigorous statistical analysis, and focused discussion.

Under the premise that early stress, induced by limited nest material along seven postnatal days and acute stress during adulthood, alters the development of sexual characters and periovulatory luteinizing hormone surge. Also, the authors evaluate the effect of both stressors on GABAergic neurons that modulate the GnRH release. The authors describe the no effects of both stressors on several variables that are indexes of sexual external characteristics. These results are a relevant contribution to future studies using this approximation and for constructing animal models to evaluate the effect of early stress on reproduction.

The methods are well described, and the results are well presented and described. One strength of the manuscript is that the discussion is centered on present results and offers pertinent limitations.

A few issues require attention before it is published.

1. The introduction section lacks information about how stress affects the activity of GABAergic neurons and, consequently, this impact on GnRH release. [Author response] Requested information on how the activity of GABAergic neurons could be affected by stress and the postulated consequences of this on GnRH release have been added.

2. In relation to the administration of corticosterone to verify the effect of this hormone on sexual characters, authors must justify the dose used. [Author response] Thank you for pointing out this omission. This dose was chosen based on in-progress studies in females in other work in the lab. The dose that mimicked the corticosterone rise induced by the ALPS paradigm in females was tested in males for the present work and produced serum corticosterone levels that were similar to the ALPS paradigm. We have added this to the results and referenced the abstract in which the female data have been presented.
