# Supplementary material for: Early-Life Resource Scarcity in Mice Does Not Alter Adult Corticosterone or Preovulatory Luteinizing Hormone Surge Responses to Acute Psychosocial Stress
Source: eNeuro. 2024 Jul 26;11(7):ENEURO.0125-24.2024. doi: 10.1523/ENEURO.0125-24.2024 (PMC11287788; doi:10.1523/ENEURO.0125-24.2024)
Supplement: Extended Data — Zip file of custom code for PSC detection and analysis, ffmpeg recording of dam behavior, and R analysis. Download Extended Data, ZIP file. [file eneuro-11-ENEURO.0125-24.2024-s002.zip › PSC-analysis/AGG_VBWPanel/helpDocs/AGG_VBW_DataFolderOrg.docx]

Data Folder Functions

- create[x]df()
  - Create (with overwrite tag) the indicated data folder
  - This does not seem to consistently always delete and overwrite an existing folder
  - Best to kill data folder if definitely need it to be cleared
- get[x]df()
  - Get the data folder reference for the indicated folder
  - This does not make or check that data folder exists
  - Returns DFREF
- kill[x]df()
  - First checks if data folder exists
  - If it does, it kills the folder

Folder structure

| Folder | Functions | Description |
| --- | --- | --- |
| SCIW | createTempSCTwavesDF()  getTempSCTwavesDF()  killTempSCTwavesDF() | Where imported SCT waves are stored temporarily  Folder is created by loadData function b/c the SCT waves from JPSmartConc are stored here |
| VBW | createVBWDF()  getVBWDF() | Holding folder for all other VBW analysis folders except for temp folder for import  Folder must be created befor sub create functions are called |
| SCTW | createSCTwavesDF()  getSCTwavesDF() | Location where SCT waves are copied  Official reference location of waves for burst detection |
| Detect | createVBWdetectionDF()  getVBWdetectionDF() | Each cell burst detection waves are stored in this folder |
| C[cellName] | createVBWcellDetectionDF(cellName)  getVBWcellDetectionDF(cellName)  *_byFolderName(cellFolderName)   - intervals - gSw (gap starts wave) - gSwPlusE (gap starts wave plus end) - gEw (gap ends waves) - gEwPlusS (gap ends wave plus start) - intervalGapFlag - backIntervalsToGaps - forwardIntervalsToGaps - duration - gapDuration - recDuration - cellName | The folder containing the detection folders for each individual cell  Folder name prepends “c” to cell name (folders with # start can be difficult to reference)  These waves are run just once per cell |
| bw[#] | createVBWcellDetectionBWDF(cellName, bw)  getVBWcellDetectionBWDF(cellName, bw)   - burstDurations - burstFlags – near gaps - burstPlotX - burstPlotY - burstPoints - burstSpikes - burstSpikeTimes - burstStartPoints - burstStartTimes - interTimes - inter_Intervals - intraInt_burstStart - intra_intervals - spikeInfo - ssFlags - ssPoints - ssTimes - [cellName]_bw_[#]_bds - [cellName]_bw_[#]_bpx - [cellName]_bw_[#]_bpy - nBursts - nSS - numInterIntervals - numIntraIntervals | Folder containing the detection waves at a given burst window for each cell  BW is given as a variable – the actual burst window – and then the function prepends “bw”  These waves are run for each bw, but the referenced for specific analyses |
| ansys | createVBWanalysisDF()  getVBWanalysisDF() | Folder containing the specific analysis folders |
| [analysisName] | createVBWSpecAnalysisDF(analysisName)  getVBWSpecAnalysisDF(analysisName)   - useRecDur | Folder containing the individual cell analyses & some group info |
| c[CellName] | createVBWcellAnalysisDF(analysisName, cellName)  getVBWcellAnalysisDF(analysisName, cellName)   - bf – burst frequency (Hz) - bFlags – burst # near gaps - bn – burst number - bww – burst window wave - choppedSCT – chopped SCT wave for region - firingHisto – histogram for plotting - gapPlotX – x wave for plotting gaps - gapPlotY – y wave for plotting gaps - inter – interevent intervals - intra – intraburst intervals - matchingNamesIndex - mbd – mean burst duration - spb – spikes per burst - ssf – single spike frequency (Hz) - ssFlags – single spike flags - # near gaps - ssn – single spike number - tf – total frequency (Hz) - duration – total duration of region - gapDur – gaps during region - maxBW – burst window with max # bursts - recDur – duration of region minus gaps - regionDur – total duration of region - cellName – cell name | Folder containing the waves for the completed burst analysis for each cell. These waves move across each burst window |
| maxBW | createVBWAnalysisMaxBWDF(analysisName)  getVBWAnalysisMaxBWDF(analysisName) | Folder for each analysis for calculating group maxBWs |
| byAvgBF | createVBWMaxBWbyAvgBFDF(analysisName)  getVBWMaxBWbyAvgBFDF(analysisName)   - avgBF_group# – averaged burst frequency wave for each group - maxBW_forAvgBF_group# – bw at which peak of avgBF_group# occurs | Subfolder for calc by the peak of the average burst frequency of each group |
| byCellMax | createVBWMaxBWbyCellMaxDF(analysisName)  getVBWMaxBWbyCellMaxDF(analysisName)   - maxBWs_group# – wave of the maxBW for each cell in a group - avgMaxBW_group# – average of maxBWs_group# wave | Subfolder by the average peak bw of each cell in a group |
| panel | createVBWPanelDF()  getVBWPanelDF() | Folder for general panel variables and output |
| cellOut | createVBWIndivCellOutDF()  getVBWIndivCellOutDF()   - bf - bFalgs - bn - bww - firingHisto - gapPlotX - gapPlotY - inter - intra - mbd - spb - ssf - ssFlags - ssn - tf - gapAxis   Waves get replaced with values from selected analysis/cell | Folder for waves related to creating the individual cell table and histogram |
| outByBW | createVBWallCellsOutDF()  getVBWallCellsOutDF()   - bf - bFlags - bn - cellName - inter - intra - mbd - spb - ssf - ssFlags - ssn - tf   These waves are replaced with the value for each cell for the selected analysis and burst window  For graphs, a _group# split of active wave is created, copied into the multCell_group# wave and then copied from there into the multCellBOut multi-dimensional wave for plotting | Folder for waves related to creating the output table for all cells at a specific burst window and making the grouped plots |
| maxBWplot | createVBWMaxBWPlotDF()  getVBWMaxBWPlotDF()   - avgBF_group# - one for each group. Replaced by values from selected analysis/group | Folder for waves related to creating the grouped max BW plots |
