# Supplementary material for: Early-Life Resource Scarcity in Mice Does Not Alter Adult Corticosterone or Preovulatory Luteinizing Hormone Surge Responses to Acute Psychosocial Stress
Source: eNeuro. 2024 Jul 26;11(7):ENEURO.0125-24.2024. doi: 10.1523/ENEURO.0125-24.2024 (PMC11287788; doi:10.1523/ENEURO.0125-24.2024)
Supplement: Extended Data — Zip file of custom code for PSC detection and analysis, ffmpeg recording of dam behavior, and R analysis. Download Extended Data, ZIP file. [file eneuro-11-ENEURO.0125-24.2024-s002.zip › PSC-analysis/AGG_VBWPanel/helpDocs/Burst Analysis flow Chart.pdf]

Burst Analysis Flow Chart

Saturday, February 6, 2021 7:49 PM

From Smart Conc

→ Get a wave for the recording
Notes include gap info

O Whole recording O Region Table

Make vBW Graph

if whole → vBWButtonProc

neoVBWButtonProc

if regions → VBWButtonProc2

vBWButtonProc
↓ processing
vbw_analysis

force_name = forza
wn = sctwn

(sctwn generated here → neoVBWButtonProc)

String sctwn = StringbyKey("sct", B_Struct.userdata)

VBWButtonProc2
↓ processing
region_vbw_analysis
Use region info waves
→ name, start, end
Split spikes into different waves depending on relationship to region times

for each region run →

ts_vbanalysis(wn, vbwmin, vbwmax, vbwint, force_name)

after → City plots

after → city plots
↓ processing

Get # of burst windows to analyze
Create waves for each output parameter
and use shortwn (force name) to name

For each burst window
bursts = backwardsBanalysis(wn, bw, force_name, usegaps)

This is where spikes are tagged as single spikes/bursts, and intervals are collected.
This recreates the backwards interval list along with gap tags for each iteration

If a spike occurs w/in burst window of start/end/gap, it is flagged but included.
If interval is affected by start/gap, it's not included

output:

- bpx: , bpy: , bds: City plot
- bn: Burst #
- mbd: Mean burst duration
- spb: Spikes/burst
- bf: Burst freq
- ssn: Single spike #
- ssf: Single spike frequency
- tf: total frequency
- mInter: mean interevent interval

mIntra: mean intraburst interval
bdoubts: # of bursts flagged w/ doubts
ssdoubts: # single spikes flagged w/ doubts

VBW_OP (wn, bursts) - uncertain if necessary w/ current iteration)

Add this burst info to output waves for this burst window

Make VBW Tables

makeVBWTables Proc

If VBW_enabled
The waves already exist, need to show them in a table
↳ MakeVBWTables

analysis_types
bn; mbd; spb; bf; ssn; ssf; tf; inter; intra; bdoubts; ssdoubts
Will look for these waves belonging in datafolder
if RegionVBWEnabled
# of regions = # of names counted
else
# of regions = 1

output to VBWtable0

for each region
For first iteration, add burst window wave

Append each parameter wave to the table
