# Supplementary material for: Early-Life Resource Scarcity in Mice Does Not Alter Adult Corticosterone or Preovulatory Luteinizing Hormone Surge Responses to Acute Psychosocial Stress
Source: eNeuro. 2024 Jul 26;11(7):ENEURO.0125-24.2024. doi: 10.1523/ENEURO.0125-24.2024 (PMC11287788; doi:10.1523/ENEURO.0125-24.2024)
Supplement: Extended Data — Zip file of custom code for PSC detection and analysis, ffmpeg recording of dam behavior, and R analysis. Download Extended Data, ZIP file. [file eneuro-11-ENEURO.0125-24.2024-s002.zip › PSC-analysis/AGG_VBWPanel/helpDocs/User Guide to AGG_VBWPanel.docx]

Multiple Cell Extracellular Analysis

AGG_VBW Panel

For video tutorials demonstrating the use of this panel, view the series of videos available here: <https://youtube.com/playlist?list=PL6PGeCwLdZ2wB1NUBnqkVwi42MkKxmLY6>

- Be sure that you have the most up-to-date version of td-analysis from the server
- Before beginning with this panel, you should complete JPSmartConc concatenation of the entire recording for each cell in your dataset. It will be easiest to proceed if these pxp files with the concatenated waves are stored in the same folder, or a small series of folders
- To open the panel go to Macros → AGG_VBWPanel
  - This will open a new panel or bring the existing panel window to the front of your screen
- There are five tabs in this panel
  - Load Data
  - Burst Analysis
  - Individual Cells
  - Max BW
  - All Tbl Output
  - Graph Output
- To load your pxp files
  - Press the “**Select a pxp folder**” button
    - You can do this for multiple folders
  - You should see the temporary _sct (smart concatenated waves) from JPSmartConc loaded in the list box on the left-hand side of the screen
  - Select the specific waves that you wish to load into your experiment
    - “**Select All Waves**” will select all temporary waves. If any of these names match the waves you already have in your experiment, they will overwrite the current version
    - “**Select Unloaded Waves**” will select only waves that are not already in your experiment
    - You can use *shift + click* to individually select waves of interest in the list box
  - Press “**Add selected waves**” to add the selected waves to your experiment
    - This will delete *all* of the temporarily loaded waves after copying the selected waves
- To update the info table
  - Use the radio buttons to decide if you want to
    - **Update and add new waves** – will create a row in the table for all loaded SCT waves in your experiment
      - This will get the name of the SCT wave, generate a suggested cell name by removing the _sct appendix, and suggest a region 1 start & end time based on the full duration of the recording
    - **Only update existing** – will update the duration information for rows that are already in your table, but will not add new rows to the table
      - This is helpful if perhaps you only want to run the burst detection on a subset of your loaded waves (though this can also be accomplished later by using groups)
  - Press **Update SCT Table** once you have made your decision
- About the SCT table
  - Each cell should have the name of the SCT wave in the first column (*sctWaveName*) – this should end in _sct
  - Each cell should have a *cellName* – generally this should be the same as the sct wave, just with the _sct appendix removed
    - Ex. 20210528a
  - *groupName* is the treatment/experimental group that should be used for this cell for further analysis
  - *reg#_start* / *reg#_end* waves give the starting time and ending time (respectively) of the regions for analysis for each individual cell
    - In the **Burst Analysis** tab, you can select whether you want to enter these times in minutes or seconds – the default is in minutes
    - In the **Burst Analysis** tab, you will also be able to provide more specific names to these regions, such as “baseline,” “treatment,” and “washout”
  - *totalDur* is the total duration of the SCT wave and is calculated when updating the SCT table
  - The other waves have not been implemented into the code at this time (2021-05-28)
  - **Clean Table** button will remove table rows that do not have a loaded SCT wave
- To run burst detection
  - Burst detection only needs to be run once per cell – this will generate waves that are then used by the burst analysis procedures to conduct the analysis for each desired region of the recording
  - In the **Burst Analysis** tab, specify your desired starting burst window and maximum burst window in seconds. You should also provide your desired increment
    - Default is 0.01 – 1.00 s in 0.01 sec increments
  - Press **Detect Bursts**
    - Before you can detect bursts, the program is going to ask you to confirm that you have accepted your most recent changes to the table. Unlike Excel, if you click out of a table in Igor without either pressing an arrow key or the enter button after editing an entry, the old value is still what would be used for your analysis
    - Enter 1 to confirm, or 0 to go back and fix it (or press cancel)
  - This process will take several minutes, especially if you have many cells or many high-firing cells
- To run burst analysis
  - Burst analysis can be run repeatedly across your dataset. New analysis names will not replace old analyses, unless they are the same name
  - Check the box next to the region(s) that you wish to analyze for this run
  - Provide a more descriptive name for the region (spontaneous, baseline, treatment, washout, etc)
  - Decide if you wish to further subdivide the region into smaller bins
    - Example – 30 min bins
      - Imagine you have 3 cells, Cell A that was recorded for 50 min, Cell B for 60 min, and Cell C for 70 min
      - For the first 30 min bin (from 0-30), all of your cells were recorded for this entire duration, so they would each be analyzed for this bin
      - For the second 30 min bin (from 30-60), only cells B and C were recorded for the full duration of this bin, so only these two cells would be analyzed for this bin
      - None of the cells were recorded for the full duration of the bin from 60-90 min, meaning that analysis for this third bin would not proceed
    - Pay attention to the units of time for the bin size – this will match the units that you’ve selected for the table entry
    - If you check the **use additional bins** box, all of the checked regions will be analyzed using these bins
      - Remember that you can **Analyze Bursts** multiple times, so if you only wish to use a particular bin size for one region, you can just check that specific region
  - Use the radio buttons to decide which type of duration you’d like to use for frequency calculations
    - **Recording dur** – this removes any gaps between series and just uses the time that data was actively being recorded
    - **Total dur** – this includes the gap times between series, providing the duration in “clock time” from the start to end of a region
  - Press **Analyze Bursts**
    - Remember to accept your most recent changes in the table before pressing this button!
    - This will also take several minutes to process. Just be patient (and use the time to get up and stretch!)
  - If the cell groups change after you complete your analysis (for example, perhaps you want to view the group results with a cell excluded from the group), press the **Update Groups** button
    - This will not re-run the full analysis, but it will regroup the cells based on the current state of the SCT table for the purposes of calculating the maximum burst window and graphing the output
      - Remember to accept your most recent changes in the table before pressing this button!
- To view the results for individual cells
  - In the **Individual Cells** tab, you can use the list boxes to choose a specific analysis and cell to inspect.
  - On this tab, you can view the table with the burst parameters for this cell at each burst window, and see a histogram of the firing rate (binned in 1-min intervals) for the cell during the selected analysis region
    - The histogram also displays the gaps in recording during the selected analysis region in blue lines
- To view the maximum burst window for each group
  - Go to the **Max BW** tab
  - There will be a graph displaying the output for the selected analysis
  - This graph plots the average burst frequency for all of the cells in a group at each burst window
  - On the right-hand side of the tab, you will see a list box displaying the different group names. If you change that selection, the output results for the maximum burst window will change accordingly
    - **By cell maximum** takes the average burst window for each cell in the group (that fired a burst) and averages these values
      - This tends to be skewed toward later burst windows, as a cell with 1000 bursts and 1 burst are equally weighted
    - **By average burst frequency** finds the peak of the burst frequency by burst window graph displayed on this tab for each group, and finds the associated burst window at which that peak occurs
      - This is more influenced by high burst frequency cells
    - *A note of caution –* though you cannot directly edit these values, you could accidentally click the arrow keys or scroll with your mouse. This would temporarily change the displayed value, but if you click back in the list box on the group of interest, it will reset the displays to the calculated values
  - You can use these peaks to help inform your decision of which burst window at which to analyze the parameters
- To view the burst parameters for all cells at a given burst window
  - Go to the **All Tbl Output** tab
  - Select the analysis of interest
  - Use the **Select BW** entry field, arrow keys/scroll bar, or slider to select a single burst window at which you want to get the burst parameter output
  - You can either copy/paste these values directly into a separate spreadsheet, or use the **Save Output Table** button to output the current table as a tab-delimited text file that can be opened by spreadsheet programs/analysis software
- To view graphs of the burst parameters at a given burst window
  - Go to the **Graph Output** tab
  - Select the region, burst window, and parameter of interest
  - Note that these graphs sometimes do wonky things on display – viewing them in Igor can give you a general impression of the data, but it’s worth loading them into a different program
  - If you want to try to reset the graphs, you can either close the panel and press “macro → AGG_VBWPanel” again (don’t worry – your data will be saved!) OR you can type updateBOutViolinPlot()into the command line
