# Supplementary material for: Early-Life Resource Scarcity in Mice Does Not Alter Adult Corticosterone or Preovulatory Luteinizing Hormone Surge Responses to Acute Psychosocial Stress
Source: eNeuro. 2024 Jul 26;11(7):ENEURO.0125-24.2024. doi: 10.1523/ENEURO.0125-24.2024 (PMC11287788; doi:10.1523/ENEURO.0125-24.2024)
Supplement: Extended Data — Zip file of custom code for PSC detection and analysis, ffmpeg recording of dam behavior, and R analysis. Download Extended Data, ZIP file. [file eneuro-11-ENEURO.0125-24.2024-s002.zip › PSC-analysis/AGG_VBWPanel/helpDocs/Vary Burst Window Analysis_AGG_2021-02-07.pptx]

## Slide 1
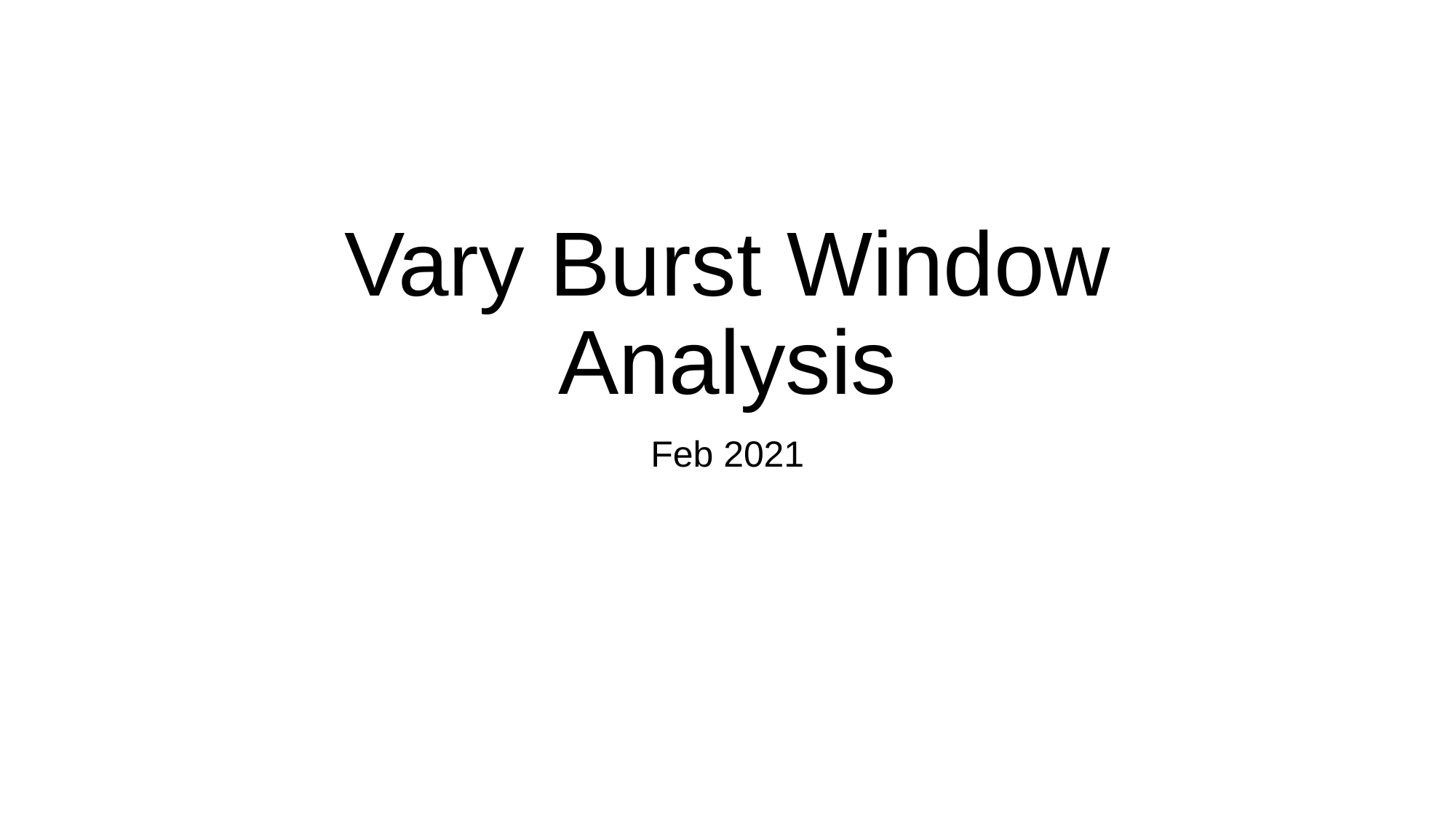

# Vary Burst Window Analysis
Feb 2021

## Slide 2
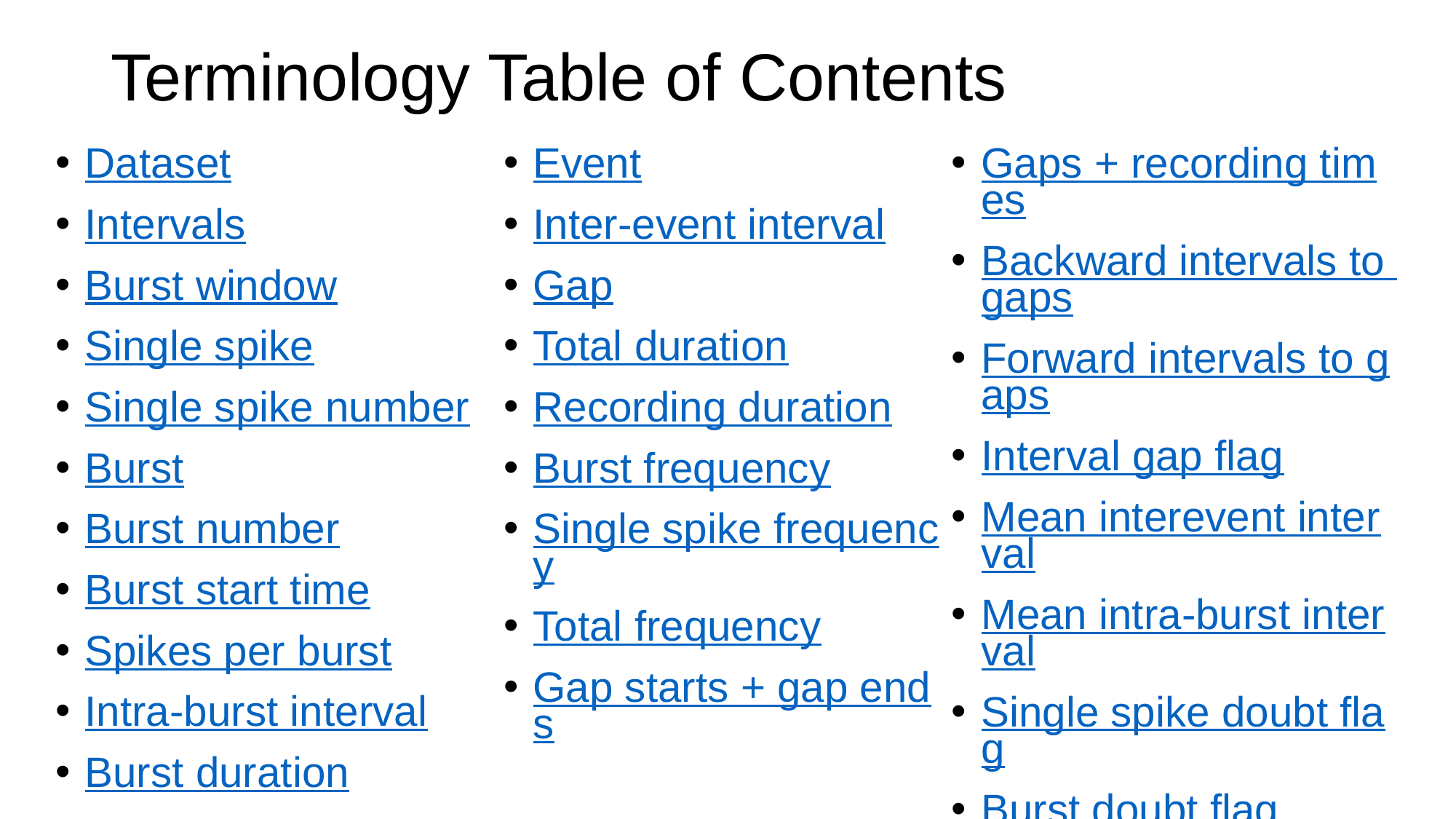

# Terminology Table of Contents
Dataset
Intervals
Burst window
Single spike
Single spike number
Burst
Burst number
Burst start time
Spikes per burst
Intra-burst interval
Burst duration
Event
Inter-event interval
Gap
Total duration
Recording duration
Burst frequency
Single spike frequency
Total frequency
Gap starts + gap ends
Gaps + recording times
Backward intervals to gaps
Forward intervals to gaps
Interval gap flag
Mean interevent interval
Mean intra-burst interval
Single spike doubt flag
Burst doubt flag
Regions
Current region processing
“Ideal” region processing

## Slide 3
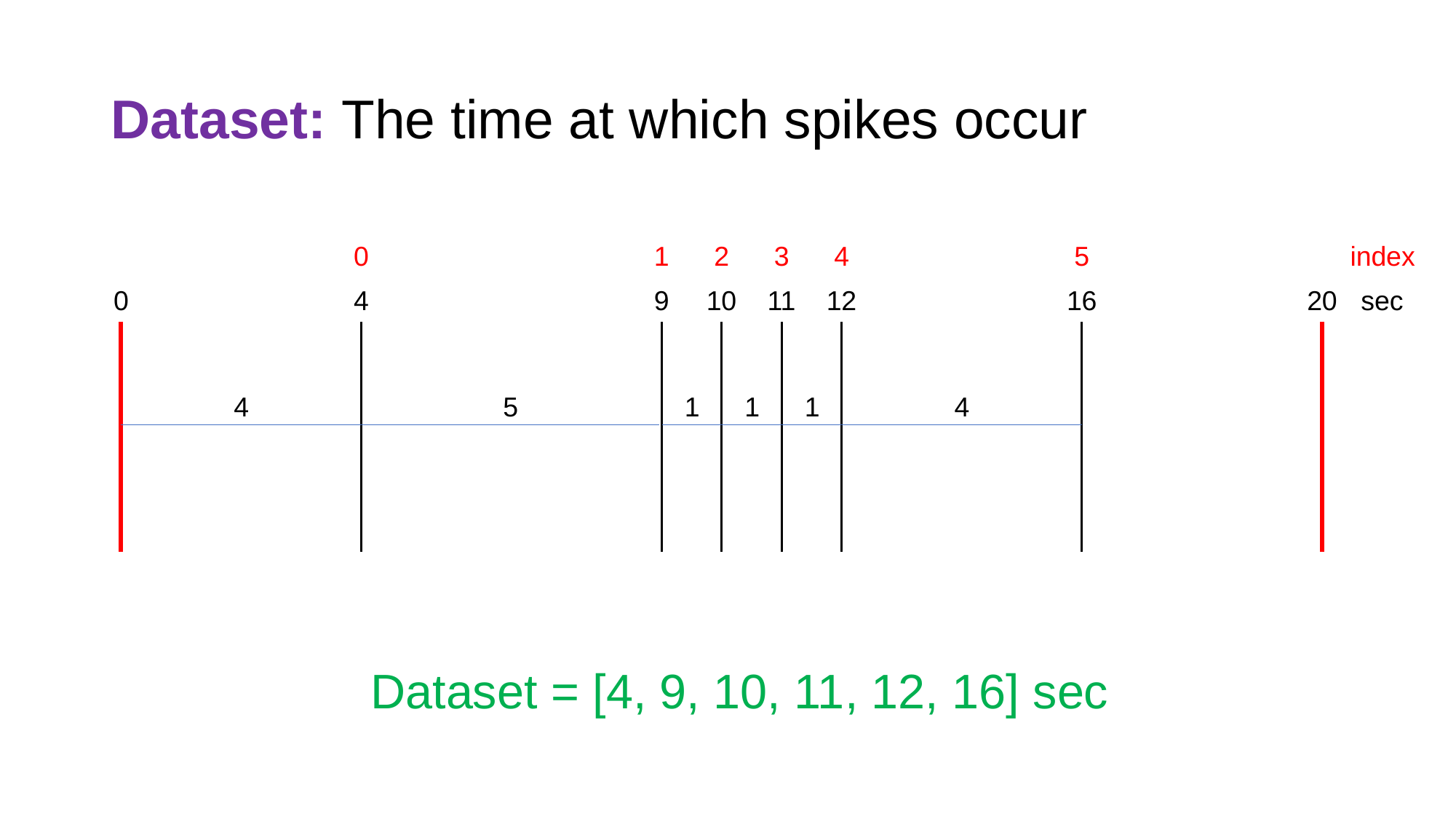

# Dataset: The time at which spikes occur
0
1
2
3
4
5
index
0
4
9
10
11
12
16
20
sec
4
5
1
1
1
4
Dataset = [4, 9, 10, 11, 12, 16] sec

## Slide 4
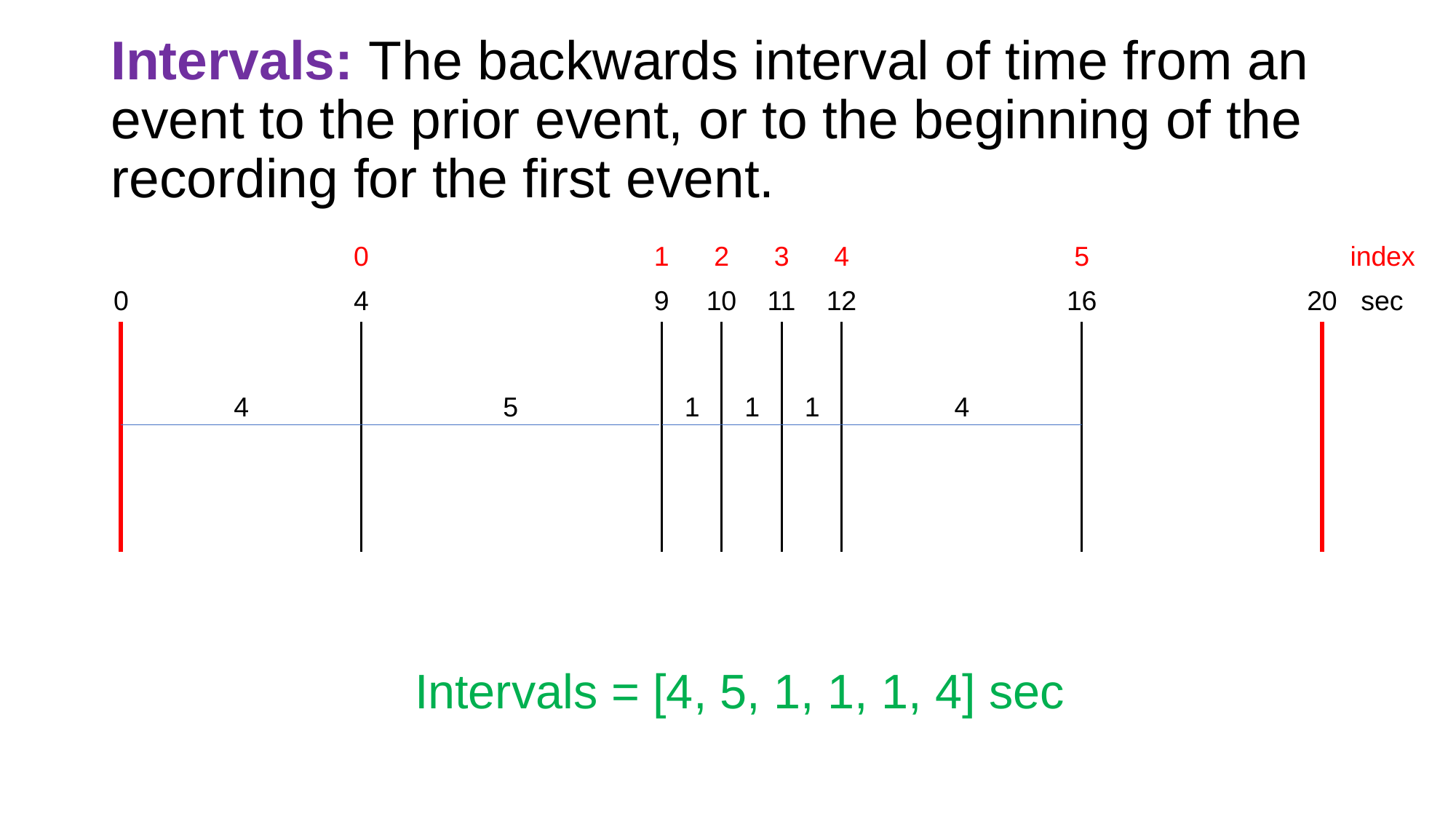

# Intervals: The backwards interval of time from an event to the prior event, or to the beginning of the recording for the first event.
0
1
2
3
4
5
index
0
4
9
10
11
12
16
20
sec
4
5
1
1
1
4
Intervals = [4, 5, 1, 1, 1, 4] sec

## Slide 5
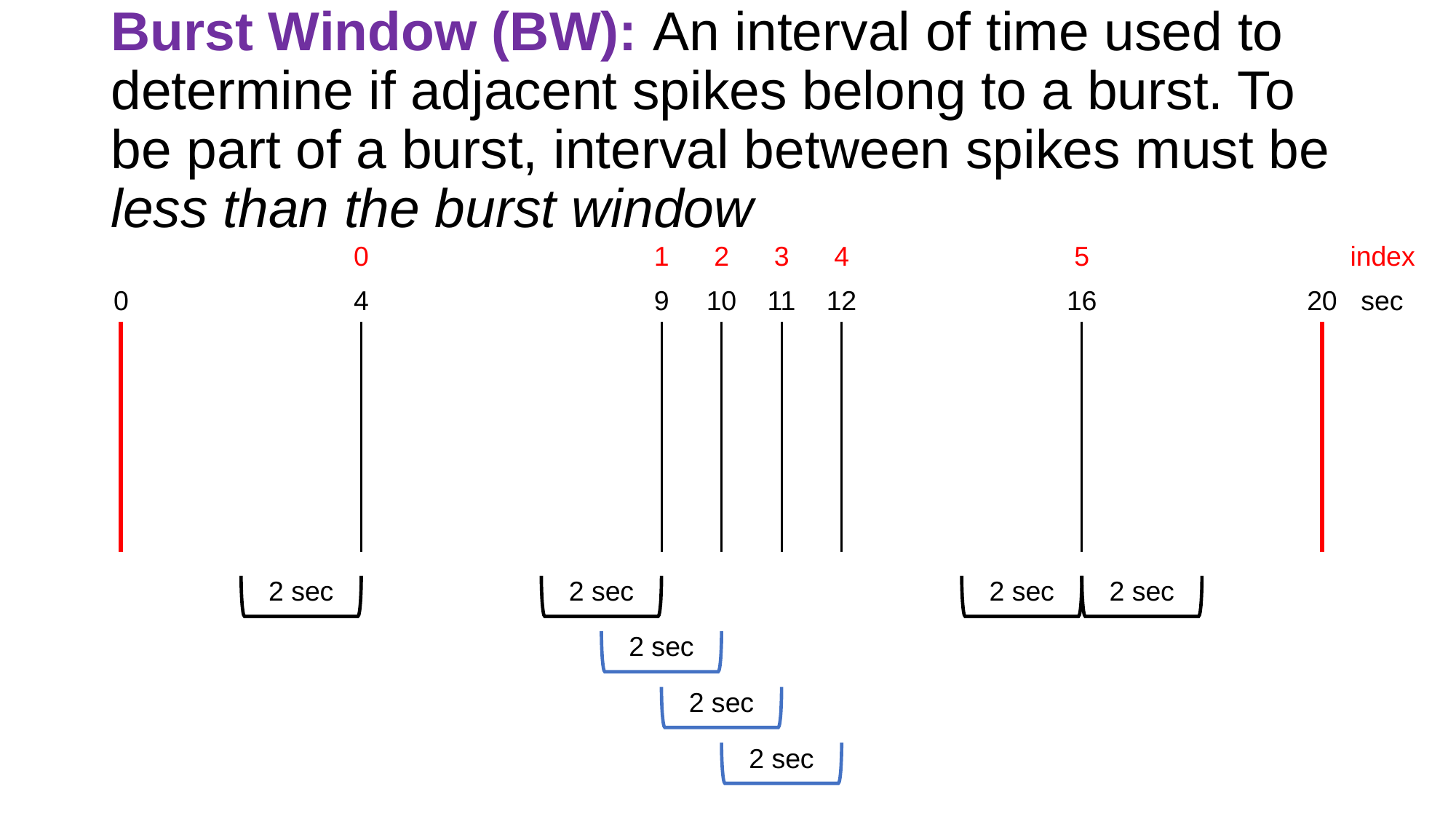

# Burst Window (BW): An interval of time used to determine if adjacent spikes belong to a burst. To be part of a burst, interval between spikes must be less than the burst window
0
1
2
3
4
5
index
0
4
9
10
11
12
16
20
sec
2 sec
2 sec
2 sec
2 sec
2 sec
2 sec
2 sec

## Slide 6
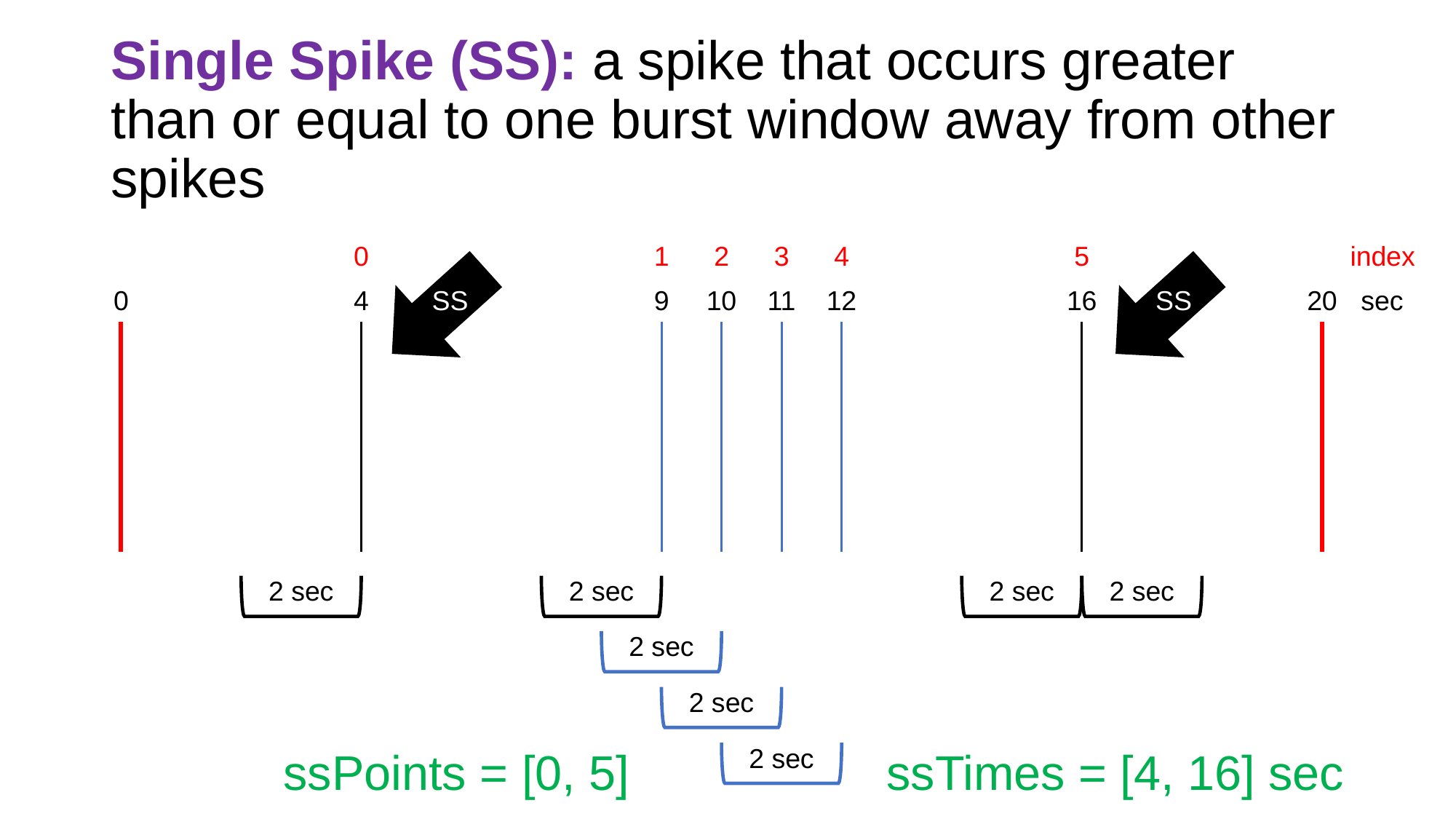

Single Spike (SS): a spike that occurs greater than or equal to one burst window away from other spikes
0
1
2
3
4
5
index
SS
SS
0
4
9
10
11
12
16
20
sec
2 sec
2 sec
2 sec
2 sec
2 sec
2 sec
2 sec
ssPoints = [0, 5]
ssTimes = [4, 16] sec

## Slide 7
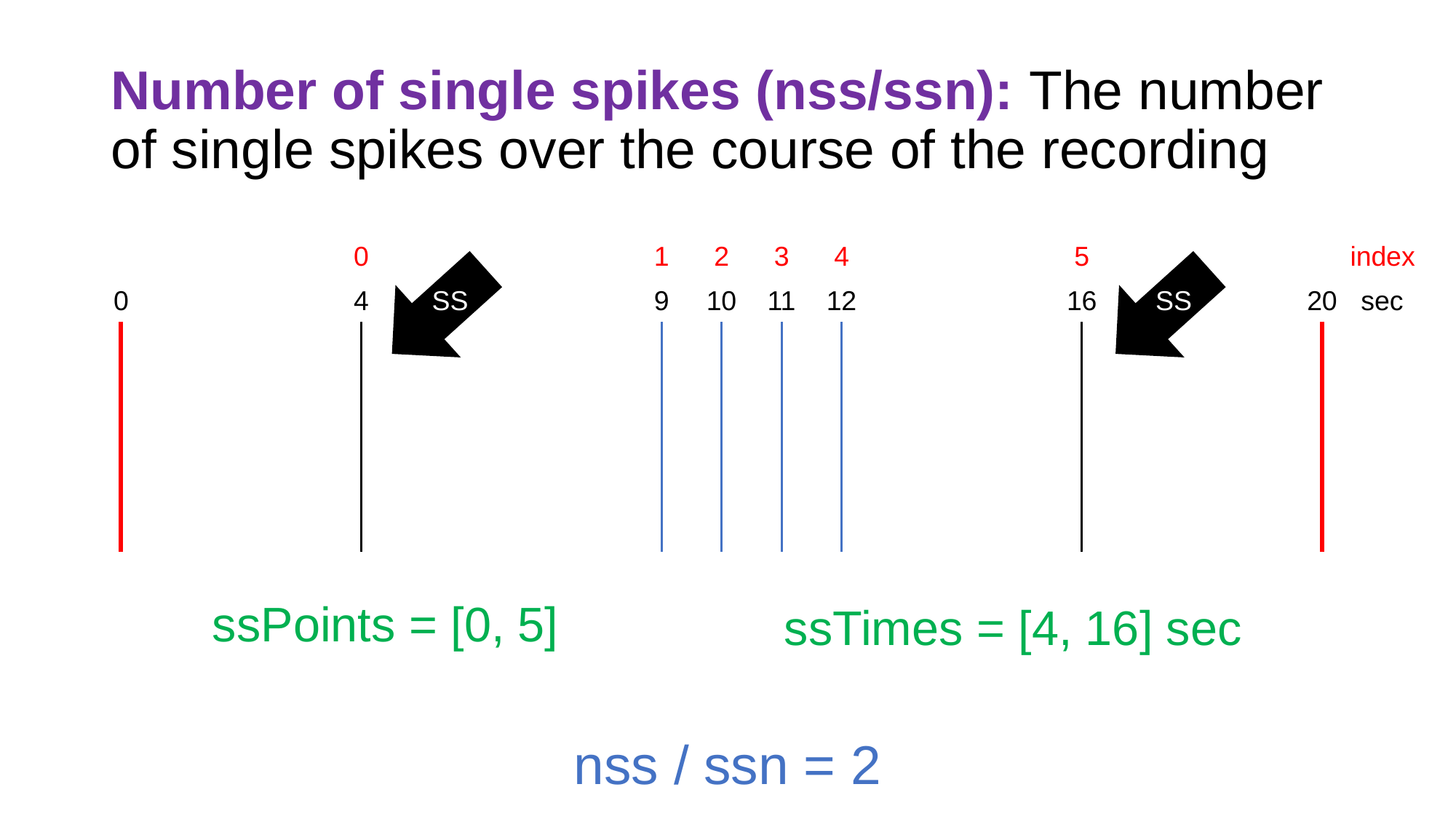

Number of single spikes (nss/ssn): The number of single spikes over the course of the recording
0
1
2
3
4
5
index
SS
SS
0
4
9
10
11
12
16
20
sec
ssPoints = [0, 5]
ssTimes = [4, 16] sec
nss / ssn = 2

## Slide 8
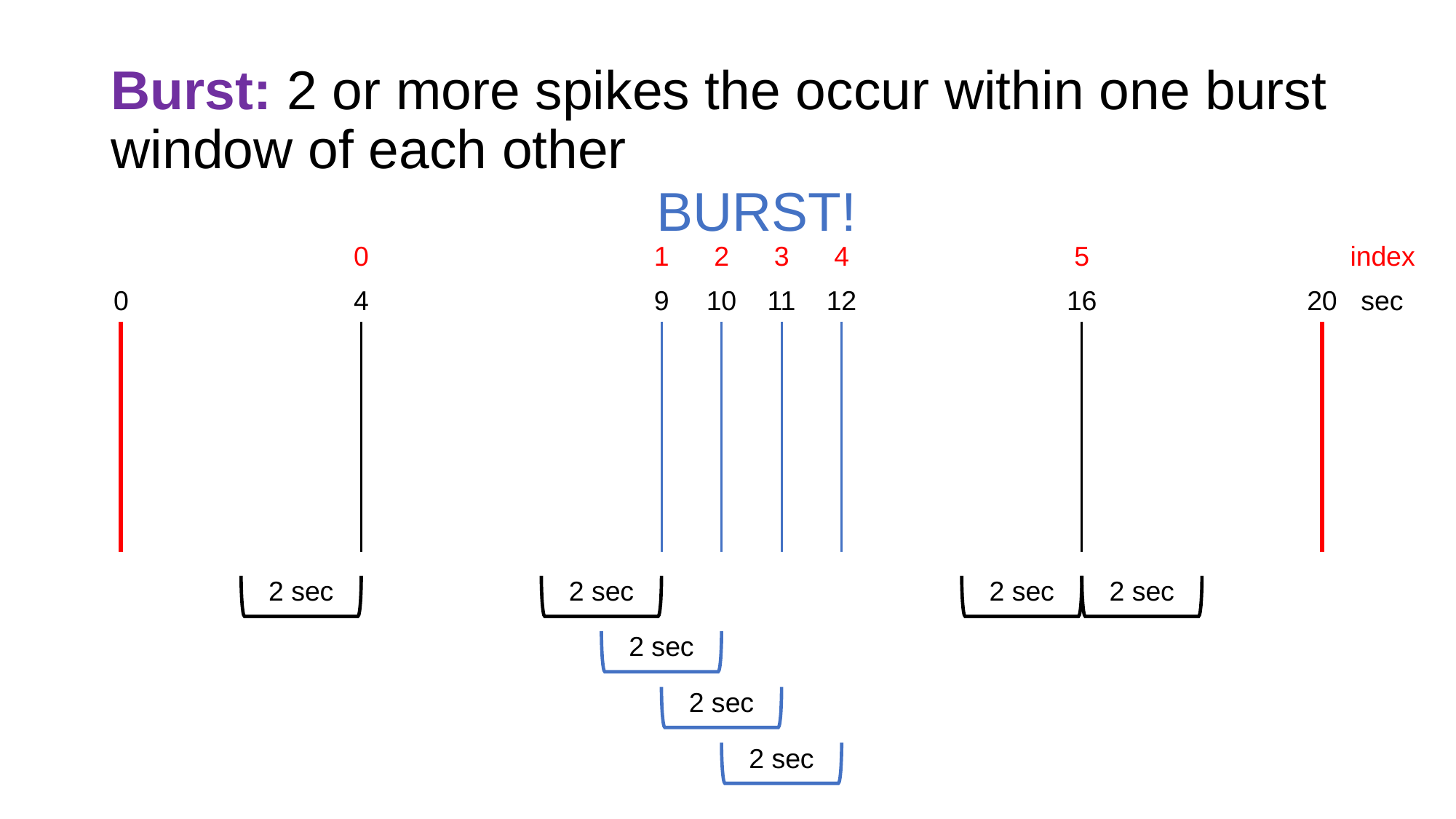

Burst: 2 or more spikes the occur within one burst window of each other
BURST!
0
1
2
3
4
5
index
0
4
9
10
11
12
16
20
sec
2 sec
2 sec
2 sec
2 sec
2 sec
2 sec
2 sec

## Slide 9
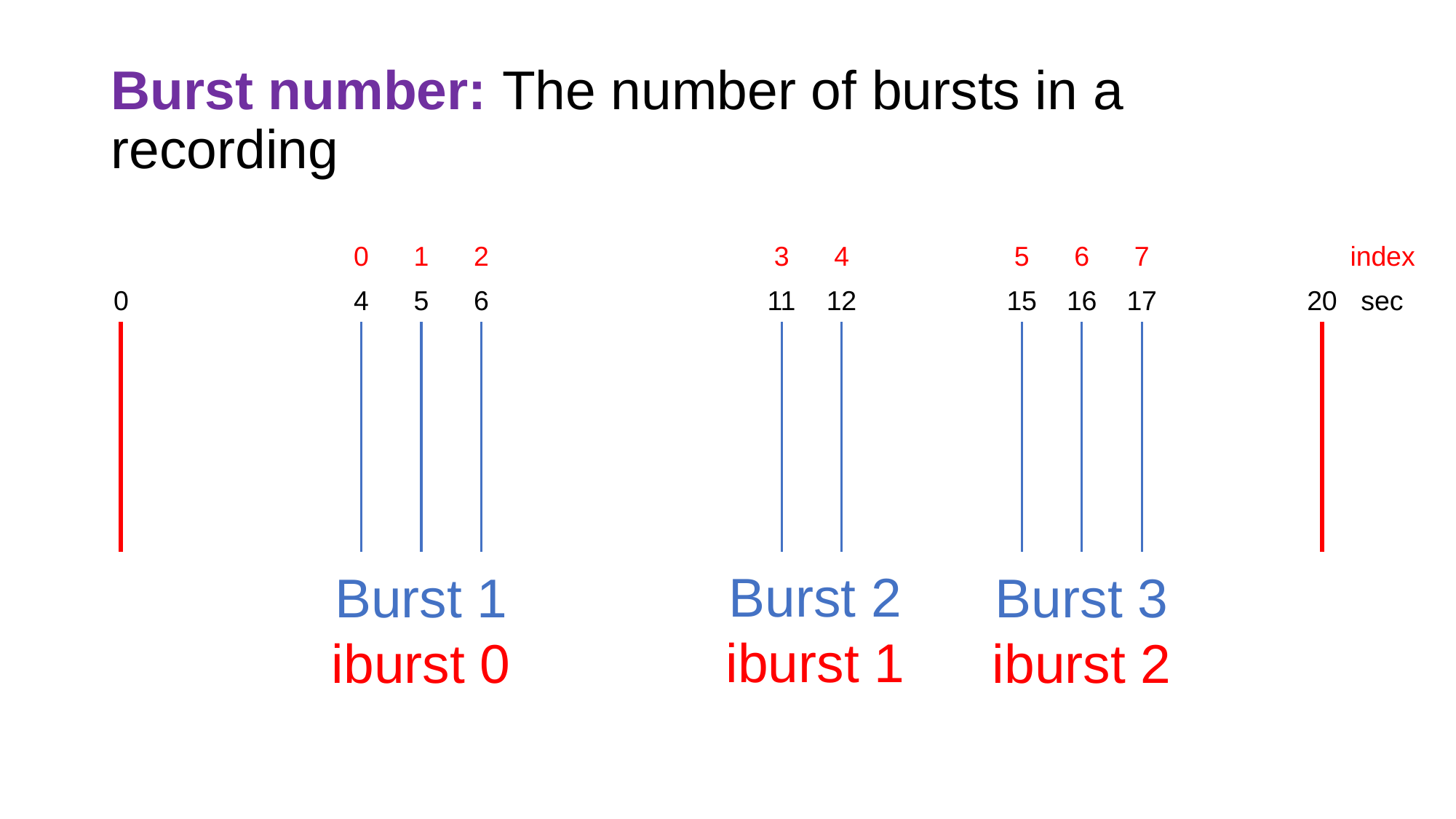

Burst number: The number of bursts in a recording
0
1
2
3
4
5
6
7
index
0
4
5
6
11
12
15
16
17
20
sec
Burst 2
iburst 1
Burst 1
iburst 0
Burst 3
iburst 2

## Slide 10
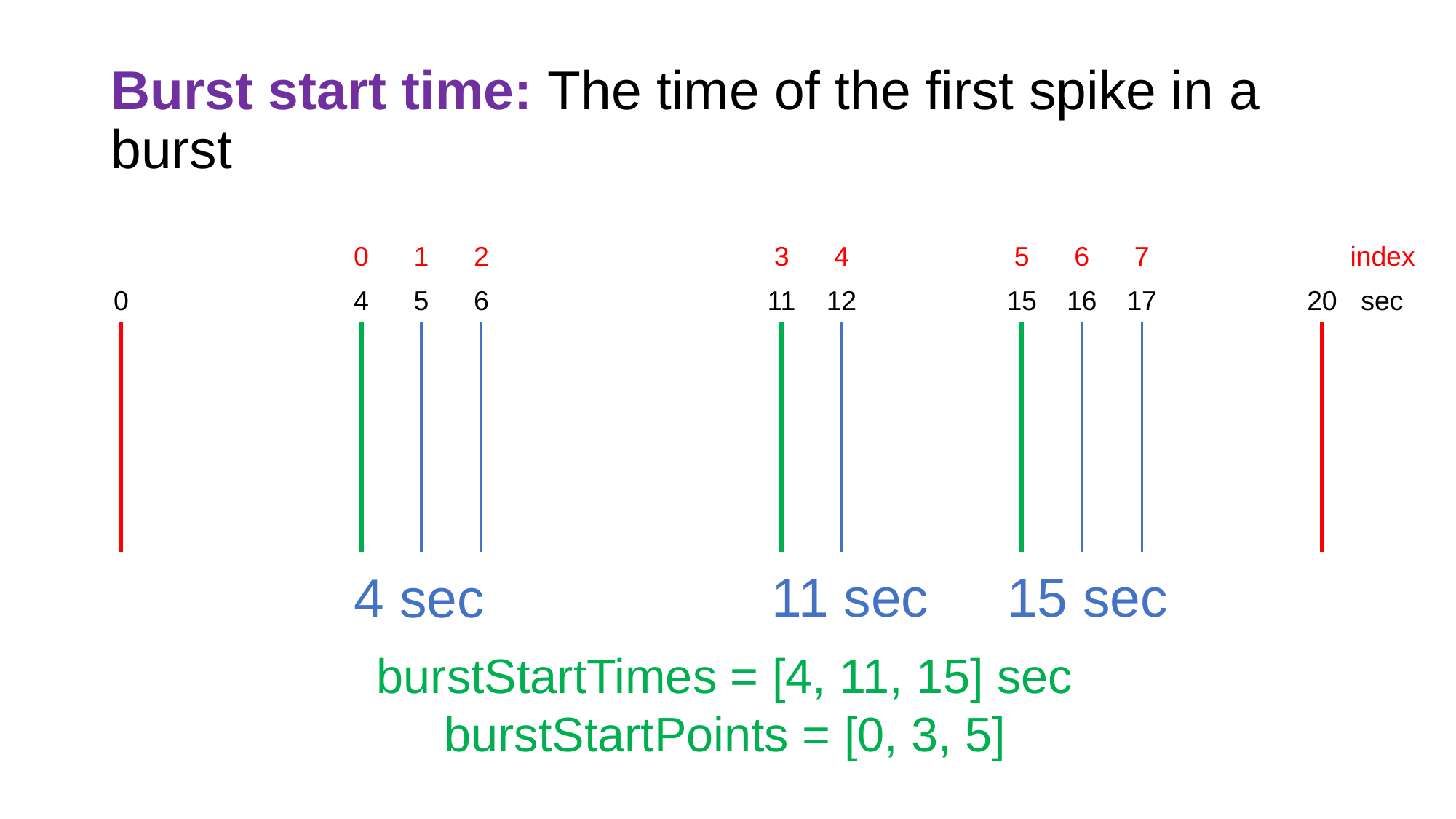

Burst start time: The time of the first spike in a burst
0
1
2
3
4
5
6
7
index
0
4
5
6
11
12
15
16
17
20
sec
11 sec
15 sec
4 sec
burstStartTimes = [4, 11, 15] sec
burstStartPoints = [0, 3, 5]

## Slide 11
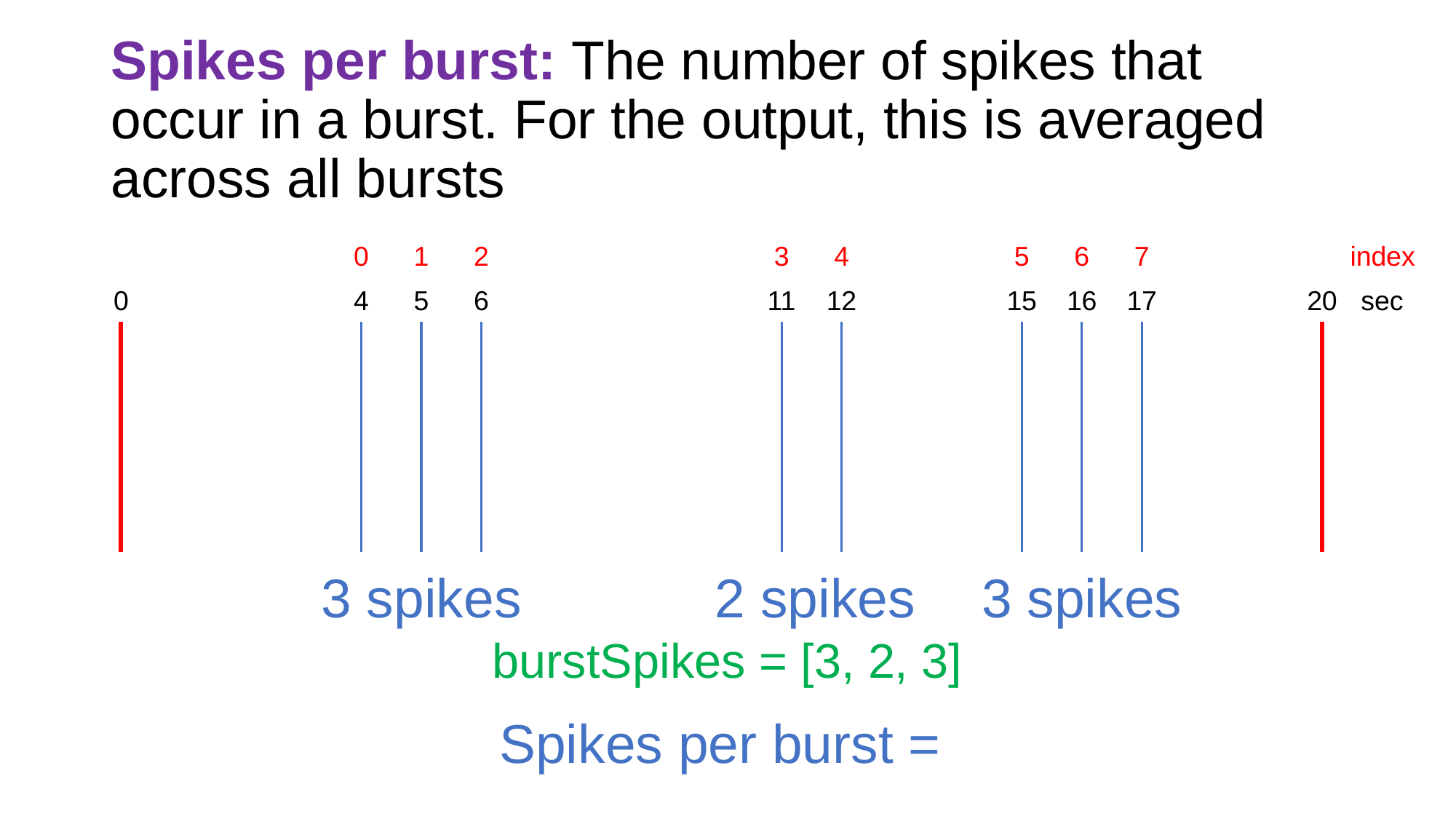

Spikes per burst: The number of spikes that occur in a burst. For the output, this is averaged across all bursts
0
1
2
3
4
5
6
7
index
0
4
5
6
11
12
15
16
17
20
sec
3 spikes
2 spikes
3 spikes
burstSpikes = [3, 2, 3]

## Slide 12
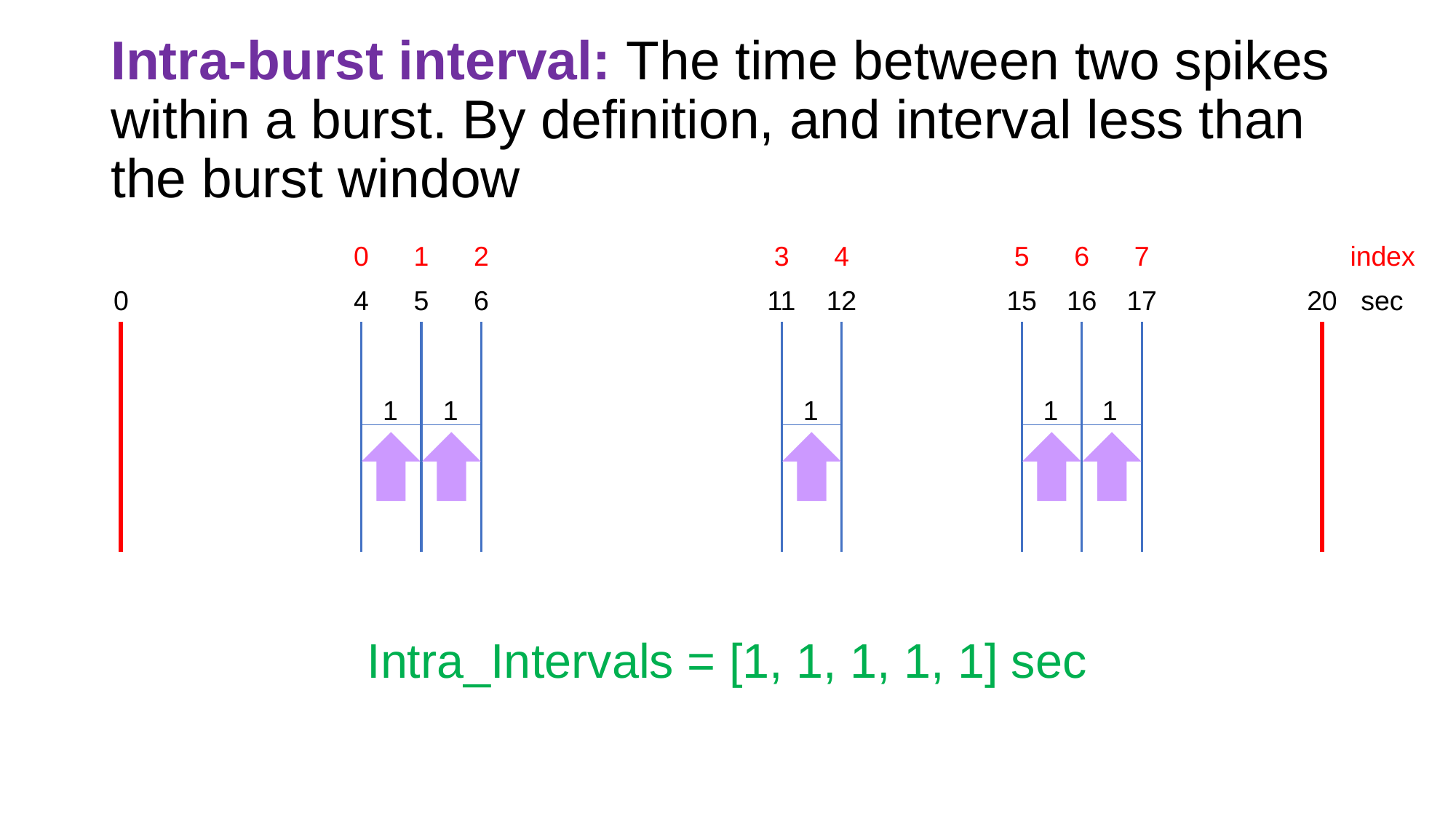

Intra-burst interval: The time between two spikes within a burst. By definition, and interval less than the burst window
0
1
2
3
4
5
6
7
index
0
4
5
6
11
12
15
16
17
20
sec
1
1
1
1
1
Intra_Intervals = [1, 1, 1, 1, 1] sec

## Slide 13
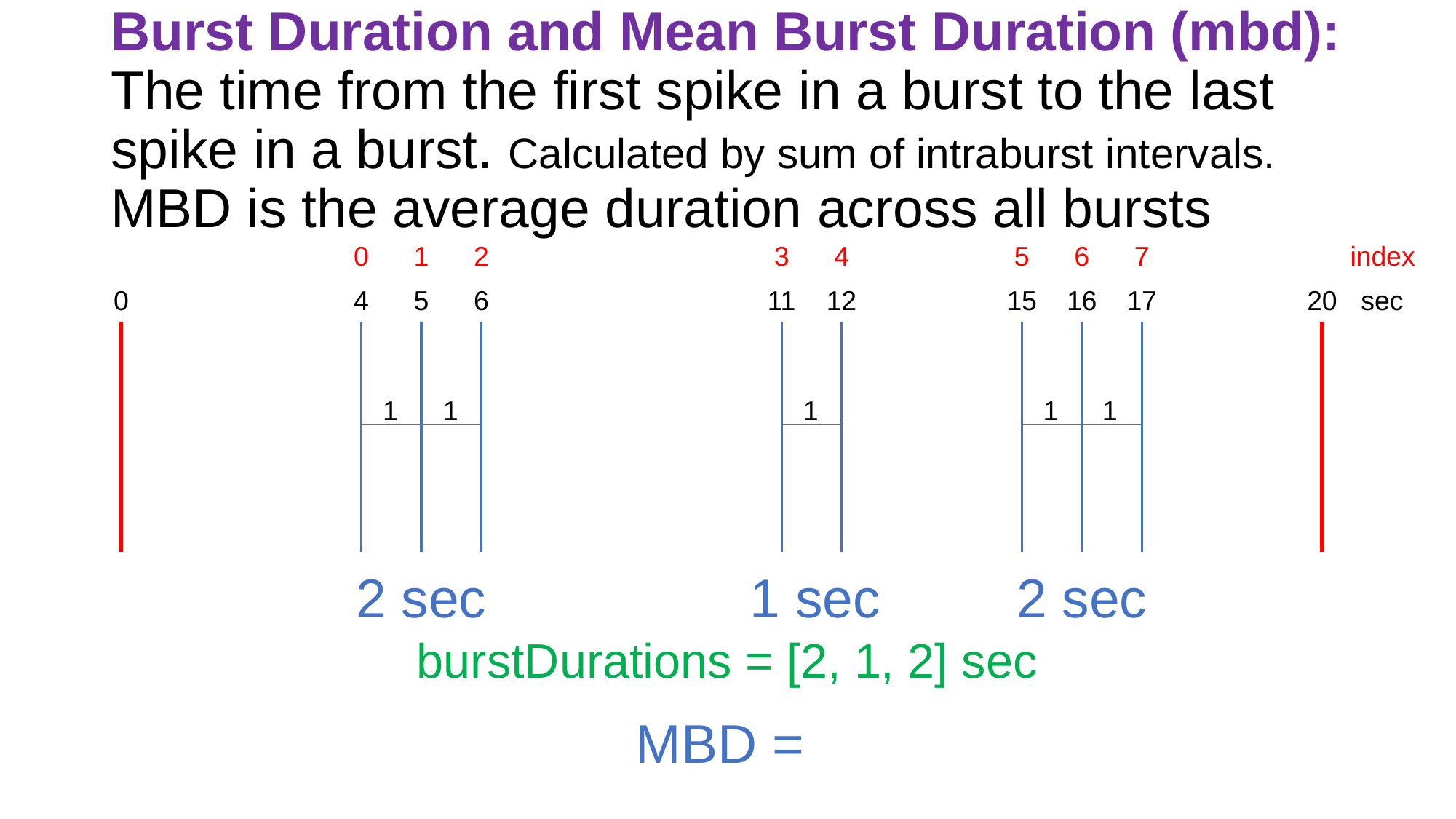

Burst Duration and Mean Burst Duration (mbd): The time from the first spike in a burst to the last spike in a burst. Calculated by sum of intraburst intervals. MBD is the average duration across all bursts
0
1
2
3
4
5
6
7
index
0
4
5
6
11
12
15
16
17
20
sec
1
1
1
1
1
2 sec
1 sec
2 sec
burstDurations = [2, 1, 2] sec

## Slide 14
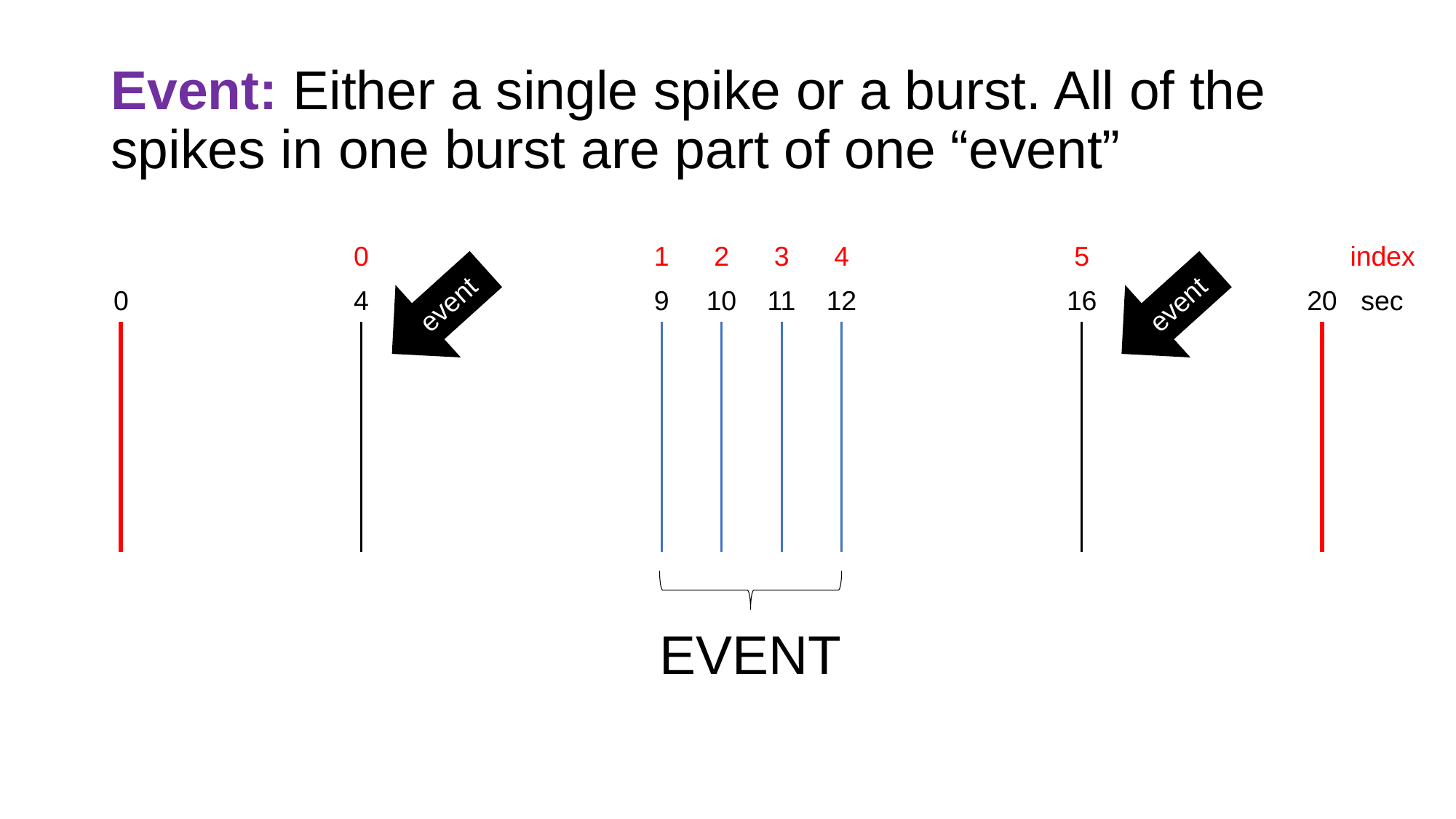

# Event: Either a single spike or a burst. All of the spikes in one burst are part of one “event”
0
1
2
3
4
5
index
event
event
0
4
9
10
11
12
16
20
sec
EVENT

## Slide 15
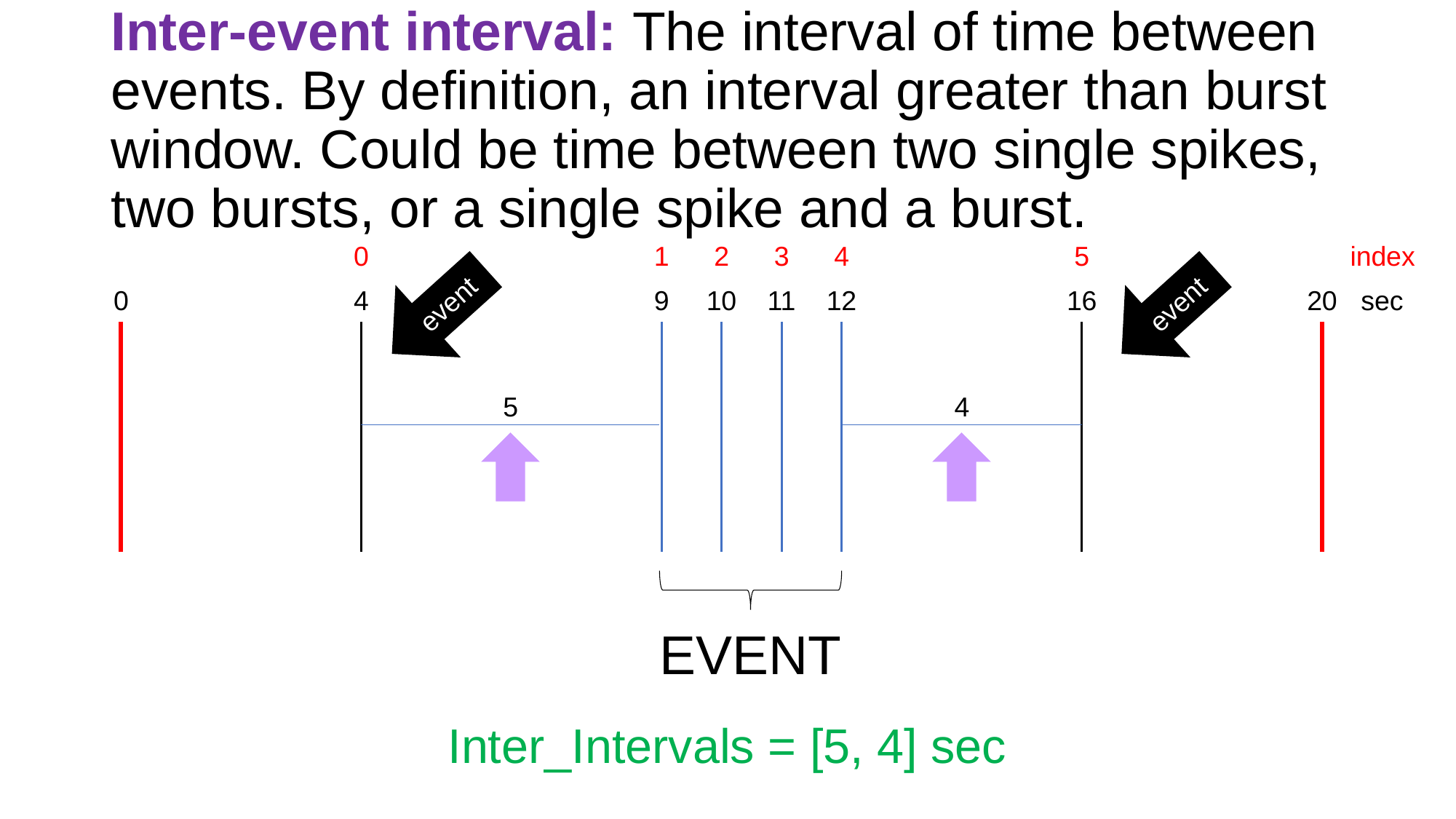

# Inter-event interval: The interval of time between events. By definition, an interval greater than burst window. Could be time between two single spikes, two bursts, or a single spike and a burst.
0
1
2
3
4
5
index
event
event
0
4
9
10
11
12
16
20
sec
5
4
EVENT
Inter_Intervals = [5, 4] sec

## Slide 16
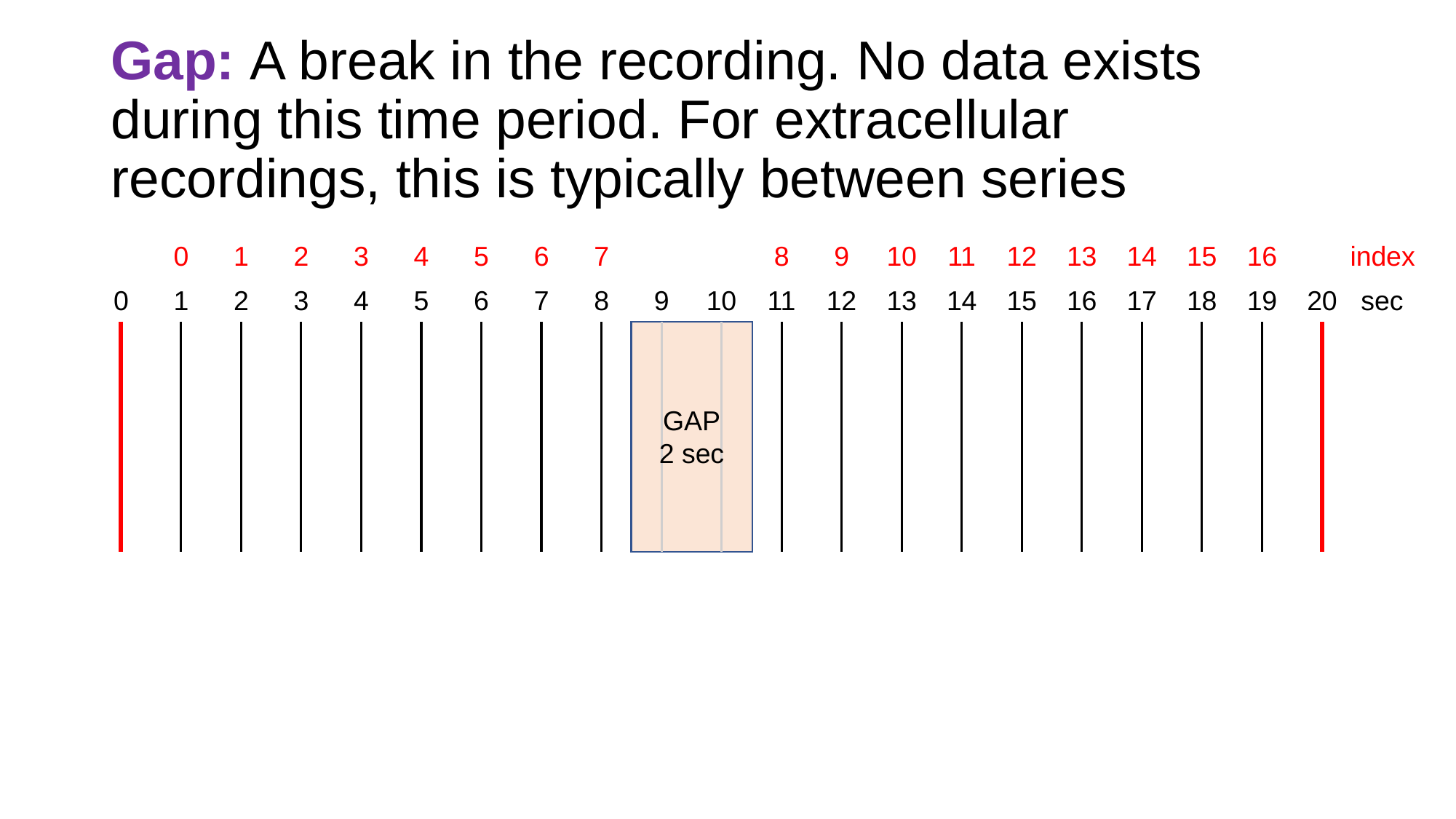

# Gap: A break in the recording. No data exists during this time period. For extracellular recordings, this is typically between series
0
1
2
3
4
5
6
7
8
9
10
11
12
13
14
15
16
index
0
1
2
3
4
5
6
7
8
9
10
11
12
13
14
15
16
17
18
19
20
sec
GAP
2 sec

## Slide 17
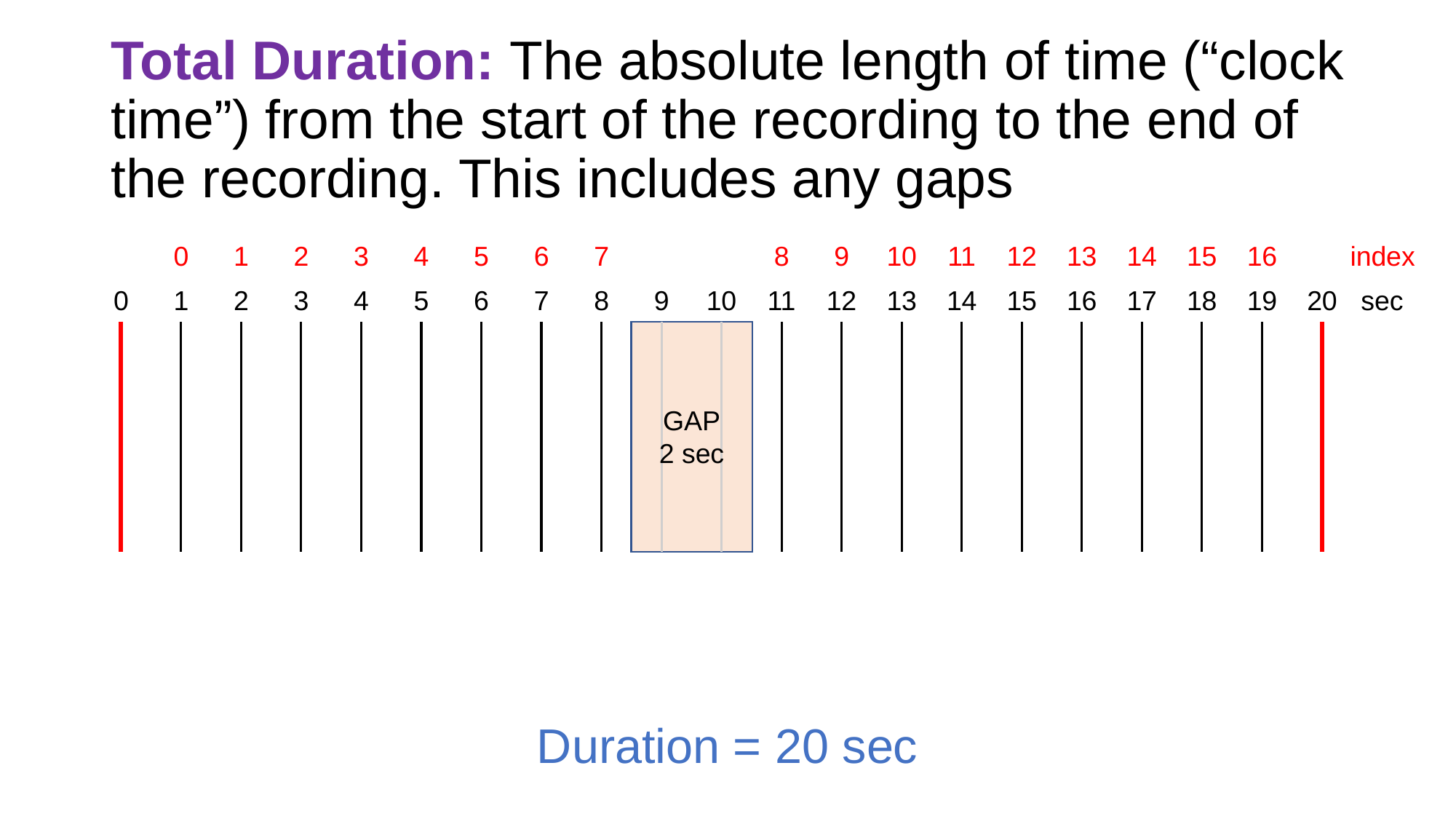

# Total Duration: The absolute length of time (“clock time”) from the start of the recording to the end of the recording. This includes any gaps
0
1
2
3
4
5
6
7
8
9
10
11
12
13
14
15
16
index
0
1
2
3
4
5
6
7
8
9
10
11
12
13
14
15
16
17
18
19
20
sec
GAP
2 sec
Duration = 20 sec

## Slide 18
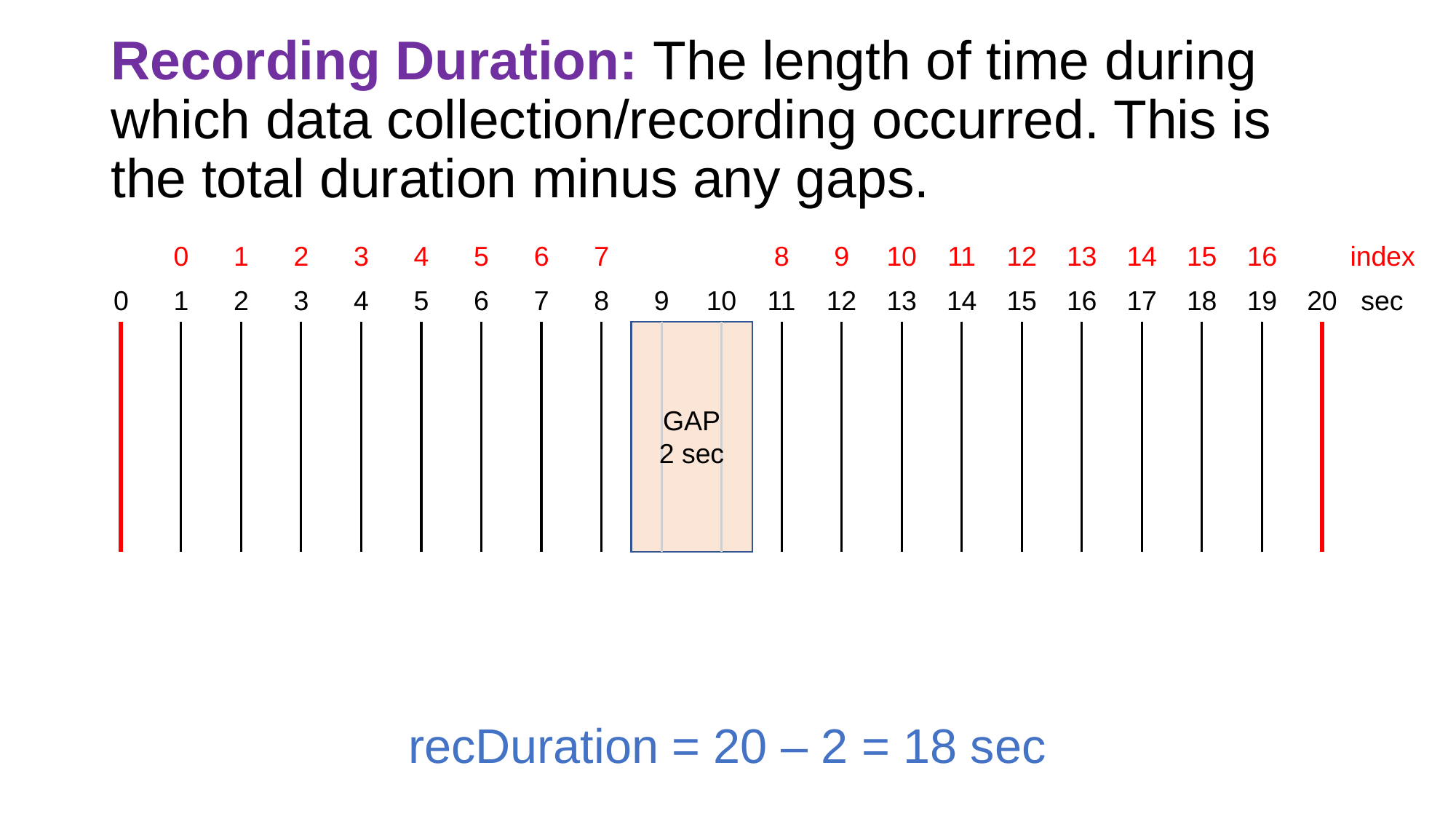

# Recording Duration: The length of time during which data collection/recording occurred. This is the total duration minus any gaps.
0
1
2
3
4
5
6
7
8
9
10
11
12
13
14
15
16
index
0
1
2
3
4
5
6
7
8
9
10
11
12
13
14
15
16
17
18
19
20
sec
GAP
2 sec
recDuration = 20 – 2 = 18 sec

## Slide 19
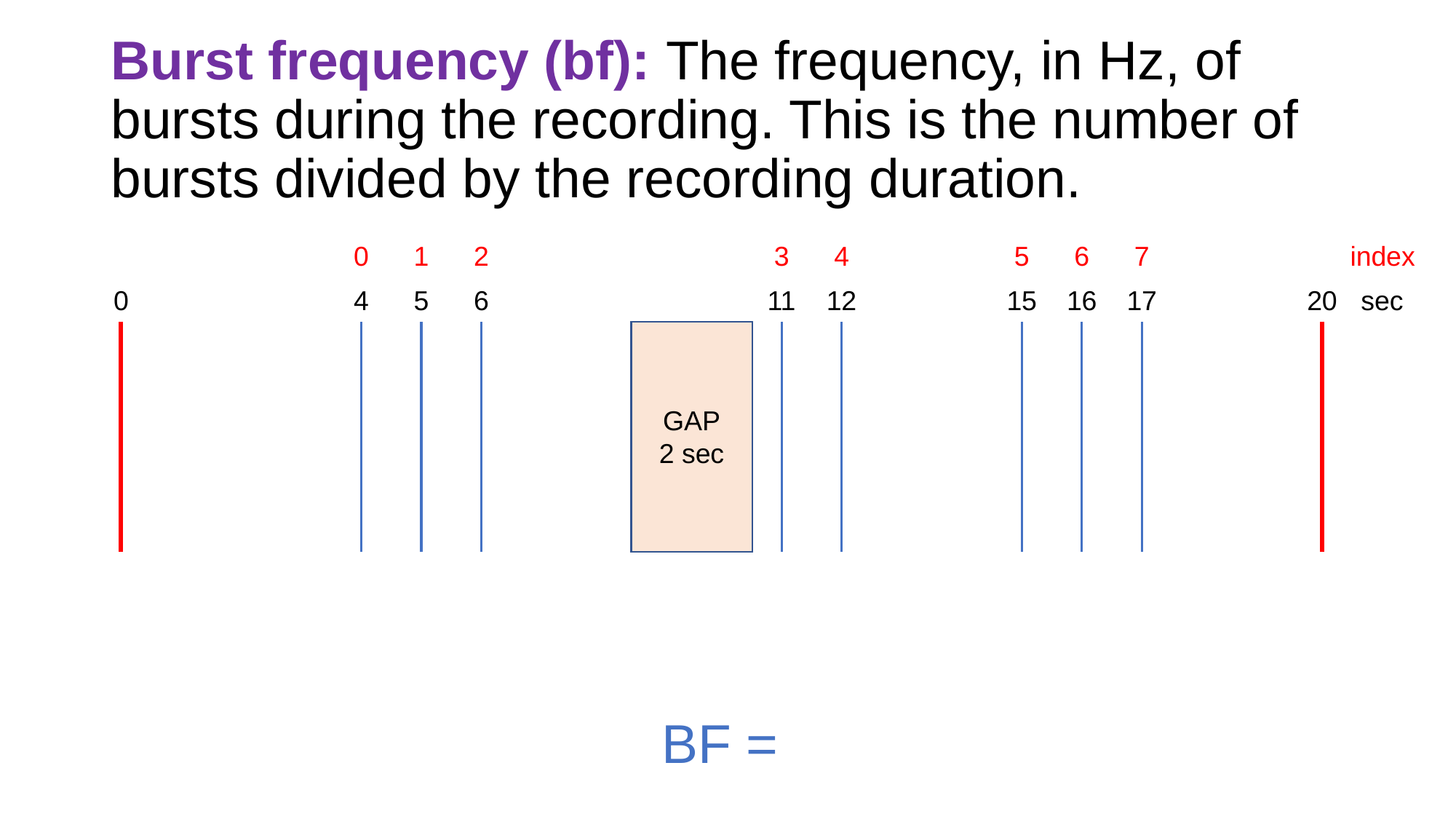

Burst frequency (bf): The frequency, in Hz, of bursts during the recording. This is the number of bursts divided by the recording duration.
0
1
2
3
4
5
6
7
index
0
4
5
6
11
12
15
16
17
20
sec
GAP
2 sec

## Slide 20
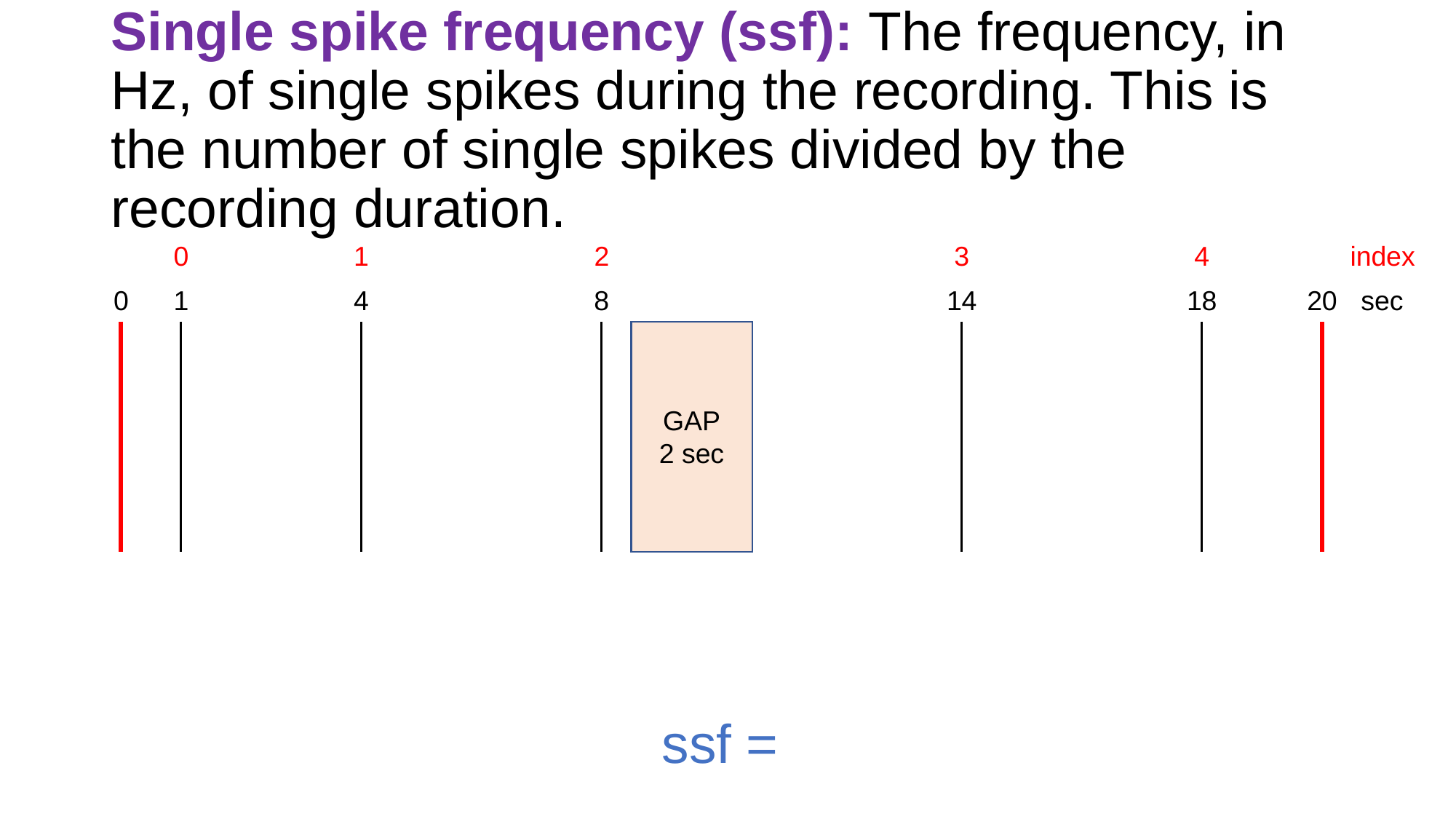

# Single spike frequency (ssf): The frequency, in Hz, of single spikes during the recording. This is the number of single spikes divided by the recording duration.
0
1
2
3
4
index
0
1
4
8
14
18
20
sec
GAP
2 sec

## Slide 21
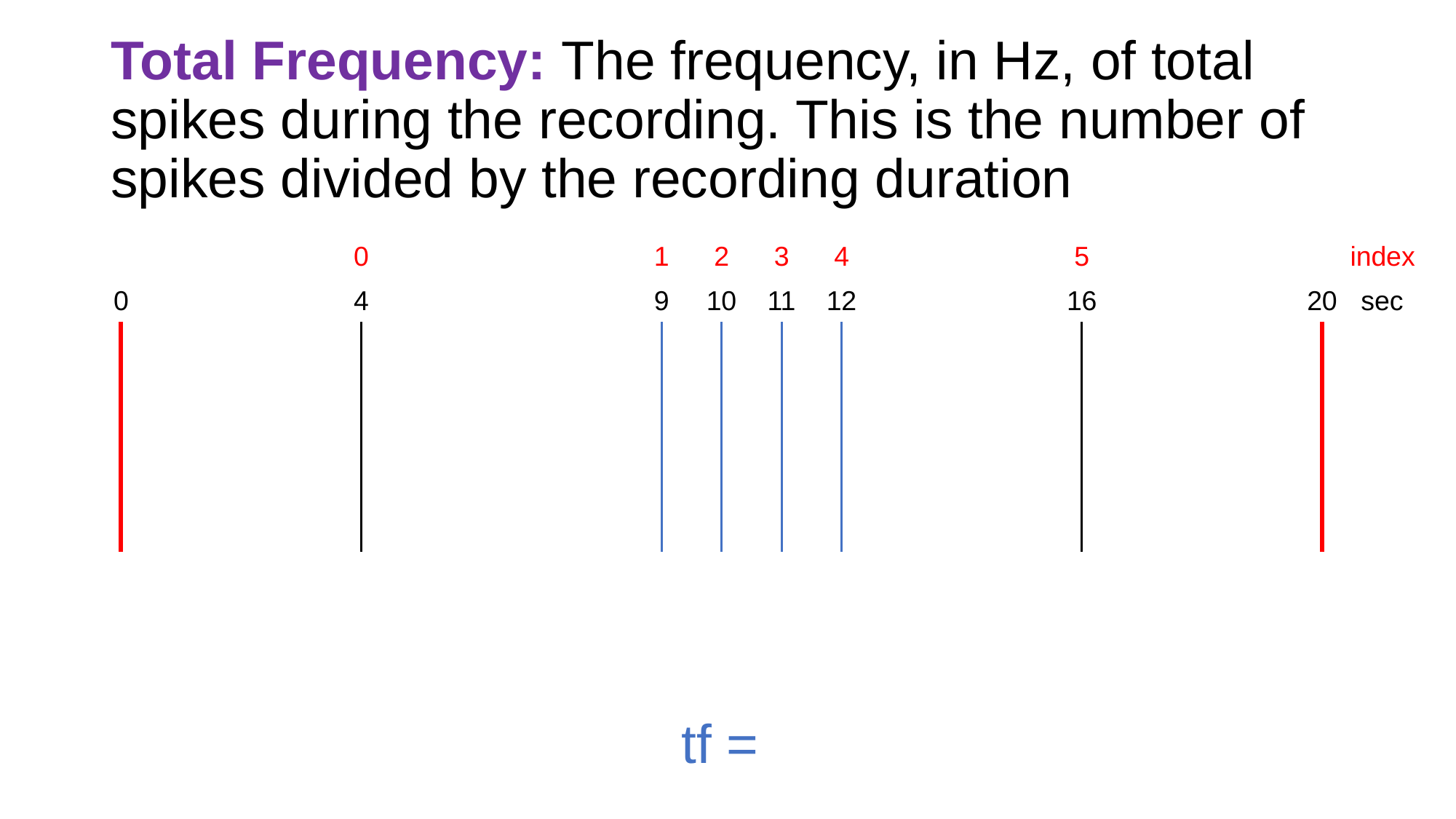

# Total Frequency: The frequency, in Hz, of total spikes during the recording. This is the number of spikes divided by the recording duration
0
1
2
3
4
5
index
0
4
9
10
11
12
16
20
sec

## Slide 22
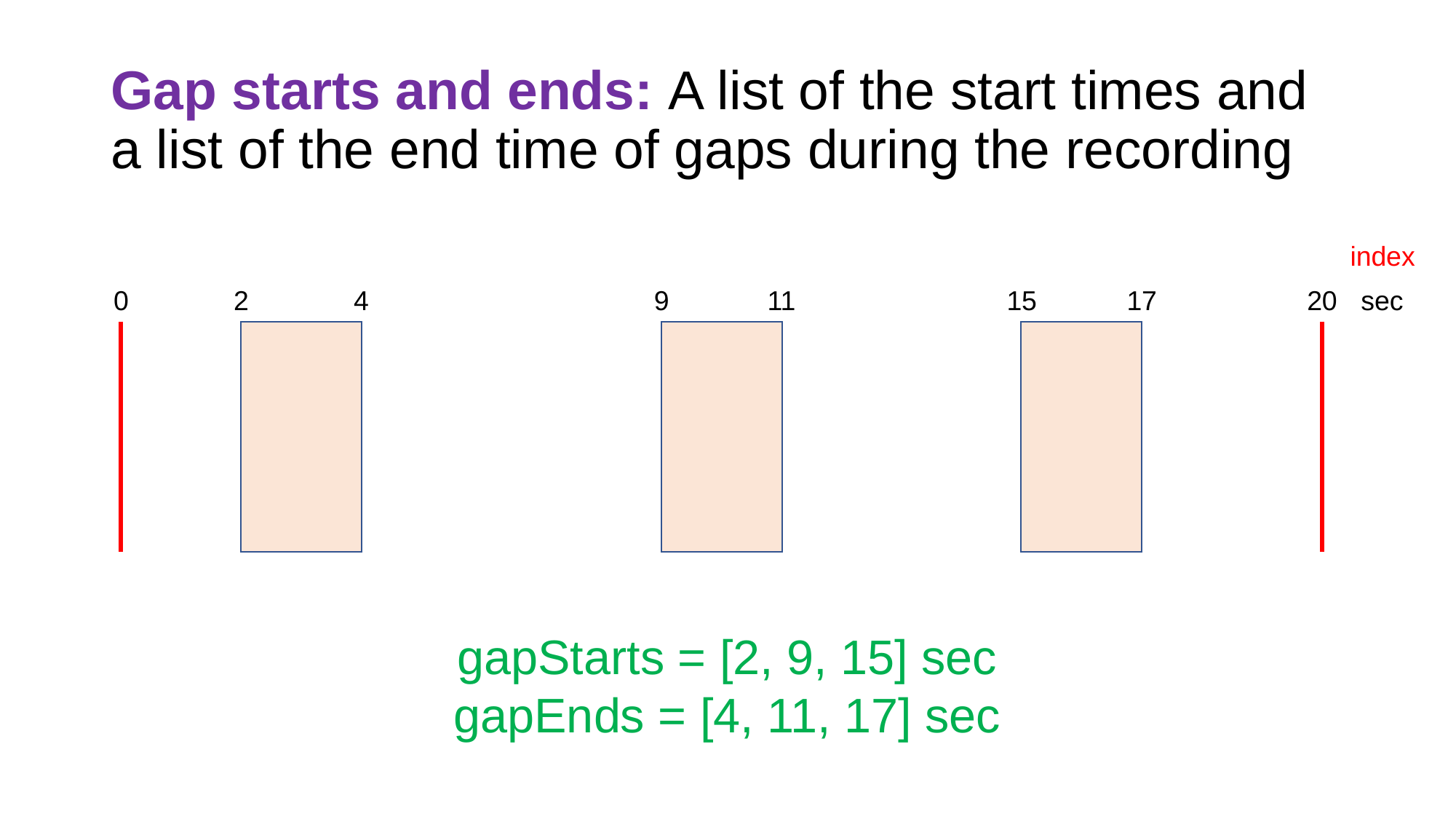

# Gap starts and ends: A list of the start times and a list of the end time of gaps during the recording
index
0
2
4
9
11
15
17
20
sec
gapStarts = [2, 9, 15] sec
gapEnds = [4, 11, 17] sec

## Slide 23
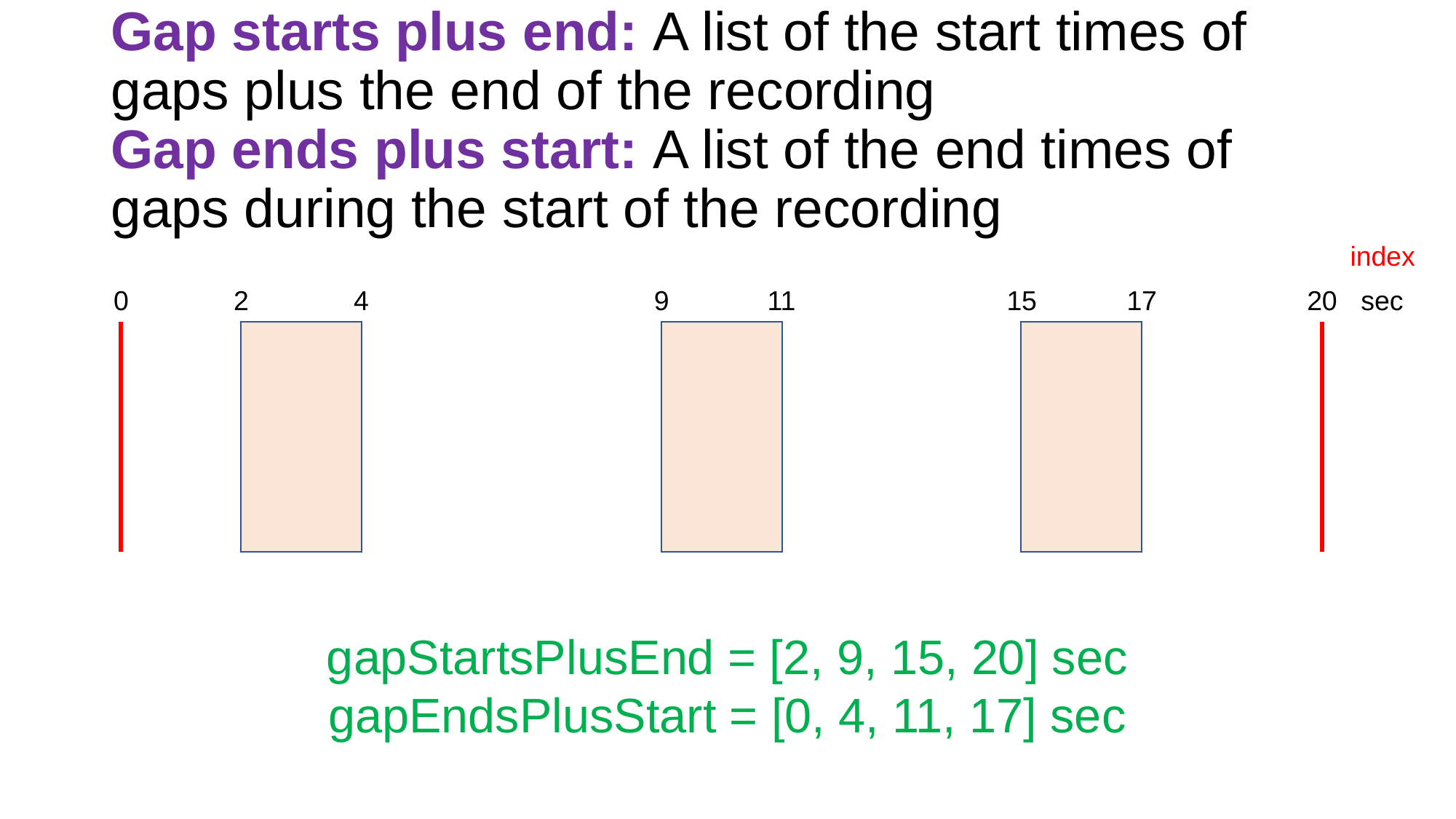

# Gap starts plus end: A list of the start times of gaps plus the end of the recording Gap ends plus start: A list of the end times of gaps during the start of the recording
index
0
2
4
9
11
15
17
20
sec
gapStartsPlusEnd = [2, 9, 15, 20] sec
gapEndsPlusStart = [0, 4, 11, 17] sec

## Slide 24
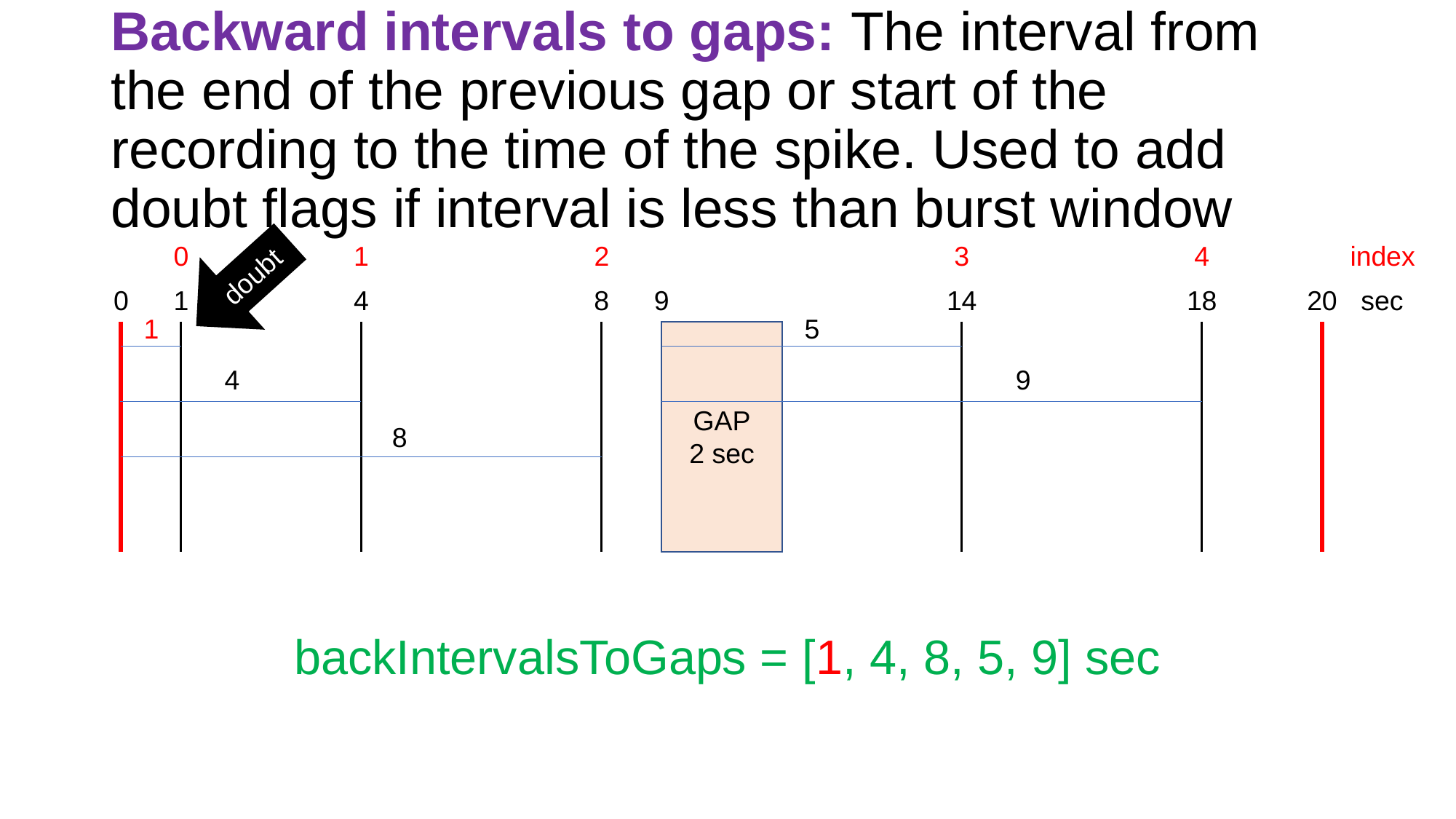

# Backward intervals to gaps: The interval from the end of the previous gap or start of the recording to the time of the spike. Used to add doubt flags if interval is less than burst window
doubt
0
1
2
3
4
index
0
1
4
8
9
14
18
20
sec
1
5
4
9
GAP
2 sec
8
backIntervalsToGaps = [1, 4, 8, 5, 9] sec

## Slide 25
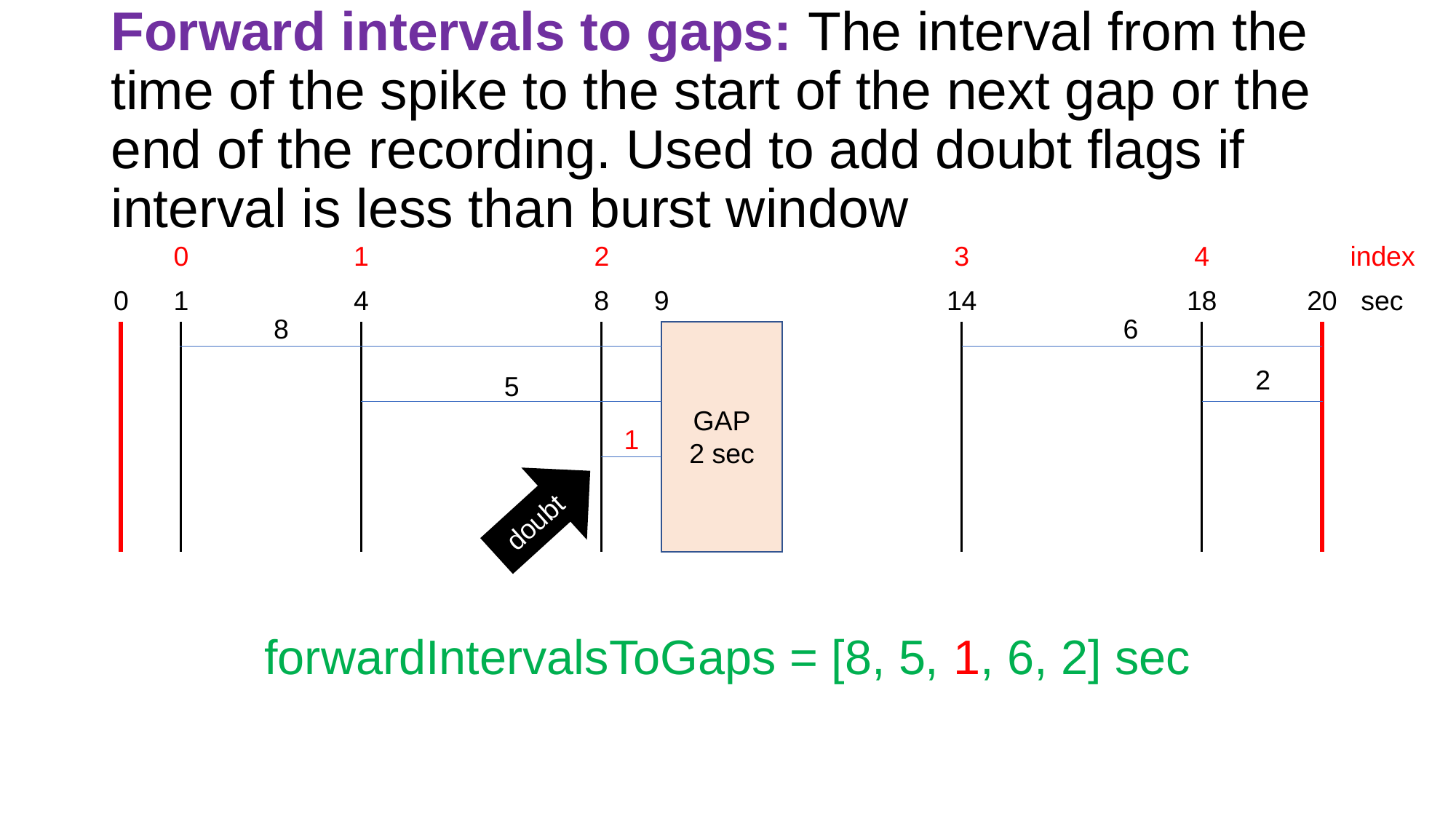

# Forward intervals to gaps: The interval from the time of the spike to the start of the next gap or the end of the recording. Used to add doubt flags if interval is less than burst window
0
1
2
3
4
index
0
1
4
8
9
14
18
20
sec
8
6
2
5
GAP
2 sec
1
doubt
forwardIntervalsToGaps = [8, 5, 1, 6, 2] sec

## Slide 26
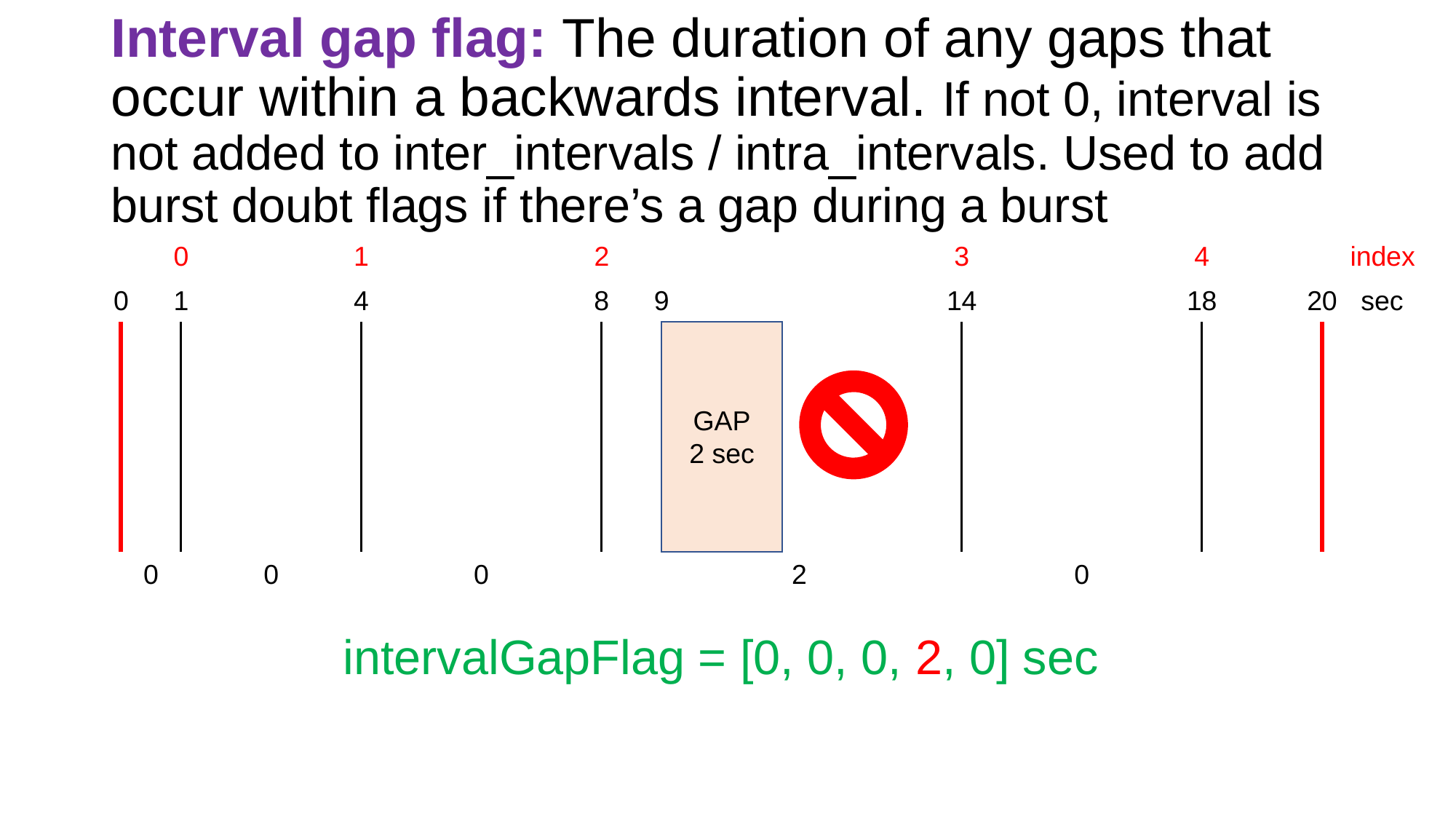

# Interval gap flag: The duration of any gaps that occur within a backwards interval. If not 0, interval is not added to inter_intervals / intra_intervals. Used to add burst doubt flags if there’s a gap during a burst
0
1
2
3
4
index
0
1
4
8
9
14
18
20
sec
GAP
2 sec
0
0
0
2
0
intervalGapFlag = [0, 0, 0, 2, 0] sec

## Slide 27
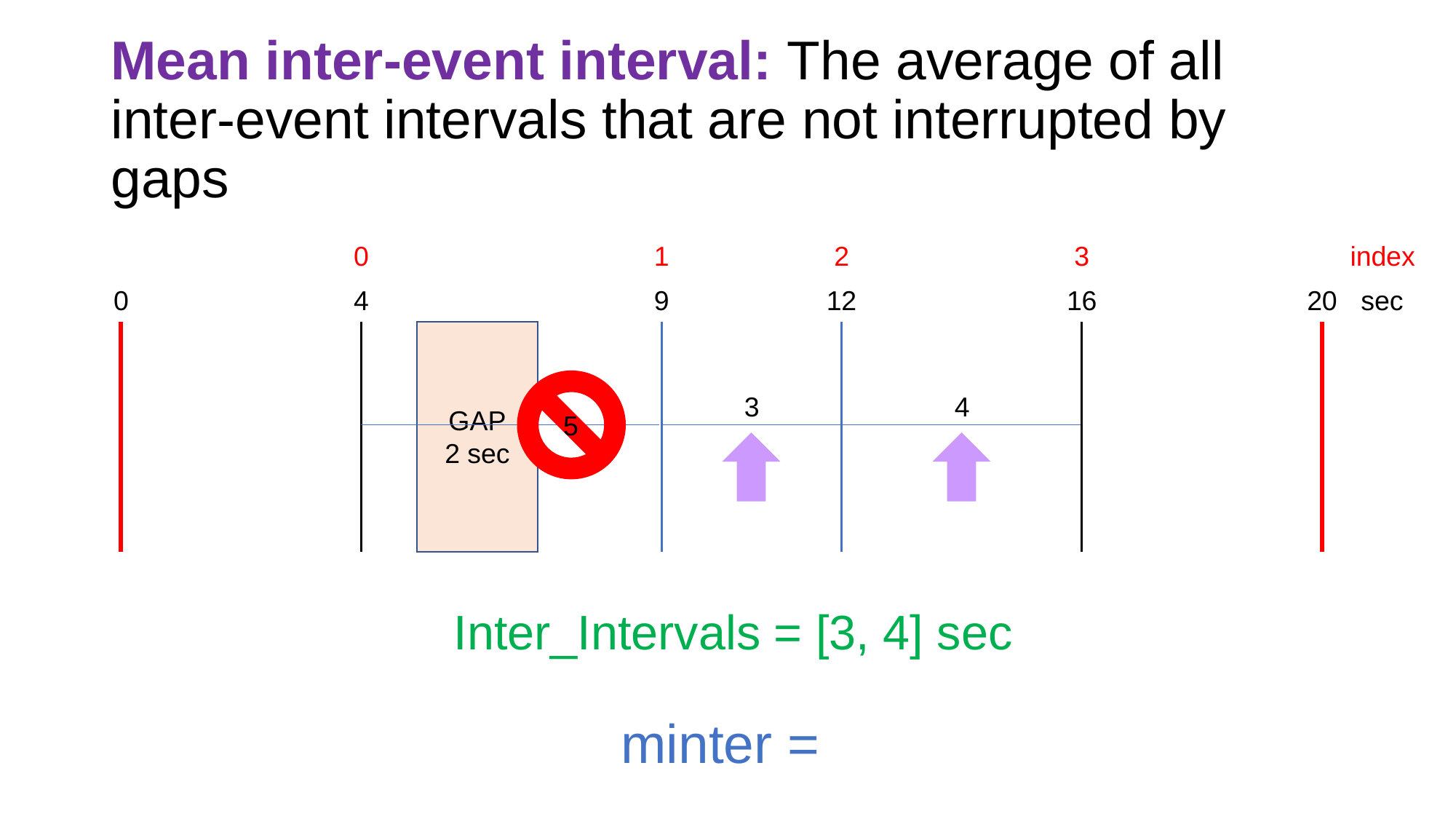

# Mean inter-event interval: The average of all inter-event intervals that are not interrupted by gaps
0
1
2
3
index
0
4
9
12
16
20
sec
3
4
GAP
2 sec
5
Inter_Intervals = [3, 4] sec

## Slide 28
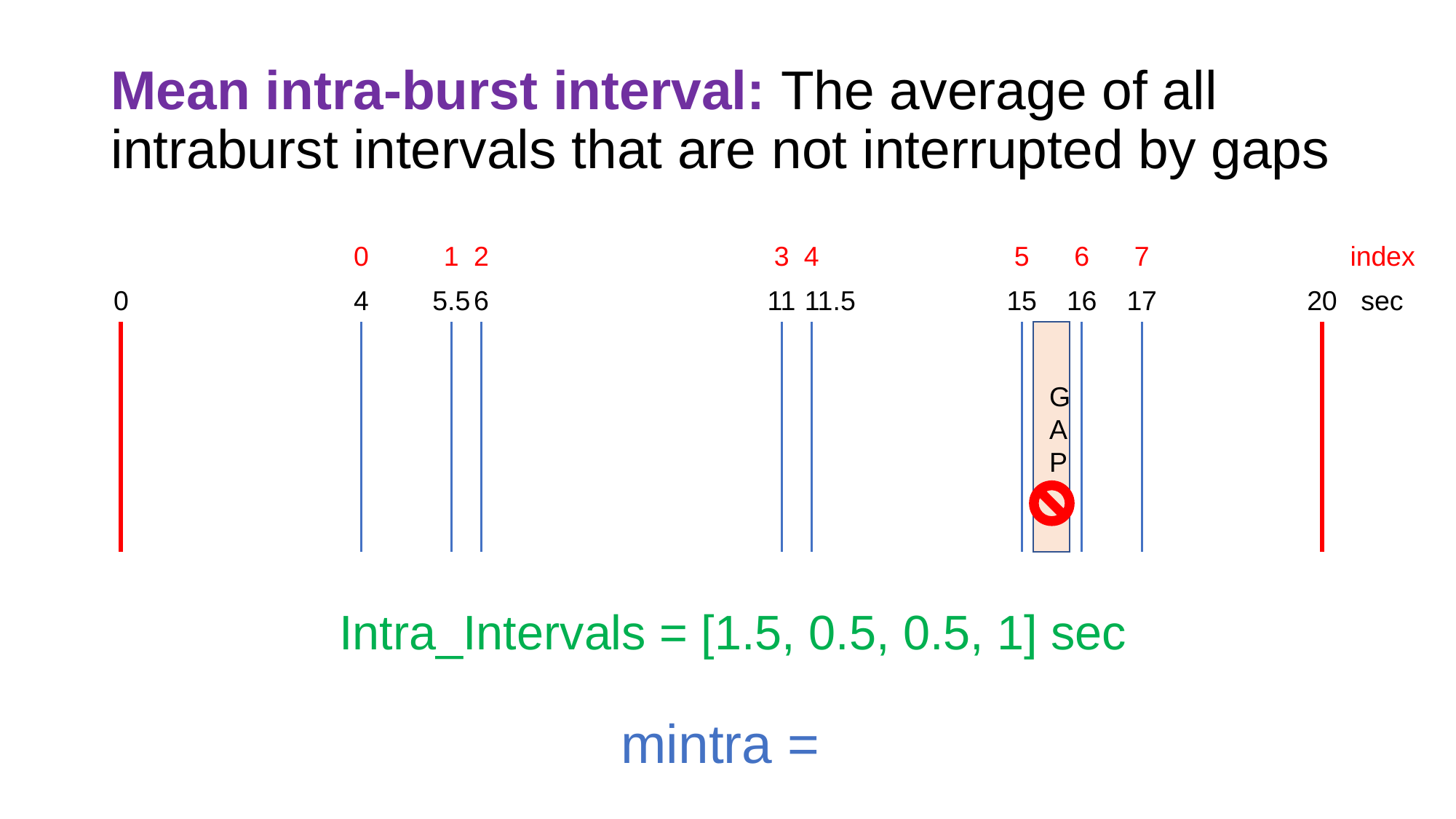

# Mean intra-burst interval: The average of all intraburst intervals that are not interrupted by gaps
0
1
2
3
4
5
6
7
index
0
4
5.5
6
11
11.5
15
16
17
20
sec
GAP
Intra_Intervals = [1.5, 0.5, 0.5, 1] sec

## Slide 29
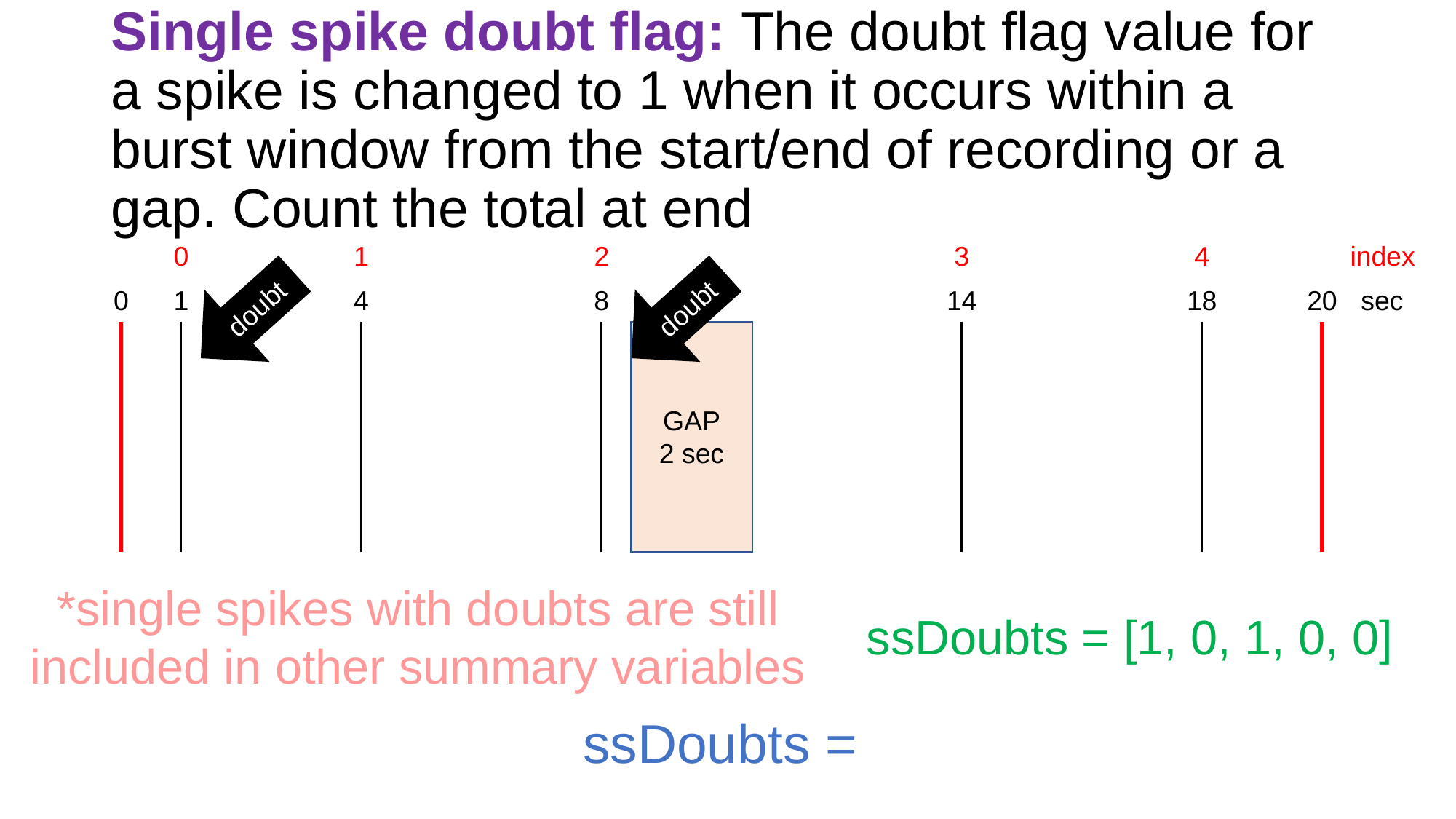

# Single spike doubt flag: The doubt flag value for a spike is changed to 1 when it occurs within a burst window from the start/end of recording or a gap. Count the total at end
0
1
2
3
4
index
doubt
doubt
0
1
4
8
14
18
20
sec
GAP
2 sec
*single spikes with doubts are still included in other summary variables
ssDoubts = [1, 0, 1, 0, 0]

## Slide 30
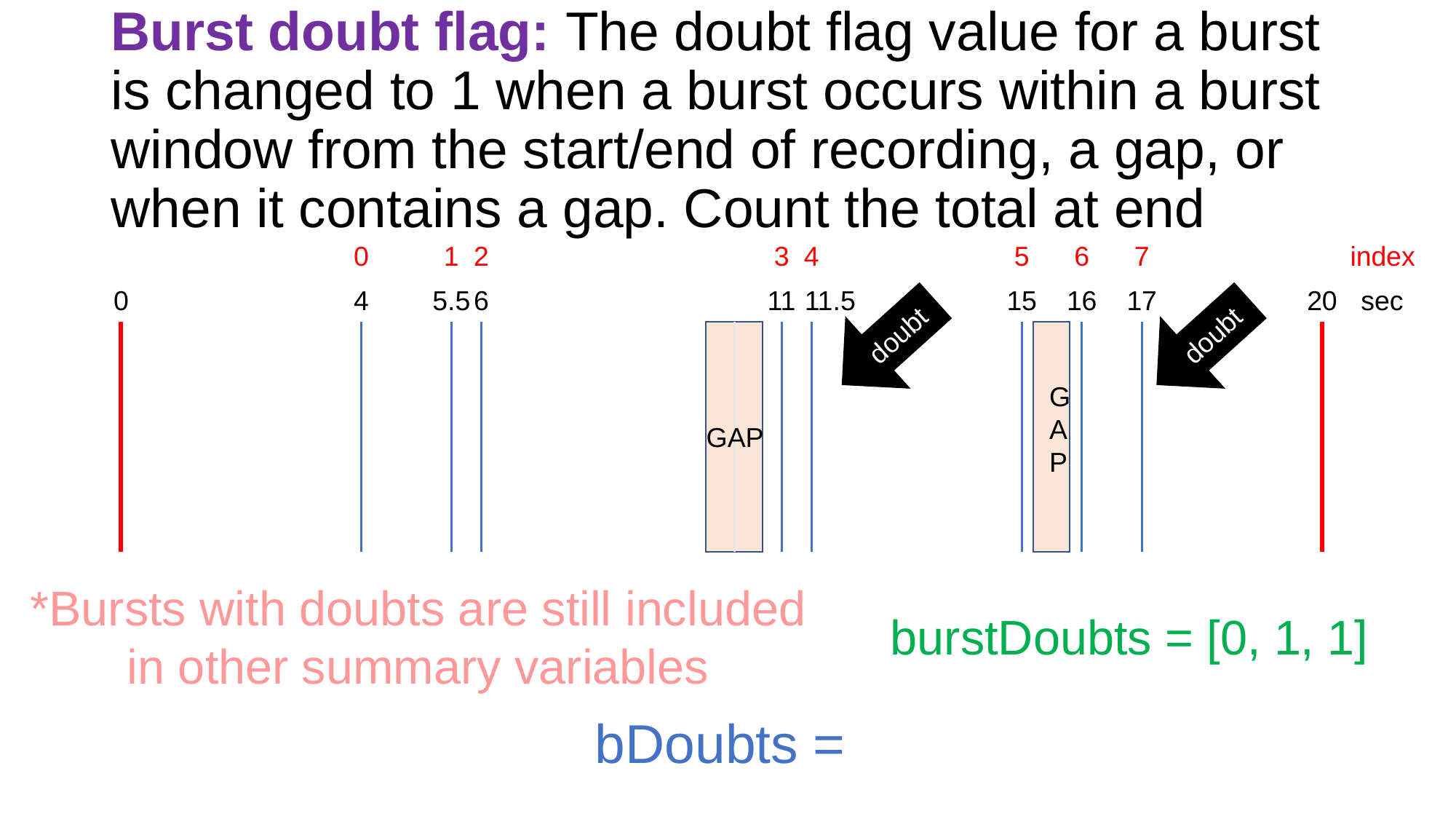

# Burst doubt flag: The doubt flag value for a burst is changed to 1 when a burst occurs within a burst window from the start/end of recording, a gap, or when it contains a gap. Count the total at end
0
1
2
3
4
5
6
7
index
0
4
5.5
6
11
11.5
15
16
17
20
sec
doubt
doubt
GAP
GAP
*Bursts with doubts are still included in other summary variables
burstDoubts = [0, 1, 1]

## Slide 31
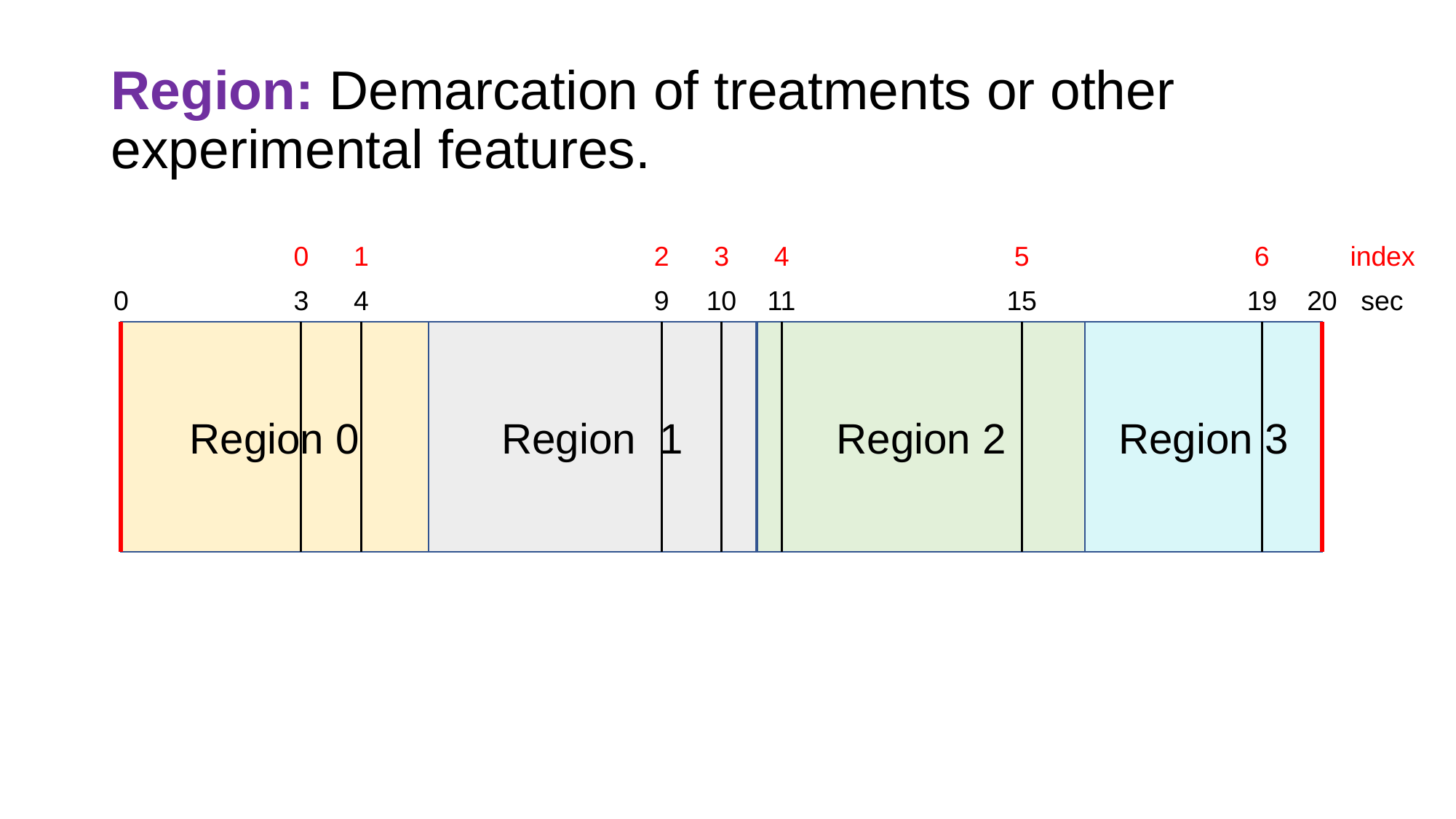

# Region: Demarcation of treatments or other experimental features.
0
1
2
3
4
5
6
index
0
3
4
9
10
11
15
19
20
sec
Region 0
Region 1
Region 2
Region 3

## Slide 32
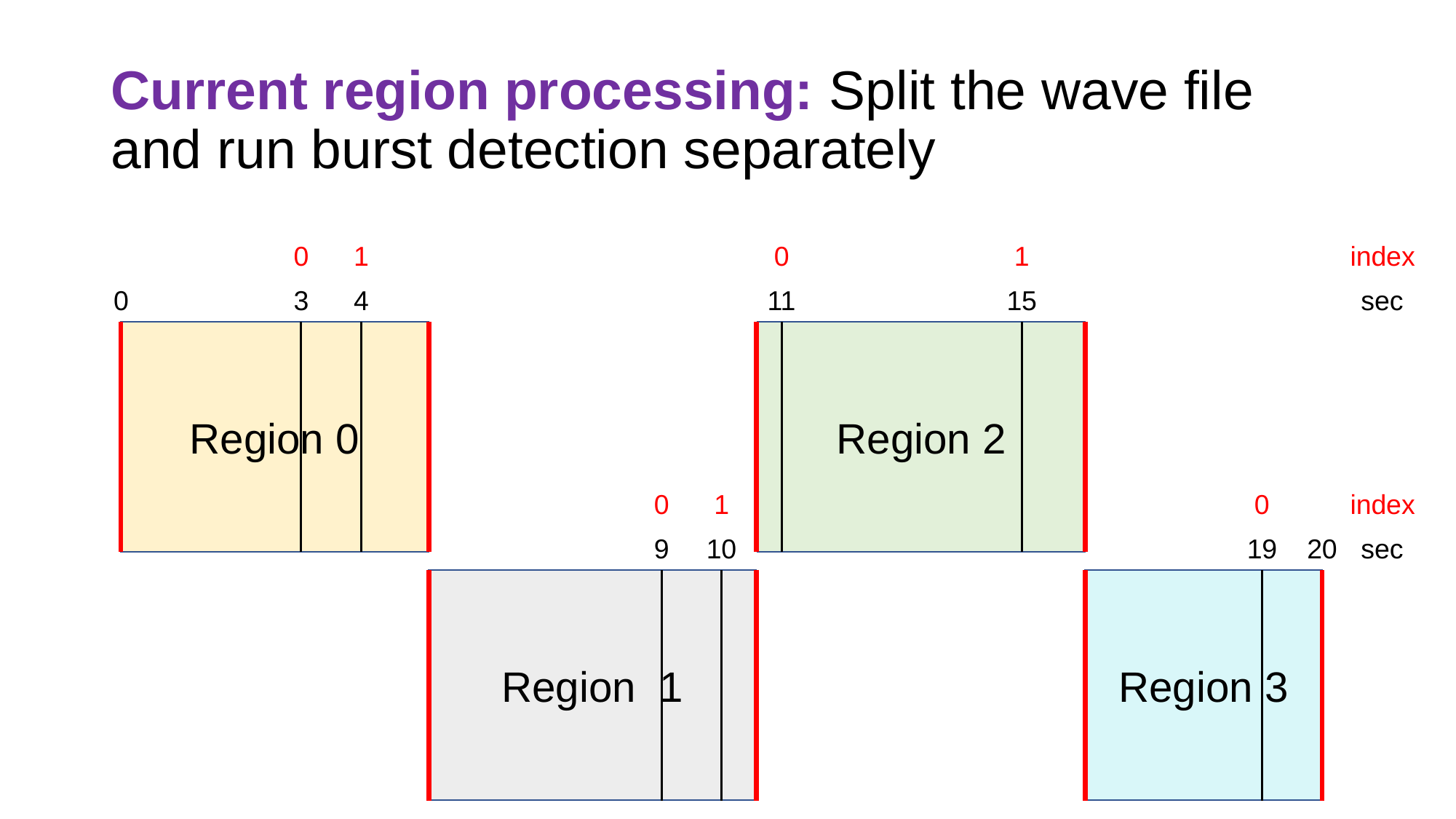

# Current region processing: Split the wave file and run burst detection separately
0
1
0
1
index
0
3
4
11
15
sec
Region 0
Region 2
0
1
0
index
9
10
19
20
sec
Region 1
Region 3

## Slide 33
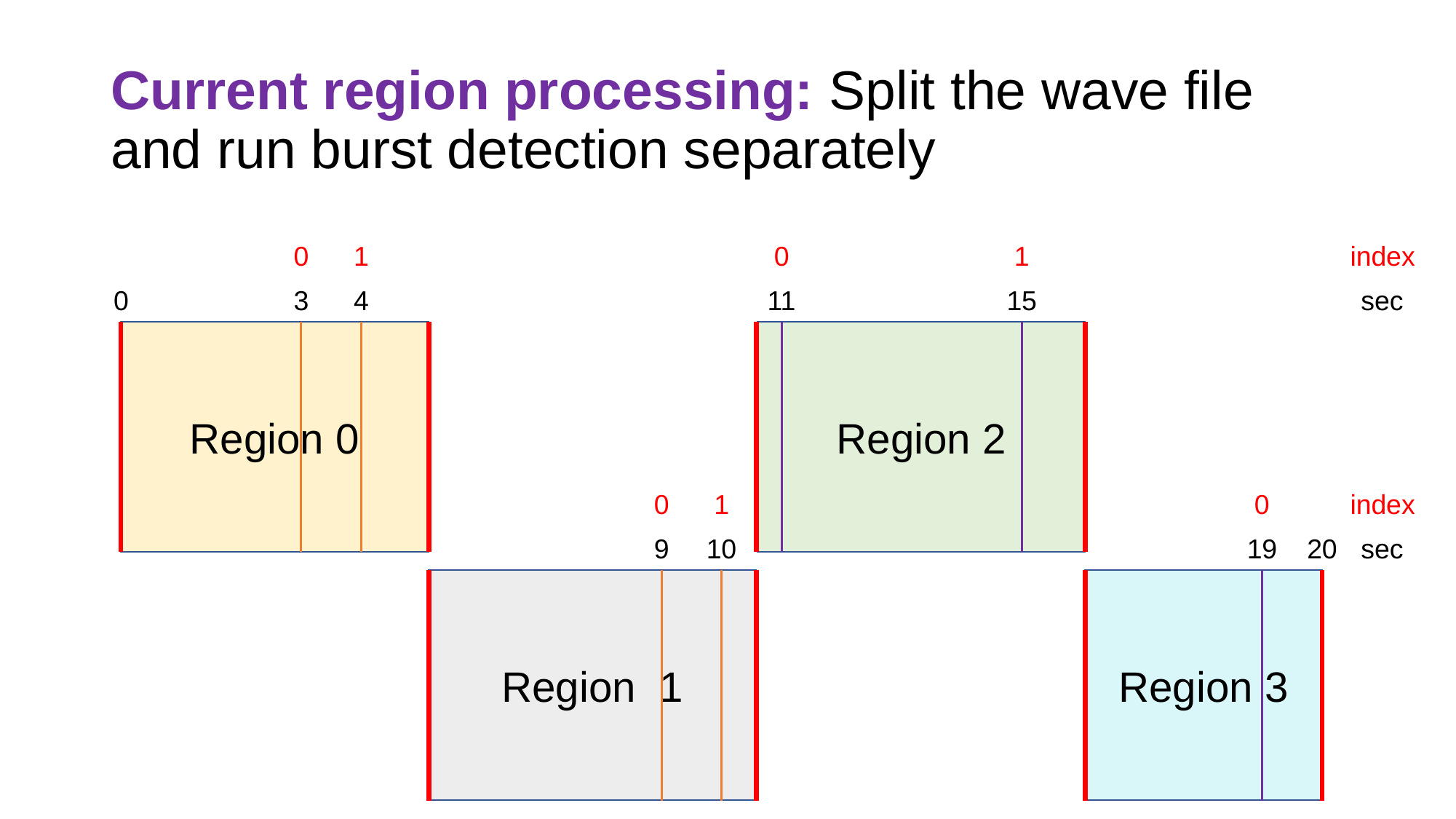

# Current region processing: Split the wave file and run burst detection separately
0
1
0
1
index
0
3
4
11
15
sec
Region 0
Region 2
0
1
0
index
9
10
19
20
sec
Region 1
Region 3

## Slide 34
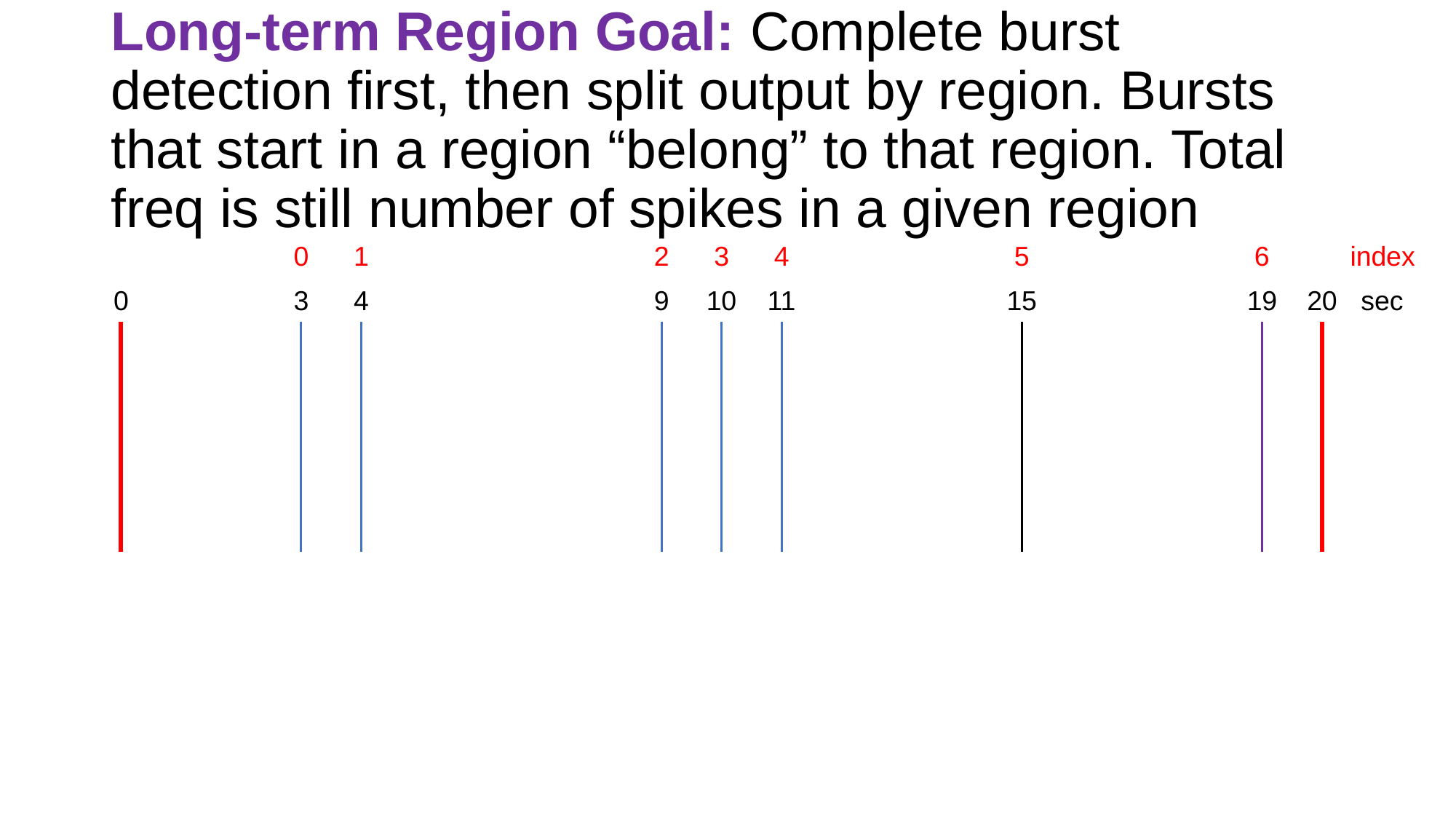

# Long-term Region Goal: Complete burst detection first, then split output by region. Bursts that start in a region “belong” to that region. Total freq is still number of spikes in a given region
0
1
2
3
4
5
6
index
0
3
4
9
10
11
15
19
20
sec

## Slide 35
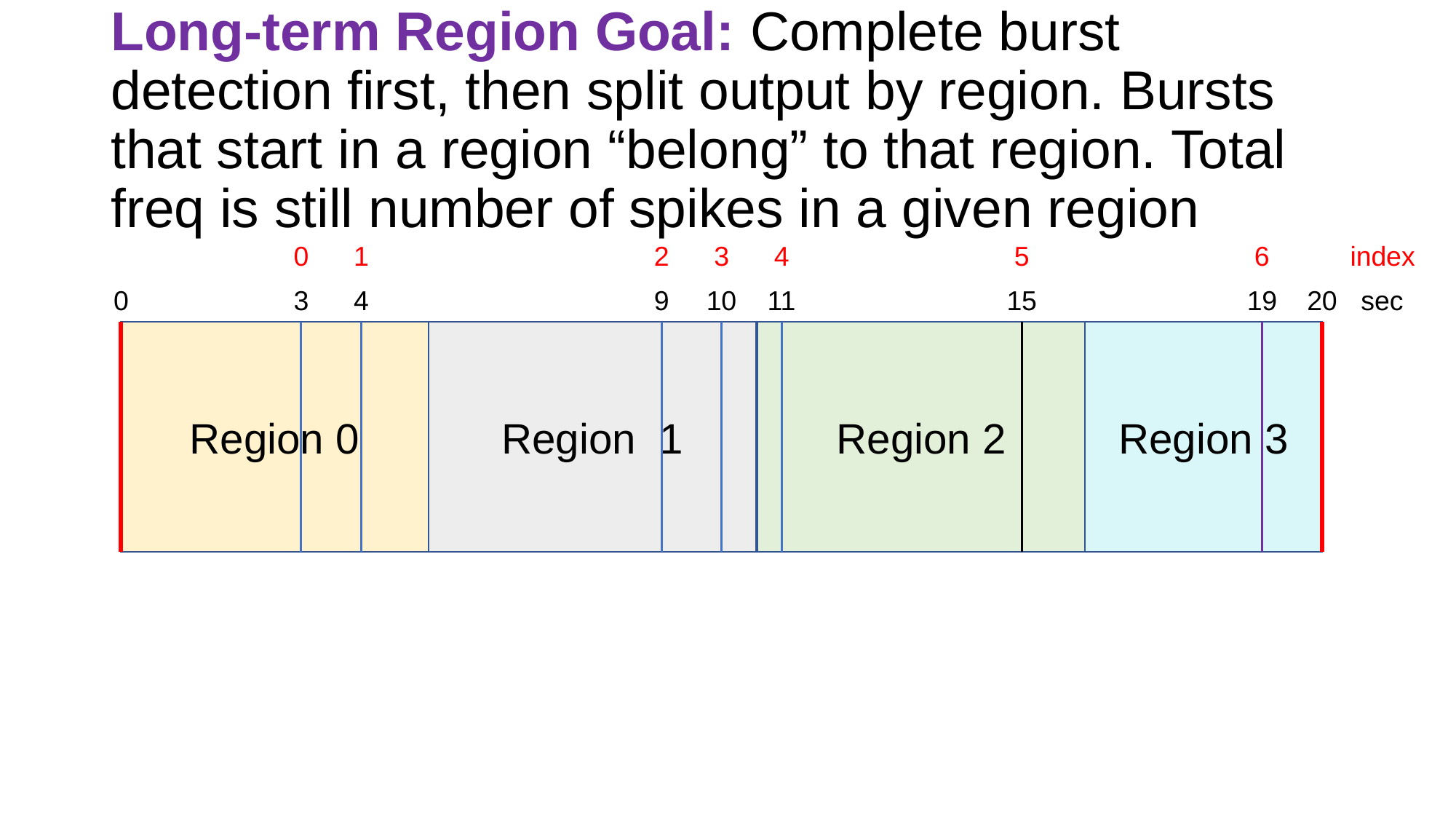

# Long-term Region Goal: Complete burst detection first, then split output by region. Bursts that start in a region “belong” to that region. Total freq is still number of spikes in a given region
0
1
2
3
4
5
6
index
0
3
4
9
10
11
15
19
20
sec
Region 0
Region 1
Region 2
Region 3
