# Supplementary material for: Early-Life Resource Scarcity in Mice Does Not Alter Adult Corticosterone or Preovulatory Luteinizing Hormone Surge Responses to Acute Psychosocial Stress
Source: eNeuro. 2024 Jul 26;11(7):ENEURO.0125-24.2024. doi: 10.1523/ENEURO.0125-24.2024 (PMC11287788; doi:10.1523/ENEURO.0125-24.2024)
Supplement: Extended Data — Zip file of custom code for PSC detection and analysis, ffmpeg recording of dam behavior, and R analysis. Download Extended Data, ZIP file. [file eneuro-11-ENEURO.0125-24.2024-s002.zip › PSC-analysis/AGG_VBWPanel/helpDocs/VBW_analysis_scenarios-preMeeting_SM.pptx]

## Slide 1
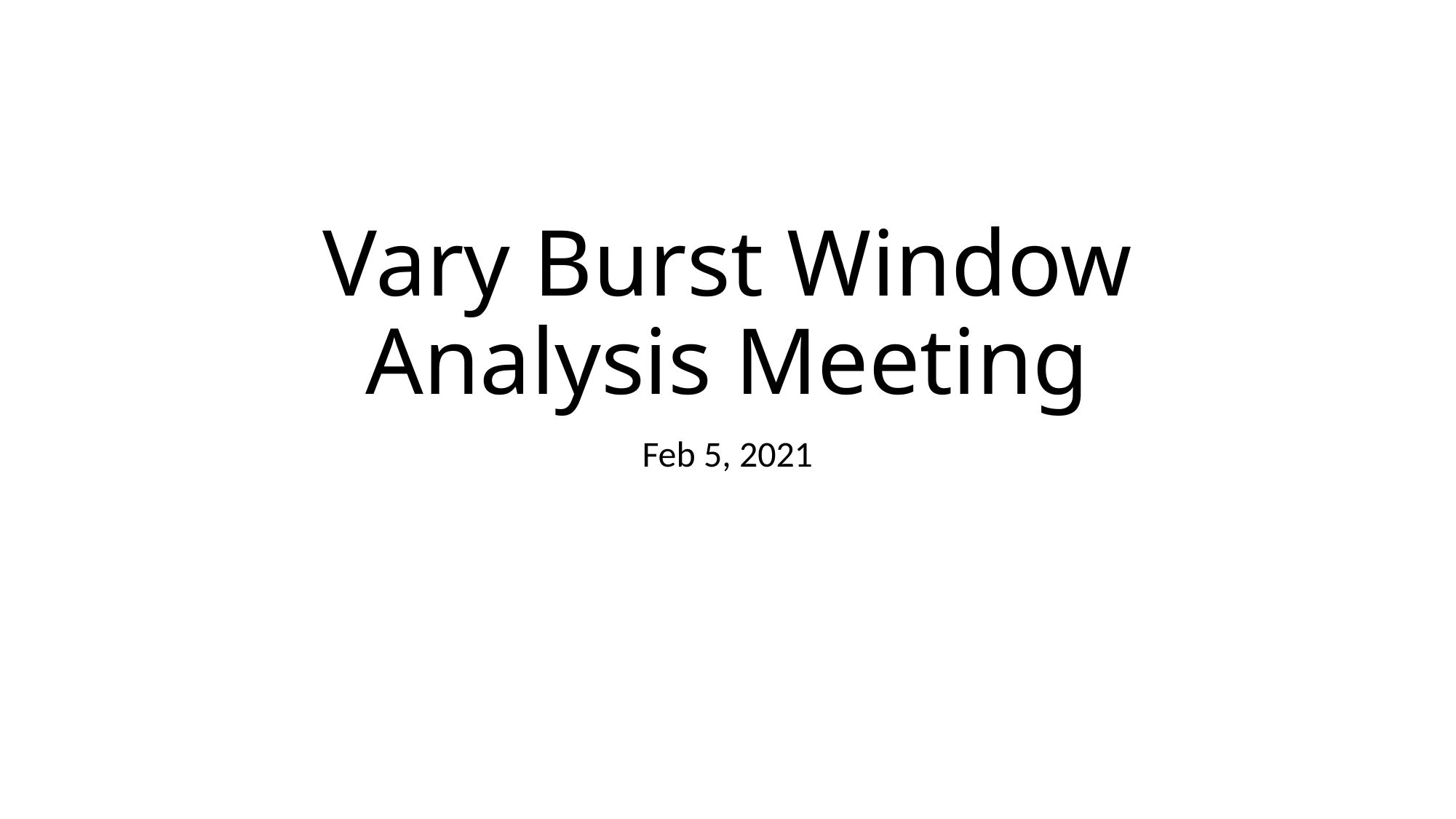

# Vary Burst Window Analysis Meeting
Feb 5, 2021

## Slide 2
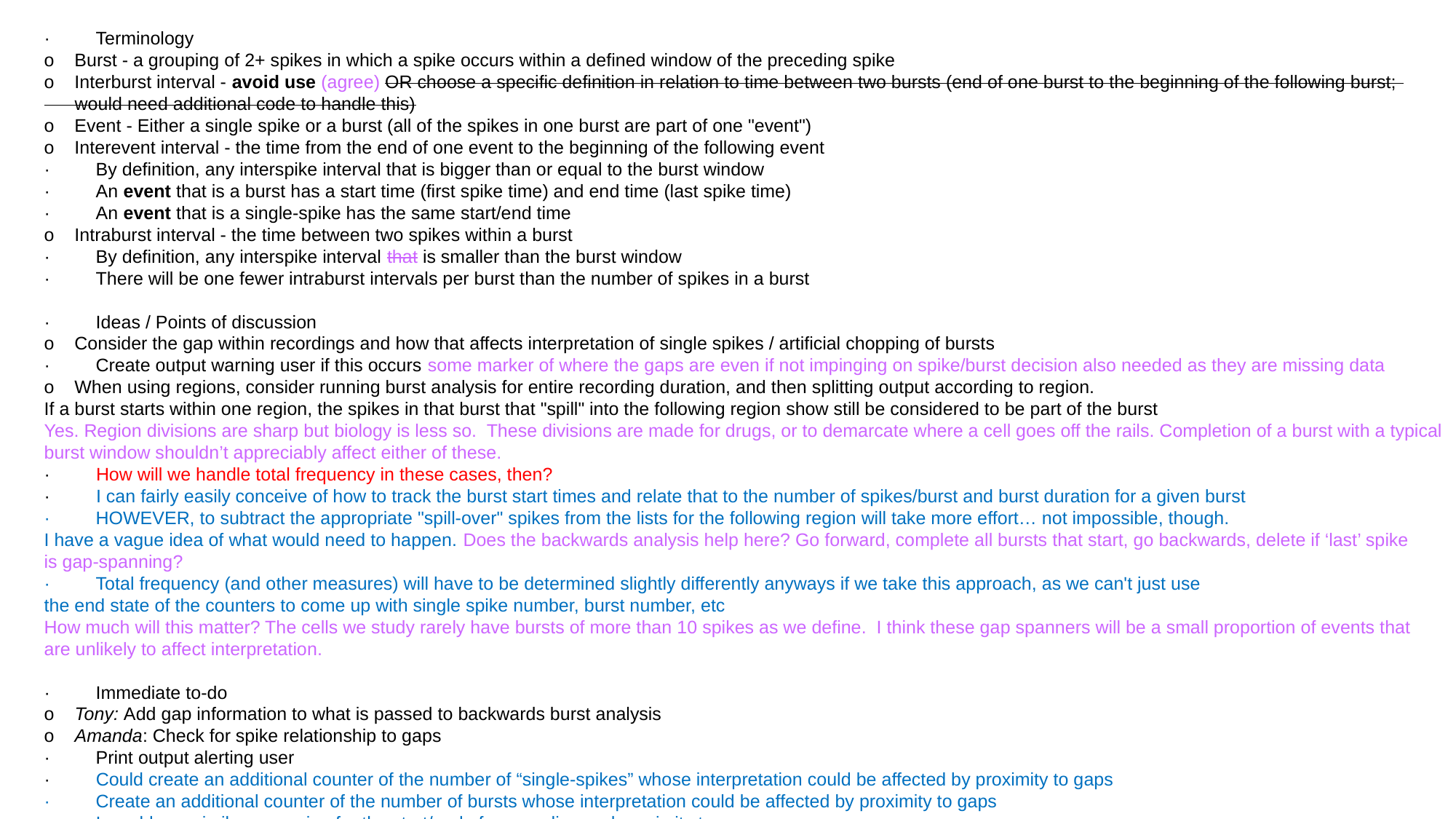

·         Terminology
o    Burst - a grouping of 2+ spikes in which a spike occurs within a defined window of the preceding spike
o    Interburst interval - avoid use (agree) OR choose a specific definition in relation to time between two bursts (end of one burst to the beginning of the following burst;
 would need additional code to handle this)
o    Event - Either a single spike or a burst (all of the spikes in one burst are part of one "event")
o    Interevent interval - the time from the end of one event to the beginning of the following event
·         By definition, any interspike interval that is bigger than or equal to the burst window
·         An event that is a burst has a start time (first spike time) and end time (last spike time)
·         An event that is a single-spike has the same start/end time
o    Intraburst interval - the time between two spikes within a burst
·         By definition, any interspike interval that is smaller than the burst window
·         There will be one fewer intraburst intervals per burst than the number of spikes in a burst
·         Ideas / Points of discussion
o    Consider the gap within recordings and how that affects interpretation of single spikes / artificial chopping of bursts
·         Create output warning user if this occurs some marker of where the gaps are even if not impinging on spike/burst decision also needed as they are missing data
o    When using regions, consider running burst analysis for entire recording duration, and then splitting output according to region.
If a burst starts within one region, the spikes in that burst that "spill" into the following region show still be considered to be part of the burst
Yes. Region divisions are sharp but biology is less so. These divisions are made for drugs, or to demarcate where a cell goes off the rails. Completion of a burst with a typical
burst window shouldn’t appreciably affect either of these.
·         How will we handle total frequency in these cases, then?
·         I can fairly easily conceive of how to track the burst start times and relate that to the number of spikes/burst and burst duration for a given burst
·         HOWEVER, to subtract the appropriate "spill-over" spikes from the lists for the following region will take more effort… not impossible, though.
I have a vague idea of what would need to happen. Does the backwards analysis help here? Go forward, complete all bursts that start, go backwards, delete if ‘last’ spike
is gap-spanning?
·         Total frequency (and other measures) will have to be determined slightly differently anyways if we take this approach, as we can't just use
the end state of the counters to come up with single spike number, burst number, etc
How much will this matter? The cells we study rarely have bursts of more than 10 spikes as we define. I think these gap spanners will be a small proportion of events that
are unlikely to affect interpretation.
·         Immediate to-do
o    Tony: Add gap information to what is passed to backwards burst analysis
o    Amanda: Check for spike relationship to gaps
·         Print output alerting user
·         Could create an additional counter of the number of “single-spikes” whose interpretation could be affected by proximity to gaps
·         Create an additional counter of the number of bursts whose interpretation could be affected by proximity to gaps
·         I would use similar reasoning for the start/end of a recording and proximity to a gap
o    Amanda: Add tracker for burst start times
·         Preps for long-term ability to use single detection analysis ????? not sure what this means-is this the single burst detection analysis mentioned below?
·         Then, for region analysis, you would need to check for each burst if the start time occurs within the region.
If it does, you'd add the corresponding burst duration and burst times to the list for that region
·         You'd do something similar with the single spikes, where you'd check if the time of the single spike is within the region, and if it is,
you'd add the spike information to the list for that region
·         Would need some way to handle the potential single spikes that are near start/end or gaps. Might need to create a separate list to track these during the primary analysis.
·
 Long-term
o    Use a single burst detection analysis, regardless of region information, and then divide this analysis based on the regions
·         This is not going to have major effect on the outcomes for the spontaneous firing rate analysis…
therefore, not top priority right now, and would require significant reorganizing of how the analysis is handled
§  There could be a burst that continues past the end of the designated "spontaneous" region; however, since we're using the hard cutoff of time for duration
(and therefore frequency calculations), I think that I still feel kind of iffy about counting spikes that occur in a burst after we've said that the region ended.
If we just stopped the physical recording at that point, we don't know that those other spikes had occurs. The point of regions is that you didn’t stop recording, you did
something-treatment or made a subjective judgement about recording quality.

## Slide 3
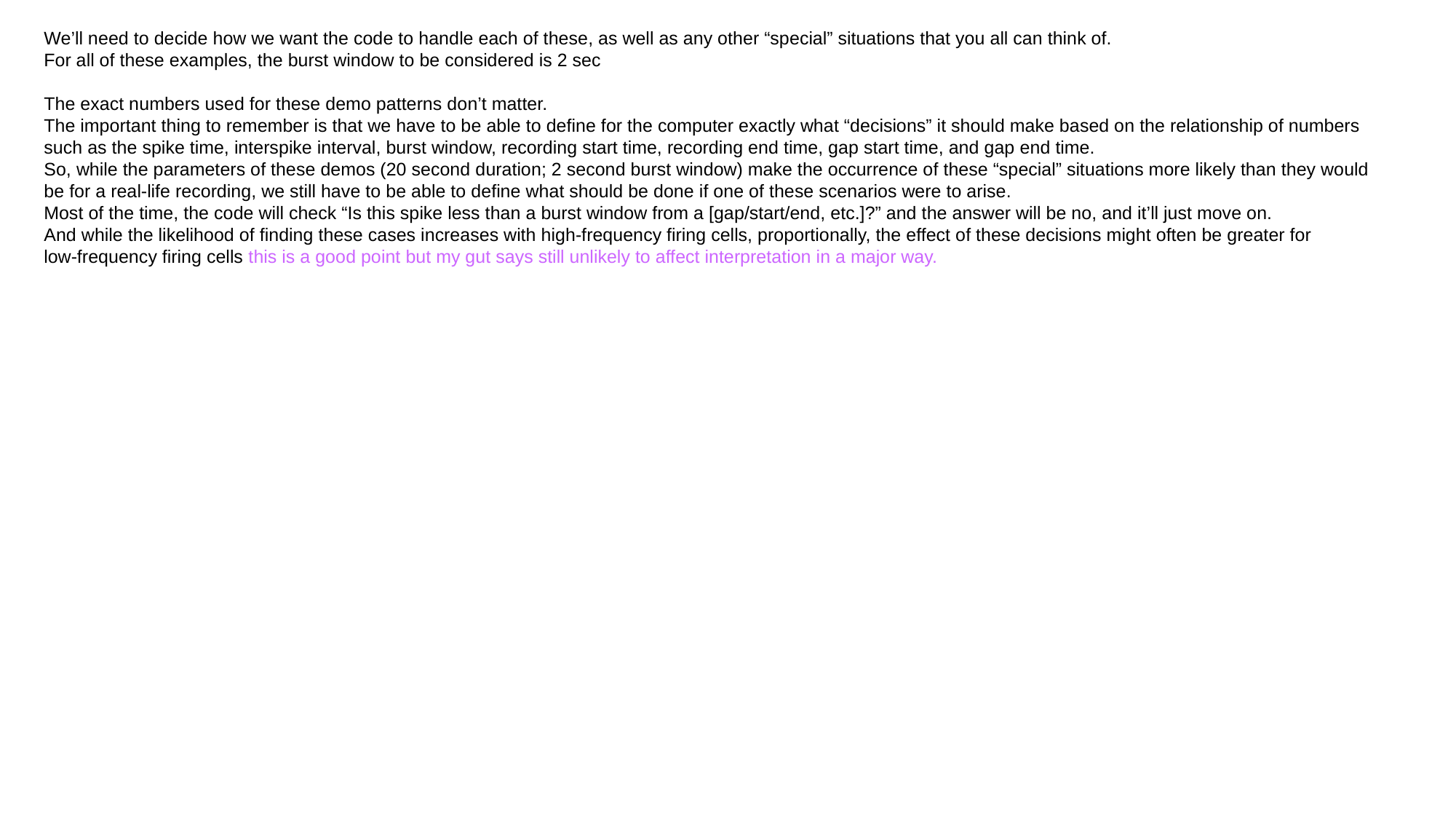

We’ll need to decide how we want the code to handle each of these, as well as any other “special” situations that you all can think of.
For all of these examples, the burst window to be considered is 2 sec
The exact numbers used for these demo patterns don’t matter.
The important thing to remember is that we have to be able to define for the computer exactly what “decisions” it should make based on the relationship of numbers
such as the spike time, interspike interval, burst window, recording start time, recording end time, gap start time, and gap end time.
So, while the parameters of these demos (20 second duration; 2 second burst window) make the occurrence of these “special” situations more likely than they would
be for a real-life recording, we still have to be able to define what should be done if one of these scenarios were to arise.
Most of the time, the code will check “Is this spike less than a burst window from a [gap/start/end, etc.]?” and the answer will be no, and it’ll just move on.
And while the likelihood of finding these cases increases with high-frequency firing cells, proportionally, the effect of these decisions might often be greater for
low-frequency firing cells this is a good point but my gut says still unlikely to affect interpretation in a major way.

## Slide 4
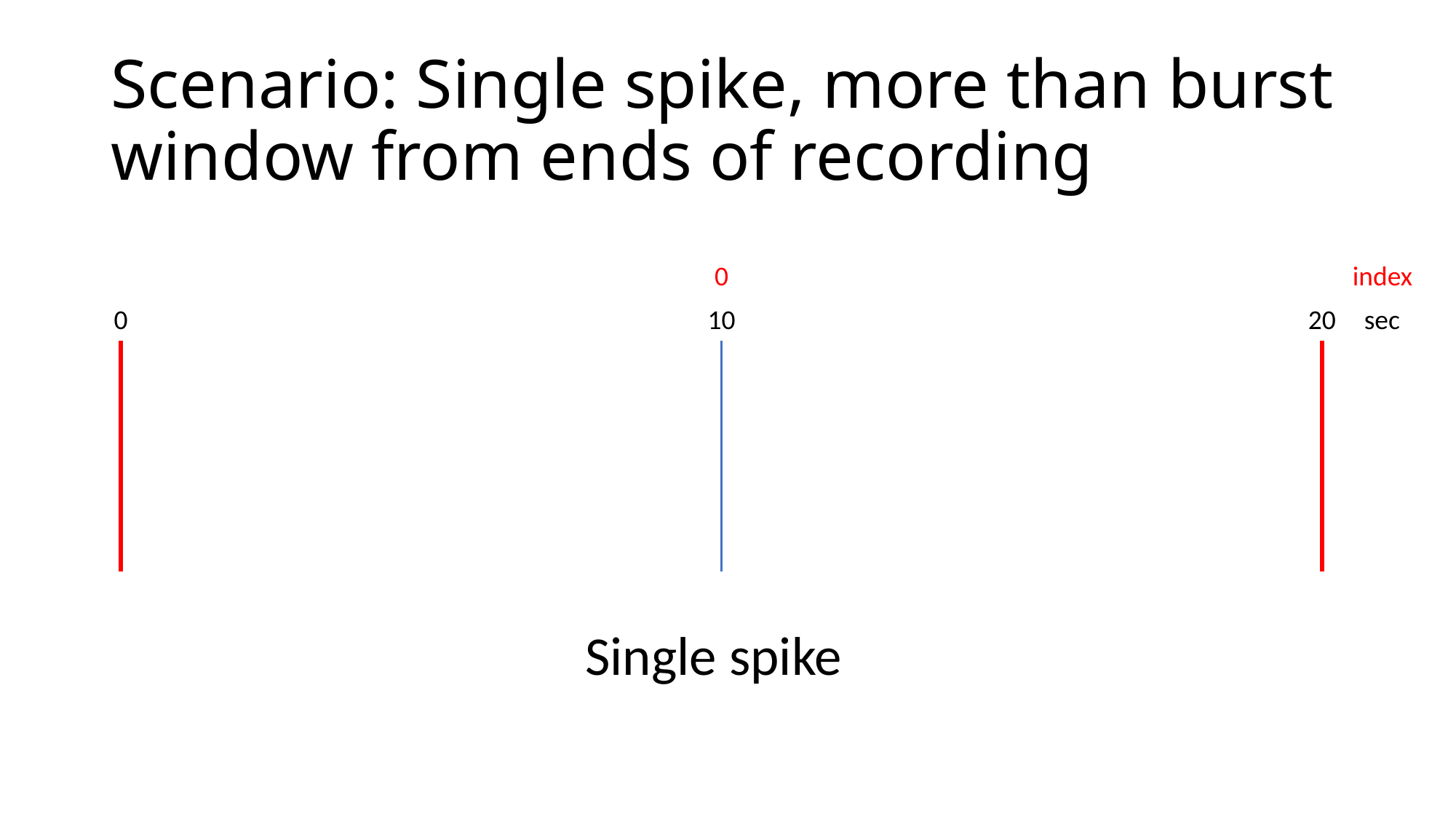

# Scenario: Single spike, more than burst window from ends of recording
0
index
0
10
20
sec
Single spike

## Slide 5
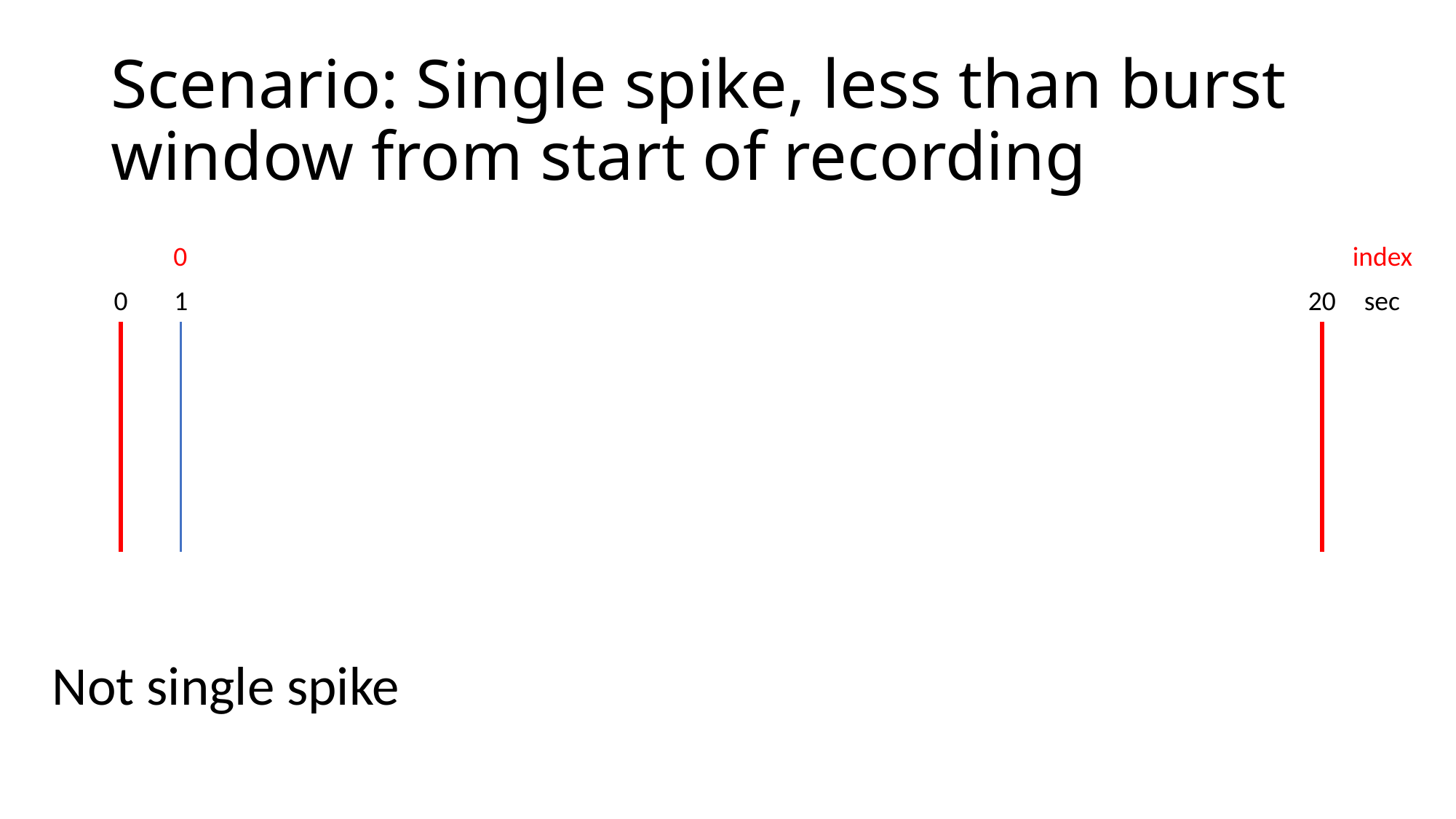

# Scenario: Single spike, less than burst window from start of recording
0
index
0
1
20
sec
Not single spike

## Slide 6
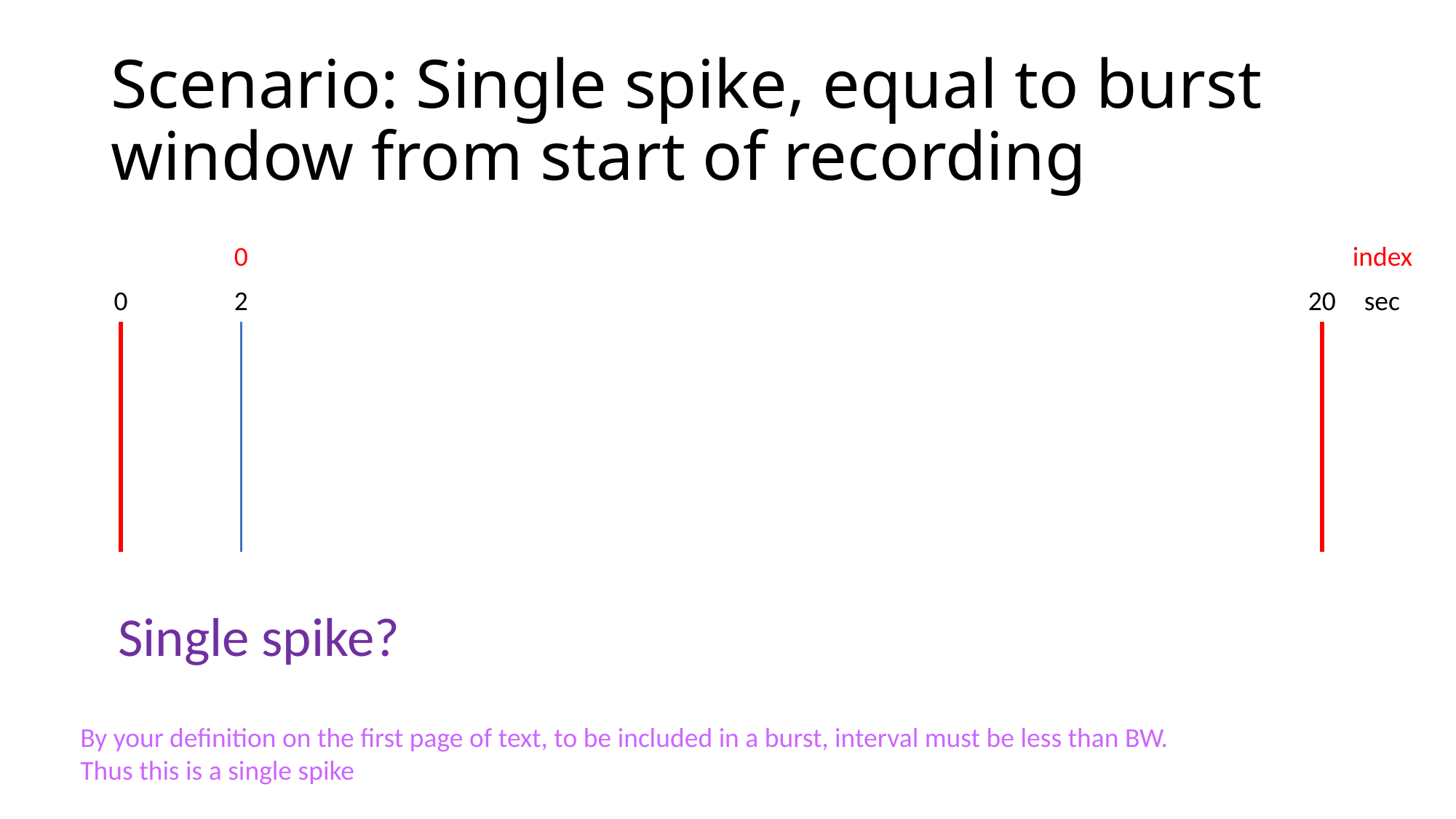

# Scenario: Single spike, equal to burst window from start of recording
0
index
0
2
20
sec
Single spike?
By your definition on the first page of text, to be included in a burst, interval must be less than BW.
Thus this is a single spike

## Slide 7
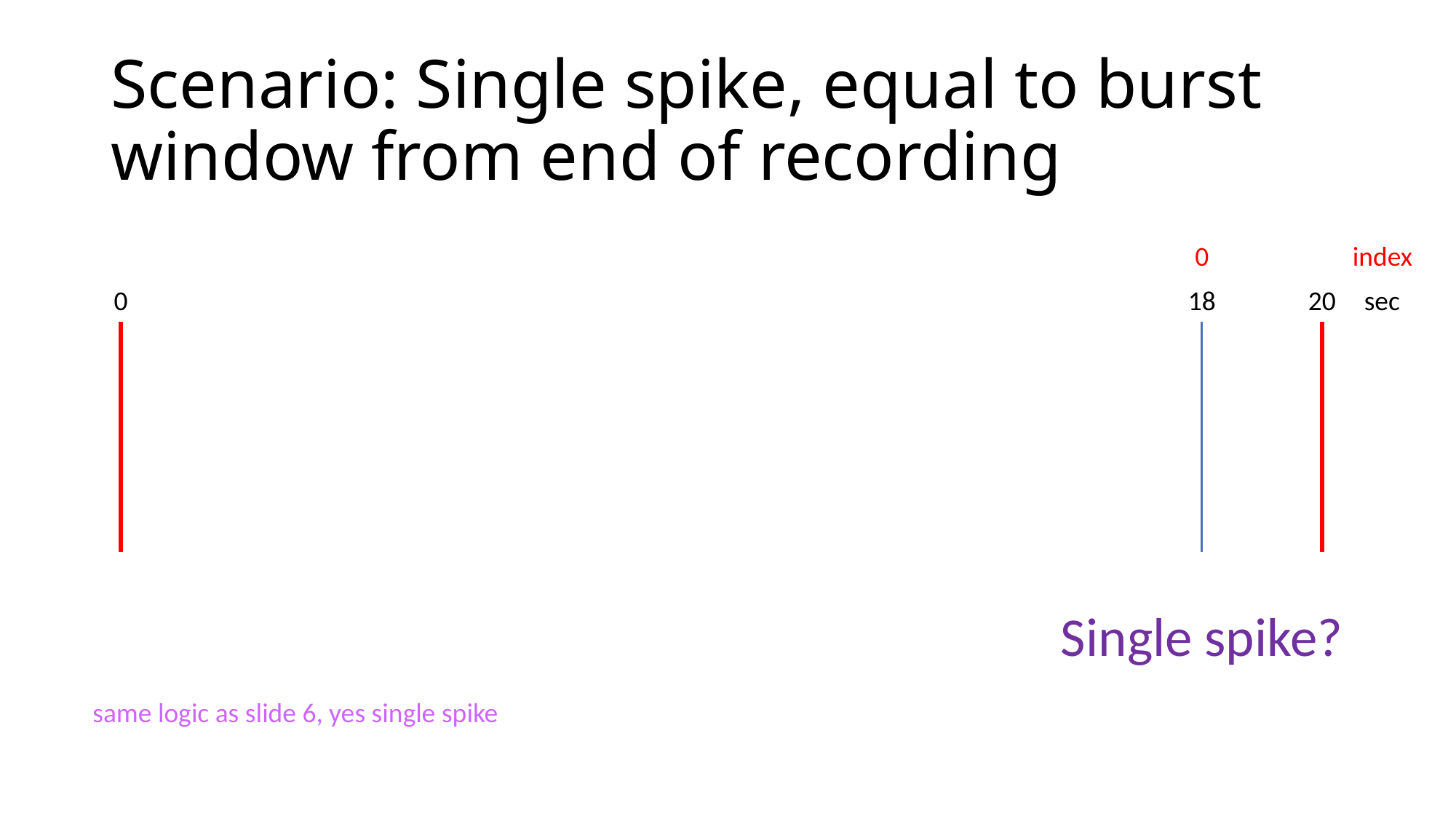

# Scenario: Single spike, equal to burst window from end of recording
0
index
0
18
20
sec
Single spike?
same logic as slide 6, yes single spike

## Slide 8
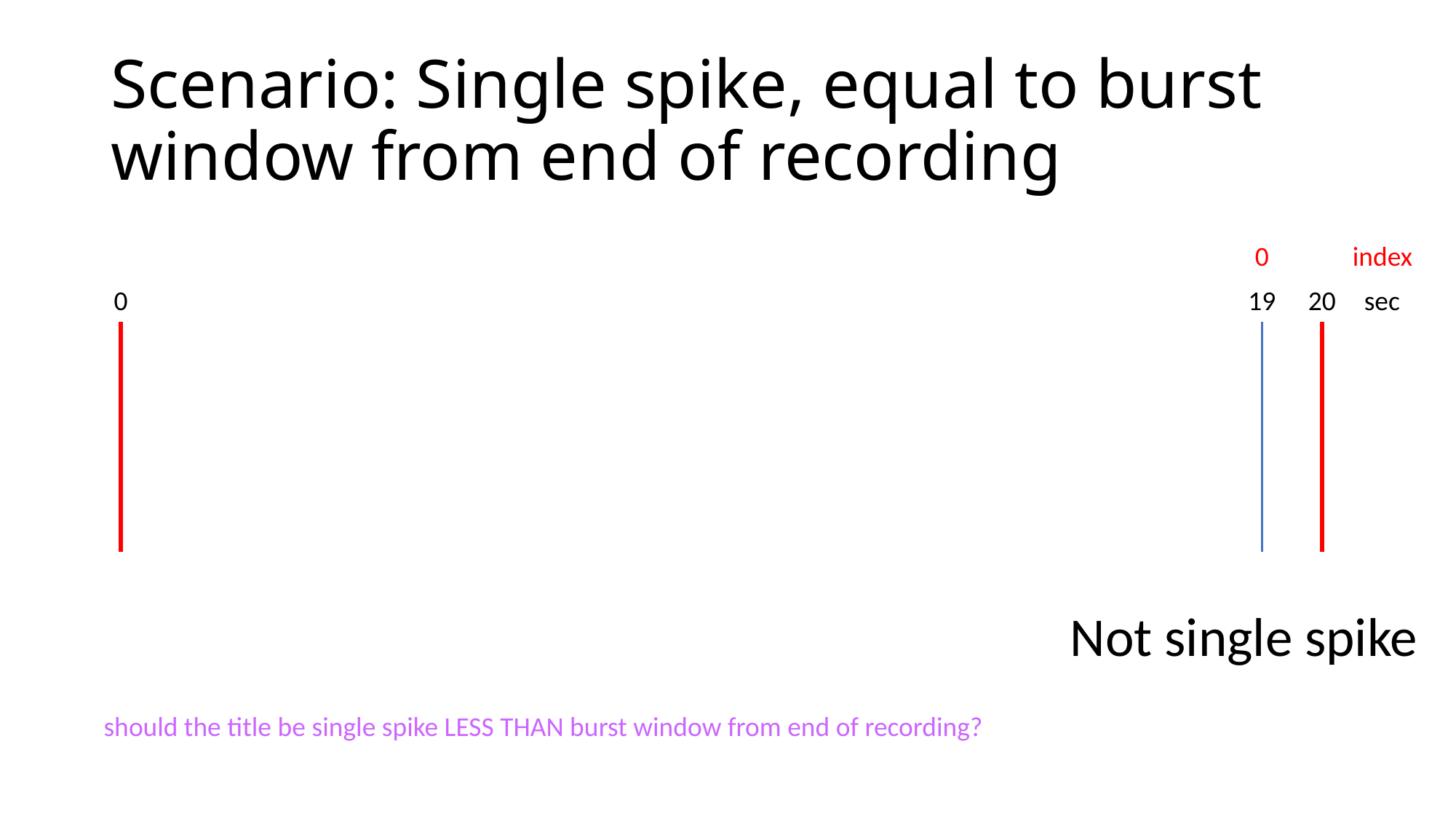

# Scenario: Single spike, equal to burst window from end of recording
0
index
0
19
20
sec
Not single spike
should the title be single spike LESS THAN burst window from end of recording?

## Slide 9
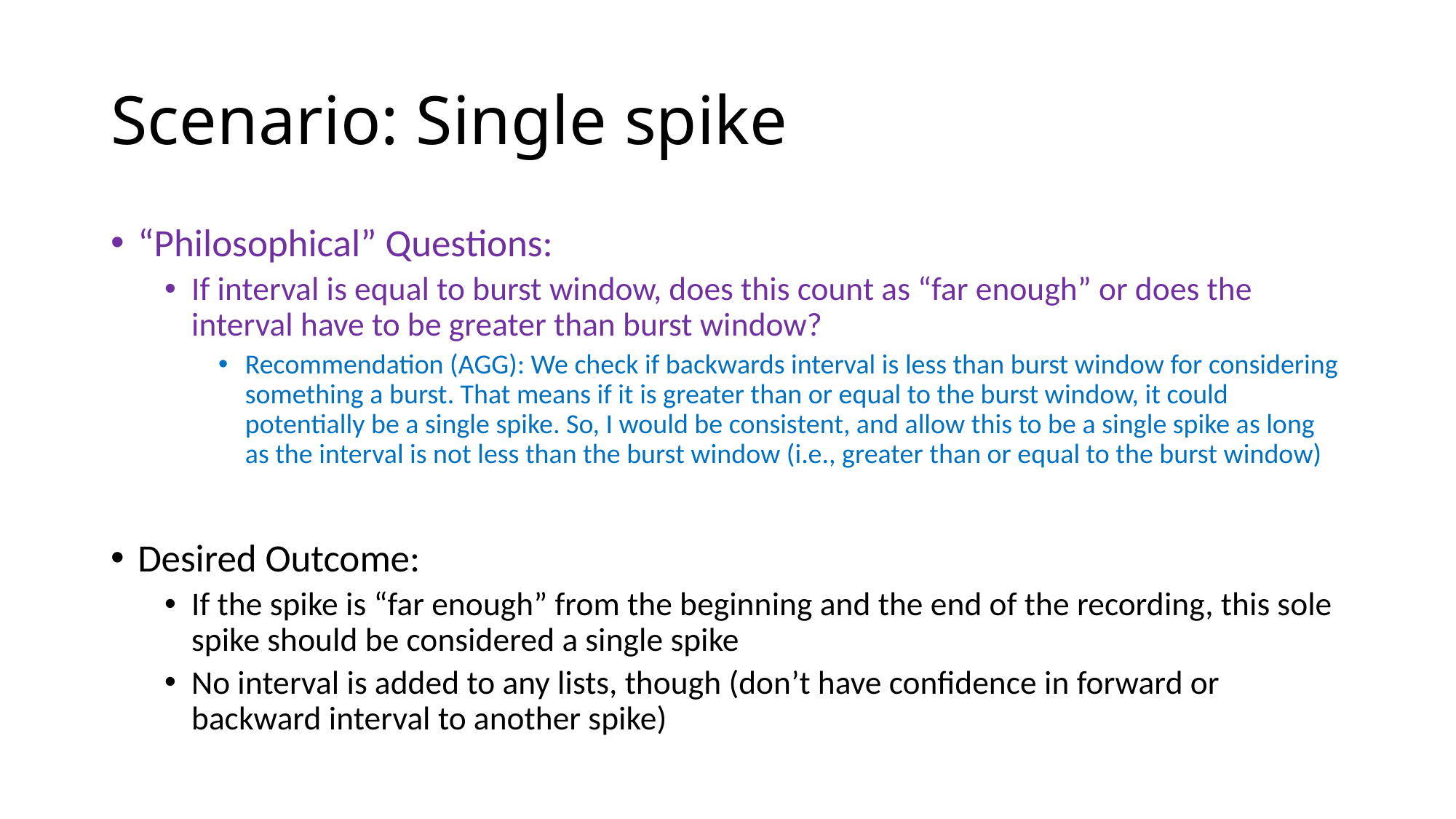

# Scenario: Single spike
“Philosophical” Questions:
If interval is equal to burst window, does this count as “far enough” or does the interval have to be greater than burst window?
Recommendation (AGG): We check if backwards interval is less than burst window for considering something a burst. That means if it is greater than or equal to the burst window, it could potentially be a single spike. So, I would be consistent, and allow this to be a single spike as long as the interval is not less than the burst window (i.e., greater than or equal to the burst window)
Desired Outcome:
If the spike is “far enough” from the beginning and the end of the recording, this sole spike should be considered a single spike
No interval is added to any lists, though (don’t have confidence in forward or backward interval to another spike)

## Slide 10
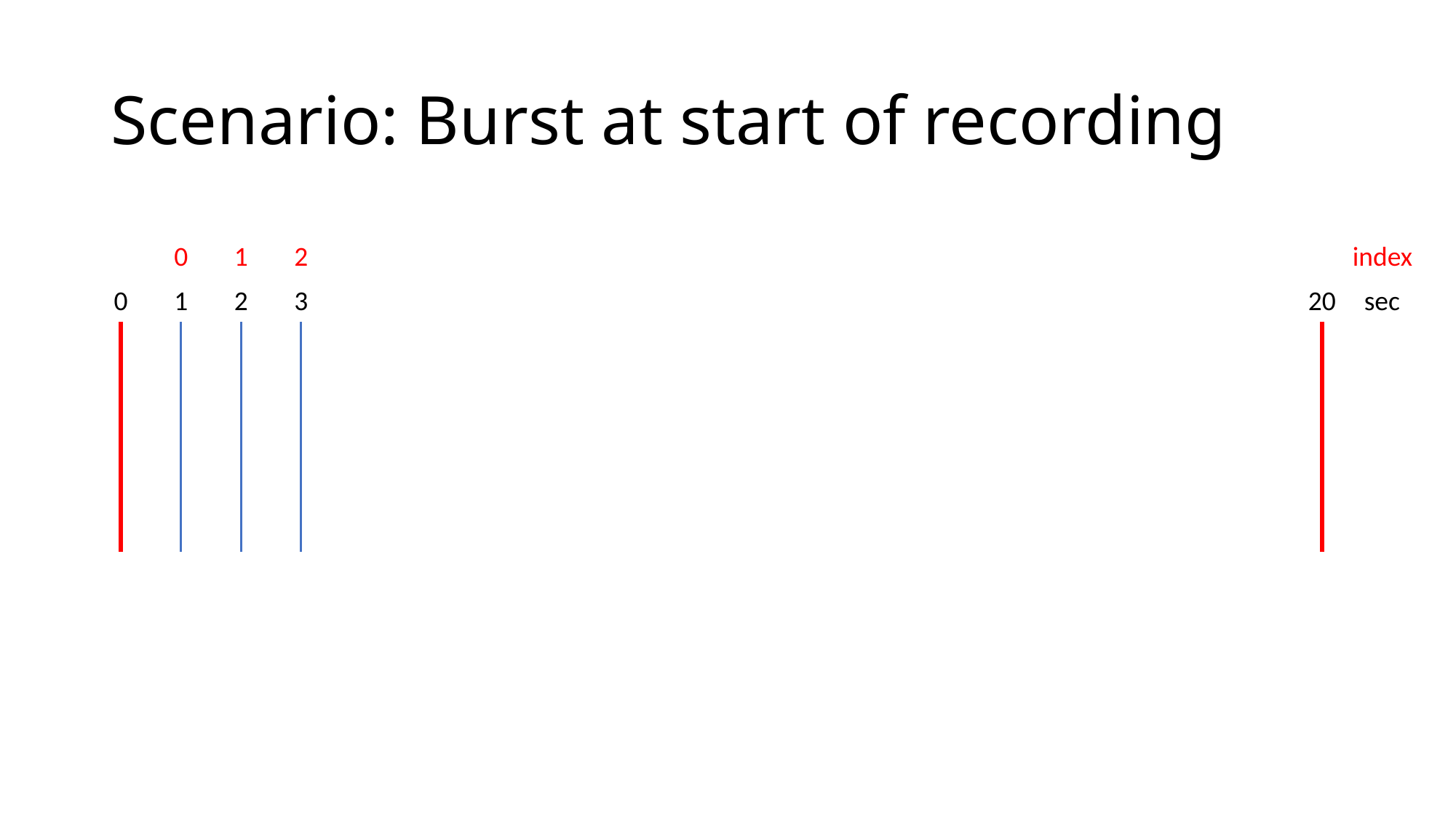

# Scenario: Burst at start of recording
0
1
2
index
0
1
2
3
20
sec

## Slide 11
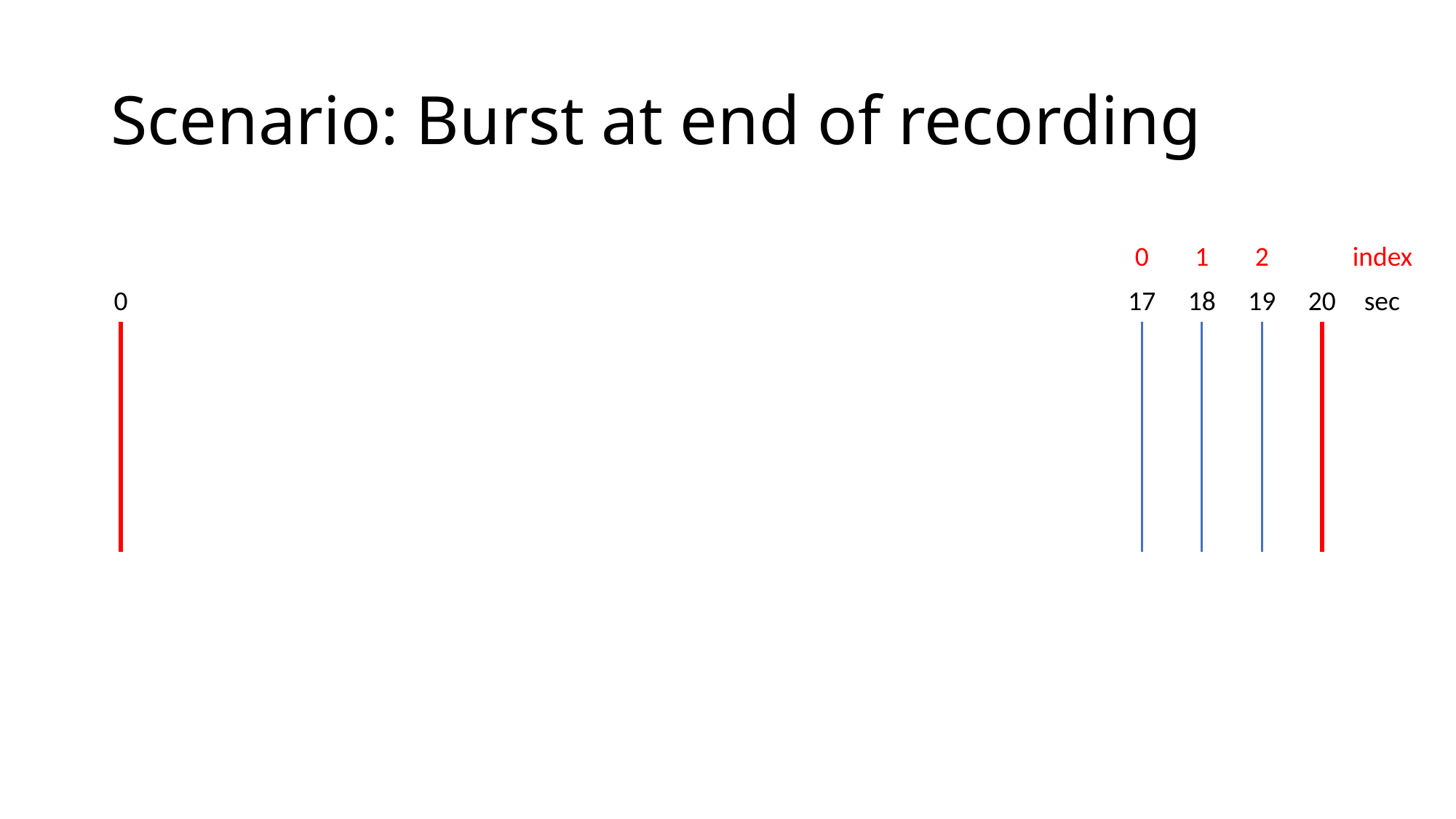

# Scenario: Burst at end of recording
0
1
2
index
0
17
18
19
20
sec

## Slide 12
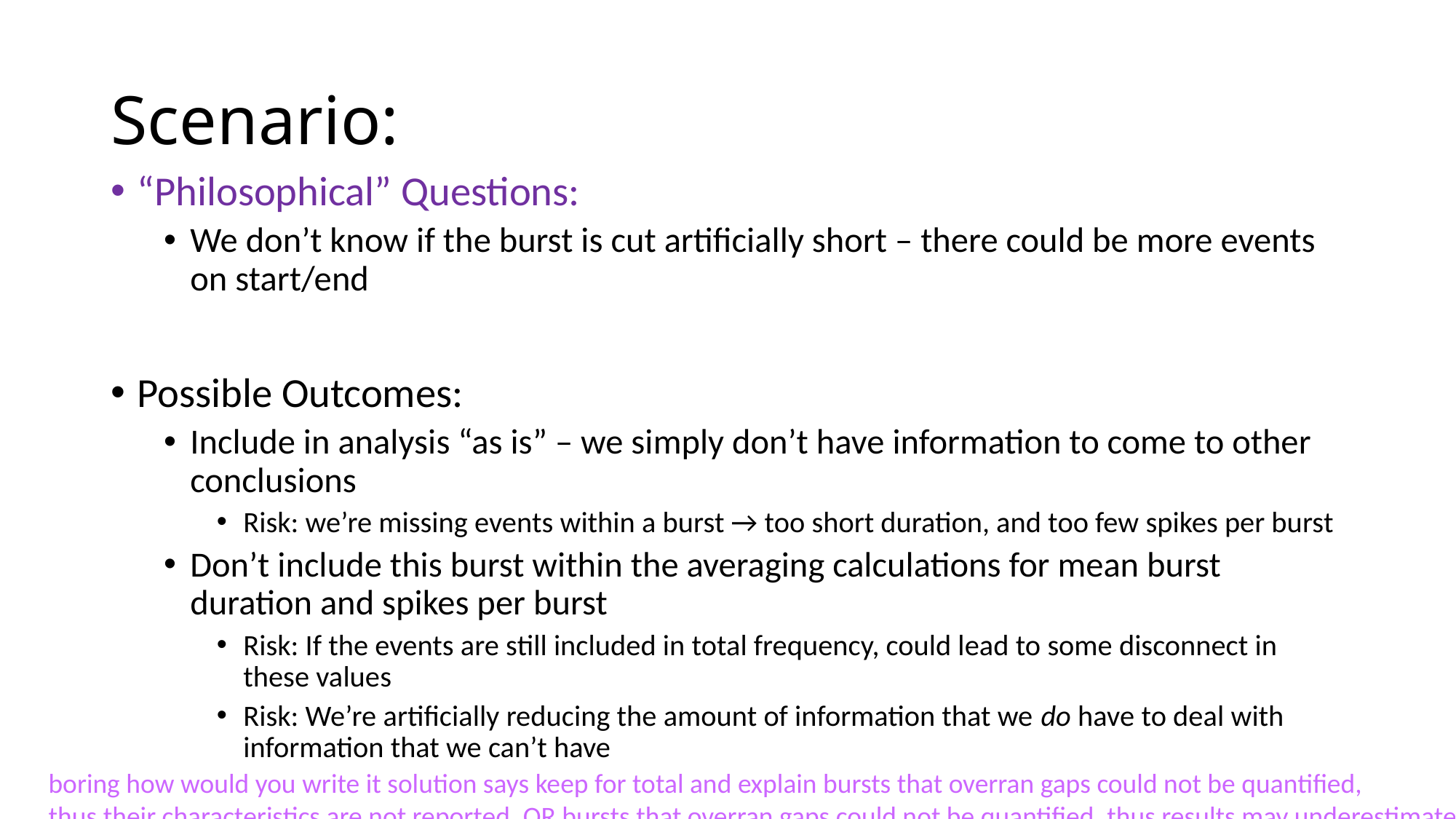

# Scenario:
“Philosophical” Questions:
We don’t know if the burst is cut artificially short – there could be more events on start/end
Possible Outcomes:
Include in analysis “as is” – we simply don’t have information to come to other conclusions
Risk: we’re missing events within a burst → too short duration, and too few spikes per burst
Don’t include this burst within the averaging calculations for mean burst duration and spikes per burst
Risk: If the events are still included in total frequency, could lead to some disconnect in these values
Risk: We’re artificially reducing the amount of information that we do have to deal with information that we can’t have
boring how would you write it solution says keep for total and explain bursts that overran gaps could not be quantified,
thus their characteristics are not reported. OR bursts that overran gaps could not be quantified, thus results may underestimate
the mean/median characteristics of bursts. Give estimate of percent of bursts affected (probably could calculate this)
Disparities between total frequency, which includes all detected events, and burst/single spike characteristics are attributable
to rejecting events near recording gaps needed to do quality control checks as these cannot be rigorously characterized as
either single spkes or bursts.

## Slide 13
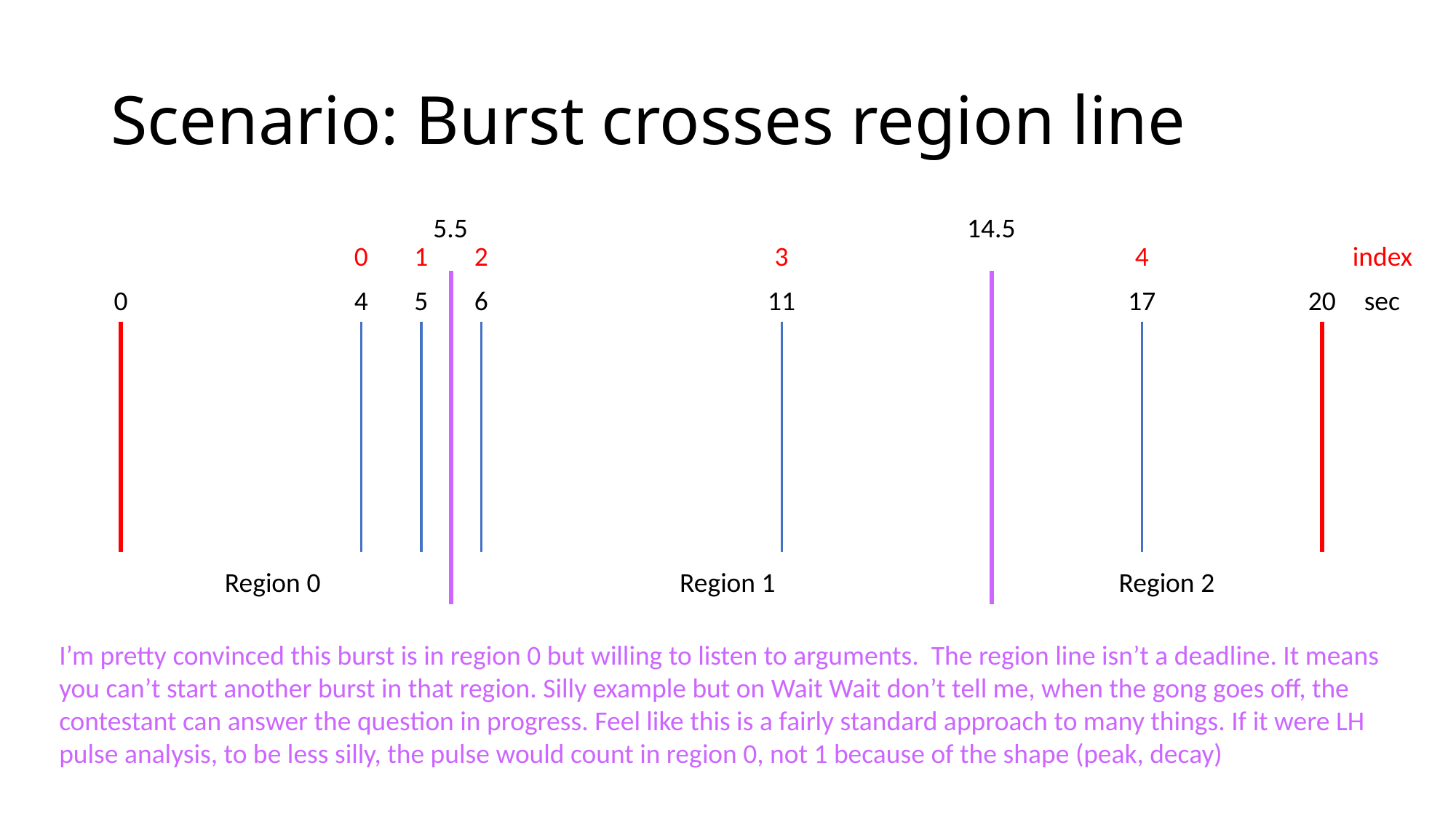

# Scenario: Burst crosses region line
5.5
14.5
0
1
2
3
4
index
0
4
5
6
11
17
20
sec
Region 0
Region 1
Region 2
I’m pretty convinced this burst is in region 0 but willing to listen to arguments. The region line isn’t a deadline. It means
you can’t start another burst in that region. Silly example but on Wait Wait don’t tell me, when the gong goes off, the
contestant can answer the question in progress. Feel like this is a fairly standard approach to many things. If it were LH
pulse analysis, to be less silly, the pulse would count in region 0, not 1 because of the shape (peak, decay)

## Slide 14
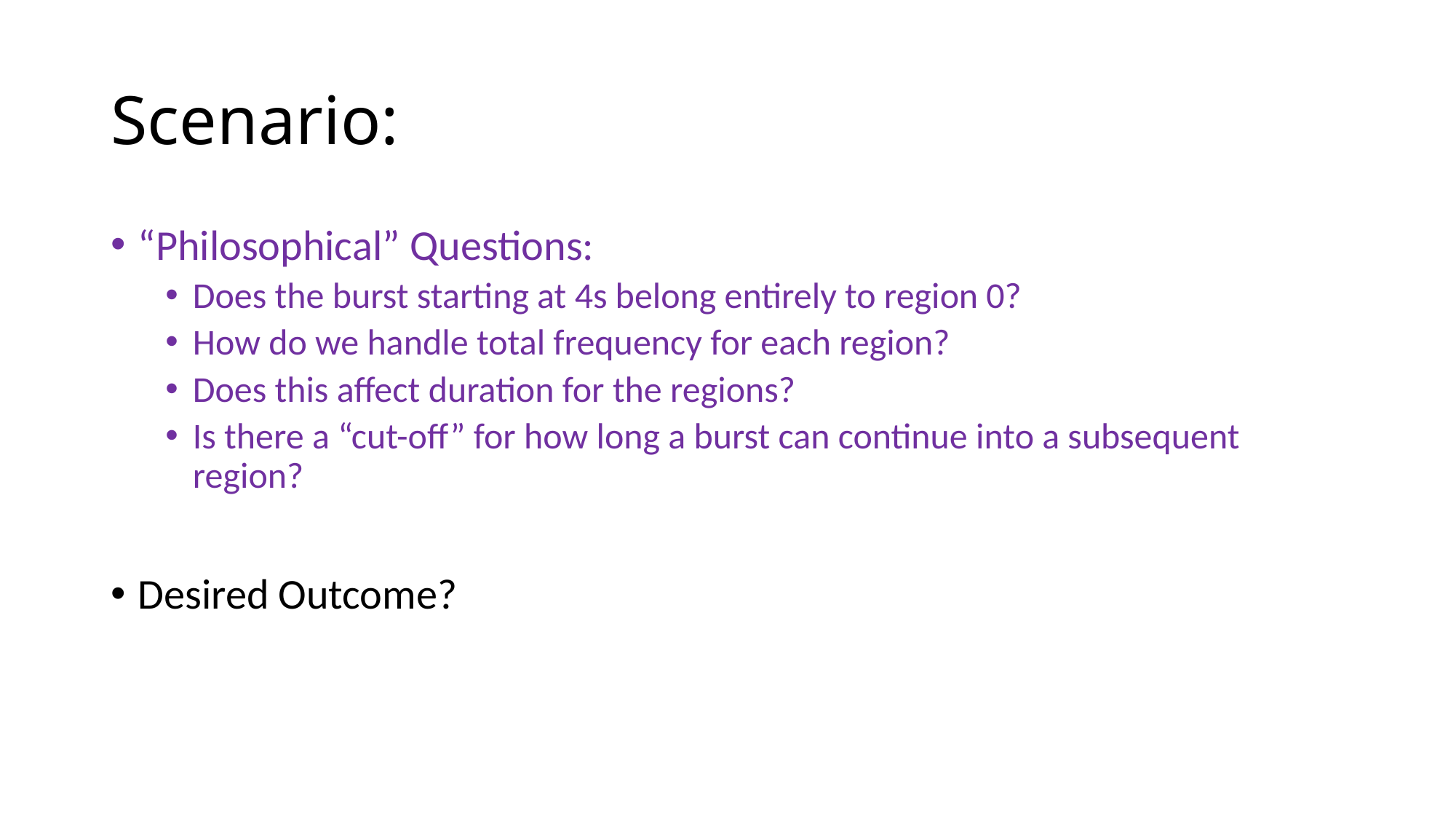

# Scenario:
“Philosophical” Questions:
Does the burst starting at 4s belong entirely to region 0?
How do we handle total frequency for each region?
Does this affect duration for the regions?
Is there a “cut-off” for how long a burst can continue into a subsequent region?
Desired Outcome?

## Slide 15
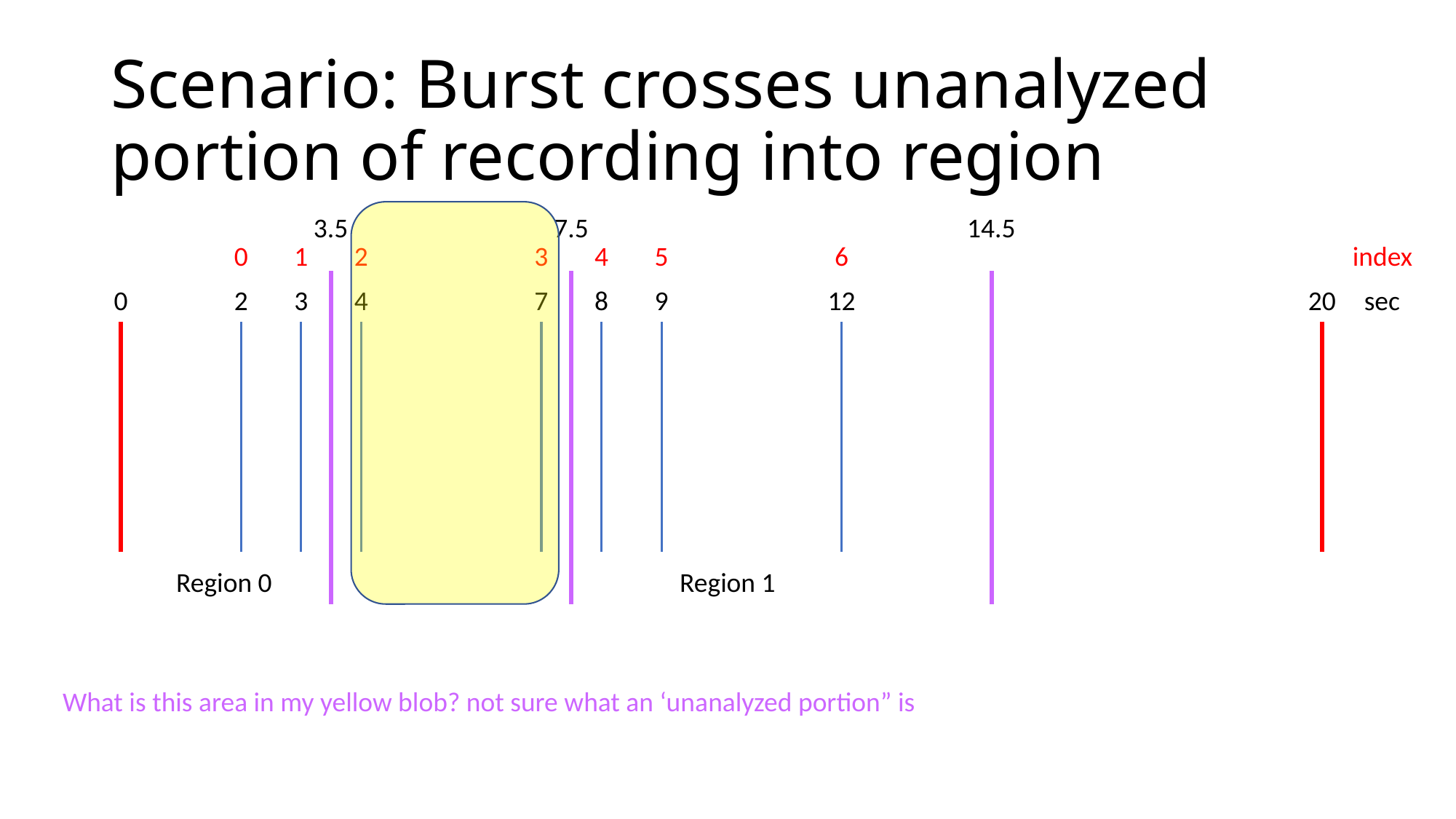

# Scenario: Burst crosses unanalyzed portion of recording into region
3.5
7.5
14.5
0
1
2
3
4
5
6
index
0
2
3
4
7
8
9
12
20
sec
Region 0
Region 1
What is this area in my yellow blob? not sure what an ‘unanalyzed portion” is

## Slide 16
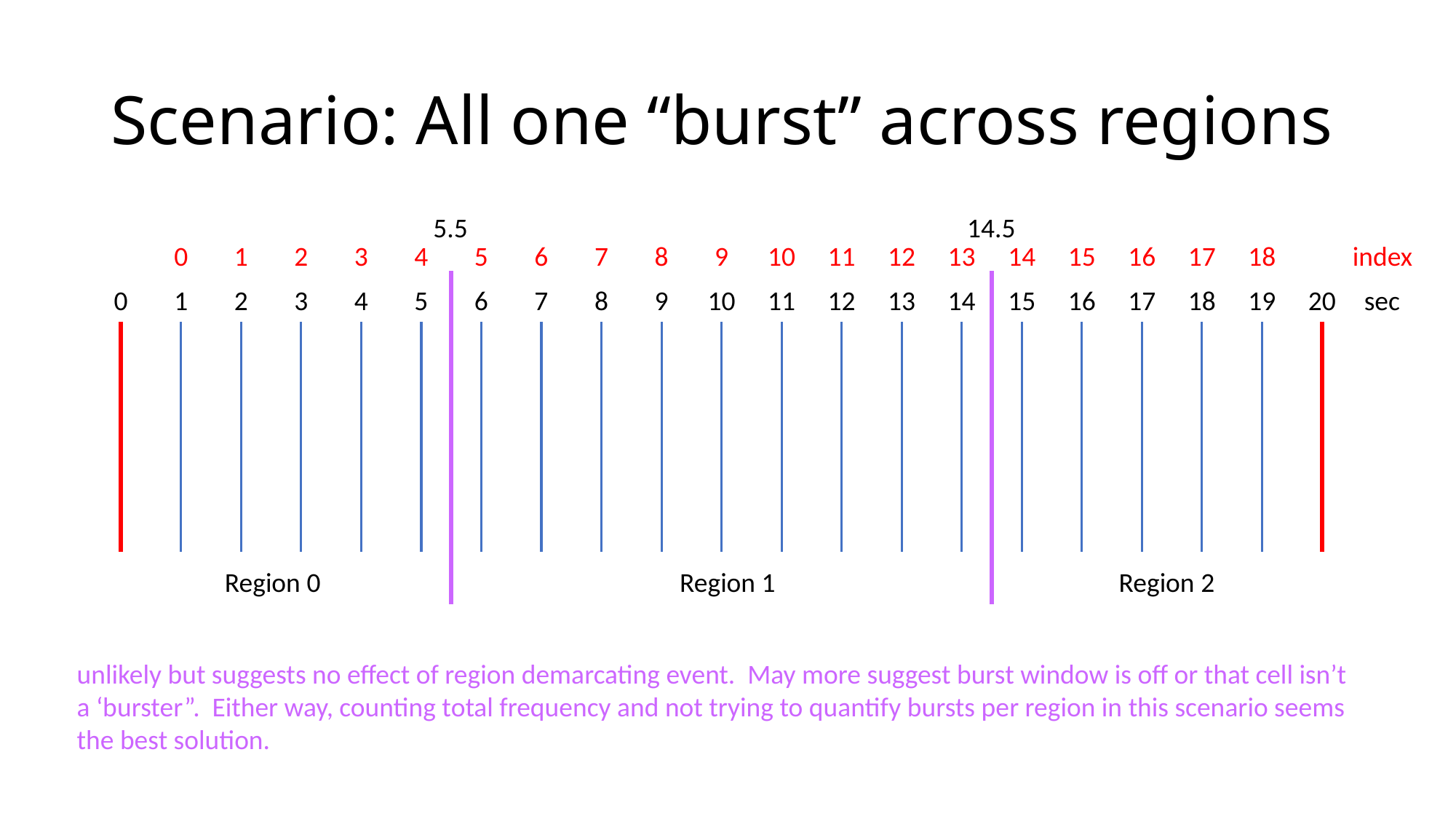

# Scenario: All one “burst” across regions
5.5
14.5
0
1
2
3
4
5
6
7
8
9
10
11
12
13
14
15
16
17
18
index
0
1
2
3
4
5
6
7
8
9
10
11
12
13
14
15
16
17
18
19
20
sec
Region 0
Region 1
Region 2
unlikely but suggests no effect of region demarcating event. May more suggest burst window is off or that cell isn’t
a ‘burster”. Either way, counting total frequency and not trying to quantify bursts per region in this scenario seems
the best solution.

## Slide 17
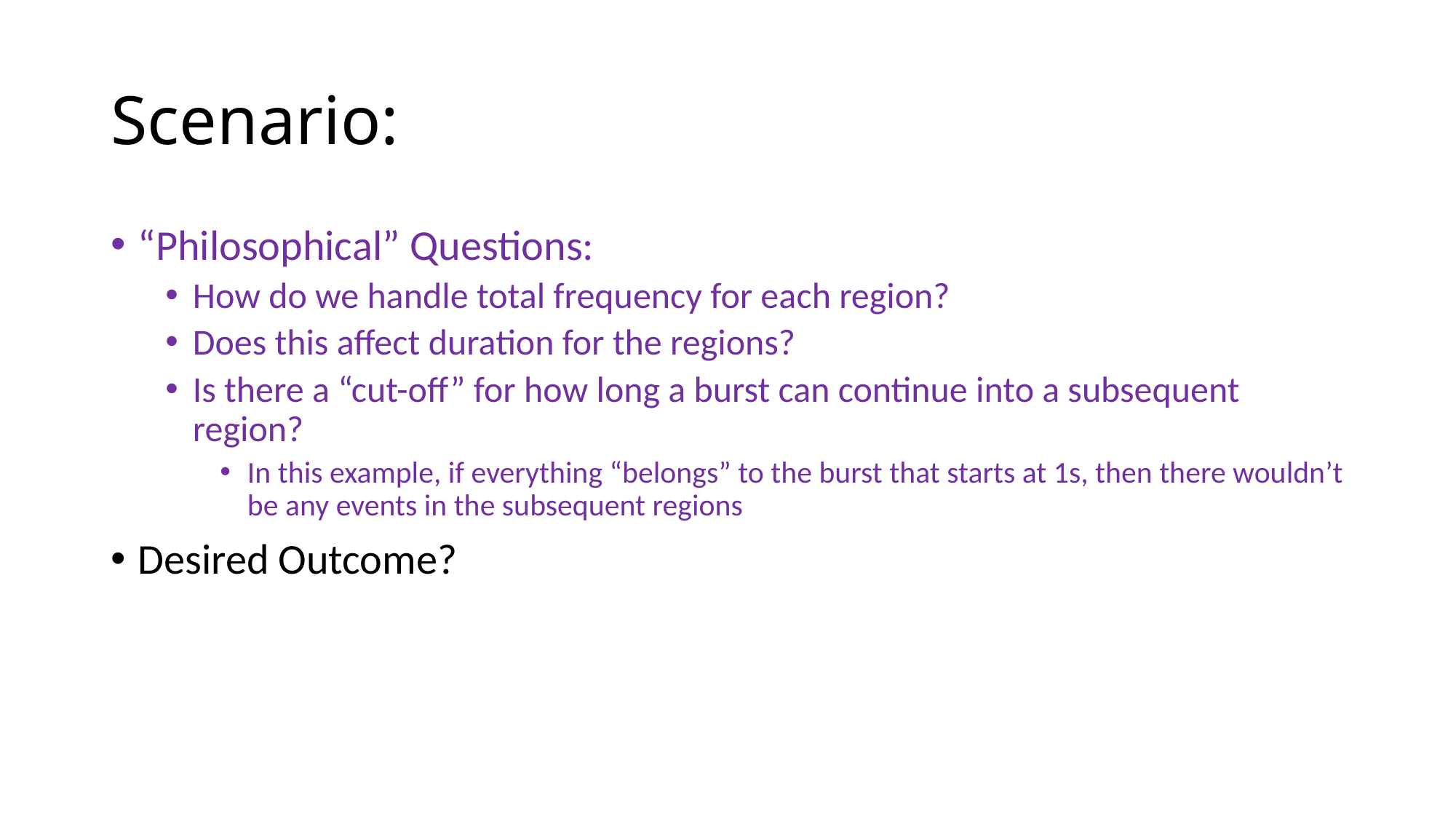

# Scenario:
“Philosophical” Questions:
How do we handle total frequency for each region?
Does this affect duration for the regions?
Is there a “cut-off” for how long a burst can continue into a subsequent region?
In this example, if everything “belongs” to the burst that starts at 1s, then there wouldn’t be any events in the subsequent regions
Desired Outcome?

## Slide 18
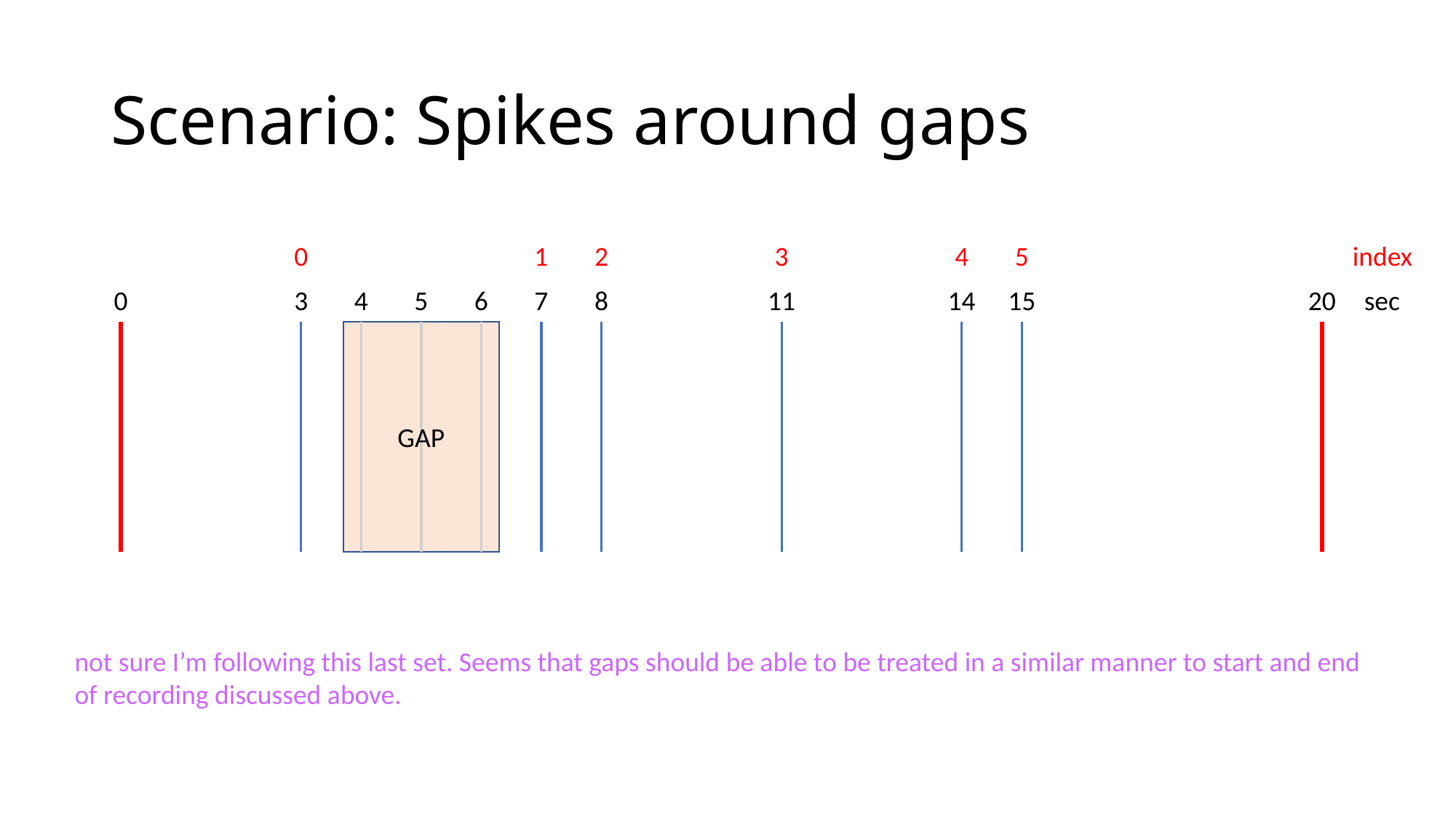

# Scenario: Spikes around gaps
0
1
2
3
4
5
index
0
3
4
5
6
7
8
11
14
15
20
sec
GAP
not sure I’m following this last set. Seems that gaps should be able to be treated in a similar manner to start and end
of recording discussed above.

## Slide 19
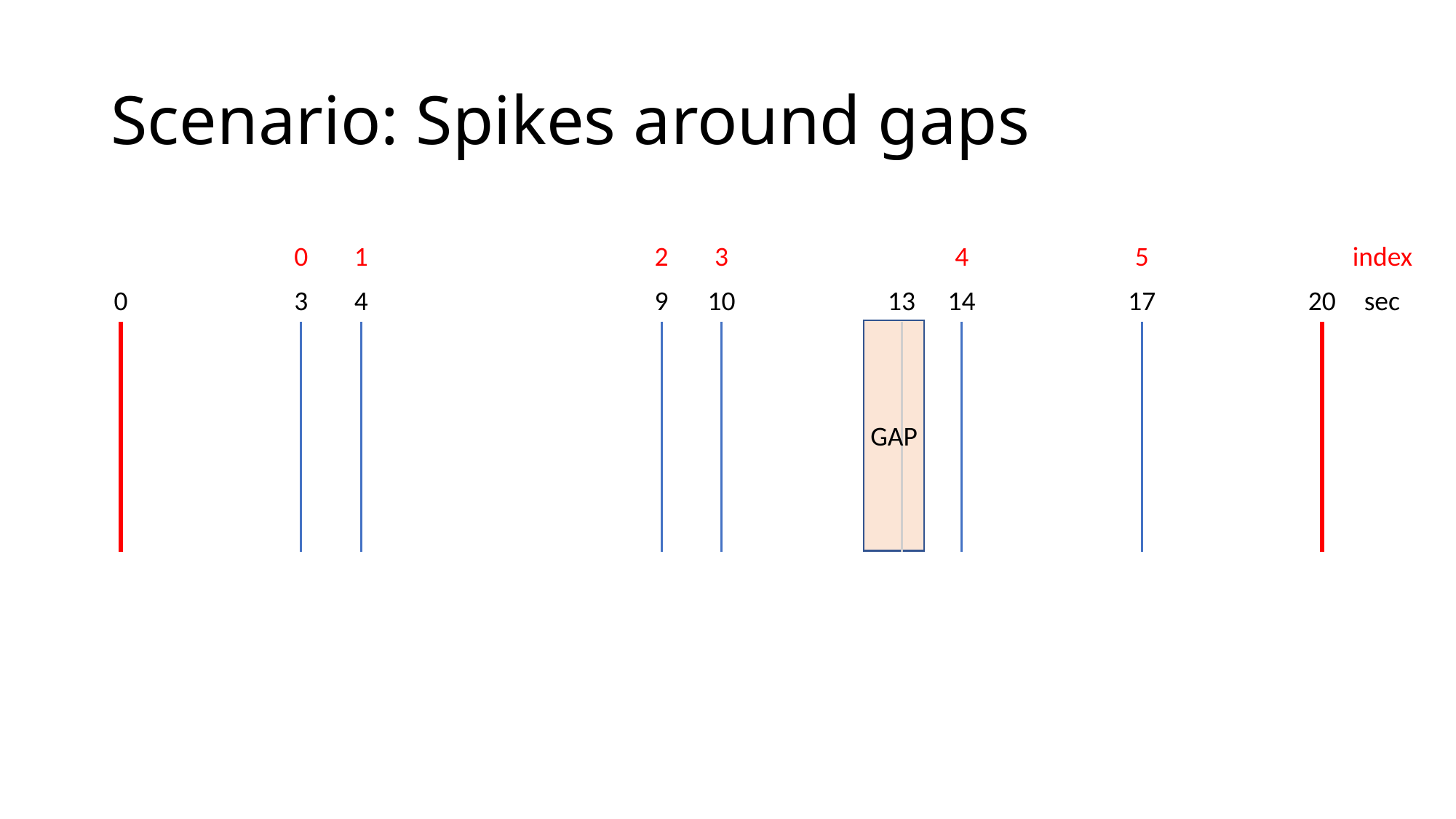

# Scenario: Spikes around gaps
0
1
2
3
4
5
index
0
3
4
9
10
13
14
17
20
sec
GAP

## Slide 20
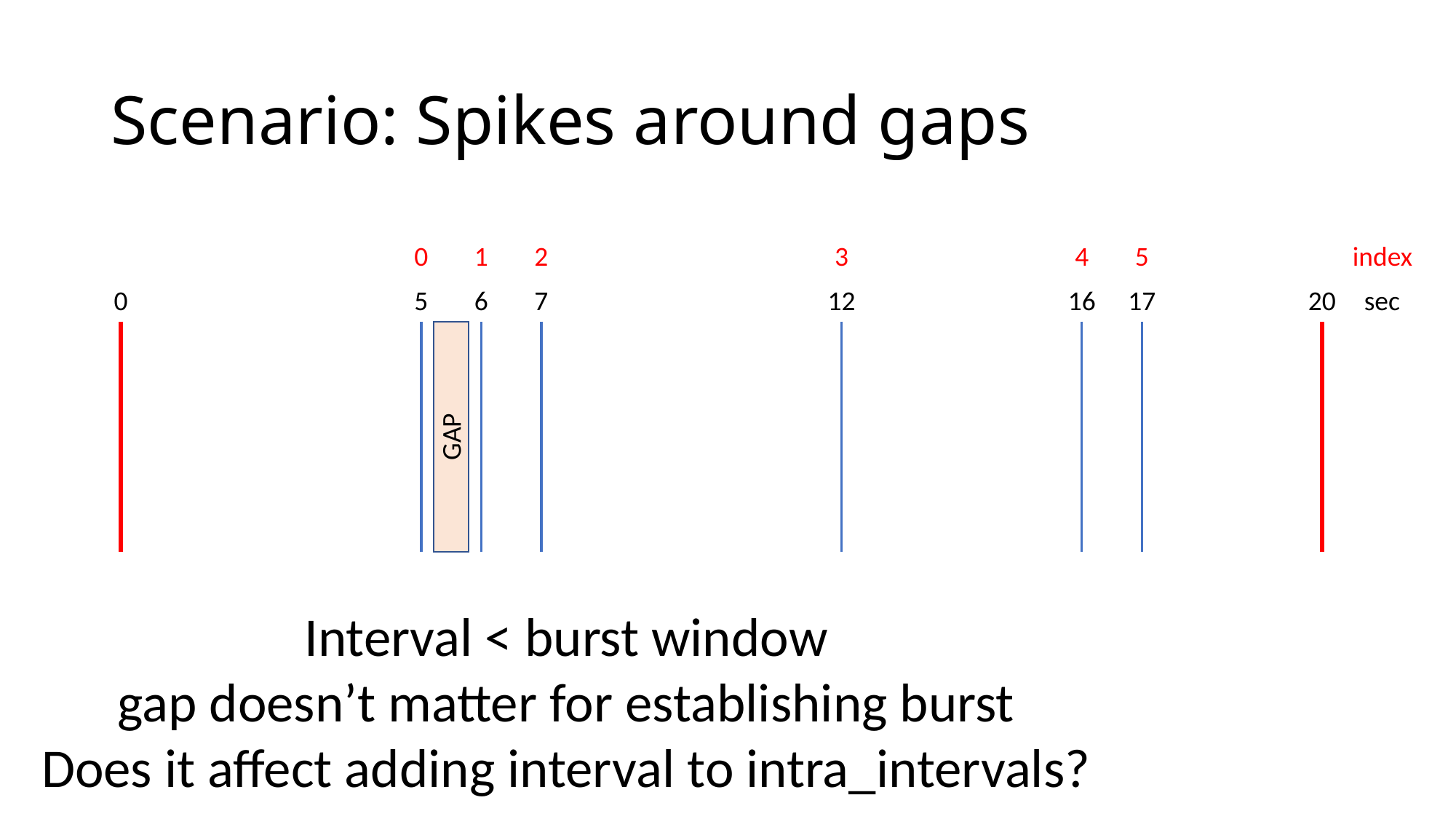

# Scenario: Spikes around gaps
0
1
2
3
4
5
index
0
5
6
7
12
16
17
20
sec
GAP
Interval < burst window
gap doesn’t matter for establishing burst
Does it affect adding interval to intra_intervals?
Interesting but will this be encountered? Seems that even with protocol editor handling the quality checks, gaps are 1.5 s
which is greater than a neuroscience-accepted ‘burst’ window. For most of our cells, if you plot number of bursts detected
vs burst window, there is a string of zeros until ~100 ms is reached

## Slide 21
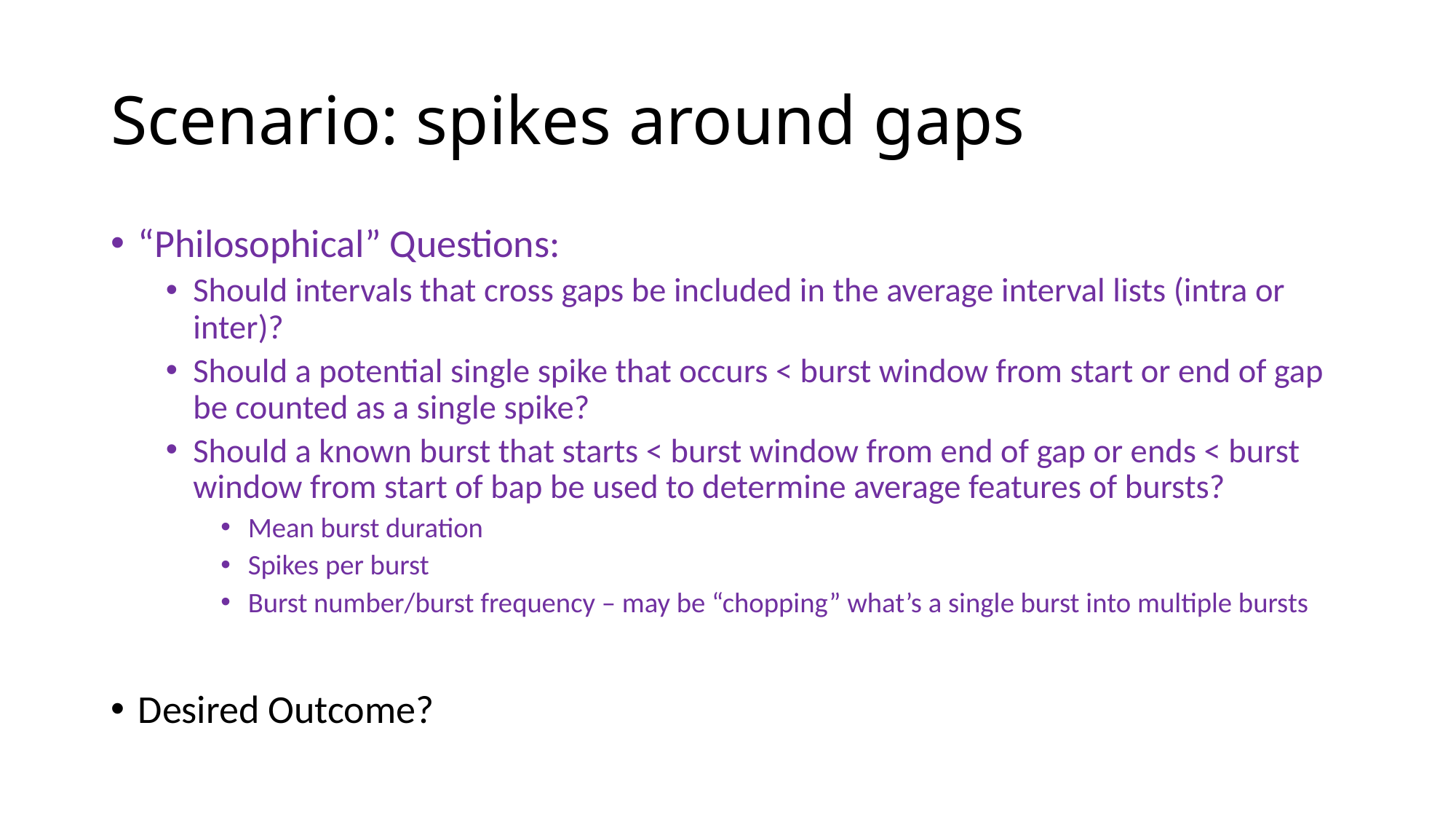

# Scenario: spikes around gaps
“Philosophical” Questions:
Should intervals that cross gaps be included in the average interval lists (intra or inter)?
Should a potential single spike that occurs < burst window from start or end of gap be counted as a single spike?
Should a known burst that starts < burst window from end of gap or ends < burst window from start of bap be used to determine average features of bursts?
Mean burst duration
Spikes per burst
Burst number/burst frequency – may be “chopping” what’s a single burst into multiple bursts
Desired Outcome?
