# Supplementary material for: Early-Life Resource Scarcity in Mice Does Not Alter Adult Corticosterone or Preovulatory Luteinizing Hormone Surge Responses to Acute Psychosocial Stress
Source: eNeuro. 2024 Jul 26;11(7):ENEURO.0125-24.2024. doi: 10.1523/ENEURO.0125-24.2024 (PMC11287788; doi:10.1523/ENEURO.0125-24.2024)
Supplement: Extended Data — Zip file of custom code for PSC detection and analysis, ffmpeg recording of dam behavior, and R analysis. Download Extended Data, ZIP file. [file eneuro-11-ENEURO.0125-24.2024-s002.zip › PSC-analysis/documentation/2022 July 7 td analysis install.docx]

2022 July 7

Td analysis Igor code install instructions

Access the folder containing the code either from a zip file or the repository. Place the folder in your documents folder. Create an alias or shortcut and copy it to the clipboard. Open the “Wavemetrics” folder in your documents folder. On this day we are using Igor Pro 9. Open the “Igor Pro 9 User Files” folder. Paste the alias/shortcut into the “Igor Procedures” folder located in the “Igor Pro 9 User Files” folder. Open Igor Pro 9. Check that the menu item “td analysis” appears as below:


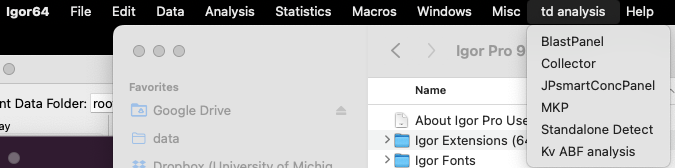


If so, you have successfully installed the code!
