# Supplementary material for: Early-Life Resource Scarcity in Mice Does Not Alter Adult Corticosterone or Preovulatory Luteinizing Hormone Surge Responses to Acute Psychosocial Stress
Source: eNeuro. 2024 Jul 26;11(7):ENEURO.0125-24.2024. doi: 10.1523/ENEURO.0125-24.2024 (PMC11287788; doi:10.1523/ENEURO.0125-24.2024)
Supplement: Extended Data — Zip file of custom code for PSC detection and analysis, ffmpeg recording of dam behavior, and R analysis. Download Extended Data, ZIP file. [file eneuro-11-ENEURO.0125-24.2024-s002.zip › PSC-analysis/documentation/AGG_procs/generalFunctions.docx]

# AGG: General Igor Functions

**Author:** *Amanda Gibson*

**Updated:** *April 13, 2022*

- [General Waves](#general-waves)
  - [getWaveNameAndDFR](#getwavenameanddfr)
  - [PrintAllWaveNames](#printallwavenames)
- [Numeric Waves](#numeric-waves)
  - [calcWaveAvg](#calcwaveavg)
  - [getPercentileFromDist](#getpercentilefromdist)
  - [fWaveAverage_waveRefs](#fwaveaverage_waverefs)
- [Text Waves](#text-waves)
  - [removeBlankCells](#removeblankcells)
  - [getUniqueGroups](#getuniquegroups)
  - [getTextWaveSubsetFromGroup](#gettextwavesubsetfromgroup)
- [Numeric Variables](#numeric-variables)
  - [avgTwoVals](#avgtwovals)

# General Waves

## getWaveNameAndDFR

### Parameters

- thisWave: a wave reference

### Returns

- waveN: string of wave name
- waveDFR: data folder reference where wave is stored

### Purpose

From a wave reference, return the name of the wave and where the wave is stored. This is helpful if using wave references within a function and you need to name or save other items in relation to that wave

### File

[AGG_generalFuncs.ipf](../../AGG_procs/general/AGG_generalFuncs.ipf)

## PrintAllWaveNames

### Parameters

- dfr: data folder reference from which to print waves

### Purpose

Prints to the command window the names of all waves within specified folder (dfr)

### File

[AGG_generalFuncs.ipf](../../AGG_procs/general/AGG_generalFuncs.ipf)

# Numeric Waves

## calcWaveAvg

### Parameters

- thisWave: a numeric wave reference

### Returns

- thisAvg: the average of thisWave

### Purpose

Uses waveStats to get the average of wave. In contrast to mean(), this ignores NaNs within the wave when calculating the average

### File

[AGG_generalFuncs.ipf](../../AGG_procs/general/AGG_generalFuncs.ipf)

## getPercentileFromDist

### Parameters

- distWave: A numeric distribution wave, such as that calculated by event detection
- percentile: A variable for the percentile at which you want the value of distWave

### Returns

- valAtPercentile: The value of distWave at the indicated percentile

### File

[AGG_generalFuncs.ipf](../../AGG_procs/general/AGG_generalFuncs.ipf)

## fWaveAverage_waveRefs

### Parameters

- yWavesToAvg: Wave of wave references for the y waves to average
- ErrorTypeStr: Type of error wave (if any) to calculate
  - “none”
  - “CI” - confidence interval
  - “stdDev” - standard deviation
  - “SEM” - standard error of the mean
- avgWave: Wave reference for the average wave
- [optional] xWavesToAvg: wave of wave references for the x waves to average
- [optional] ErrorInterval: number for the error interval
  - if CI: value from 0-100. Default is 95 if not provided
  - if stdDev: number of standard deviations. Default is 2 if not provided
  - ignored for no error type or for SEM
- [optional] errorWave: Wave reference for the error wave. If not provided, and if an error type is indicated, will be saved in the same data folder as avgWave with “_error” appended to the end of the wave name

### Global changes

- avgWave: Updated with average
- errorWave: if error type is specified, made if not provided (“[avgWaveName]_error”), and updated if provided

### Purpose

Uses the fWaveAverage function that is part of the <Waves Averages> utility to calculate the average trace for a set of waves. Options are also provided for different types of error waves that can be generated.

Possible use cases in the lab could include generating * an average event trace across cells or groups * an average current trace in response to a particular voltage input

Often, this will be done with traces that are plotted in a graph. For graphs that are stand-alone and the current top graph, you can use Analyis -> Packages -> Average Waves, which also relies on fWaveAverage.

For graphs that are within a panel, you should use the function [averageWavesInGraph](./plots.md##averageWavesInGraph) which will work for graphs that are within subpanels

NOTE: this has not yet been tested with xWaves

# Text Waves

## removeBlankCells

### Parameters

- fullWave: a text wave reference

### Global Changes

- modifies fullWave directly

### Purpose

Remove blank cells from a wave

### File

[AGG_generalFuncs.ipf](/AGG_procs/general/AGG_generalFuncs.ipf)

## getUniqueGroups

### Parameters

- groupWave: a (text) wave with group information
- [optional] storageDF: data folder reference where the uniqueGroups wave should be stored
  - defaults to store in the data folder of groupWave

### Returns

- uniqueGroups: a text wave with only the unique treatment groups

### Global Changes

- uniqueGroups is created or overwritten within either the specified storageDF or in the same data folder as groupWave

### Purpose

To the get the unique groups from a list of groups (such as which group each cell belongs to). This can then be used for creating output graphs, or to subset a different wave based on which group the values correspond to, such as with [getTextWaveSubsetFromGroup](#X5b7e0c6b051dc81d32b077ba8551442061fdadf)

### File

[AGG_generalFuncs.ipf](/AGG_procs/general/AGG_generalFuncs.ipf)

## getTextWaveSubsetFromGroup

### Parameters

- groupWave: a text wave containing the group designations
- waveToSubset: a text wave from which you want to receive the subset related to a specific group
- [optional] groupNum: a variable for the index of the group to use when subsetting the wave
- [optional] groupName: a string for the name of the group to use when subsetting the wave
  - if groupName is provided, groupNum is ignored
  - groupName is a safer option than groupNum

### Returns

- subWave: a subset of waveToSubset with the name [name of waveToSubset]_[groupName]

### Global Changes

- subWave is created within the data folder that waveToSubset is stored

### Purpose

Use a text wave that contains group designations for each item to obtain a subset of a different text wave. For example, if you have a wave a cell names and a wave of treatment groups for each cell name, you could get a wave of cell names that belong to a particular treatment group

### File

[AGG_generalFuncs.ipf](/AGG_procs/general/AGG_generalFuncs.ipf)

# Numeric Variables

## avgTwoVals

### Parameters

- val1: numeric variable 1
- val2: numeric variable 2

### Returns

- avg: the average of val1 and val2 if they both are normal numbers, otherwise NaN is returned

### File

[AGG_generalFuncs.ipf](/AGG_procs/general/AGG_generalFuncs.ipf)
