# Supplementary material for: Early-Life Resource Scarcity in Mice Does Not Alter Adult Corticosterone or Preovulatory Luteinizing Hormone Surge Responses to Acute Psychosocial Stress
Source: eNeuro. 2024 Jul 26;11(7):ENEURO.0125-24.2024. doi: 10.1523/ENEURO.0125-24.2024 (PMC11287788; doi:10.1523/ENEURO.0125-24.2024)
Supplement: Extended Data — Zip file of custom code for PSC detection and analysis, ffmpeg recording of dam behavior, and R analysis. Download Extended Data, ZIP file. [file eneuro-11-ENEURO.0125-24.2024-s002.zip › PSC-analysis/documentation/AGG_procs/plots.docx]

# AGG: Plot Functions

**Author:** *Amanda Gibson*

**Updated:** *April 13, 2022*

- [Plots](#plots)
  - [clearDisplay](#cleardisplay)
  - [plotValByTime](#plotvalbytime)
  - [averageWavesInGraph](#averagewavesingraph)
  - [removeAxes](#removeaxes)
- [Group Plots](#group-plots)
  - [plotGroupData](#plotgroupdata)
  - [getGroupMean](#getgroupmean)
  - [getGroupSEM](#getgroupsem)
  - [getGroupCI](#getgroupci)

# Plots

## clearDisplay

### Parameters

- target: string for graph name; full name, including #

### Purpose

Removes all traces from a graph, including boxplots and violin plots

### File

[AGG_plots.ipf](../../AGG_procs/plots/AGG_plots.ipf)

## plotValByTime

### Parameters

- valWave: a numerical wave reference - y value
- timeWave: a numerical wave reference for the time - x value
- [optional] paramLabel: a string to label the y axis
- [optional] displayHost: a string with the name of the dispaly where the graph should be plotted
  - if not provided, default is to make a new display

### Global Changes

- Appends traces either to the provided displayHost or creates a new display
- Sets the scale of timeWave to "dat"

### Purpose

This plots y values over time. This is useful for plotting how values change across different series.

The resulting plot has markers at each time/y-val pair, and a line connecting each marker

### File

[AGG_plots.ipf](../../AGG_procs/plots/AGG_plots.ipf)

## averageWavesInGraph

### Parameters

- ErrorTypeStr: Type of error wave (if any) to calculate
  - “none”
  - “CI” - confidence interval
  - “stdDev” - standard deviation
  - “SEM” - standard error of the mean
- avgWave: Wave reference for the average wave
- [optional] displayName: string of full graph name, including panel + # if it’s a subwindow
  - default is top window
- [optional] addToGraph: variable to indicate if the average wave (and error wave, if applicable) should be added to the graph
  - 1 = add
  - 0 = don’t add
- [optional] ErrorInterval: number for the error interval
  - if CI: value from 0-100. Default is 95 if not provided
  - if stdDev: number of standard deviations. Default is 2 if not provided
  - ignored for no error type or for SEM
- [optional] errorWave: Wave reference for the error wave. If not provided, and if an error type is indicated, will be saved in the same data folder as avgWave with “_error” appended to the end of the wave name

### Global changes

- avgWave: Updated with average
- errorWave: if error type is specified, made if not provided (“[avgWaveName]_error”), and updated if provided
- avgWave and errorWave are added to the graph if indicated by addToGraph

### Purpose

Uses the [fWaveAverage_waveRefs](/documentation/AGG_procs/generalFunctions.md##%60fWaveAverage_waveRefs%60) and underlying fWaveAverage function that is part of the <Waves Averages> utility to calculate the average trace for waves in either top graph or specified graph. Options are also provided for different types of error waves that can be generated. This will work with graphs that are within panels, in contrast to package utility

### File

[AGG_plots.ipf](../../AGG_procs/plots/AGG_plots.ipf)

## removeAxes

### Parameters

- graphName: [optional] string for specific graph from which you want to remove axes

### Global Changes

- Removes the axes and labels from either the active graph or graph specified by graphName

### Purpose

- Remove axes, useful for ephys traces for use in figures
- Called by removeAxesProc within save panel

### File

[AGG_plots.ipf](../../AGG_procs/plots/AGG_plots.ipf)

# Group Plots

These functions use the function [getUniqueGroups](/documentation/AGG_procs/generalFunctions.md##getUniqueGroups) to get the groups that will be plotted

## plotGroupData

### Parameters

- groupWave: A text wave with the group data
- numWave: Numeric wave with the y values to be plotted. Should be the same length as groupWave
- [optional] storageDF: data folder where the dataWave wave should be stored
  - default is data folder for the numWave
  - Not currently used for uniqueWaves storage (defaults to groupWave DF within uniqueWaves function), but could be changed to do so
- [optional] displayHost: name of the graph window where the plot should be created. If not provided, creates a display with the name groupData
- [optional] yLabel: Label for the y-axis

### Global changes

- Clears displayHost or groupData if they already have plotted data
- Creates or overwrites the uniqueGroups wave
  - data folder is default of the getUniqueGroups function
- Creates or overwrites the dataWave in the storageDF data folder. This is a multi-dimensional wave with the data from numWave split into different columns by group
- Creates or overwrites meanWave and SEMWave in the data folder of dataWave
- Adds individual data points and mean + SEM to the displayHost

### Purpose

To graph the individual values and mean + SEM of the values in numWave based on the group specified in groupWave.

This is achieved by creating a dataWave with the values for each group in a different column.

The dataWave is then plotted as a box plot versus the uniqueGroups. This wave has to be plotted first before the mean data can be added. There are some oddities about the ordering with categorical data on Igor graphs. There are some other ways to do this, such as with the /CATL label, but these are more challenging to work with, especially if trying to recreate the graph.

The lines of the boxplot are removed from the graph so that it is just the individual data points; all of which are plotted at the same size.

The group means are calculated with [getGroupMean](#X490e3f195f41c8ec97b4062c6472fce40833a3d) function and the SEMs with the [getGroupSEM](#Xdf6429e89bb9198ea1d61f060540e2b171d1bd9) function. The meanWave is also plotted versus uniqueGroups * When plotting meanWave you have to specify which row to plot and which columns * meanWave is generated by per-column wave statistics, so the average was pulled out and the row is labeled, so it is specified as %avg * SEMwave is added as error bars

### File

[AGG_groupPlotOutput.ipf](../../AGG_procs/plots/AGG_groupPlotOutput.ipf)

## getGroupMean

### Parameters

- dataWave: multi-column wave with numeric data, split into columns by group

### Returns

- meanWave: A multi-column wave with the average for each column (group)

### Global Changes

- Creates or overwrites meanWave in the same data folder as the dataWave
- Creates or overwrites M_waveStats when doing per-column statistics

### Purpose

Get the average for each group. Dimension label is %avg

### File

[AGG_groupPlotOutput.ipf](../../AGG_procs/plots/AGG_groupPlotOutput.ipf)

## getGroupSEM

### Parameters

- dataWave: multi-column wave with numeric data, split into columns by group

### Returns

- meanWave: A multi-column wave with the SEM for each column (group)

### Global Changes

- Creates or overwrites SEMWave in the same data folder as the dataWave
- Creates or overwrites M_waveStats when doing per-column statistics

### Purpose

Get the standard error of the mean for each group. Dimension label is %SEM

### File

[AGG_groupPlotOutput.ipf](../../AGG_procs/plots/AGG_groupPlotOutput.ipf)

## getGroupCI

### Parameters

- dataWave: multi-column wave with numeric data, split into columns by group

### Returns

- CIWave: A multi-column wave with the 95% CI for each column (group). Row 0: below mean, Row 1: above mean

### Global Changes

- Creates or overwrites CIWave in the same data folder as the dataWave
- Creates or overwrites M_waveStats when doing per-column statistics

### Purpose

Uses the critical value of Student T distribution for alpha = 0.05. Add /ALPH=val and an optional parameter for alphaVal if want a different confidence interval than 95%.

### File

[AGG_groupPlotOutput.ipf](../../AGG_procs/plots/AGG_groupPlotOutput.ipf)
