# Supplementary material for: Early-Life Resource Scarcity in Mice Does Not Alter Adult Corticosterone or Preovulatory Luteinizing Hormone Surge Responses to Acute Psychosocial Stress
Source: eNeuro. 2024 Jul 26;11(7):ENEURO.0125-24.2024. doi: 10.1523/ENEURO.0125-24.2024 (PMC11287788; doi:10.1523/ENEURO.0125-24.2024)
Supplement: Extended Data — Zip file of custom code for PSC detection and analysis, ffmpeg recording of dam behavior, and R analysis. Download Extended Data, ZIP file. [file eneuro-11-ENEURO.0125-24.2024-s002.zip › PSC-analysis/documentation/AGG_procs/savePlots.docx]

# AGG: Plot Functions

**Author:** *Amanda Gibson*

**Updated:** *April 14, 2022*

- [Save Plots Panel Interface](#save-plots-panel-interface)
  - [makeSavePanel](#makesavepanel)
- [Notes on exporting from Igor to Illustrator](#X8c3d454614a9cf599bda73bd7c4d3f59872a4aa)
  - [General Information](#general-information)
  - [Windows](#windows)
  - [Mac](#mac)
- [Save Plots](#save-plots)
  - [savePlot](#saveplot)
- [Plot Panel Procedures](#plot-panel-procedures)
  - [updateGraphsProc](#updategraphsproc)
  - [updateSaveGraphList](#updatesavegraphlist)
  - [dupGraphProc](#dupgraphproc)
  - [updateSavePanelSelsProc](#updatesavepanelselsproc)
  - [updateSavePanelSelections](#updatesavepanelselections)
  - [removeAxesProc](#removeaxesproc)
  - [openCalibratorProc](#opencalibratorproc)
  - [copyGraphButtonProc](#copygraphbuttonproc)
  - [saveGraphButtonProc](#savegraphbuttonproc)
  - [openSaveIntProc](#opensaveintproc)

# Save Plots Panel Interface

## makeSavePanel

### Hosting macro

- AGG analysis menu -> Save Panel

### Parameters

- hostPanel: [optional] string of the host panel that should be selected initially. If not provided, defaults to “none” for standalone graphs

### Returns

- panelName: name of the save interface panel

### Global Changes

- Create the saveInterface panel for copying or saving a graph for use outside of Igor

### Instructions

- View [Exporting Graphs From Igor To Illustrator](https://youtu.be/pfxpGJk5XwY) for a demonstration of using this panel to copy a raw data trace into Igor.
- Host Panel:
  - Select the host panel for the graph that you wish to export
  - If you want a standalone graph, select “none”
- Pick a graph:
  - Select the graph that you wish to export
    - The list will show only the graphs in the selected host panel or all standalone graphs if “none” is selected
- REMAKE GRAPH button:
  - This will create a new standalone duplicate of the selected graph
  - This is useful if you want to make modifications before export, or if you want to use the Igor Export Graphics window on a graph that is a subwindow
- remove axes button:
  - This will remove the axes from the selected graph
  - This is useful for exporting traces with a calibration scale instead
- open calibrator button:
  - This will open the Append Calibrator window that is built into Igor
  - This can be used to add a calibration scale to the graph
  - Another option to add calibration is to use the scalebar1 or scalecc macros which provide some defaults for common ephys graphs
- Units:
  - Select whether you want to provide the size of the graph in inches, centimeters, or pixels
- Width:
  - Enter the width for the exported graph
- Height:
  - Enter the height for the exported graph
- File type:
  - Select the file type for the exported graph
    - Use EMF on Windows and PDF on Mac for copying into Illustrator
- COPY button:
  - Copy graph image to clipboard
- File Name:
  - Enter the desired file name if saving the graph to a file
  - You’ll have a chance to edit this
  - If not entered, defaults to graph name
- SAVE button:
  - Opens file save interface to save image as selected file type

Note: if you have made additional panels since last creating the save plots interface, you should run the macro or call the makeSavePanel function again. It will save you current choices even if you recreate the panel

### File

[AGG_tabProcs_savePlots.ipf](../../AGG_procs/plots/savePlots/AGG_tabProcs_savePlots.ipf)

# Notes on exporting from Igor to Illustrator

## General Information

- As much as possible, edit the traces to as close to their final state in Igor, rather than trying to edit within Illustrator
- Adjust resolution if needed, though be aware that this will change file size

## Windows

- Simple copy (ctrl + c)
  - Standalone graph
    - Copies as an EMF (enhanced metadata file)
    - This can be pasted directly into illustrator
  - Graph within a panel
    - Copies as a PNG, with the entire panel image
- Copying to the clipboard using SavePICT function
  - Use the [Save Plots Panel Interface](#save-plots-panel-interface) OR edit the output from the Export Graphics window. Use as "clipboard" for the filename at the end of the command string and run in the command window
  - **EMF is the best file type** for copying and pasting into Illustrator
    - This is a vector-based format, meaning the contents of the plot can be edited in Illustrator (such as changing line width or color)
    - The size will be very close to what is specified by the width and height options, but will be slightly off due to white space in the plot
      - You could try using ModifyGraph to adjust the whitespace that is present, or resize the graph itself
  - EPS should be a save-only file type, and when using [savePlot](#saveplot), the copy output will switch to EMF
  - BMP (high resolution bitmap) does allow you to paste into Illustrator, but this is not vector-based and is just a static image of the traces
  - SVG, TIFF, PDF, JPEG, and PNG will not paste directly into Illustrator after being saved on the clipboard in Igor
- Saving the graph first and then embedding into Illustrator
  - SVG, PDF, EPS, and EMF are all vector-based
  - PDF and EPS can be directly linked/embedded into the file that you’re editing in Illustrator
  - SVG and EMF first open in their own file, and then you can copy and paste the vector traces into the file that you’re editing
  - White space
    - There are some slight differences in exactly how each file type seems to handle some of the white space and line information that lead to some small differences in the exact final size of the graph in Illustrator, but that are all the correct aspect ratio as specified by the SavePICT function
  - Font embedding
    - Despite embedding the fonts in the files, the PDF document had some trouble with the embedded fonts when the file was embedded into an Illustrator file.
      - Igor called the font “ArialRegular,” and had to be changed with a fonts interface to “Arial.”
    - Fonts were not a problem for SVG, EPS, or EMF

## Mac

- Quartz PDF option is the best for use in Illustrator

# Save Plots

## savePlot

### Parameters

- displayName: string for which graph should be saved
- fileName: [optional] string for name for the image - if provided, will prompt to save plot. If not, will save to clipboard
- units: [optional] string for the units for the size of the plot. Default is points. Options are
  - “in” - inches
  - “cm” - centimeters
  - “pts” - points
- width: [optional] variable for the width of the plot on output
- height: [optional] variable for the height of the plot on output
  - Both width and height must be provided if either is going to be used
  - If not provided, default is the size of the plot in Igor
- fileType: String for type of file. Defaults to PNG if not provided. Options are:
  - SVG
  - PDF
    - On Mac, uses Quartz PDF
    - On Windows, uses Igor PDF
  - TIFF
  - JPEG
  - PNG
  - EPS
    - If selected, but trying to copy the file, uses Quartz PDF on Mac and EMF on Windows
  - EMF (enhanced metafile)
    - If selected on a Mac, defaults to Quartz PDF instead

### Returns

- If displayName is a graph, saves to clipboard to opens file dialog based on provided parameters

### Global Changes

- If no fileName is provided, will overwrite clipboard with graph plot

### Purpose

To save graphs for use outside of Igor. Will work with graphs that are in subpanels.

Note that the function does not currently allow for changing the resolution of the image. See SavePICT Igor function for information about how to edit the command to change the resolution

### File

[AGG_savePlots.ipf](../../AGG_procs/plots/savePlots/AGG_savePlots.ipf)

# Plot Panel Procedures

## updateGraphsProc

### Procedure type

- Called by changing the popup menu

### Associated controls

- hostPanelToPick: the popup menu for host panel

### Global Changes

- Changes the graph options under the popup menu graphToPick

### Purpose

- Calls [updateSaveGraphList](#updatesavegraphlist)

### File

[AGG_tabProcs_savePlots](../../AGG_procs/plots/savePlots/AGG_tabProcs_savePlots.ipf)

## updateSaveGraphList

### Global Changes

- Changes the graphsToPick popup menu to return all graphs in the selected host panel, or standalone graphs

### Purpose

- Gets the list of graphs with the returnGraphsInPanel function
- Updates the “pick a graph” popup menu

### File

[AGG_tabProcs_savePlots](../../AGG_procs/plots/savePlots/AGG_tabProcs_savePlots.ipf)

## dupGraphProc

### Procedure Type

- Button Control

### Associated Controls

- dupGraphButton: REMAKE GRAPH

### Global Changes

- Duplicates the selected graph into a standalone graph
- Selects the newly created graph wihtin the save interface

### Purpose

- Gets the host panel (if applicable) and the graph name from updateSavePanelSelections
- Uses the duplGraphInPanelSubwndw function to duplicate the graph
- Resets the host panel to “none” and then updates the graph list popup with updateSaveGraphList

### File

[AGG_tabProcs_savePlots](../../AGG_procs/plots/savePlots/AGG_tabProcs_savePlots.ipf)

## updateSavePanelSelsProc

### Procedure Type

- Popup menu control

### Associated Controls

- unitsToPick: units popup menu
- fileTypeToPick: file type popup menu

### Global Changes

- Runs updateSavePanelSelections to set global variables when these popups change

### File

[AGG_tabProcs_savePlots](../../AGG_procs/plots/savePlots/AGG_tabProcs_savePlots.ipf)

## updateSavePanelSelections

### Global Changes

- Updates the hostPanel, graphName, units, and fileType global strings based on current save interface panel selections
- If “none” is selected for the host panel, the string is set to "" (empty string)

### Purpose

Updates the global variables so that these values can be used in other functions when copying or saving the data

### File

[AGG_tabProcs_savePlots](../../AGG_procs/plots/savePlots/AGG_tabProcs_savePlots.ipf)

## removeAxesProc

### Procedure Type

- Button control

### Associated Controls

- removeAxesButton: “remove axes”

### Global Changes

- Runs the removeAxes function on the selected graph and elements the axes and labels

### Purpose

- Remove the axes from traces - generally useful for exporting example traces for creation of figures. Typically will be used in conjunction with the addition of a calibrator scale

### File

[AGG_tabProcs_savePlots](../../AGG_procs/plots/savePlots/AGG_tabProcs_savePlots.ipf)

## openCalibratorProc

### Procedure Type

- Button control

### Associated Controls

- openCalibratorButton: “open calibrator”

### Global Changes

- Brings the selected host/graph to the front
- Sets the graph as the active subwindow
- Runs the calibrator function for the built in Append Calibrator package in Igor
- Rescales the calibrator panel to ensure it fits on the device screen

### Purpose

- Open panel to allow user to customize and set the calibrator scale
- This is typically going to be used for example electrophysiology traces where the axis is removed. See removeAxesProc

### File

[AGG_tabProcs_savePlots](../../AGG_procs/plots/savePlots/AGG_tabProcs_savePlots.ipf)

## copyGraphButtonProc

### Procedure Type

- Button control

### Associated Controls

- copyButton: “COPY”

### Global Changes

- Updates the global string variables using updateSavePanelSelections
- Builds the display name using the host panel (if applicable) and graph name
- Copies the plot with the selected settings using the savePlot function
  - Because no file name is provided, savePlot defaults to copy the graph instead of saving it

### Purpose

- Copy the selected graph to clipboard

### File

[AGG_tabProcs_savePlots](../../AGG_procs/plots/savePlots/AGG_tabProcs_savePlots.ipf)

## saveGraphButtonProc

### Procedure Type

- Button control

### Associated Controls

- saveButton: “SAVE”

### Global Changes

- Updates the global string variables using updateSavePanelSelections
- Builds the display name using the host panel (if applicable) and graph name
- Saves the plot with the selected settings using the savePlot function
- If no file name is provided, defaults to the name of the graph
- Opens a save dialog for user to select path, and edit name if necessary

### Purpose

- Save the selected graph to device

### File

- Updates the global string variables using updateSavePanelSelections
- Builds the display name using the host panel (if applicable) and graph name
- Copies the plot with the selected settings using the savePlot function
  - Because no file name is provided, savePlot defaults to copy the graph instead of saving it

## openSaveIntProc

### Procedure Type

- Button control

### Associated Controls

- savePassGraph
- saveSeriesSum
- saveSeriesGroups
- saveRawGraph
- saveCellSum
- saveCellsGroups
- saveGroupEvents

### Global Changes

- (Re)opens the save interface panel using with defaults set based on the properties of the calling button
- Uses the window in which the calling button is located for the selection of the host panel
  - This is provided to makeSavePanel when creating the panel
- The button with this procedure attached should have the name of the graph in the userdata field that should be selected when the panel is opened
  - The graphsToPick PopupMenu is updated using popmatch

### Purpose

- Open the save interface to copy or save a graph - set host panel and graph to those associated with the calling button

### File

[AGG_tabProcs_savePlots](../../AGG_procs/plots/savePlots/AGG_tabProcs_savePlots.ipf)
