# Supplementary material for: Early-Life Resource Scarcity in Mice Does Not Alter Adult Corticosterone or Preovulatory Luteinizing Hormone Surge Responses to Acute Psychosocial Stress
Source: eNeuro. 2024 Jul 26;11(7):ENEURO.0125-24.2024. doi: 10.1523/ENEURO.0125-24.2024 (PMC11287788; doi:10.1523/ENEURO.0125-24.2024)
Supplement: Extended Data — Zip file of custom code for PSC detection and analysis, ffmpeg recording of dam behavior, and R analysis. Download Extended Data, ZIP file. [file eneuro-11-ENEURO.0125-24.2024-s002.zip › PSC-analysis/documentation/AGG_procs/saveTables.docx]

# AGG: Save Tables

**Author:** *Amanda Gibson*

**Updated:** *April 13, 2022*

- [Save tables](#save-tables)
  - [saveTable](#savetable)
  - [saveTableProc](#savetableproc)

# Save tables

## saveTable

### Parameters

- tableName: string of table name, including host
- [optional] fileName: string of file name
  - user will also get a chance to change this when saving

### Purpose

Save an Igor table as a .csv file. Works with standalone tables or tables within a panel. Default file name if not provided is the name of the table

### File

[AGG_saveTables.ipf](../../AGG_procs/tables/AGG_saveTables.ipf)

## saveTableProc

### Parameters

- Button structure - passed by Button
  - Should have userdata with a "tableName" field containing the name of the table
  - Optional "tableWindow" field of user data. If not provided, it will default to looking within the same window as the button
  - Optional "fileName" field of user data. Default is table name if not provided

### Purpose

Save a table as a .csv file by pressing a button within a panel

### File

[AGG_saveTables.ipf](../../AGG_procs/tables/AGG_saveTables.ipf)
