# Supplementary material for: Early-Life Resource Scarcity in Mice Does Not Alter Adult Corticosterone or Preovulatory Luteinizing Hormone Surge Responses to Acute Psychosocial Stress
Source: eNeuro. 2024 Jul 26;11(7):ENEURO.0125-24.2024. doi: 10.1523/ENEURO.0125-24.2024 (PMC11287788; doi:10.1523/ENEURO.0125-24.2024)
Supplement: Extended Data — Zip file of custom code for PSC detection and analysis, ffmpeg recording of dam behavior, and R analysis. Download Extended Data, ZIP file. [file eneuro-11-ENEURO.0125-24.2024-s002.zip › PSC-analysis/documentation/old/NEW INSTALL INSTRUCTIONS 20191213.docx]

1 Installation

Unzip the td analysis file. Make an alias to the folder. Move the alias to the Igor Procedures folder in your Documents/Wavemetrics folder. Use the folder in your Documents folder, not the Wavemetrics folder in the applications folder.
