# Supplementary material for: Early-Life Resource Scarcity in Mice Does Not Alter Adult Corticosterone or Preovulatory Luteinizing Hormone Surge Responses to Acute Psychosocial Stress
Source: eNeuro. 2024 Jul 26;11(7):ENEURO.0125-24.2024. doi: 10.1523/ENEURO.0125-24.2024 (PMC11287788; doi:10.1523/ENEURO.0125-24.2024)
Supplement: Extended Data — Zip file of custom code for PSC detection and analysis, ffmpeg recording of dam behavior, and R analysis. Download Extended Data, ZIP file. [file eneuro-11-ENEURO.0125-24.2024-s002.zip › PSC-analysis/documentation/old/td analysis install 2018.docx]

1 Installation

Note: file and folder names are in quotes to help minimize confusion with long names.

First, unzip the “td analysis.zip” file provided and make either aliases (macOS) or shortcuts (Windows) of both the “td analysis” folder and the “link to igor procs” folder contained within.

Igor makes a folder called “Wavemetrics” that can be found in either your “Documents” (macOS) or “My Documents” (Windows) folder. Inside this folder there are two additional folders named “Igor Procedures” and “User Procedures.” Move the “link to igor procs” alias you created previously into the “Igor Procedures” folder. Similarly, move the “td analysis” alias you created into the “User Procedures” folder.

Next, go back to the “td analysis” folder and open the “link to igor procs” folder. Copy the file “IVtiming.txt.” Move the copy to the “Igor Procedures” folder inside the “Wavemetrics” folder (inside your “Documents” folder). Rename the copied file to remove the ‘copy’ and the space before ‘copy’ and ensure the name is exactly “IVtiming.txt”—capitalization is important.

Note: the “Igor Pro” folder in the Applications folder contains folders with the same names as above but these should NOT be modified. Instead, make sure all modifications are done in the “Wavemetrics” folder in either the “Documents” or “My Documents” folder.
