# Supplementary material for: Early-Life Resource Scarcity in Mice Does Not Alter Adult Corticosterone or Preovulatory Luteinizing Hormone Surge Responses to Acute Psychosocial Stress
Source: eNeuro. 2024 Jul 26;11(7):ENEURO.0125-24.2024. doi: 10.1523/ENEURO.0125-24.2024 (PMC11287788; doi:10.1523/ENEURO.0125-24.2024)
Supplement: Extended Data — Zip file of custom code for PSC detection and analysis, ffmpeg recording of dam behavior, and R analysis. Download Extended Data, ZIP file. [file eneuro-11-ENEURO.0125-24.2024-s002.zip › PSC-analysis/documentation/Smart Concatenate/JPsmartConcPanel info.docx]

JPsmartConcPanel info

Please report any trouble you have. Be very careful to save copies of your analyzed data before you play with the new routines! They are destructive.

The steps to get the new event analysis software to work:

0. Back up your igor files that you plan to examine.

1. Open a pre-existing igor project containing detected events you have already confirmed and finalized.

2. Close all windows, panels, graphs, tables, etc.

3. Run the macro "destroyallmonsters". This kills all waves except those needed by the new "smart concatenate" routine (_ptb and associated raw data waves). you must enter 666 to confirm destruction of so many waves. Please be sure that you have backed up your igor file before running this macro!

4. Run the macro JPsmartConcPanel.

5. Click the update button, this populates the listbox with all the _ptb

6. Click select all ( or select the ones you want to analyze ). the selected waves will be concatenated

7. If you want a histogram (necessary for cluster), set the binsize and check the box. Note that some bins may be too thin to display!

8. Click the "smart conc" button. graphs should appear of the raw data and the histogram. blue vertical lines indicate gaps between the _ptb series. Red histogram bins are bins that overlap with these gaps and are therefore compromised.

9. Click the vary burst window tab. select vbw settings (start, max, increment). click the "make vbw graph". the city plot should appear. VBW runs on the the concatenated _ptb (_sct), no histogram required. The VBW city plot is not a substitute for the histogram (i.e. cluster needs a histogram).

10. If you have experiments which would benefit from analysis regions, click the regions button. this generates an error sometimes (just click the green arrow if you have the debugger on, click ok if you don't). A table should appear with the columns: names, starts, ends. Enter the names of the regions, then enter the start times (in seconds) and end times (in seconds). You can make extra rows by entering info in the gray boxes or use insert points. When you have finished entering the information and there are no blank cells, click the "regions vbw" button again. Now the city plot should appear with the regions colored rainbow-style (black to red). Note that some bins may be too thin to display! Also, note that a number of summary tables are created. These reflect the summary data (average) for each analysis parameter, for each burst duration by region.

11. To run cluster-like analysis, click the cluster tab, adjust the settings to taste, click calculate. Yellow vertical bars indicate peaks detected by cluster. Note that some bins may be too thin to display! also note that cluster runs on data in the histogram: no histogram, no cluster. The histogram and the peaks of the histogram may not line up exactly with the _ptb/_sct data because of the size of the histogram bins. However, histograms and cluster-like analysis should line up "perfectly".

Right now there is no export utility. You can do screen grabs until we have a work-around.
