# Supplementary material for: Early-Life Resource Scarcity in Mice Does Not Alter Adult Corticosterone or Preovulatory Luteinizing Hormone Surge Responses to Acute Psychosocial Stress
Source: eNeuro. 2024 Jul 26;11(7):ENEURO.0125-24.2024. doi: 10.1523/ENEURO.0125-24.2024 (PMC11287788; doi:10.1523/ENEURO.0125-24.2024)
Supplement: Extended Data — Zip file of custom code for PSC detection and analysis, ffmpeg recording of dam behavior, and R analysis. Download Extended Data, ZIP file. [file eneuro-11-ENEURO.0125-24.2024-s002.zip › PSC-analysis/documentation/td analysis/ap analysis/ap analysis slides 20131002 v0.pptx]

## Slide 1
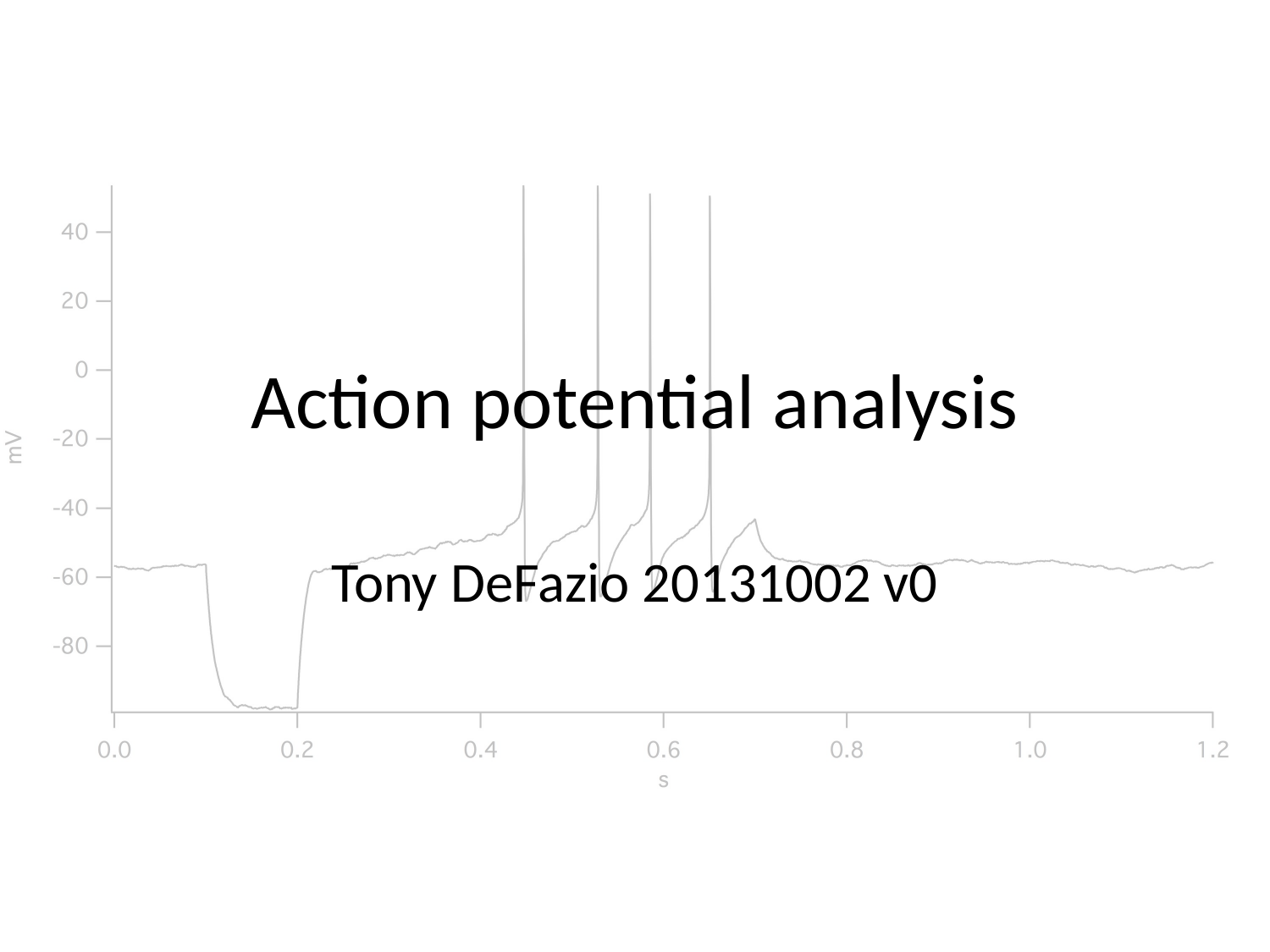

# Action potential analysis
Tony DeFazio 20131002 v0

## Slide 2
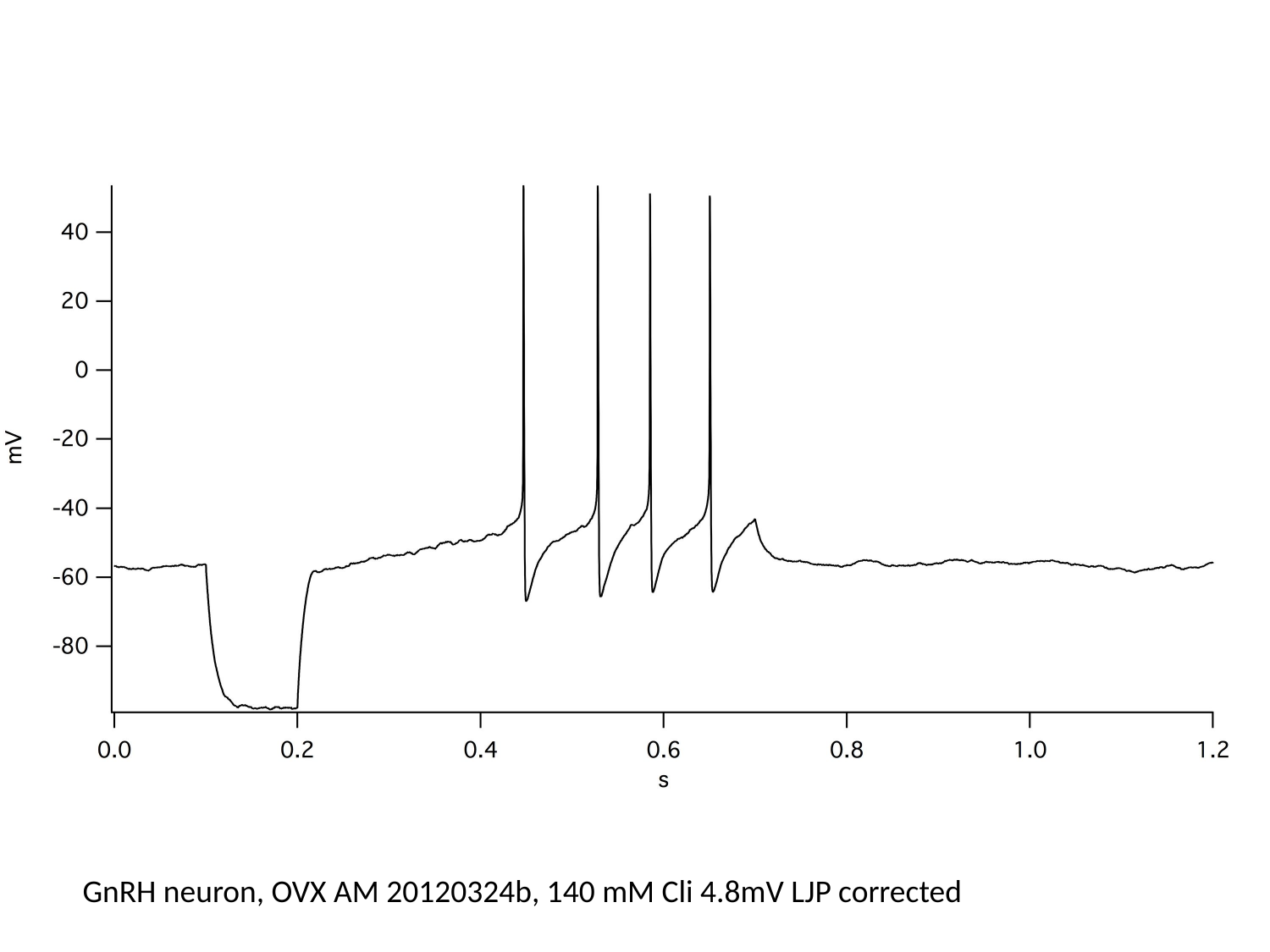

GnRH neuron, OVX AM 20120324b, 140 mM Cli 4.8mV LJP corrected

## Slide 3
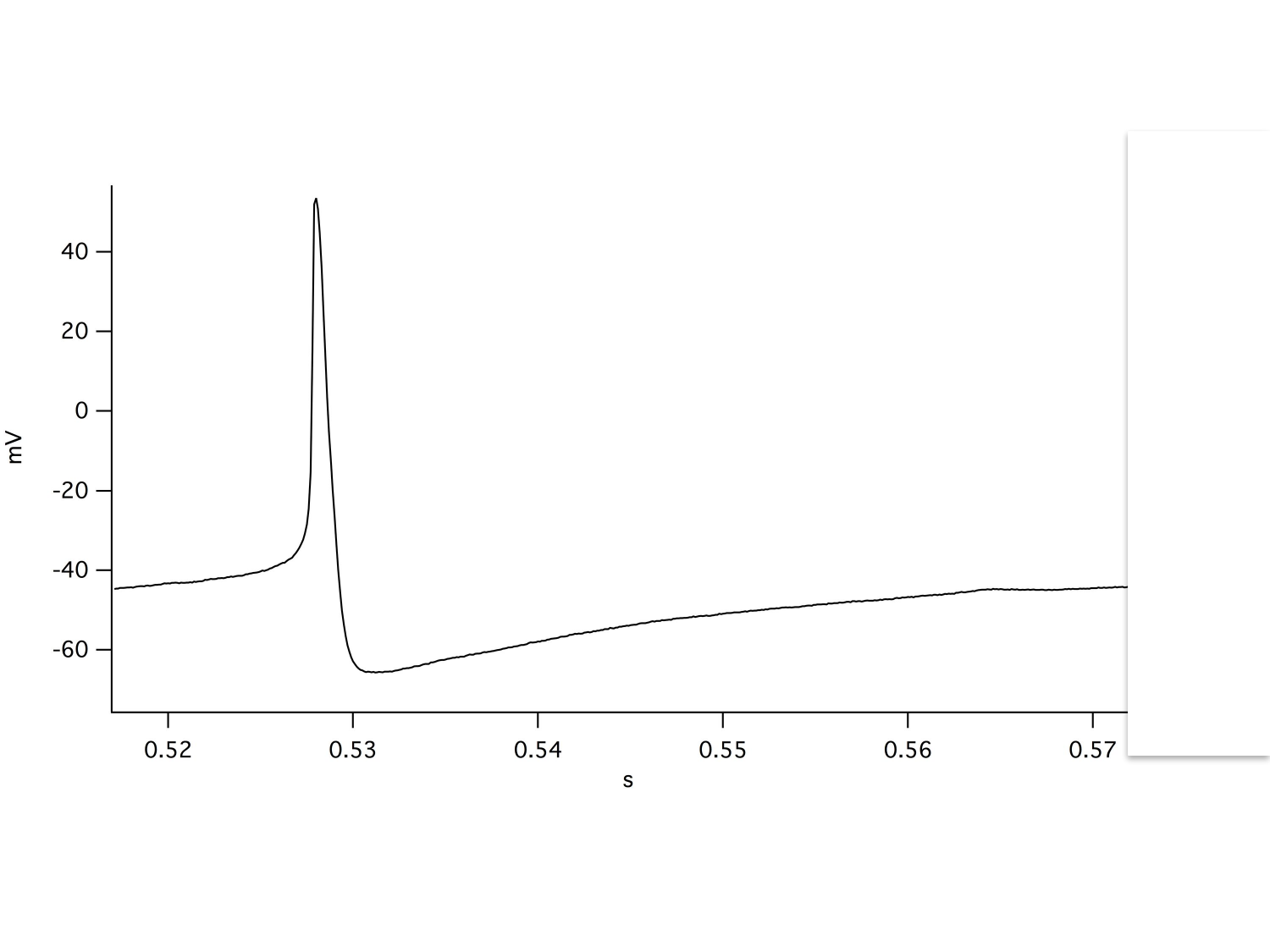

## Slide 4
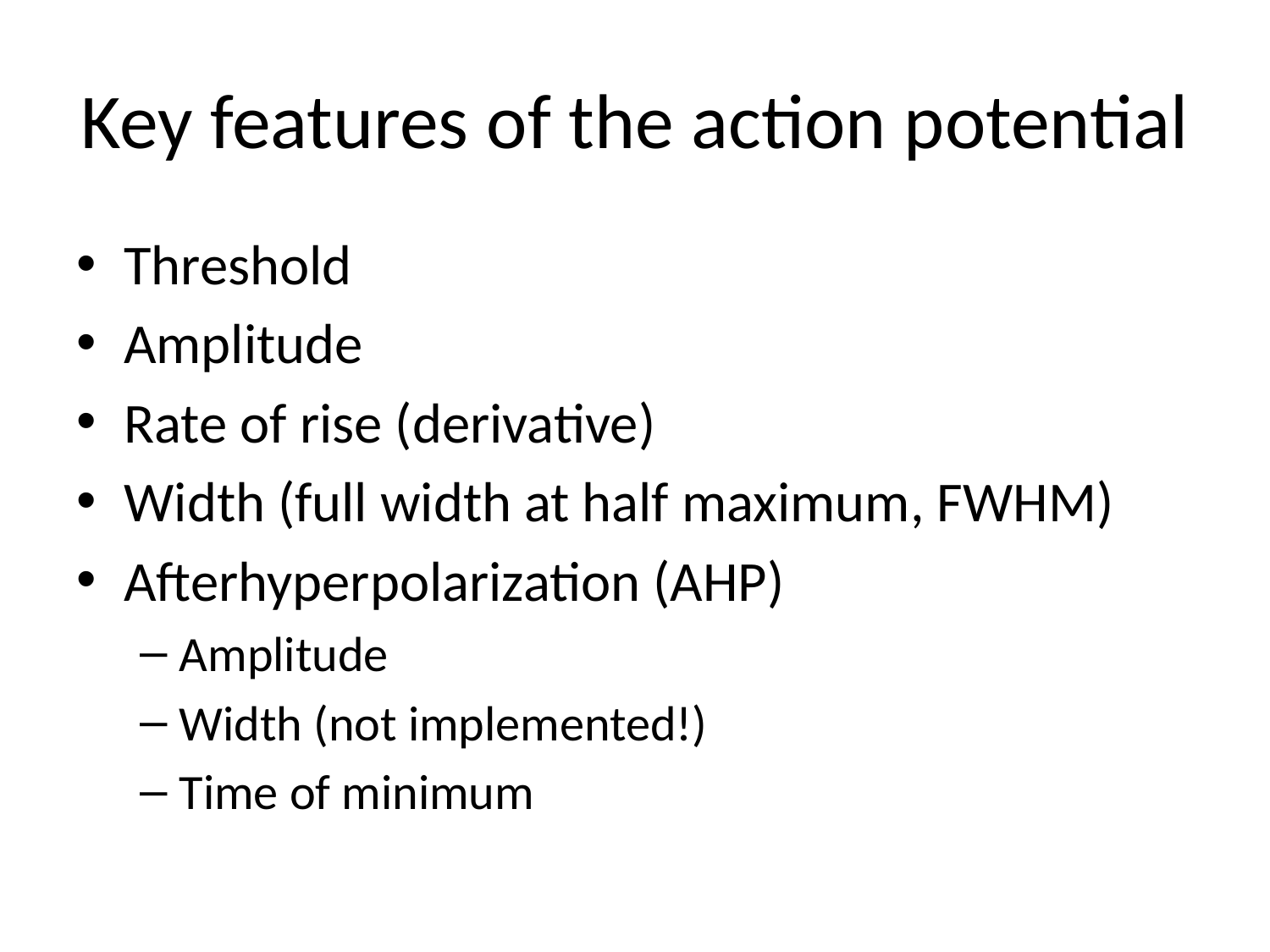

# Key features of the action potential
Threshold
Amplitude
Rate of rise (derivative)
Width (full width at half maximum, FWHM)
Afterhyperpolarization (AHP)
Amplitude
Width (not implemented!)
Time of minimum

## Slide 5
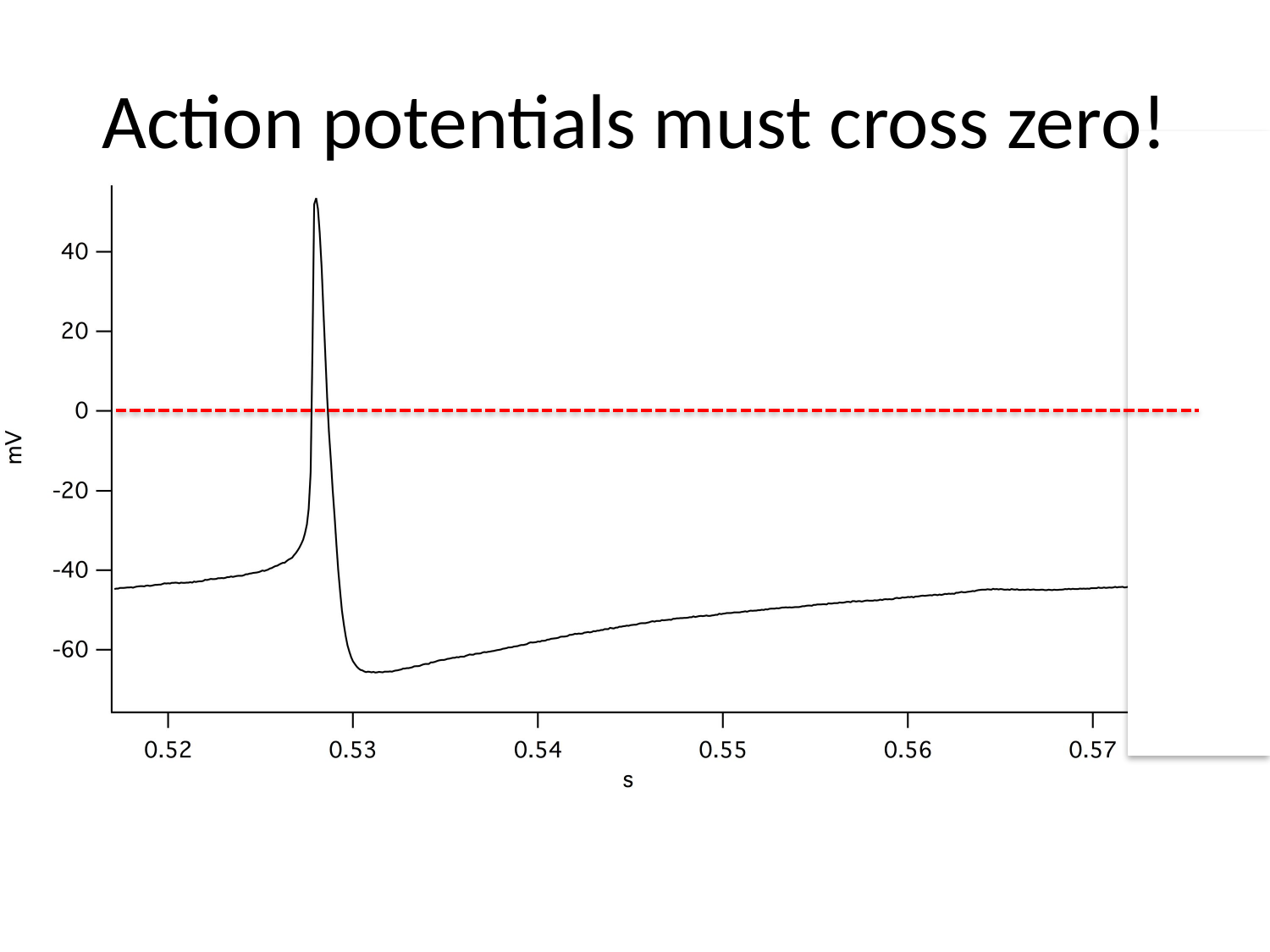

# Action potentials must cross zero!

## Slide 6
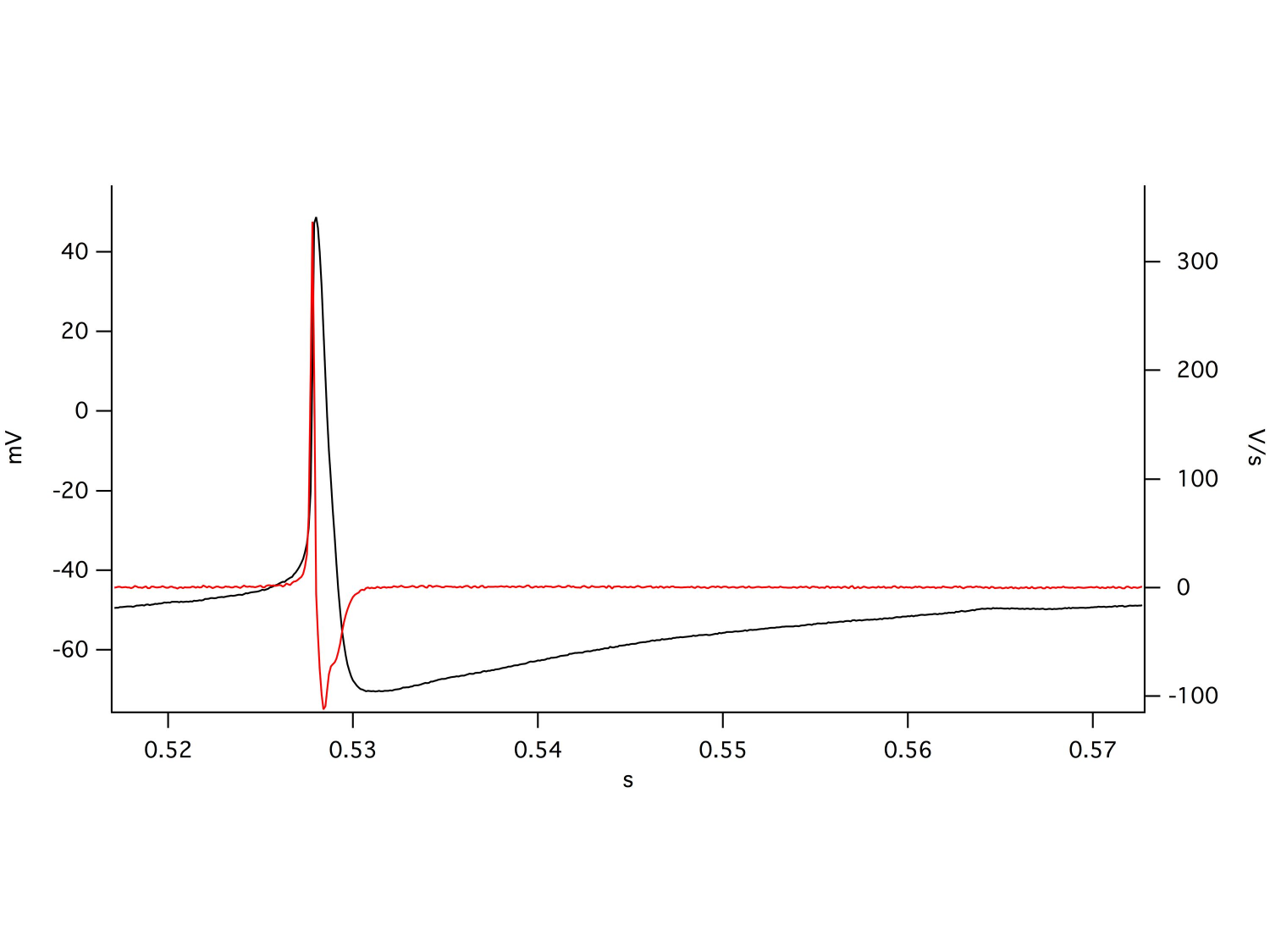

## Slide 7
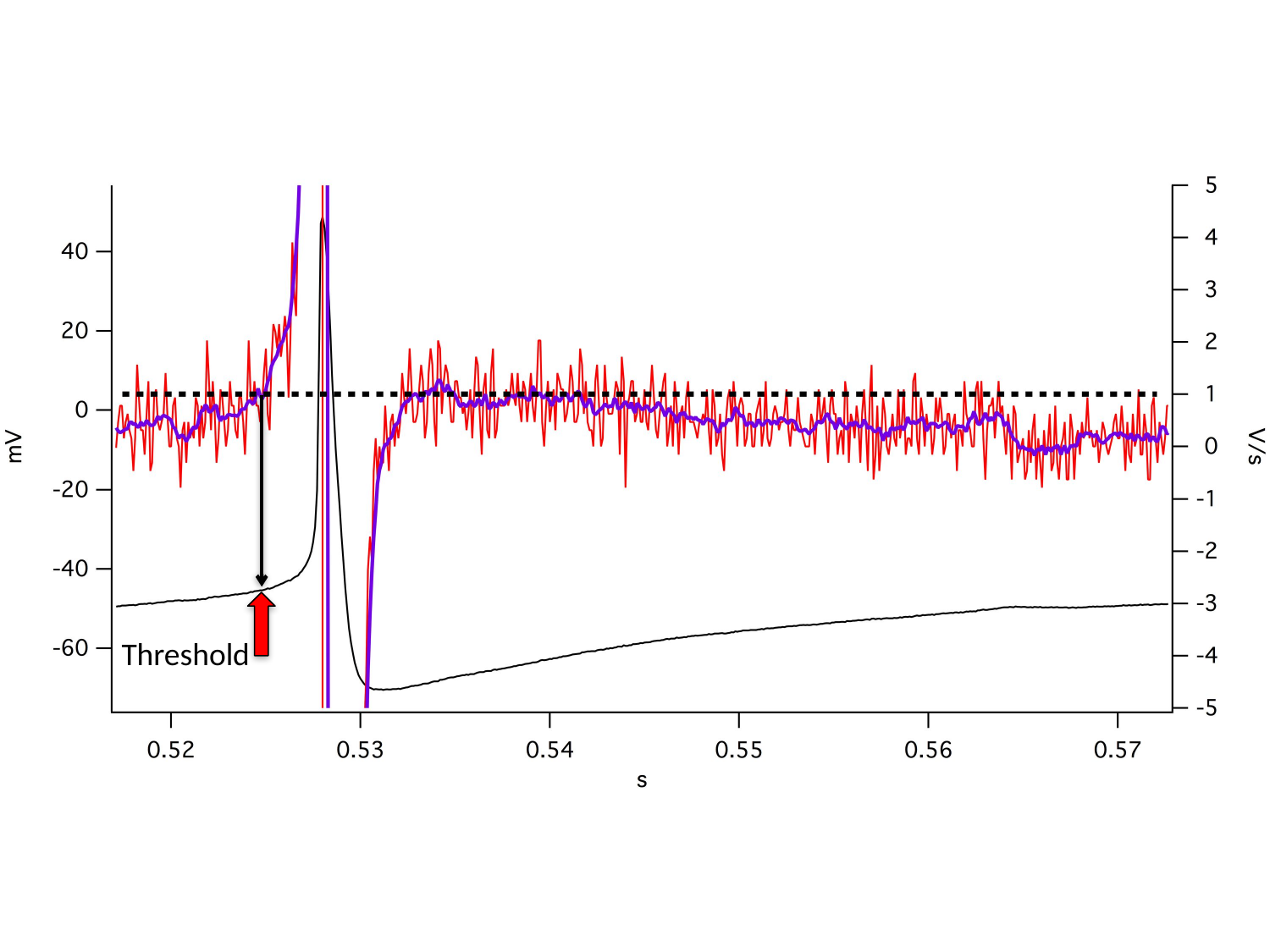

Threshold

## Slide 8
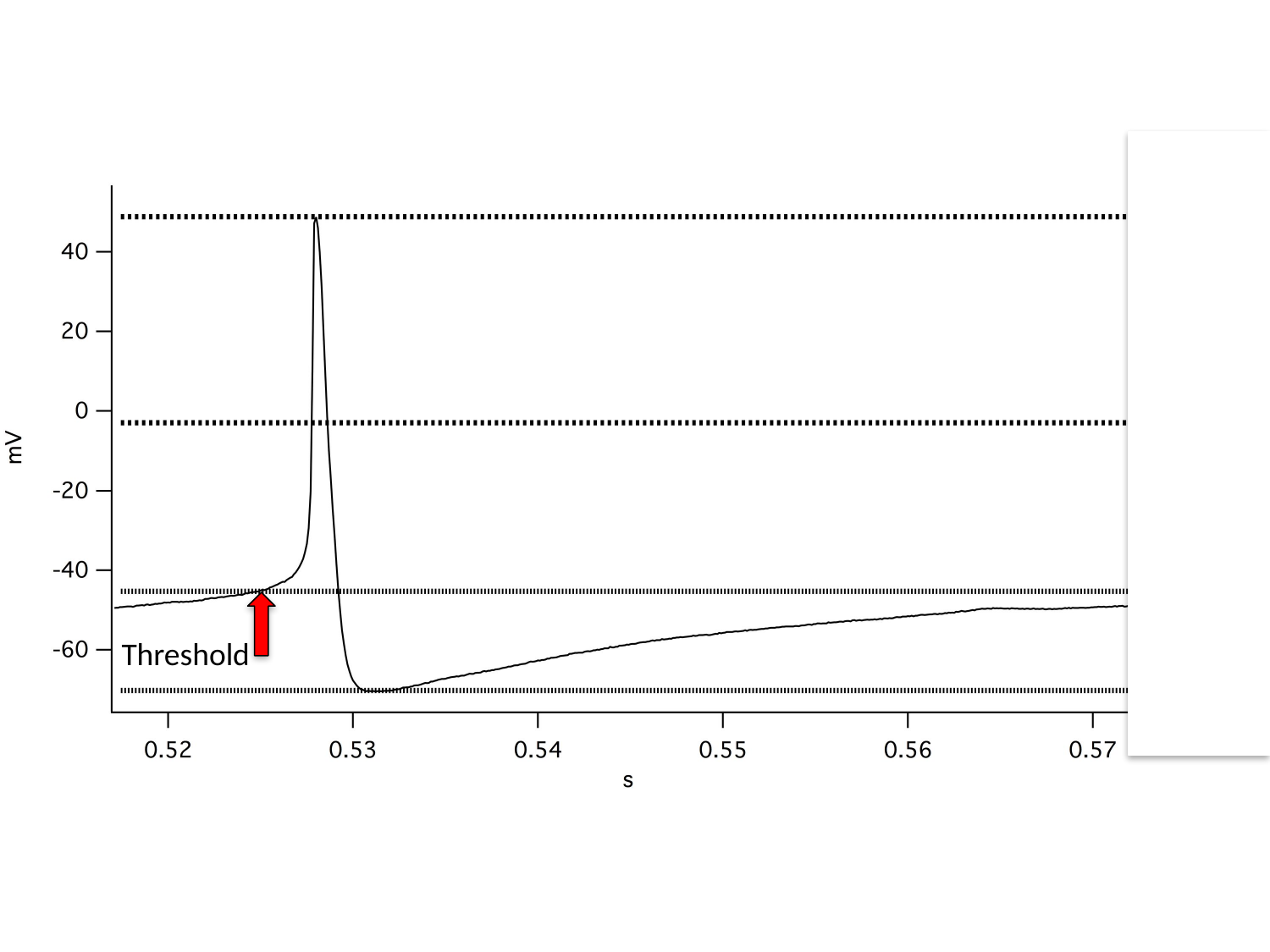

Threshold

## Slide 9
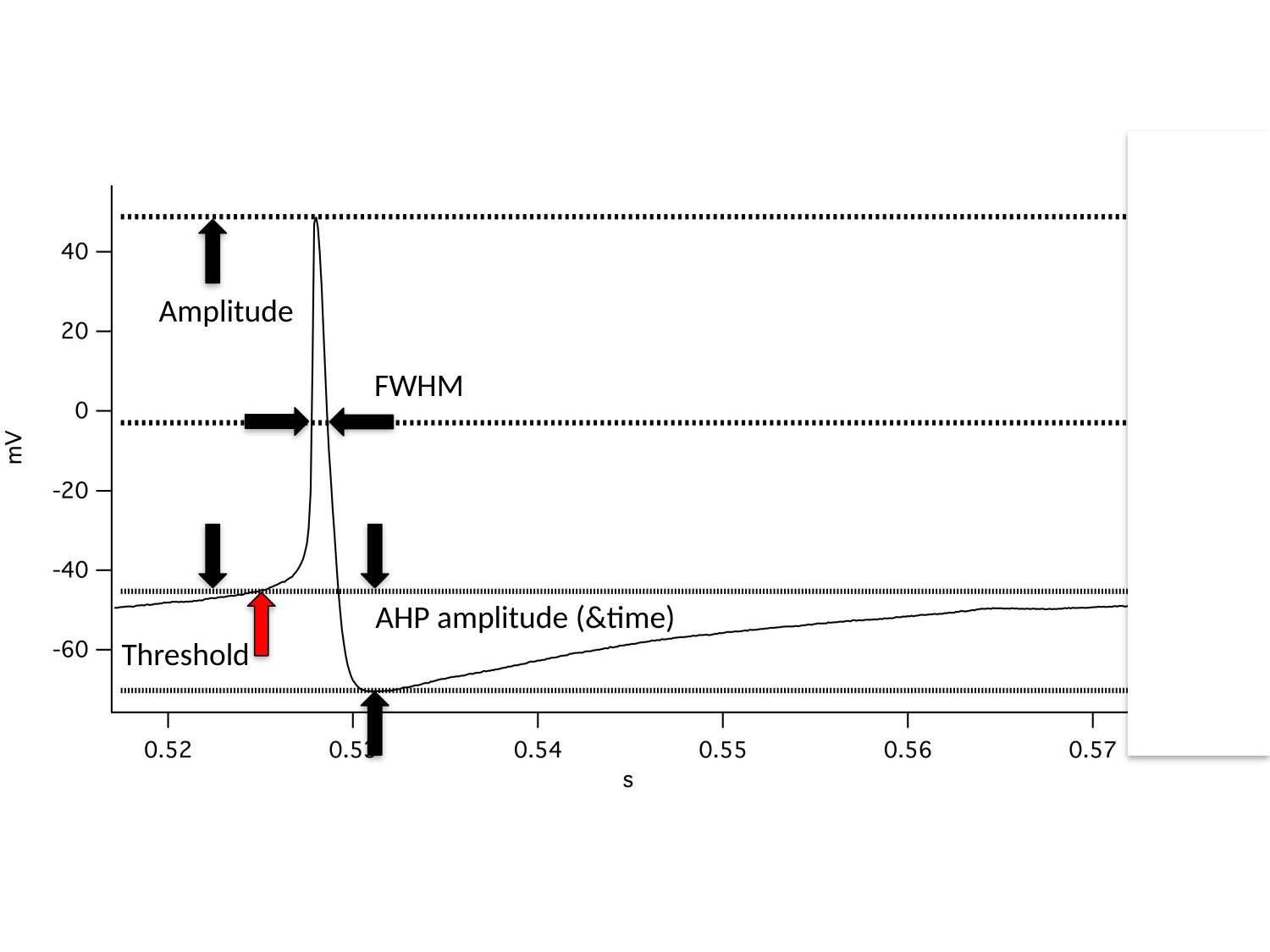

Amplitude
FWHM
AHP amplitude (&time)
Threshold

## Slide 10
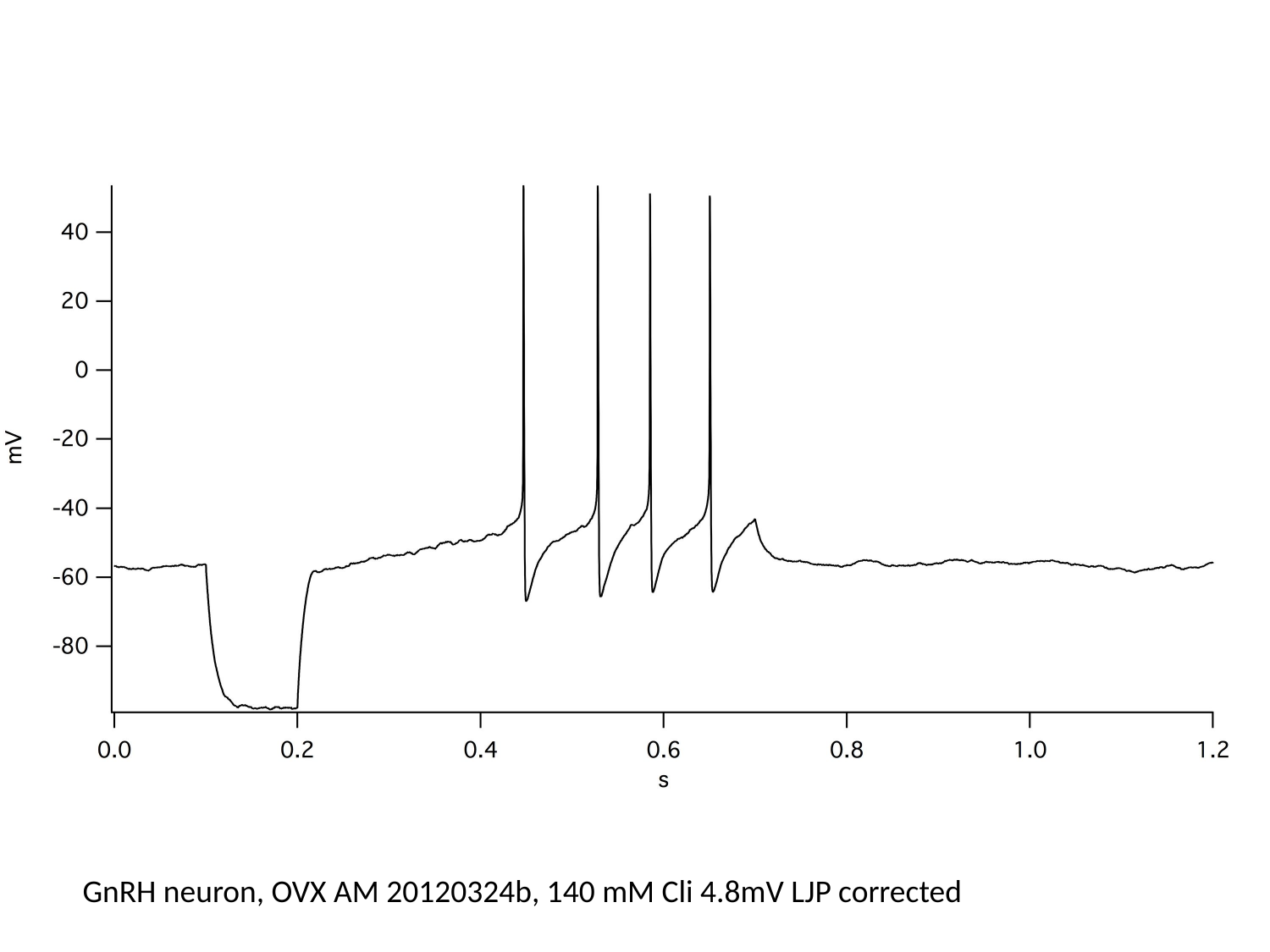

GnRH neuron, OVX AM 20120324b, 140 mM Cli 4.8mV LJP corrected

## Slide 11
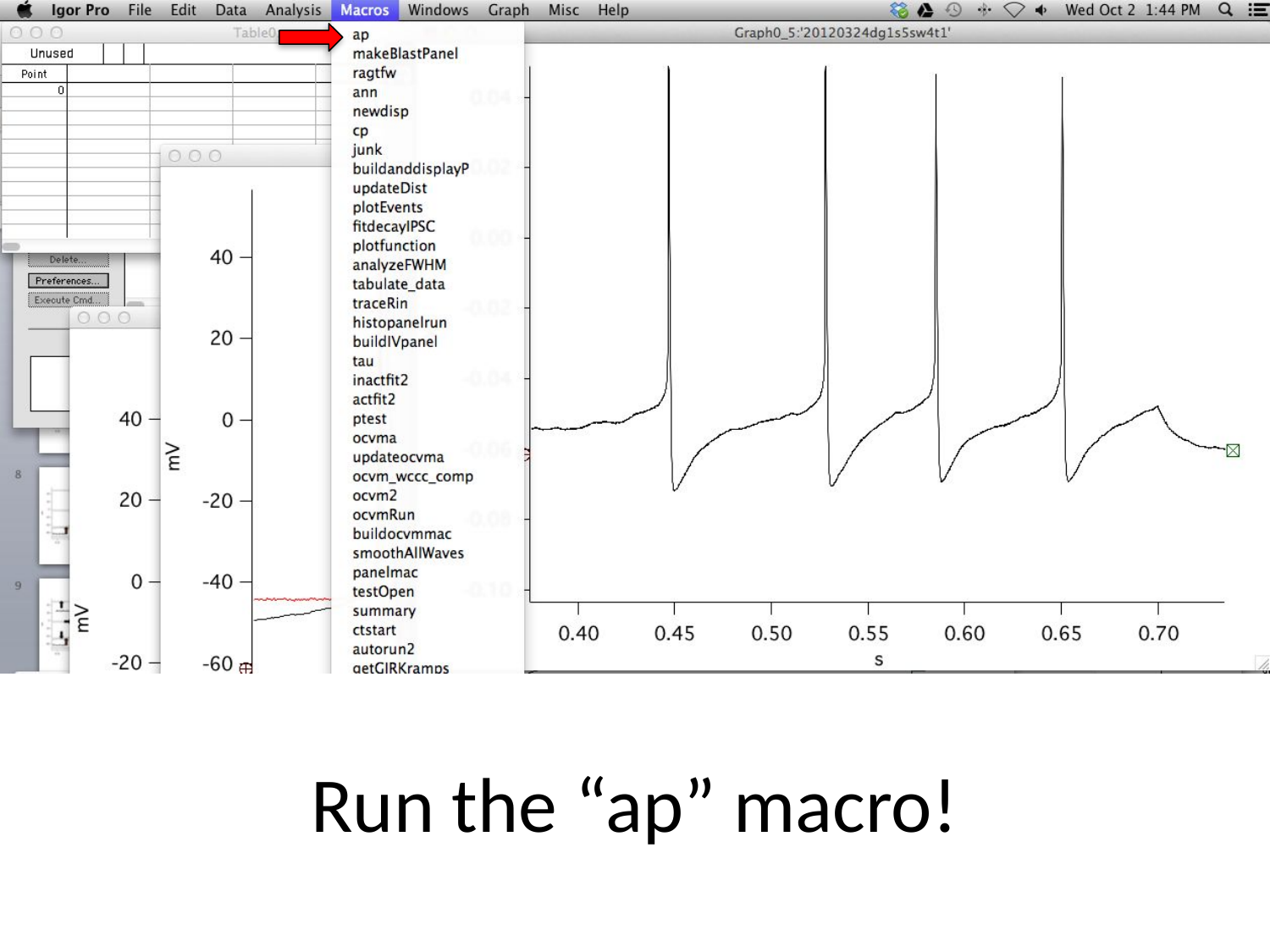

# Run the “ap” macro!

## Slide 12
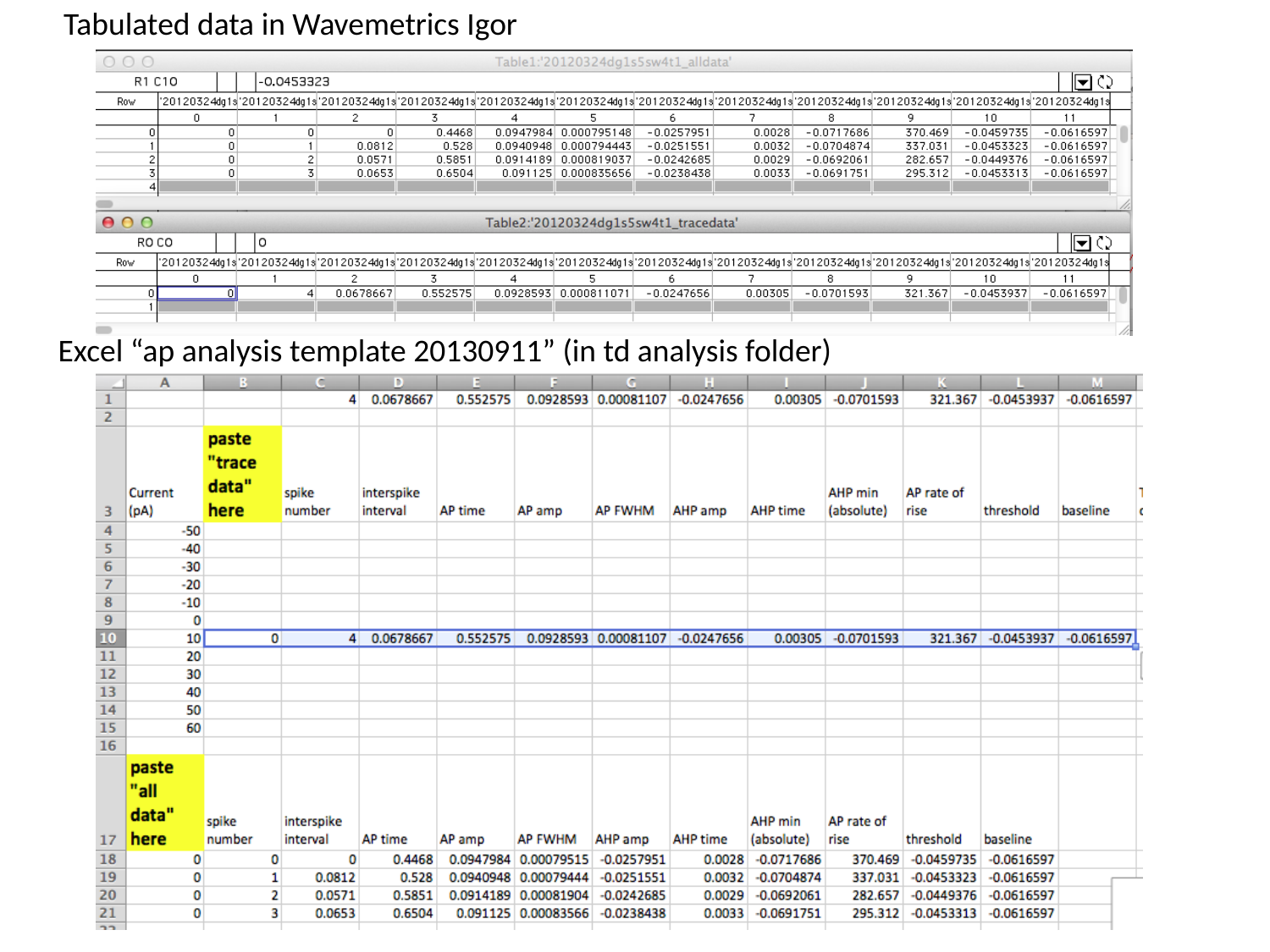

Tabulated data in Wavemetrics Igor
Excel “ap analysis template 20130911” (in td analysis folder)
