# Supplementary material for: Early-Life Resource Scarcity in Mice Does Not Alter Adult Corticosterone or Preovulatory Luteinizing Hormone Surge Responses to Acute Psychosocial Stress
Source: eNeuro. 2024 Jul 26;11(7):ENEURO.0125-24.2024. doi: 10.1523/ENEURO.0125-24.2024 (PMC11287788; doi:10.1523/ENEURO.0125-24.2024)

Chapter X: On-cell subtraction for passive analysis 2013-09-30

Often it is not possible to completely compensate electrode capacitance when making voltage clamp recordings in brain slices. Generally, this is not a major inconvenience as long as raw and leak subtraction traces are acquired at sufficiently low gain to fully capture the large capacitive transients without clipping. However, a major concern is an accurate measure of series resistance. We measure this property from the peak of the capacitive transient during a 5 mV hyperpolarizing step. Since this transient includes both the electrode resistance and capacitance and the charging of the series resistance, input resistance, and the whole-cell capacitance, it is important to minimize the contribution of the pipette. In cases when the electrode capacitance cannot be fully compensated electronically, *post hoc* digital subtraction is necessary.

In this case, acquisition of the passive properties (response to a 5 mV step, use a step PGF [assuming HEKA PatchMaster] identical to the one you use in whole-cell) during the on-cell phase of whole-cell recording is extremely valuable. After baseline correction of the on-cell passive trace, subtraction from the whole-cell passive trace yields our best approximation of the series resistance and capacitance of recording configuration (pipette, seal, and cell). Since the properties of on-cell can be dynamic, it is important to capture the on-cell passive trace immediately prior to any degree of whole-cell access. Generally, this is evidenced by extremely fast transients (figure X.1c). I tend to acquire 2-5 on-cell passive series (16 sweeps each) as the seal stabilizes over the course of 1-2 minutes prior to going whole-cell.

In BlastPanel, load your .dat file using the “Open PM Bundled File” button. To select the on-cell passive series for subtraction during whole-cell passive properties analysis, click on the popup item titled “On-cell ave series” (Figure X.1). It is labeled “NONE” at the start of the analysis. Click it once to detect all passive series. Now when you click it, it shows the series number of each passive series. When you select a series, it is displayed in Analysis Window 1 (the blue one) as a red trace to distinguish it from other types of display. Now that you’ve selected the on-cell trace for subtraction, click on the Passive analysis check box. The selected trace will be subtracted from each passive series for capacitive and series resistance measurement. For reference, the average series are saved as filename_avl. The subtracted average series are saved as filename_avl_sub.

Figure X.1


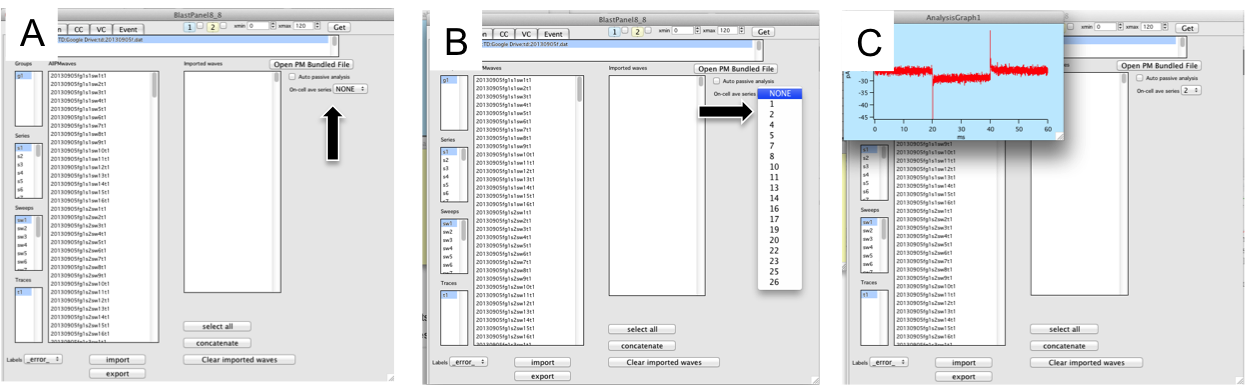

Supplement: Extended Data — Zip file of custom code for PSC detection and analysis, ffmpeg recording of dam behavior, and R analysis. Download Extended Data, ZIP file. [file eneuro-11-ENEURO.0125-24.2024-s002.zip › PSC-analysis/documentation/td analysis/Chapter on-cell subtraction v0-0.docx]
