# Supplementary material for: Early-Life Resource Scarcity in Mice Does Not Alter Adult Corticosterone or Preovulatory Luteinizing Hormone Surge Responses to Acute Psychosocial Stress
Source: eNeuro. 2024 Jul 26;11(7):ENEURO.0125-24.2024. doi: 10.1523/ENEURO.0125-24.2024 (PMC11287788; doi:10.1523/ENEURO.0125-24.2024)
Supplement: Extended Data — Zip file of custom code for PSC detection and analysis, ffmpeg recording of dam behavior, and R analysis. Download Extended Data, ZIP file. [file eneuro-11-ENEURO.0125-24.2024-s002.zip › PSC-analysis/documentation/td analysis/labels.docx]

2015-10-27

PGF names in PatchMaster and Labels in Igor

Some issues have arisen regarding how the "td_analysis" routines handle PGF names in Igor. This document is intended as guidance to getting the most out of your PGF name and facilitating future analysis sessions. The most recent versions of the "td_analysis" software take store the PGF name as a label for every trace during the import into Igor. These are accessible in the wave note (use "browse waves" to see the wave note in Igor). The analysis software can use these labels to sort waves for analysis. A prime example of this is the passive analysis: when you click this button, all waves with "passive" in the PGF name are analyzed. This allows you to use a PGF to monitor on-cell stability prior to whole-cell ("passive-fast") and another PGF to collect and average 16 sweeps during a recording (call this "passive"). Both sets of PGFs will be analyzed by the passive analysis routines, allowing you to visualize the progression of the passive properties of the entire experiment.

Therefore, I encourage you to use PGF naming conventions to distinguish between data series in PatchMaster that will be analyzed in different ways. Another example is OCVM. In this case there are several types of OCVM experiments: single ramps to monitor Vm over a long period of time (e.g. "OCVM-long"), double ramps to assess the effect of a neurotransmitter (e.g. "OCVM-double"). However, mild chaos will ensue if you use a passive protocol during an OCVM routine and call that PGF "OCVM-passive", since these traces won't have ramps for the OCVM analysis software.

The ability of the software to read the PGF name is new and can greatly facilitate analysis. If you want advice on naming conventions to optimize analysis, please feel free to talk to me. Furthermore, if you're having trouble with labels from old experiments, I can help you figure out how to make the best of it.
