# Supplementary material for: Early-Life Resource Scarcity in Mice Does Not Alter Adult Corticosterone or Preovulatory Luteinizing Hormone Surge Responses to Acute Psychosocial Stress
Source: eNeuro. 2024 Jul 26;11(7):ENEURO.0125-24.2024. doi: 10.1523/ENEURO.0125-24.2024 (PMC11287788; doi:10.1523/ENEURO.0125-24.2024)
Supplement: Extended Data — Zip file of custom code for PSC detection and analysis, ffmpeg recording of dam behavior, and R analysis. Download Extended Data, ZIP file. [file eneuro-11-ENEURO.0125-24.2024-s002.zip › PSC-analysis/documentation/td analysis/offline leak.docx]

2019 September 30

Offline leak math

Equation 1: I_leak_ = g_leak_ ( V – V_leak_ ) ; V_leak_ is the equilibrium potential for the leak current. This equation is generally true, i.e. this is the goal.

I_leak_ave_ is the trace obtained from a step from V_leak_pre_ to V_leak_post_. V_leak_pre_ is usually V_leak_

Equation 2: I_leak_sub_ = I_leak_ave_ * scale + I_offset_ ; this is the equation we used for offline leak subtraction

Equation 3: scale = ( V_post_ – V_pre_ ) / V_leak_delta_ ; this scales the leak_ave to the size of the test pulse (V_post_).

Equation 4: I_offset_ = g_leak_ ( V_pre_ – V_leak_ ) ; this offsets the leak_ave to take into account the leak current before the test pulse (V_pre_).

Equation 5: V_leak_delta_ = V_leak_post_ – V_leak_pre_ ; size of the leak voltage pulse.

I_leak_ave_ = g_leak_ ( V – V_leak_ ) ; V_leak_pre_ = V_leak_, V_leak_post_ = V_leak_ – 5 mV

V_leak_delta_ = 5 mV

I_leak_ave_ = g_leak_ * V_leak_delta_

So, at steady state, leak subtracting the leak average, we should get back to equation 1:

I_leak_ave_ * [ scale ] + I_offset_ = g_leak_ * V_leak_delta_ * [ (V_post_ -V_pre_) / V_leak_delta_ ] + g_leak_ (V_pre_ - V_leak_) ; V_leak_delta_ cancels out;

= g_leak_ * (V_post_ – V_pre_) + g_leak_ * (V_pre_ – V_leak_)

= g_leak_ * ( V_post_ – V_pre_ + V_pre_ – V_leak_ )

= g_leak_ * ( V_post_ – V_leak_ ) ; equation 1 when V = V_post_
