# Supplementary material for: Early-Life Resource Scarcity in Mice Does Not Alter Adult Corticosterone or Preovulatory Luteinizing Hormone Surge Responses to Acute Psychosocial Stress
Source: eNeuro. 2024 Jul 26;11(7):ENEURO.0125-24.2024. doi: 10.1523/ENEURO.0125-24.2024 (PMC11287788; doi:10.1523/ENEURO.0125-24.2024)

DeFazio 20150219

Ramp analysis panel

(place rampanalysis v1_0.ipf into the igor procs folder)

This tool allows you to select a fit region to subtract a linear correction to a ramp trace. New traces are created which contain the subtracted data. These use the same trace names as the originals with “_sub” appended to the name.

Begin by placing all of the ramp data you wish to analyze into “Analysis Graph 1”. If you use a single pgf in an experiment, you can quickly import these by selecting the appropriate item in the labels drop down list at the bottom left of the “blast panel”. And these imported waves to the Analysis Graph 1 by shift-clicking each one.


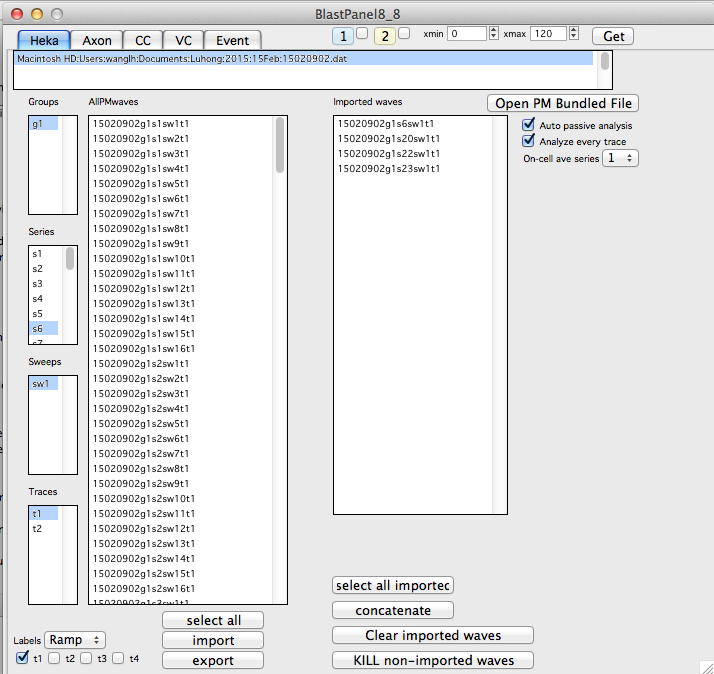


Make Analysis Graph 1 the top graph. Check that no duplicate waves are plotted (use the “graph/remove from graph” menu item to check.

Create the ramp analysis panel by running the ramps macro (type ramps() at the command line or select from macro menu).

If you click Fit without specifying cursors, cursors will automatically be set to 0.1 and 1 sec and this range will be used for the linear fit.

You can specify the fit region ahead of time by placing the cursors A and B at the start and end of the desired region, then click Fit. You can repeat the fit with new settings by clicking Fit again after making adjustments.

Clicking the smooth buttons will apply 10 point box car smoothing to all traces in the appropriate window.

To change the traces please close the ramppanel, add or remove the appropriate traces to “Analysis Graph 1”, then re-run the ramps() macro.


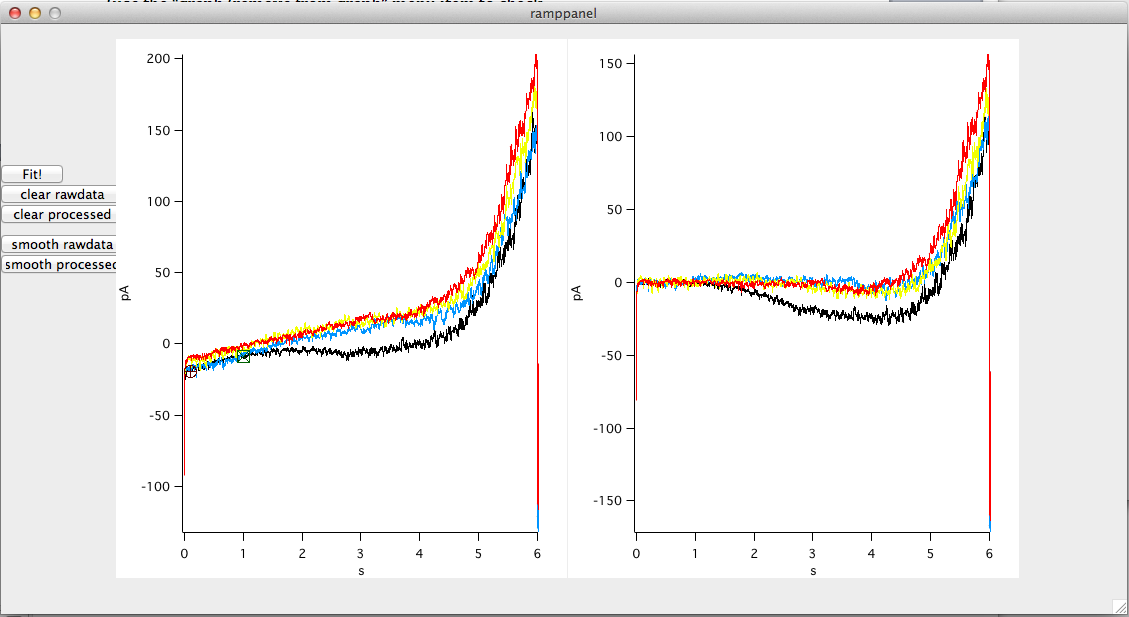

Supplement: Extended Data — Zip file of custom code for PSC detection and analysis, ffmpeg recording of dam behavior, and R analysis. Download Extended Data, ZIP file. [file eneuro-11-ENEURO.0125-24.2024-s002.zip › PSC-analysis/documentation/td analysis/ramp analysis v1.docx]
