# Supplementary material for: Early-Life Resource Scarcity in Mice Does Not Alter Adult Corticosterone or Preovulatory Luteinizing Hormone Surge Responses to Acute Psychosocial Stress
Source: eNeuro. 2024 Jul 26;11(7):ENEURO.0125-24.2024. doi: 10.1523/ENEURO.0125-24.2024 (PMC11287788; doi:10.1523/ENEURO.0125-24.2024)
Supplement: Extended Data — Zip file of custom code for PSC detection and analysis, ffmpeg recording of dam behavior, and R analysis. Download Extended Data, ZIP file. [file eneuro-11-ENEURO.0125-24.2024-s002.zip › PSC-analysis/documentation/td analysis/temperature.docx]

Finding temperature in your PatchMaster .dat files

2015-10-16

The latest version of the “td analysis” software is on the lab server (“td analysis 20151015.zip”, in the all users folder, td analysis versions). This version reads the temperature value from the .dat file for each sweep in each series. If the value is not set by you, the default is 20C. To check your temperature for a given sweep, use the “browse waves…” function in the Igor menu:


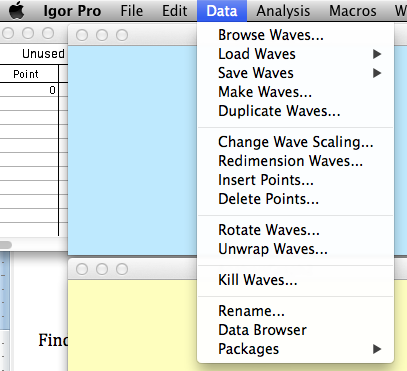


This opens a window that looks like this:


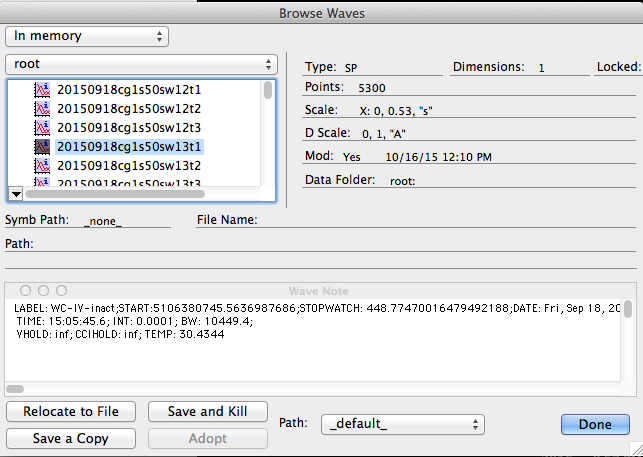


Currently, temperature is the last entry in the Wave Note text near the bottom. These values are accessible for custom routines. If you need these values extracted for graphing purposes, please let me know.
