# Supplementary material for: Early-Life Resource Scarcity in Mice Does Not Alter Adult Corticosterone or Preovulatory Luteinizing Hormone Surge Responses to Acute Psychosocial Stress
Source: eNeuro. 2024 Jul 26;11(7):ENEURO.0125-24.2024. doi: 10.1523/ENEURO.0125-24.2024 (PMC11287788; doi:10.1523/ENEURO.0125-24.2024)
Supplement: Extended Data — Zip file of custom code for PSC detection and analysis, ffmpeg recording of dam behavior, and R analysis. Download Extended Data, ZIP file. [file eneuro-11-ENEURO.0125-24.2024-s002.zip › PSC-analysis/documentation/td analysis/Using the scale bar macro.docx]

Using the scale bar macro

1. Finalize your graph so the x- and y-axis scales are appropriate for your figure.


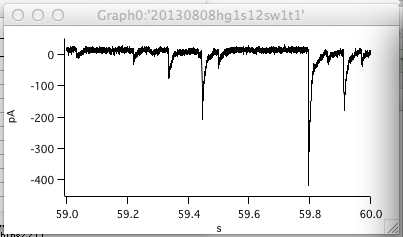


2. Select the “scalebar1” macro from the Macro menu list.


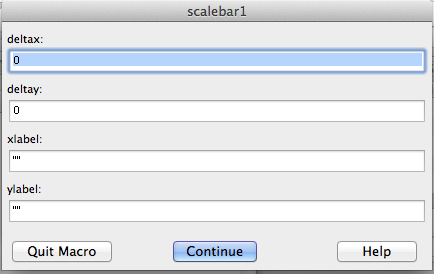


3. Enter the size of the x-axis scale bar in native units of the data (e.g. secs). In this case 0.1 for 0.1 sec.

4. Enter the size of the y-axis scale bar in native units (e.g. amps). In this case 100e-12 for 100 pA.

5. Enter the desired label for the x and y axes. You must match these with your actual scale sizes. In this case 100 msec and 100 pA. There is no error checking in this routine.


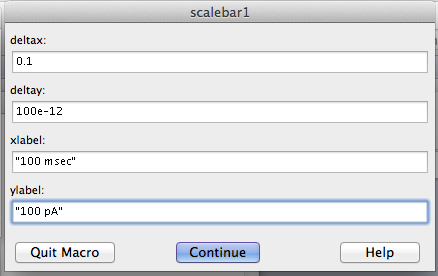

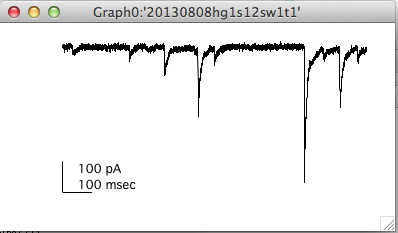


6. Double check your results!
