# Supplementary material for: Early-Life Resource Scarcity in Mice Does Not Alter Adult Corticosterone or Preovulatory Luteinizing Hormone Surge Responses to Acute Psychosocial Stress
Source: eNeuro. 2024 Jul 26;11(7):ENEURO.0125-24.2024. doi: 10.1523/ENEURO.0125-24.2024 (PMC11287788; doi:10.1523/ENEURO.0125-24.2024)
Supplement: Table 1-1 — Number of dams in each group for studies in Figure 1. Dam behavior includes values for number of nest exits per hour and percentage of time spent off the nest. Download Table 1-1, DOCX file. [file eneuro-11-ENEURO.0125-24.2024-s003.docx]

**Table 1-1.** Number of dams in each group for studies in Figure 1. Dam behavior includes values for number of nest exits per hour and percentage of time spent off the nest.

|  | | # of dam measurements on postnatal day | | | | | | | | |
| --- | --- | --- | --- | --- | --- | --- | --- | --- | --- | --- |
| variable | treatment | 4 | 5 | 6 | 7 | 8 | 9 | 10 | 11 | 21 |
| dam mass | STD | 25 |  |  |  |  |  |  | 25 | 25 |
|  | LBN | 24 |  |  |  |  |  |  | 24 | 24 |
| dam corticosterone | STD |  |  |  |  |  |  |  | 24 |  |
|  | LBN |  |  |  |  |  |  |  | 24 |  |
| dam behavior | STD | 23 | 24 | 25 | 19 | 19 | 19 | 19 | 19 |  |
|  | LBN | 22 | 23 | 24 | 20 | 20 | 20 | 20 | 19 |  |
