# Supplementary material for: Early-Life Resource Scarcity in Mice Does Not Alter Adult Corticosterone or Preovulatory Luteinizing Hormone Surge Responses to Acute Psychosocial Stress
Source: eNeuro. 2024 Jul 26;11(7):ENEURO.0125-24.2024. doi: 10.1523/ENEURO.0125-24.2024 (PMC11287788; doi:10.1523/ENEURO.0125-24.2024)
Supplement: Table 1-2 — Statistics for tests of dam mass and dam behavior over time. Linear mixed model and pairwise comparisons of the dam mass on PND4, 11, and 21 was fit with the equation mass ∼ early-life treatment * PND@ + (1 | dam). Postnatal day (PND) was treated as a factor variable. Dam behavior parameters were analyzed with a nonparametric longitudinal model using the nparLD package in R, with the F1 LD F1 Model. The subject variable was each dam, early-life treatment (STD or LBN cage) was the between-subject factor (‘whole-plot’ factor), and PND was the within-subject factor (‘sub-plot’ repeated factor). Download Table 1-2, DOCX file. [file eneuro-11-ENEURO.0125-24.2024-s004.docx]

**Table 1-2.** Statistics for tests of dam mass and dam behavior over time. Linear mixed model and pairwise comparisons of the dam mass on PND4, 11, and 21 was fit with the equation mass ~ early-life treatment * PND@ + (1 | dam). Postnatal day (PND) was treated as a factor variable. Dam behavior parameters were analyzed with a nonparametric longitudinal model using the nparLD package in R, with the F1 LD F1 Model. The subject variable was each dam, early-life treatment (STD or LBN cage) was the between-subject factor ('whole-plot' factor), and PND was the within-subject factor ('sub-plot' repeated factor).

|  | early-life treatment | | | PND | | | early-life treatment * PND | | |
| --- | --- | --- | --- | --- | --- | --- | --- | --- | --- |
| feature | F | df | p | F | df | p | F | df | p |
| dam mass | 4.83 | 1, 47.0 | 0.033 | 82.02 | 2, 94.0 | <0.001 | 1.69 | 2, 94.0 | 0.189 |
| # of exits | 19.49 | 1.0 | <0.001 | 12.33 | 5.7 | <0.001 | 1.11 | 5.7 | 0.356 |
| % off nest | 2.01 | 1.0 | 0.156 | 0.90 | 5.6 | 0.490 | 1.52 | 5.6 | 0.171 |
