# Supplementary material for: Early-Life Resource Scarcity in Mice Does Not Alter Adult Corticosterone or Preovulatory Luteinizing Hormone Surge Responses to Acute Psychosocial Stress
Source: eNeuro. 2024 Jul 26;11(7):ENEURO.0125-24.2024. doi: 10.1523/ENEURO.0125-24.2024 (PMC11287788; doi:10.1523/ENEURO.0125-24.2024)
Supplement: Table 2-1 — Linear mixed models of offspring mass on PND11 and anogenital distance. Equations were outcome ∼ early-life treatment * sex + (1 | dam). Early-life treatment is STD vs LBN rearing. Sex is males vs females. Download Table 2-1, DOCX file. [file eneuro-11-ENEURO.0125-24.2024-s005.docx]

**Table 2-1**. Linear mixed models of offspring mass on PND11 and anogenital distance. Equations were outcome ~ early-life treatment * sex + (1 | dam). Early-life treatment is STD vs LBN rearing. Sex is males vs females.

|  | early-life treatment | | | sex | | | early-life treatment * sex | | |
| --- | --- | --- | --- | --- | --- | --- | --- | --- | --- |
| feature | F | df | p | F | df | p | F | df | p |
| PND11 mass | 8.26 | 1, 47.0 | 0.006 | 0.25 | 1, 249.7 | 0.616 | 2.38 | 1, 249.7 | 0.124 |
| anogenital distance | 1.32 | 1, 46.5 | 0.257 | 25763.19 | 1, 239.8 | <0.001 | 0.01 | 1, 239.8 | 0.940 |
