# Supplementary material for: Early-Life Resource Scarcity in Mice Does Not Alter Adult Corticosterone or Preovulatory Luteinizing Hormone Surge Responses to Acute Psychosocial Stress
Source: eNeuro. 2024 Jul 26;11(7):ENEURO.0125-24.2024. doi: 10.1523/ENEURO.0125-24.2024 (PMC11287788; doi:10.1523/ENEURO.0125-24.2024)
Supplement: Table 2-2 — Linear mixed model of the offspring mass from PND11-72 fit with the equation mass ∼ early-life treatment * PND@ + (1 | dam) + (1 | mouse). Early-life treatment is STD vs LBN rearing. PND@: Linear splines at PND21 and 35 allow the model to change the slope of the line for the segments between PND11-21, from 21-35, and from 35-72. Male and female offspring were fit with separate models. Download Table 2-2, DOCX file. [file eneuro-11-ENEURO.0125-24.2024-s006.docx]

**Table 2-2.** Linear mixed model of the offspring mass from PND11-72 fit with the equation mass ~ early-life treatment * PND@ + (1 | dam) + (1 | mouse). Early-life treatment is STD vs LBN rearing. PND@: Linear splines at PND21 and 35 allow the model to change the slope of the line for the segments between PND11-21, from 21-35, and from 35-72. Male and female offspring were fit with separate models.

|  | females | | | males | | |
| --- | --- | --- | --- | --- | --- | --- |
| variable | F | df | p | F | df | p |
| early-life treatment | 0.78 | 1, 76.8 | 0.381 | 3.37 | 1, 69.4 | 0.071 |
| PND@ | 59283.03 | 3, 3229.4 | <0.001 | 68522.70 | 3, 2619.4 | <0.001 |
| early-life treatment * PND@ | 0.85 | 3, 3229.4 | 0.466 | 6.84 | 3, 2619.4 | <0.001 |
