# Supplementary material for: Early-Life Resource Scarcity in Mice Does Not Alter Adult Corticosterone or Preovulatory Luteinizing Hormone Surge Responses to Acute Psychosocial Stress
Source: eNeuro. 2024 Jul 26;11(7):ENEURO.0125-24.2024. doi: 10.1523/ENEURO.0125-24.2024 (PMC11287788; doi:10.1523/ENEURO.0125-24.2024)
Supplement: Table 2-3 — Linear mixed models for maturation with the equation maturation feature ∼ early-life treatment + (1 | dam). Early-life treatment is STD vs LBN rearing. Models were fit for age and for mass at vaginal opening, first estrus, and preputial separation. Download Table 2-3, DOCX file. [file eneuro-11-ENEURO.0125-24.2024-s007.docx]

**Table 2-3.** Linear mixed models for maturation with the equation maturation feature ~ early-life treatment + (1 | dam). Early-life treatment is STD vs LBN rearing. Models were fit for age and for mass at vaginal opening, first estrus, and preputial separation.

|  | effect of early-life treatment | | | | | |
| --- | --- | --- | --- | --- | --- | --- |
|  | age | | | mass | | |
|  | F | df | p | F | df | p |
| vaginal opening | 1.57 | 1, 43.6 | 0.217 | 0.10 | 1, 41.2 | 0.754 |
| first estrus | 1.54 | 1, 43.7 | 0.221 | 0.10 | 1, 41.4 | 0.758 |
| preputial separation | 1.91 | 1, 30.9 | 0.177 | 0.04 | 1, 30.7 | 0.846 |
