# Supplementary material for: Early-Life Resource Scarcity in Mice Does Not Alter Adult Corticosterone or Preovulatory Luteinizing Hormone Surge Responses to Acute Psychosocial Stress
Source: eNeuro. 2024 Jul 26;11(7):ENEURO.0125-24.2024. doi: 10.1523/ENEURO.0125-24.2024 (PMC11287788; doi:10.1523/ENEURO.0125-24.2024)
Supplement: Table 3-1 — Statistics for estrous cycles from postnatal days 70-90. The number of cycles was fit with the linear mixed model equation # of cycles ∼ early-life treatment + (1 | dam). The log of the mean cycle length in days was fit with equation log10(cycle length) ∼ early-life treatment + (1 | dam). Early-life treatment is STD vs LBN rearing. The number of days spent in each cycle stage was assessed with a Chi-squared test (n = 3066). Download Table 3-1, DOCX file. [file eneuro-11-ENEURO.0125-24.2024-s008.docx]

**Table 3-1.** Statistics for estrous cycles from postnatal days 70-90. The number of cycles was fit with the linear mixed model equation # of cycles ~ early-life treatment + (1 | dam). The log of the mean cycle length in days was fit with equation log_10_(cycle length) ~ early-life treatment + (1 | dam). Early-life treatment is STD vs LBN rearing. The number of days spent in each cycle stage was assessed with a Chi-squared test (n = 3066).

|  | # cycles | | | mean cycle length | | | Cycle stage distribution | | |
| --- | --- | --- | --- | --- | --- | --- | --- | --- | --- |
| variable | F | df | p | F | df | p | Chi-sq | df | p |
| early-life treatment | 0.86 | 1, 37.3 | 0.359 | 0.56 | 1, 37.2 | 0.457 | 0.29 | 2 | 0.865 |
