# Supplementary material for: Early-Life Resource Scarcity in Mice Does Not Alter Adult Corticosterone or Preovulatory Luteinizing Hormone Surge Responses to Acute Psychosocial Stress
Source: eNeuro. 2024 Jul 26;11(7):ENEURO.0125-24.2024. doi: 10.1523/ENEURO.0125-24.2024 (PMC11287788; doi:10.1523/ENEURO.0125-24.2024)
Supplement: Figure 4-1 — The ALPS paradigm caused small changes in tissue mass in males. Individual values and model mean ± SEM for A. morning body mass; B. percent change in body mass after adult treatment; C. adrenal mass; D. normalized adrenal mass; E. seminal vesicle mass; F. normalized seminal vesicle mass; G. testicular mass; and H. normalized testicular mass. Some error bars obscured by mean lines. * p < 0.05, ** p < 0.01, *** p < 0.001. Numbers are in Table 4-3. Results from the full statistical models are in Table 4-4. Abbreviations: STD, standard-reared; LBN, limited bedding and nesting; CON, adult control; ALPS, acute, layered, psychosocial stress in adulthood. Download Figure 4-1, TIF file. [file eneuro-11-ENEURO.0125-24.2024-s021.docx]

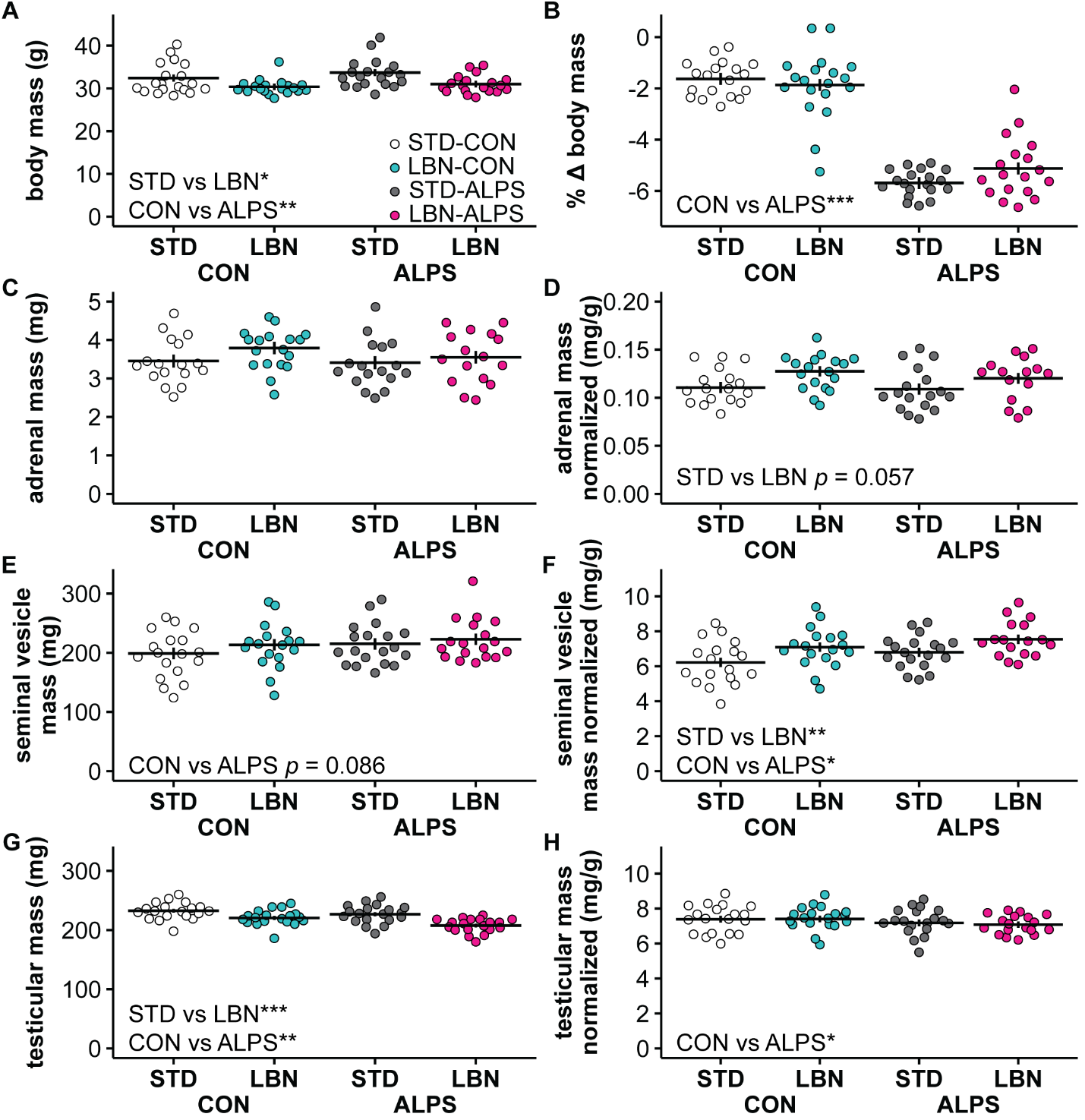
 **Figure 4-1**. The ALPS paradigm caused small changes in tissue mass in males. Individual values and model mean±SEM for **A**. morning body mass; **B**. percent change in body mass after adult treatment; **C**. adrenal mass; **D**. normalized adrenal mass; **E**. seminal vesicle mass; **F**. normalized seminal vesicle mass; **G**. testicular mass; and **H**. normalized testicular mass. Some error bars obscured by mean lines. * *p* < 0.05, ** *p* < 0.01, *** *p* < 0.001. Numbers are in Table 4-3. Results from the full statistical models are in Table 4-4. Abbreviations: STD, standard-reared; LBN, limited bedding and nesting; CON, adult control; ALPS, acute, layered, psychosocial stress in adulthood.
