# Supplementary material for: Early-Life Resource Scarcity in Mice Does Not Alter Adult Corticosterone or Preovulatory Luteinizing Hormone Surge Responses to Acute Psychosocial Stress
Source: eNeuro. 2024 Jul 26;11(7):ENEURO.0125-24.2024. doi: 10.1523/ENEURO.0125-24.2024 (PMC11287788; doi:10.1523/ENEURO.0125-24.2024)
Supplement: Figure 4-2 — LBN and ALPS cause limited changes in tissue masses in females. Individual values and model mean ± SEM for A. morning body mass; B. percent change in body mass after adult treatment. C. adrenal mass; D. normalized adrenal mass; E. uterine mass; F. normalized uterine mass in diestrous (left) and proestrous (right) females. Some error bars obscured by mean lines. * p < 0.05, ** p < 0.01, *** p < 0.001. Numbers are in Table 4-7. Results for the full statistical models are in Table 4-8. Abbreviations: STD, standard-reared; LBN, limited bedding and nesting; CON, adult control; ALPS, acute, layered, psychosocial stress in adulthood. Download Figure 4-2, TIF file. [file eneuro-11-ENEURO.0125-24.2024-s022.docx]

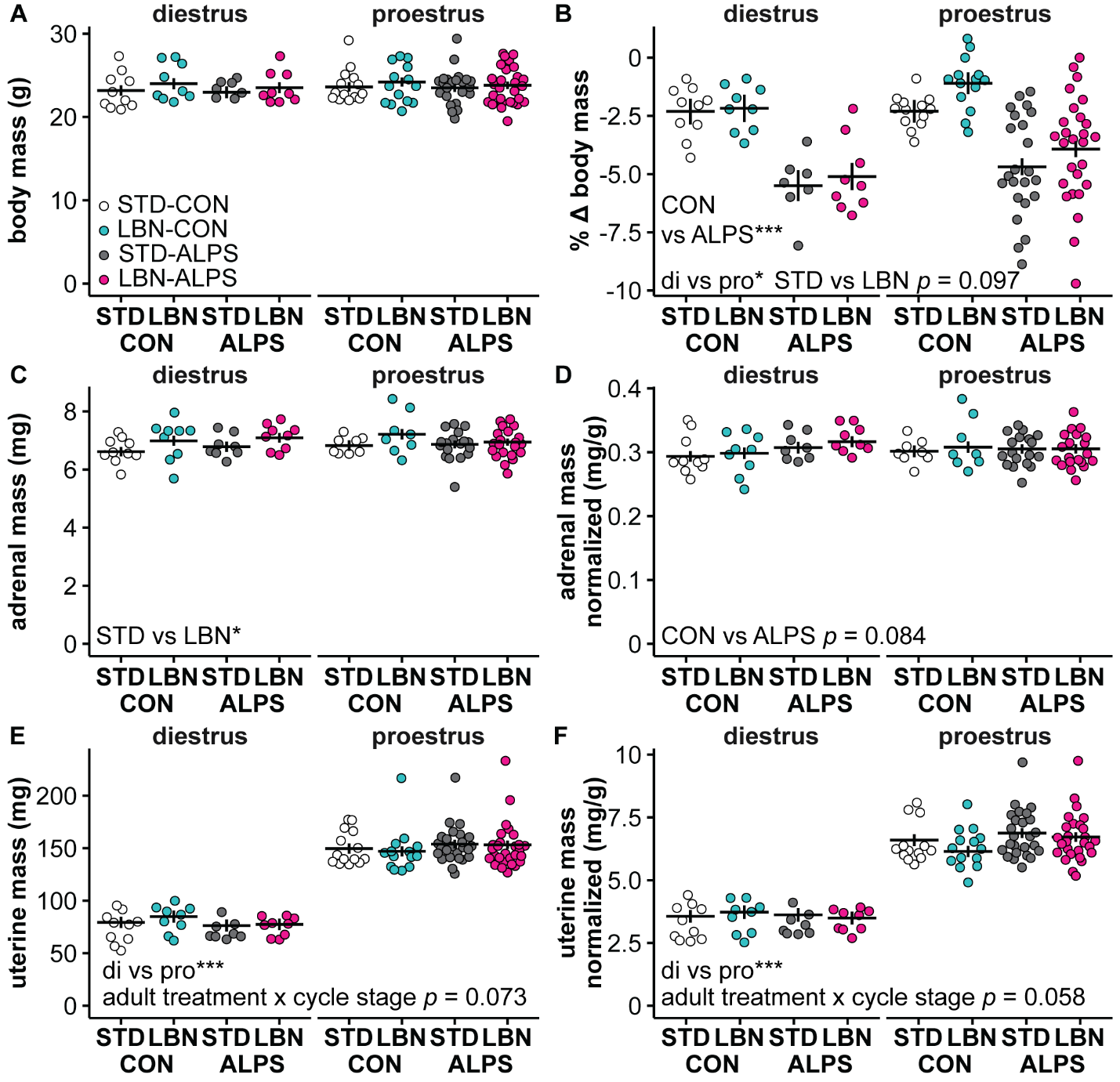


**Figure 4-2.** LBN and ALPS cause limited changes in tissue masses in females. Individual values and model mean±SEM for **A**. morning body mass; **B**. percent change in body mass after adult treatment. **C**. adrenal mass; **D**. normalized adrenal mass; **E**. uterine mass; **F**. normalized uterine mass in diestrous (left) and proestrous (right) females. Some error bars obscured by mean lines. * *p* < 0.05, ** *p* < 0.01, *** *p* < 0.001. Numbers are in Table 4-7. Results for the full statistical models are in Table 4-8. Abbreviations: STD, standard-reared; LBN, limited bedding and nesting; CON, adult control; ALPS, acute, layered, psychosocial stress in adulthood.
