# Supplementary material for: Early-Life Resource Scarcity in Mice Does Not Alter Adult Corticosterone or Preovulatory Luteinizing Hormone Surge Responses to Acute Psychosocial Stress
Source: eNeuro. 2024 Jul 26;11(7):ENEURO.0125-24.2024. doi: 10.1523/ENEURO.0125-24.2024 (PMC11287788; doi:10.1523/ENEURO.0125-24.2024)
Supplement: Figure 4-3 — Acute elevation of serum corticosterone decreases testicular mass in males. Individual and model mean ± SEM for A. morning body mass; B. serum corticosterone concentrations; comparisons between 0 and 2 mg/kg treatment at each hour; C. percent change in body mass after adult treatment. D. adrenal mass; E. normalized adrenal mass; F. seminal vesicle mass; G. normalized seminal vesicle mass; H. testicular mass; and I. normalized testicular mass. Some error bars are obscured by mean line. Vehicle (0 mg/kg): 11 litters and 19 mice, except for adrenal mass with 18 mice; corticosterone (2 mg/kg): 11 litters and 17 mice. * p < 0.05, ** p < 0.01, *** p < 0.001. Results for the full statistical models are in Tables 4-5 and 4-6. Download Figure 4-3, TIF file. [file eneuro-11-ENEURO.0125-24.2024-s023.docx]

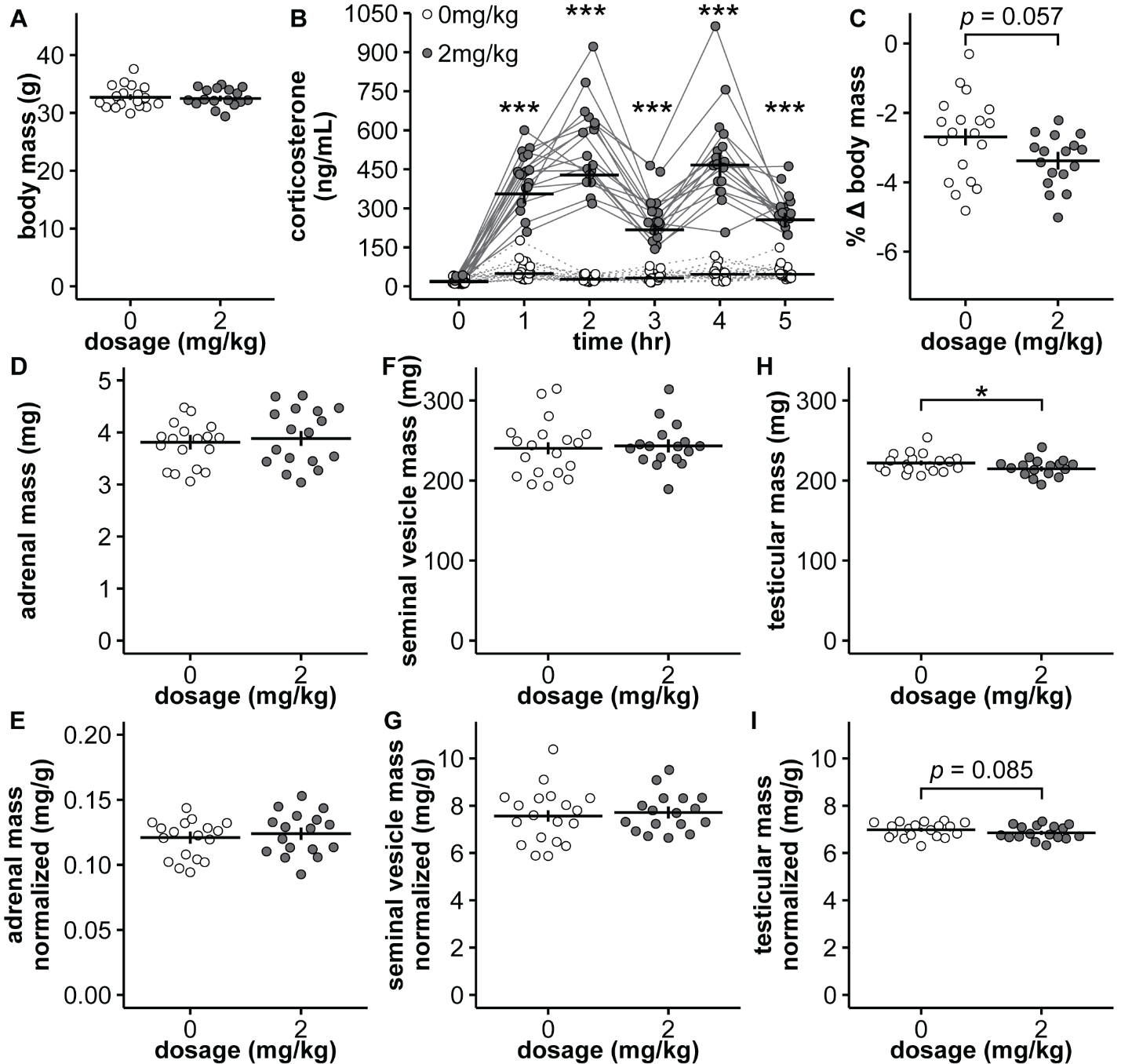


**Figure 4-3.** Acute elevation of serum corticosterone decreases testicular mass in males. Individual and model mean±SEM for **A**. morning body mass; **B**. serum corticosterone concentrations; comparisons between 0 and 2 mg/kg treatment at each hour; **C**. percent change in body mass after adult treatment. **D**. adrenal mass; **E**. normalized adrenal mass; **F**. seminal vesicle mass; **G**. normalized seminal vesicle mass; **H**. testicular mass; and **I**. normalized testicular mass. Some error bars are obscured by mean line. Vehicle (0mg/kg): 11 litters and 19 mice, except for adrenal mass with 18 mice; corticosterone (2mg/kg): 11 litters and 17 mice. * *p* < 0.05, ** *p* < 0.01, *** *p* < 0.001. Results for the full statistical models are in Tables 4-5 and 4-6.
