# Supplementary material for: Early-Life Resource Scarcity in Mice Does Not Alter Adult Corticosterone or Preovulatory Luteinizing Hormone Surge Responses to Acute Psychosocial Stress
Source: eNeuro. 2024 Jul 26;11(7):ENEURO.0125-24.2024. doi: 10.1523/ENEURO.0125-24.2024 (PMC11287788; doi:10.1523/ENEURO.0125-24.2024)
Supplement: Table 4-1 — Number of litters and mice with serum corticosterone measurements before and after adult treatment. Download Table 4-1, DOCX file. [file eneuro-11-ENEURO.0125-24.2024-s009.docx]

**Table 4-1**. Number of litters and mice with serum corticosterone measurements before and after adult treatment.

|  | | STD | | | | LBN | | | |
| --- | --- | --- | --- | --- | --- | --- | --- | --- | --- |
|  | | CON | | ALPS | | CON | | ALPS | |
|  | time | litters | mice | litters | mice | litters | mice | litters | mice |
| male | pre | 11 | 20 | 11 | 20 | 13 | 19 | 13 | 19 |
|  | post | 11 | 19 | 11 | 20 | 13 | 19 | 13 | 19 |
| diestrus | pre | 9 | 10 | 8 | 8 | 7 | 9 | 9 | 9 |
|  | post | 9 | 10 | 8 | 8 | 7 | 9 | 9 | 9 |
| proestrus | pre | 11 | 14 | 17 | 25 | 12 | 14 | 19 | 28 |
|  | post | 11 | 14 | 17 | 25 | 12 | 14 | 19 | 28 |
