# Supplementary material for: Early-Life Resource Scarcity in Mice Does Not Alter Adult Corticosterone or Preovulatory Luteinizing Hormone Surge Responses to Acute Psychosocial Stress
Source: eNeuro. 2024 Jul 26;11(7):ENEURO.0125-24.2024. doi: 10.1523/ENEURO.0125-24.2024 (PMC11287788; doi:10.1523/ENEURO.0125-24.2024)
Supplement: Table 4-2 — Statistics for serum corticosterone in male and female offspring. Data from males were fit with the linear mixed model equation log10(cort) ∼ early-life treatment * adult treatment * time + (1 | mouse) + (1 | dam). Data from females were fit with the linear mixed model equation log10(cort) ∼ cycle stage * early-life treatment * adult treatment * time + (1 | mouse) + (1 | dam). Cycle stage is diestrus vs proestrus; early-life treatment is STD vs LBN rearing; adult treatment is CON vs ALPS; time is pre (0 h) vs post (5 h). Download Table 4-2, DOCX file. [file eneuro-11-ENEURO.0125-24.2024-s010.docx]

**Table 4-2.** Statistics for serum corticosterone in male and female offspring. Data from males were fit with the linear mixed model equation log_10_(cort) ~ early-life treatment * adult treatment * time + (1 | mouse) + (1 | dam). Data from females were fit with the linear mixed model equation log_10_(cort) ~ cycle stage * early-life treatment * adult treatment * time + (1 | mouse) + (1 | dam). Cycle stage is diestrus vs proestrus; early-life treatment is STD vs LBN rearing; adult treatment is CON vs ALPS; time is pre (0h) vs post (5h).

|  | males | | | females | | |
| --- | --- | --- | --- | --- | --- | --- |
| variable | F | df | p | F | df | p |
| cycle stage |  |  |  | 23.85 | 1, 216.3 | <0.001 |
| early-life treatment | 1.60 | 1, 44.0 | 0.213 | 1.36 | 1, 102.0 | 0.246 |
| adult treatment | 0.02 | 1, 121.7 | 0.883 | 0.03 | 1, 200.2 | 0.867 |
| time | 356.09 | 1, 73.6 | <0.001 | 539.59 | 1, 109.0 | <0.001 |
| cycle stage * early-life treatment |  |  |  | 0.18 | 1, 216.3 | 0.672 |
| cycle stage * adult treatment |  |  |  | 3.06 | 1, 212.1 | 0.081 |
| early-life treatment * adult treatment | 0.01 | 1, 121.7 | 0.943 | 0.53 | 1, 200.2 | 0.468 |
| cycle stage * time |  |  |  | 9.65 | 1, 109.0 | 0.002 |
| early-life treatment * time | 1.39 | 1, 73.6 | 0.241 | 0.83 | 1, 109.0 | 0.364 |
| adult treatment * time | 42.80 | 1, 73.6 | <0.001 | 37.79 | 1, 109.0 | <0.001 |
| cycle stage * early-life treatment * adult treatment |  |  |  | 0.00 | 1, 212.1 | 0.975 |
| cycle stage * early-life treatment * time |  |  |  | 0.38 | 1, 109.0 | 0.539 |
| cycle stage * adult treatment * time |  |  |  | 3.95 | 1, 109.0 | 0.049 |
| early-life treatment * adult treatment * time | 0.07 | 1, 73.6 | 0.791 | 0.22 | 1, 109.0 | 0.643 |
| cycle stage * early-life treatment * adult treatment * time |  |  |  | 0.02 | 1, 109.0 | 0.878 |
