# Supplementary material for: Early-Life Resource Scarcity in Mice Does Not Alter Adult Corticosterone or Preovulatory Luteinizing Hormone Surge Responses to Acute Psychosocial Stress
Source: eNeuro. 2024 Jul 26;11(7):ENEURO.0125-24.2024. doi: 10.1523/ENEURO.0125-24.2024 (PMC11287788; doi:10.1523/ENEURO.0125-24.2024)
Supplement: Table 4-3 — Number of litters and male mice with mass measurements on the day of adult treatment. Lower numbers for some tissue masses are attributable to loss of or damage to tissue at dissection. Download Table 4-3, DOCX file. [file eneuro-11-ENEURO.0125-24.2024-s011.docx]

**Table 4-3.** Number of litters and male mice with mass measurements on the day of adult treatment. Lower numbers for some tissue masses are attributable to loss of or damage to tissue at dissection.

|  | STD | | | | LBN | | | |
| --- | --- | --- | --- | --- | --- | --- | --- | --- |
|  | CON | | ALPS | | CON | | ALPS | |
| feature | litters | mice | litters | mice | litters | mice | litters | mice |
| AM body mass (g) | 11 | 19 | 11 | 19 | 13 | 19 | 13 | 19 |
| % change body mass | 11 | 19 | 11 | 19 | 13 | 19 | 13 | 18 |
| adrenal mass (mg) | 10 | 17 | 10 | 17 | 12 | 18 | 11 | 16 |
| adrenal mass normalized to PM mass (mg/g) | 10 | 17 | 10 | 17 | 12 | 18 | 11 | 15 |
| seminal vesicle mass (mg) | 11 | 18 | 11 | 19 | 13 | 18 | 13 | 18 |
| seminal vesicle mass normalized to PM mass (mg/g) | 11 | 18 | 11 | 19 | 13 | 18 | 13 | 17 |
| testicular mass (mg) | 11 | 19 | 11 | 19 | 13 | 19 | 13 | 19 |
| testicular mass normalized to PM mass (mg/g) | 11 | 19 | 11 | 19 | 13 | 19 | 13 | 18 |
