# Supplementary material for: Early-Life Resource Scarcity in Mice Does Not Alter Adult Corticosterone or Preovulatory Luteinizing Hormone Surge Responses to Acute Psychosocial Stress
Source: eNeuro. 2024 Jul 26;11(7):ENEURO.0125-24.2024. doi: 10.1523/ENEURO.0125-24.2024 (PMC11287788; doi:10.1523/ENEURO.0125-24.2024)
Supplement: Table 4-4 — Statistics from linear mixed models of male masses on day of adult treatment. Data were fit with the formula feature ∼ early-life treatment * adult treatment + (1 | dam). Early-life treatment is STD vs LBN rearing; adult treatment is CON vs ALPS. Download Table 4-4, DOCX file. [file eneuro-11-ENEURO.0125-24.2024-s012.docx]

**Table 4-4**. Statistics from linear mixed models of male masses on day of adult treatment. Data were fit with the formula feature ~ early-life treatment * adult treatment + (1 | dam). Early-life treatment is STD vs LBN rearing; adult treatment is CON vs ALPS.

|  | Early-life treatment | | | adult treatment | | | early-life treatment * adult treatment | | |
| --- | --- | --- | --- | --- | --- | --- | --- | --- | --- |
| feature | F | df | p | F | df | p | F | df | p |
| AM body mass (g) | 4.95 | 1, 21.9 | 0.037 | 7.57 | 1, 50.7 | 0.008 | 0.78 | 1, 50.7 | 0.381 |
| % change body mass | 0.50 | 1, 20.4 | 0.489 | 255.89 | 1, 56.7 | <0.001 | 3.07 | 1, 56.7 | 0.085 |
| adrenal mass (mg) | 1.30 | 1, 21.2 | 0.267 | 1.80 | 1, 46.0 | 0.186 | 0.87 | 1, 46.0 | 0.356 |
| adrenal mass normalized to PM mass (mg/g) | 4.06 | 1, 21.2 | 0.057 | 1.44 | 1, 45.6 | 0.237 | 0.59 | 1, 45.6 | 0.448 |
| seminal vesicle mass (mg) | 0.95 | 1, 21.4 | 0.341 | 3.07 | 1, 49.4 | 0.086 | 0.22 | 1, 49.4 | 0.643 |
| seminal vesicle mass normalized to PM mass (mg/g) | 8.47 | 1, 20.6 | 0.008 | 4.12 | 1, 52.4 | 0.048 | 0.06 | 1, 52.4 | 0.804 |
| testicular mass (mg) | 18.95 | 1, 20.8 | <0.001 | 9.43 | 1, 55.0 | 0.003 | 1.35 | 1, 55.0 | 0.250 |
| testicular mass normalized to PM mass (mg/g) | 0.03 | 1, 21.7 | 0.862 | 5.63 | 1, 50.6 | 0.021 | 0.25 | 1, 50.6 | 0.622 |
