# Supplementary material for: Early-Life Resource Scarcity in Mice Does Not Alter Adult Corticosterone or Preovulatory Luteinizing Hormone Surge Responses to Acute Psychosocial Stress
Source: eNeuro. 2024 Jul 26;11(7):ENEURO.0125-24.2024. doi: 10.1523/ENEURO.0125-24.2024 (PMC11287788; doi:10.1523/ENEURO.0125-24.2024)
Supplement: Table 4-5 — Number of litters and female mice with mass measurements on the day of adult treatment. Adrenal masses were not collected from females used for electrophysiology studies (Figures 6-7). Lower numbers for some tissue masses are attributable to loss of or damage to tissue at dissection. Download Table 4-5, DOCX file. [file eneuro-11-ENEURO.0125-24.2024-s013.docx]

**Table 4-5**. Number of litters and female mice with mass measurements on the day of adult treatment. Adrenal masses were not collected from females used for electrophysiology studies (Figures 6-7). Lower numbers for some tissue masses are attributable to loss of or damage to tissue at dissection.

|  | | STD | | | | LBN | | | |
| --- | --- | --- | --- | --- | --- | --- | --- | --- | --- |
|  | | CON | | ALPS | | CON | | ALPS | |
| cycle stage | feature | litters | mice | litters | mice | litters | mice | litters | mice |
| diestrus | AM body mass (g) | 9 | 10 | 7 | 7 | 7 | 9 | 9 | 9 |
|  | % change body mass | 9 | 10 | 7 | 7 | 7 | 9 | 9 | 9 |
|  | adrenal mass (mg) | 9 | 10 | 8 | 8 | 7 | 9 | 9 | 9 |
|  | adrenal mass normalized to PM mass (mg/g) | 9 | 10 | 8 | 8 | 7 | 9 | 9 | 9 |
|  | uterine mass (mg) | 9 | 10 | 8 | 8 | 7 | 9 | 9 | 9 |
|  | uterine mass normalized to PM mass (mg/g) | 9 | 10 | 8 | 8 | 7 | 9 | 9 | 9 |
| proestrus | AM body mass (g) | 11 | 14 | 15 | 23 | 12 | 14 | 19 | 28 |
|  | % change body mass | 10 | 13 | 15 | 23 | 12 | 14 | 18 | 27 |
|  | adrenal mass (mg) | 7 | 8 | 12 | 18 | 8 | 8 | 14 | 21 |
|  | adrenal mass normalized to PM mass (mg/g) | 7 | 8 | 12 | 18 | 8 | 8 | 13 | 20 |
|  | uterine mass (mg) | 11 | 14 | 17 | 25 | 12 | 14 | 19 | 28 |
|  | uterine mass normalized to PM mass (mg/g) | 10 | 13 | 17 | 25 | 12 | 14 | 18 | 27 |
