# Supplementary material for: Early-Life Resource Scarcity in Mice Does Not Alter Adult Corticosterone or Preovulatory Luteinizing Hormone Surge Responses to Acute Psychosocial Stress
Source: eNeuro. 2024 Jul 26;11(7):ENEURO.0125-24.2024. doi: 10.1523/ENEURO.0125-24.2024 (PMC11287788; doi:10.1523/ENEURO.0125-24.2024)
Supplement: Table 4-6 — Statistics from linear mixed models of female masses on day of adult treatment. Data were fit with the formula feature ∼ early-life treatment * adult treatment * cycle stage + (1 | dam). Early-life treatment is STD vs LBN rearing; adult treatment is CON vs ALPS; cycle stage is diestrus vs proestrus. Download Table 4-6, DOCX file. [file eneuro-11-ENEURO.0125-24.2024-s014.docx]

**Table 4-6.** Statistics from linear mixed models of female masses on day of adult treatment. Data were fit with the formula feature ~ early-life treatment * adult treatment * cycle stage + (1 | dam). Early-life treatment is STD vs LBN rearing; adult treatment is CON vs ALPS; cycle stage is diestrus vs proestrus.

| feature | variable | F | df | p |
| --- | --- | --- | --- | --- |
| AM body mass (g) | early-life treatment | 0.96 | 1, 43.5 | 0.333 |
|  | adult treatment | 0.78 | 1, 77.9 | 0.380 |
|  | cycle stage | 1.02 | 1, 86.0 | 0.315 |
|  | early-life treatment * adult treatment | 0.16 | 1, 77.9 | 0.689 |
|  | early-life treatment * cycle stage | 0.11 | 1, 86.0 | 0.744 |
|  | adult treatment * cycle stage | 0.02 | 1, 83.1 | 0.896 |
|  | early-life treatment * adult treatment * cycle stage | 0.00 | 1, 83.1 | 0.987 |
| % change body mass | early-life treatment | 2.91 | 1, 34.0 | 0.097 |
|  | adult treatment | 60.96 | 1, 93.6 | <0.001 |
|  | cycle stage | 4.41 | 1, 103.9 | 0.038 |
|  | early-life treatment * adult treatment | 0.02 | 1, 93.6 | 0.899 |
|  | early-life treatment * cycle stage | 0.96 | 1, 103.9 | 0.330 |
|  | adult treatment * cycle stage | 0.38 | 1, 101.7 | 0.539 |
|  | early-life treatment * adult treatment * cycle stage | 0.23 | 1, 101.7 | 0.631 |
| adrenal mass (mg) | early-life treatment | 4.20 | 1, 30.6 | 0.049 |
|  | adult treatment | 0.01 | 1, 68.6 | 0.913 |
|  | cycle stage | 0.76 | 1, 67.9 | 0.386 |
|  | early-life treatment * adult treatment | 0.82 | 1, 68.6 | 0.368 |
|  | early-life treatment * cycle stage | 0.25 | 1, 67.9 | 0.617 |
|  | adult treatment * cycle stage | 1.40 | 1, 76.3 | 0.241 |
|  | early-life treatment * adult treatment * cycle stage | 0.32 | 1, 76.3 | 0.575 |
| adrenal mass normalized to PM mass (mg/g) | early-life treatment | 0.37 | 1, 32.5 | 0.547 |
|  | adult treatment | 3.08 | 1, 60.8 | 0.084 |
|  | cycle stage | 0.05 | 1, 61.7 | 0.831 |
|  | early-life treatment * adult treatment | 0.01 | 1, 60.8 | 0.926 |
|  | early-life treatment * cycle stage | 0.15 | 1, 61.7 | 0.696 |
|  | adult treatment * cycle stage | 2.40 | 1, 66.9 | 0.126 |
|  | early-life treatment * adult treatment * cycle stage | 0.28 | 1, 66.9 | 0.601 |
| uterine mass (mg) | early-life treatment | 0.03 | 1, 43.6 | 0.871 |
|  | adult treatment | 0.00 | 1, 77.2 | 0.995 |
|  | cycle stage | 573.41 | 1, 85.5 | <0.001 |
|  | early-life treatment * adult treatment | 0.05 | 1, 77.2 | 0.819 |
|  | early-life treatment * cycle stage | 0.69 | 1, 85.5 | 0.407 |
|  | adult treatment * cycle stage | 3.30 | 1, 83.6 | 0.073 |
|  | early-life treatment * adult treatment * cycle stage | 0.28 | 1, 83.6 | 0.595 |
| uterine mass normalized to PM mass (mg/g) | early-life treatment | 0.34 | 1, 43.3 | 0.565 |
|  | adult treatment | 1.81 | 1, 74.9 | 0.183 |
|  | cycle stage | 467.39 | 1, 84.4 | <0.001 |
|  | early-life treatment * adult treatment | 0.00 | 1, 74.9 | 0.993 |
|  | early-life treatment * cycle stage | 1.39 | 1, 84.4 | 0.242 |
|  | adult treatment * cycle stage | 3.70 | 1, 81.0 | 0.058 |
|  | early-life treatment * adult treatment * cycle stage | 1.21 | 1, 81.0 | 0.274 |
