# Supplementary material for: Early-Life Resource Scarcity in Mice Does Not Alter Adult Corticosterone or Preovulatory Luteinizing Hormone Surge Responses to Acute Psychosocial Stress
Source: eNeuro. 2024 Jul 26;11(7):ENEURO.0125-24.2024. doi: 10.1523/ENEURO.0125-24.2024 (PMC11287788; doi:10.1523/ENEURO.0125-24.2024)
Supplement: Table 4-7 — Statistics from linear mixed models of male masses on day of vehicle (0 mg/kg) or corticosterone (2 mg/kg) treatment. Data were fit with the formula feature ∼ dosage + (1 | dam). Download Table 4-7, DOCX file. [file eneuro-11-ENEURO.0125-24.2024-s015.docx]

**Table 4-7.** Statistics from linear mixed models of male masses on day of vehicle (0mg/kg) or corticosterone (2mg/kg) treatment. Data were fit with the formula feature ~ dosage + (1 | dam).

|  | Dosage | | |
| --- | --- | --- | --- |
| feature | F | df | p |
| AM body mass (g) | 0.28 | 1, 24.5 | 0.602 |
| % change body mass | 3.96 | 1, 27.5 | 0.057 |
| adrenal mass (mg) | 0.55 | 1, 23.4 | 0.466 |
| adrenal mass normalized to PM mass (mg/g) | 0.70 | 1, 23.5 | 0.410 |
| seminal vesicle mass (mg) | 0.07 | 1, 27.5 | 0.790 |
| seminal vesicle mass normalized to PM mass (mg/g) | 0.18 | 1, 27.5 | 0.674 |
| testicular mass (mg) | 7.77 | 1, 24.5 | 0.010 |
| testicular mass normalized to PM mass (mg/g) | 3.22 | 1, 24.5 | 0.085 |
