# Supplementary material for: Early-Life Resource Scarcity in Mice Does Not Alter Adult Corticosterone or Preovulatory Luteinizing Hormone Surge Responses to Acute Psychosocial Stress
Source: eNeuro. 2024 Jul 26;11(7):ENEURO.0125-24.2024. doi: 10.1523/ENEURO.0125-24.2024 (PMC11287788; doi:10.1523/ENEURO.0125-24.2024)
Supplement: Table 4-8 — Statistics for serum corticosterone in males with vehicle or corticosterone administration. Data were fit with the linear mixed model equation log10(cort) ∼ dosage * time + (1 | mouse) + (1 | dam). Dosage is 0 mg/kg vs 2 mg/kg; time compares 0 h, 1 h, 2 h, 3 h, 4 h, and 5 h. Download Table 4-8, DOCX file. [file eneuro-11-ENEURO.0125-24.2024-s016.docx]

**Table 4-8**. Statistics for serum corticosterone in males with vehicle or corticosterone administration. Data were fit with the linear mixed model equation log_10_(cort) ~ dosage * time + (1 | mouse) + (1 | dam). Dosage is 0mg/kg vs 2mg/kg; time compares 0h, 1h, 2h, 3h, 4h, and 5h.

| variable | F | df | p |
| --- | --- | --- | --- |
| dosage | 432.21 | 1, 36.4 | <0.001 |
| time | 178.35 | 5, 245.0 | <0.001 |
| dosage * time | 75.13 | 5, 245.0 | <0.001 |
