# Supplementary material for: Early-Life Resource Scarcity in Mice Does Not Alter Adult Corticosterone or Preovulatory Luteinizing Hormone Surge Responses to Acute Psychosocial Stress
Source: eNeuro. 2024 Jul 26;11(7):ENEURO.0125-24.2024. doi: 10.1523/ENEURO.0125-24.2024 (PMC11287788; doi:10.1523/ENEURO.0125-24.2024)
Supplement: Table 5-1 — Statistics from linear mixed models of average LH in diestrous mice on day of adult treatment. Data were fit with the formula average LH ∼ early-life treatment * adult treatment + (1 | dam). Early-life treatment is STD vs LBN treatment; adult treatment is CON vs ALPS treatment. Download Table 5-1, DOCX file. [file eneuro-11-ENEURO.0125-24.2024-s017.docx]

**Table 5-1.** Statistics from linear mixed models of average LH in diestrous mice on day of adult treatment. Data were fit with the formula average LH ~ early-life treatment * adult treatment + (1 | dam). Early-life treatment is STD vs LBN treatment; adult treatment is CON vs ALPS treatment.

| variable | F | df | p |
| --- | --- | --- | --- |
| early-life treatment | 0.17 | 1, 16.4 | 0.687 |
| adult treatment | 3.69 | 1, 23.1 | 0.067 |
| early-life treatment * adult treatment | 0.44 | 1, 23.1 | 0.515 |
