# Supplementary material for: Early-Life Resource Scarcity in Mice Does Not Alter Adult Corticosterone or Preovulatory Luteinizing Hormone Surge Responses to Acute Psychosocial Stress
Source: eNeuro. 2024 Jul 26;11(7):ENEURO.0125-24.2024. doi: 10.1523/ENEURO.0125-24.2024 (PMC11287788; doi:10.1523/ENEURO.0125-24.2024)
Supplement: Table 6-1 — Statistics from linear mixed models of electrophysiology properties on day of adult treatment. The mean value for each cell was calculated, and data were fit with the formula feature ∼ early-life treatment * adult treatment + (1 | dam) + (1 | mouse). Early-life treatment is STD vs LBN rearing; adult treatment is CON vs ALPS. Download Table 6-1, DOCX file. [file eneuro-11-ENEURO.0125-24.2024-s018.docx]

**Table 6-1.** Statistics from linear mixed models of electrophysiology properties on day of adult treatment. The mean value for each cell was calculated, and data were fit with the formula feature ~ early-life treatment * adult treatment + (1 | dam) + (1 | mouse). Early-life treatment is STD vs LBN rearing; adult treatment is CON vs ALPS.

|  | early-life treatment | | | adult treatment | | | early-life treatment * adult treatment | | |
| --- | --- | --- | --- | --- | --- | --- | --- | --- | --- |
| feature | F | df | p | F | df | p | F | df | p |
| capacitance (pF) | 0.03 | 1, 8.1 | 0.863 | 0.77 | 1, 9.4 | 0.401 | 0.07 | 1, 9.4 | 0.791 |
| input resistance (MOhm) | 0.23 | 1, 8.4 | 0.641 | 1.52 | 1, 9.5 | 0.248 | 1.50 | 1, 9.5 | 0.250 |
| series resistance (MOhm) | 0.30 | 1, 7.8 | 0.601 | 0.97 | 1, 11.3 | 0.346 | 0.27 | 1, 11.3 | 0.613 |
| holding current (pA) | 0.02 | 1, 7.8 | 0.898 | 0.01 | 1, 9.2 | 0.909 | 0.35 | 1, 9.2 | 0.566 |
| interevent interval (pA) | 0.12 | 1, 7.8 | 0.740 | 0.05 | 1, 9.2 | 0.820 | 0.00 | 1, 9.2 | 0.946 |
| amplitude (pA) | 0.45 | 1, 7.0 | 0.524 | 0.72 | 1, 8.8 | 0.420 | 0.12 | 1, 8.8 | 0.737 |
| decay time (ms) | 0.50 | 1, 7.0 | 0.503 | 0.85 | 1, 8.8 | 0.381 | 1.31 | 1, 8.8 | 0.283 |
