# Supplementary material for: Early-Life Resource Scarcity in Mice Does Not Alter Adult Corticosterone or Preovulatory Luteinizing Hormone Surge Responses to Acute Psychosocial Stress
Source: eNeuro. 2024 Jul 26;11(7):ENEURO.0125-24.2024. doi: 10.1523/ENEURO.0125-24.2024 (PMC11287788; doi:10.1523/ENEURO.0125-24.2024)
Supplement: Table 7-1 — Statistics for number of postsynaptic current (PSC) events per 240 s in GnRH neurons on the day of adult treatment. As the frequency data were skewed right and included zeros, a generalized linear mixed effects negative binomial model was used. Data were fit with the model equation # events per 240 s ∼ early-life treatment * adult treatment + (1 | mouse) + (1 | dam). The joint_tests function of the emmeans package was used to obtain these p-value estimates from the model. Early-life treatment is STD vs LBN rearing; adult treatment is CON vs ALPS. Download Table 7-1, DOCX file. [file eneuro-11-ENEURO.0125-24.2024-s019.docx]

**Table 7-1.** Statistics for number of postsynaptic current (PSC) events per 240s in GnRH neurons on the day of adult treatment. As the frequency data were skewed right and included zeros, a generalized linear mixed effects negative binomial model was used. Data were fit with the model equation # events per 240s ~ early-life treatment * adult treatment + (1 | mouse) + (1 | dam). The joint_tests function of the emmeans package was used to obtain these p-value estimates from the model. Early-life treatment is STD vs LBN rearing; adult treatment is CON vs ALPS.

| variable | F ratio | df | Chi-sq | p |
| --- | --- | --- | --- | --- |
| early-life treatment | 0.013 | 1, Inf | 0.013 | 0.910 |
| adult treatment | 1.476 | 1, Inf | 1.476 | 0.224 |
| early-life treatment * adult treatment | 0.067 | 1, Inf | 0.067 | 0.796 |
