# Supplementary material for: Early-Life Resource Scarcity in Mice Does Not Alter Adult Corticosterone or Preovulatory Luteinizing Hormone Surge Responses to Acute Psychosocial Stress
Source: eNeuro. 2024 Jul 26;11(7):ENEURO.0125-24.2024. doi: 10.1523/ENEURO.0125-24.2024 (PMC11287788; doi:10.1523/ENEURO.0125-24.2024)
Supplement: Table 7-2 — Pairwise comparisons of distributions of interevent interval and amplitude for PSCs recorded in GnRH neurons. The Anderson-Darling criterion (AD), the standardized test statistic (T AD), and asymptotic p-value were calculated with the kSamples package (Scholz & Zhu, 2023). Bootstrapping was used to estimate the mean difference. The confidence interval is not adjusted for multiple comparisons. The p-values for both tests were adjusted using Holm’s method for multiple comparisons. Download Table 7-2, DOCX file. [file eneuro-11-ENEURO.0125-24.2024-s020.docx]

**Table 7-2**. Pairwise comparisons of distributions of interevent interval and amplitude for PSCs recorded in GnRH neurons. The Anderson-Darling criterion (AD), the standardized test statistic (T AD), and asymptotic p-value were calculated with the kSamples package (Scholz & Zhu, 2023). Bootstrapping was used to estimate the mean difference. The confidence interval is not adjusted for multiple comparisons. The p-values for both tests were adjusted using Holm’s method for multiple comparisons.

|  | | # PSCs | | Anderson-Darling | | | bootstrap | | |
| --- | --- | --- | --- | --- | --- | --- | --- | --- | --- |
| variable | comparison | group 1 | group 2 | AD | T AD | p | mean diff | 95% CI | p |
| interevent interval (ms) | STD-CON vs STD-ALPS | 4,597 | 2,324 | 531.5 | 696.91 | <0.001 | 0.78 | [0.65, 0.92] | <0.001 |
|  | STD-CON vs LBN-CON | 4,597 | 3,032 | 214.5 | 280.48 | <0.001 | 0.35 | [0.25, 0.46] | <0.001 |
|  | LBN-CON vs LBN-ALPS | 3,032 | 2,217 | 104.0 | 135.40 | <0.001 | 0.32 | [0.19, 0.46] | <0.001 |
|  | STD-ALPS vs LBN-ALPS | 2,324 | 2,217 | 0.5 | -0.65 | 0.744 | -0.11 | [‑0.27, 0.05] | 0.177 |
| amplitude (pA) | STD-CON vs STD-ALPS | 1,787 | 1,849 | 15.5 | 19.02 | <0.001 | 3.60 | [1.36, 5.81] | 0.006 |
|  | STD-CON vs LBN-CON | 1,787 | 1,908 | 3.8 | 3.62 | 0.023 | -1.23 | [-3.36, 0.80] | 0.498 |
|  | LBN-CON vs LBN-ALPS | 1,908 | 1,759 | 2.3 | 1.69 | 0.065 | -0.39 | [-2.42, 1.66] | 0.711 |
|  | STD-ALPS vs LBN-ALPS | 1,849 | 1,759 | 21.1 | 26.37 | <0.001 | -5.22 | [‑7.34, ‑3.09] | <0.001 |
