# Supplementary material for: Early-Life Resource Scarcity in Mice Does Not Alter Adult Corticosterone or Preovulatory Luteinizing Hormone Surge Responses to Acute Psychosocial Stress
Source: eNeuro. 2024 Jul 26;11(7):ENEURO.0125-24.2024. doi: 10.1523/ENEURO.0125-24.2024 (PMC11287788; doi:10.1523/ENEURO.0125-24.2024)
Supplement: Extended Data — Zip file of custom code for PSC detection and analysis, ffmpeg recording of dam behavior, and R analysis. Download Extended Data, ZIP file. [file eneuro-11-ENEURO.0125-24.2024-s002.zip › PSC-analysis/documentation/HEKA PatchMaster PMN fileformats/PatchmasterV1000/FileFormat_v1000.zip!/FileFormat_v1000/DataFormat_v1000.doc]

PatchMaster v2x90.4, 30-Oct-2018

# Data Format

In this chapter we describe the general structure of the files generated by *PatchMAster*.

## Data Files

*PatchMAster* typically generates up to 9 files, when a data file is created:

1. The *Raw Data* file (file extension *.dat).
2. The *Stimulus Templates* file (file extension *.pgf).
3. The *Acquisition Parameters* file (file extension *.pul).
4. The *Amplifiers* file (file extension *.amp).
5. The *Solutions* file (file extension *.sol).
6. The *Notebook* file (file extension *.txt).
7. The *Markers* file (file extension *.mrk).
8. The *Protocol Methods* file (file extension *.mth).
9. The *Analysis Methods* file (file extension *.onl).

The *Raw Data* file is a binary file with a file header.

The files with the extensions *.pul, *.pgf, *.onl, *.sol, and *.amp have a *Tree* structure, see below.

The *Protocol Methods* file is a combined binary and text file.

And the *Notebook* file is a simple text file.

All sub-files are kept in memory, whereas the raw data is always written to disk. All these files are bundled into one file upon closing the data file, when the option “Make Bundle File” (default) is activated.

### 1. Raw Data File

The raw data file has an optional header, followed by a continuous data stream. Each data point is a 16-bit signed integer or 32-bit IEEE real as defined in the respective PGF-template. When a sweep is stored, *PatchMAster* stores the various traces (if available) sequentially as defined in the respective PGF-template. Traces with leak pulses are stored leak-subtracted. The leak traces are normally stored after the traces themselves.

The structure of the *Raw Data file* (extension “dat”) is defined by the file “DataFile_v1000.txt”.

Very long traces with “continuous” segments may be stored as interleaved blocks. The size of such a block is given in the “InterleaveSize” field (in bytes) of the Trace record, the distance to the next block in the “InterleaveSkip” field (in bytes).

### 2. Stimulation Templates File

Stores the stimulation protocol. The structure of the *Stimulation Templates File* (extension “pgf”) is defined by the file “StimFile_v1000.txt”. It has a tree structure:

| **Record** | **Description** |
| --- | --- |
| Root | Version information |
| Stimulation | Description of an ensemble of pulse patterns; e.g., I-V curve |
| Channel | Combines the definition for one output (DAC) and one input (ADC) trace |
| Segment | Individual segment of a pulse pattern |

Stimulation files can be loaded into the *Pulse Generator (Stimulus Editor)*. In fact, the *Pulse Generator* files for the stimulation protocols used during the experiments have the same data structure as the PGF-files, which belong to the recorded data. In this way it is possible to exactly repeat an experiment by using a copy of a PGF-file as *Pulse Generator* file.

### 3. Acquisition Parameters File

Stores parameters, such as gain, capacitance, etc. The pointer to the data stored in the raw data file is also contained in this file. The structure of the *Acquisition Parameters File* (extension “pul”) is defined by the file “PulsedFile_v1000.txt”. It has a tree structure:

| **Record** | **Description** |
| --- | --- |
| Root | Version information |
| Group | Larger section of an experiment; e.g., cell or patch |
| Series | Description of an ensemble of sweeps |
| Sweep | Description of a sweep, i.e. one collection of traces |
| Trace | Description of an individual data trace |

A graphical template of the *Pulsed File* (*Tree*) is shown in the *Replay* window. It contains information necessary to reconstruct the experimental conditions as the data were recorded.

### 4. Amplifiers file

This contains the amplifier state records of each amplifier for every Series. The structure of the *Amplifiers File* (extension “amp”) is defined by the file “AmplTreeFile_v1000.txt”. It has a tree structure:

| **Record** | **Description** |
| --- | --- |
| Root | Version information |
| Series | Collection of all amplifier state records of one Series |
| Amplifier State | The amplifier state record |

### 5. Solutions file

This file is only generated, when the option “Solution Base” is activated. It contains the solution records for every Series. The structure of the *Solutions File* (extension “sol”) is defined by the file “SolutionsFile_v1000.txt”. It has a tree structure:

| **Record** | **Description** |
| --- | --- |
| Root | Version information |
| Solution | Description of one solution |
| Chemical | Description of one chemical compound |

### 6. Markers file

It contains the marker records. The structure of the *Markers File* (extension “mrk”) is defined by the file “MarkerFile_v9.txt”. It has a tree structure:

| **Record** | **Description** |
| --- | --- |
| Root | Version information |
| Marker | Description of one marker event |

### 7. Analysis Methods file

It contains the assigned analysis method records for every Series. The structure of the *Analysis Methods File* (extension “onl”) is defined by the file “AnalysisFile_v11.txt”. It has a tree structure:

| **Record** | **Description** |
| --- | --- |
| Root | Version information |
| Method | Description of one analysis method |
| Function | Description of one analysis function |

### 8. Protocol Methods File

It contains possibly used protocol method records. The structure of the *Protocol Methods File* (extension “mth”) is defined by the file “MethodFile_v9.txt”.

### 9. Notebook File

The notebook file (extension “txt”) is a standard ASCII text file with line breaks

## The Tree Format

The idea of *PatchMaster* is to order the data of an experiment in *Trees*. The trunk of the tree (*Root*) is the main descriptor of a data file (it could, for example, correspond to one cell or the entire experiments of one day). The next level is the *Group*. This level can be defined by the user to identify data that belong together. An example would be to open a new group for each patch. The group may contain several families of records. Such a family (e.g., records of a current–voltage relationship) is called *Series*. The individual records of a family are called *Sweeps*. Finally, each sweep may be composed of *Traces* (at the moment, there are two traces per sweep). The latter are not yet part of the data tree (it is planned to allow for more traces in the future). A copy of this data tree is accessible to the user throughout the experiment (so one has an overview of what was recorded, and one can immediately edit the entries (e.g., discard bad records).

The following is a description of the *Tree* format. *Appendix III* has a source code listing for a program that shows how to scan and load a *Tree* file. The code is commented and can easily be translated to other languages.

An example tree with 4 levels can be diagrammed as follows:

There is only one record of level 0, the root record. The above tree has four levels, the root (level 0), levels 1 and 2, and the leaf nodes (level 3).

The format of a tree stored to a file is as follows:

1. Magic number: 054726565 (hex) = “Tree”
2. Number of levels
3. Level sizes, one per level
4. Tree records, top down, left-to-right. Each record has the format:

- Record contents
- Number of children

All of the values (except the record contents) are INT32 values, i.e., 32-bit (4 bytes) values.

**Note 1**	Check the record sizes in the file headers. The record sizes may differ from what you are expecting, e.g., because the file has been created by an older program version which used fewer fields than it is currently using, or a newer version with additional fields**. You must use the record sizes stored in the files themselves.**

**Note 2**	The “Magic” number will be 065657254 (hex) = “eerT”, if the file was written on an operating system with opposite byte ordering (e.g. written under MacOS, read under Windows). In that case, appropriate byte swapping has to be performed (highest -> lowest, etc.), when the data is read in.
